# Disease classification for whole-blood DNA methylation: Meta-analysis, missing values imputation, and XAI

**DOI:** 10.1093/gigascience/giac097

**Published:** 2022-10-19

**Authors:** Alena Kalyakulina, Igor Yusipov, Maria Giulia Bacalini, Claudio Franceschi, Maria Vedunova, Mikhail Ivanchenko

**Affiliations:** Institute of Information Technologies, Mathematics and Mechanics, Lobachevsky State University, 603022 Nizhny Novgorod, Russia; Institute of Information Technologies, Mathematics and Mechanics, Lobachevsky State University, 603022 Nizhny Novgorod, Russia; IRCCS Istituto delle Scienze Neurologiche di Bologna, 40139 Bologna, Italy; Institute of Information Technologies, Mathematics and Mechanics, Lobachevsky State University, 603022 Nizhny Novgorod, Russia; Institute of Biology and Biomedicine, Lobachevsky State University, 603022 Nizhny Novgorod, Russia; Institute of Information Technologies, Mathematics and Mechanics, Lobachevsky State University, 603022 Nizhny Novgorod, Russia

**Keywords:** DNA methylation, machine learning, data harmonization, explainable artificial intelligence

## Abstract

**Background:**

DNA methylation has a significant effect on gene expression and can be associated with various diseases. Meta-analysis of available DNA methylation datasets requires development of a specific workflow for joint data processing.

**Results:**

We propose a comprehensive approach of combined DNA methylation datasets to classify controls and patients. The solution includes data harmonization, construction of machine learning classification models, dimensionality reduction of models, imputation of missing values, and explanation of model predictions by explainable artificial intelligence (XAI) algorithms. We show that harmonization can improve classification accuracy by up to 20% when preprocessing methods of the training and test datasets are different. The best accuracy results were obtained with tree ensembles, reaching above 95% for Parkinson’s disease. Dimensionality reduction can substantially decrease the number of features, without detriment to the classification accuracy. The best imputation methods achieve almost the same classification accuracy for data with missing values as for the original data. XAI approaches have allowed us to explain model predictions from both populational and individual perspectives.

**Conclusions:**

We propose a methodologically valid and comprehensive approach to the classification of healthy individuals and patients with various diseases based on whole-blood DNA methylation data using Parkinson’s disease and schizophrenia as examples. The proposed algorithm works better for the former pathology, characterized by a complex set of symptoms. It allows to solve data harmonization problems for meta-analysis of many different datasets, impute missing values, and build classification models of small dimensionality.

## Introduction

### Background

DNA methylation (DNAm) plays an important role in human development and is associated with gene expression, genomic imprinting, and other biological processes without altering the DNA sequence [1–8]. Abnormal methylation patterns can lead to numerous diseases [9]. DNA methylation consists of binding a methyl group to cytosine in the cytosine–guanine dinucleotides (CpG sites). Hypermethylation of CpG sites near the gene promoter is known to repress transcription, while hypermethylation in the gene body appears to have an opposite, also less pronounced effect [10, 11]. Changes in DNAm patterns are associated with aging and environmental exposures [12,13]. Current epigenome-wide association studies (EWAS) test DNAm associations with human phenotypes, health conditions, and diseases [14–16]. Microarray-based technologies, such as the Illumina HumanMethylation450 (450K) and HumanMethylationEPIC (850K) arrays [17], are based on the hybridization of bisulfite-converted DNA to 50-mer probes and for each CpG site included in the design allow estimating the fraction of methylated DNA copies. Two metrics are used to represent methylation levels: the β-value, ranging from 0 to 1, and the M-value, the log_2_ ratio of the intensities of methylated versus unmethylated probes [18–20]. M-values are more robust quantifiers since β-values close to 0 and 1 suffer from substantial heteroscedasticity [18].

Nowadays, machine learning has become a broadly applicable method for data modeling and analysis in a wide range of applications. The availability of large data sets and a variety of unreinforced generative methods make these approaches more accurate, simple, and relevant in bioinformatics, particularly for transcriptomic and epigenetic data analysis [21–26]. DNA methylation data are often used for classification tasks. One of the most common examples is the classification of different types of cancer using The Cancer Genome Atlas (TCGA) repository [27]. Such classifiers usually demonstrate high accuracy [26, 28–34], based on both cancer-induced changes in methylation and the differences in methylation of various tumor tissues [28, 35]. Classifying different human conditions—phenotypes or pathologies—using DNA methylation data from a single tissue is more difficult. Phenotype classification can question smoking or obesity status, although existing results suggest that such conditions may not be clearly reflected in DNA methylation [26,36, 37]. Classification of cases and controls for certain diseases is also performed using DNA methylation data. Examples of machine learning applications using epigenetic data include classification of coronary heart disease, neurodevelopmental syndromes, schizophrenia, Alzheimer’s disease, psychiatric disorders, and others [38–44].

One of the main challenges is that methylation datasets are limited in the number of samples. Increasing the amount of data requires combining many datasets collected under different conditions and then performing analysis for the merged data, which can cause a variety of problems. There are many factors that lead to significant differences in methylation data that are not directly related to the development of pathological conditions—namely, the effect of the laboratory batch, different experimental conditions, normalization, and others [45].

Methylation levels can be affected by systematic variation due to biosample processing—that is, batch-related variability (a subset of samples processed simultaneously), chip position in batches, and sample position within the chip [46, 47]. Batch effects can dramatically reduce the accuracy of measurements and produce false-positive effects if the sample distribution is not uniform [48]. Most of the existing works avoid the question of the applicability of obtained models to new data. A central issue of meta-analysis is data harmonization. Sala et al. [49] developed an approach to systematically assess the impact of different preprocessing methods on meta-analysis. Its main advantage is the possibility of harmonization of the newly introduced datasets that does not require corrections to the previously analyzed datasets, employed for training the machine learning model.

Making use of new datasets to validate the model raises another problem: missing values in the data and the need to fill them in. New (test) datasets can lack information about some relatively small number of CpG sites on which the model was built. Experimental methylation data often contain missing values due to failing quality control checks, which can affect subsequent analysis. Since such missing CpG sites are necessary input parameters for the model, their values must be imputed. Examples include epigenetic clocks, which estimate biological age from small sets of preselected age-correlated CpG sites [5, 50–52], sensitive to small deviations in methylation levels [53]. Consequently, accurate imputation of missing data is required to improve the quality of DNA methylation analysis [54].

Dimensionality presents yet another problem. High data dimensionality is often associated with various undesirable consequences: increased computational effort, retraining, and visualization difficulties [55]. High-dimensional data may contain redundant information and introduce noise, while low-dimensional data may be sufficient for comprehensive data characterization. Since methylation data are multivariate, are continuous, and have nonlinear dependencies, traditional approaches often encounter the problem of multiple hypothesis testing and multicollinearity [26]. In addition, the most common epigenetic models [5,51, 56, 57] contain a small number of variables to simplify data processing, for better interpretation of the results and for the possibility of applying these models in real life. It is also worth noting that small DNA methylation panels are significantly less costly [58], which is an undeniable advantage for the possibility of widespread use.

Modern artificial intelligence (AI) systems based on machine learning are powerful and promising tools in a wide range of applications, from computer vision, machine translation, and speech recognition [59–61] to the analysis of biomedical data, particularly DNA methylation [26, 62, 63]. However, while these models provide impressive predictive accuracy, their nonlinear structure makes them poorly interpretable (i.e., it is hard to explain what information in the input data leads AI to particular outputs). The need for trustworthy solutions has recently attracted much attention to methods that would “open” black box models [64–74]. This includes developing methods to help better understand what the model has learned [75, 76] as well as methods to explain individual predictions [65–67,77, 78].

In summary, individual DNA methylation datasets contain an insufficient number of samples to apply machine learning approaches, so there is a need to combine and harmonize different datasets. Problems that arise on the way include tackling batch effects in individual datasets, missing values for certain samples, and high data dimensionality. Here, we analyze several existing fragmented solutions to these problems, develop a generalized unifying approach integrated in a workflow, validate it, and demonstrate its efficiency.

### Study design and novelty

Our primary goal is to offer a methodologically complete workflow for building machine learning models, as well as classifying cases and controls for various diseases from whole-blood DNA methylation data on many datasets, ranging from data harmonization to explainable AI models. DNA methylation data are taken from different human body tissues, but the most widespread is whole-blood methylation, the least invasive analysis and, therefore, of broad diagnostic prospects. We restrict our analysis to this kind of data. Our workflow solves a problem of harmonization of methylation data from different datasets. They are collected in different laboratories, with different setups and experimental conditions. In general, the data are of different quality and have been preprocessed differently. Harmonization is used to eliminate the unavoidable bias between the data and to minimize the associated machine learning model errors. The proposed workflow uses harmonization with the selection of a reference dataset, in which case all other datasets are aligned with the reference one, so that when a new dataset is introduced, there is no need to renormalize the training data and hence rebuild the model. The workflow uses the generally recognized types of machine learning models for classification on methylation data in tabular representation, particularly gradient-boosted decision trees. A hyperparametric search for the optimal combination of the parameters of these models is performed to ensure the best classification accuracy. Next, the dimensionality of the feature space is reduced to build portable models. In such models, the number of features has the same order as the most popular epigenetic models, such as the Horvath clock (353 CpG sites) [5], Hannum clock (71 CpG sites) [51], DNAm PhenoAge (513 CpG sites) [56], and DNAm GrimAge (1,030 unique CpGs were used to predict plasma protein levels) [57]. Such portable models allow them to be used for early diagnosis of various diseases—analysis of small CpG panels is much cheaper than full-genome analyses. Reducing the dimensionality of the data can also help discard noisy features that do not carry relevant information for classifiers. Also, the proposed approach includes the possibility of imputing missing values (CpG sites), and different approaches are used for this purpose. This is especially important when testing the model on new data, where some CpG sites critical for the model may be missed (e.g., because of technical errors in data acquisition and processing or failing quality checks). For the best models in terms of accuracy, explainable artificial intelligence (XAI) methods are applied to explore the global influence of individual CpG sites on model predictions and to get explanations of how the methylation-level values of individual CpG sites for specific subjects shape their individual predictions. Lists of the most important CpG sites in terms of machine learning models are compared with lists of CpG sites (and their corresponding genes) from existing studies associated with the considered diseases. Biological pathways of diseases based on these lists are identified and investigated.

## Results

**Figure 1.**
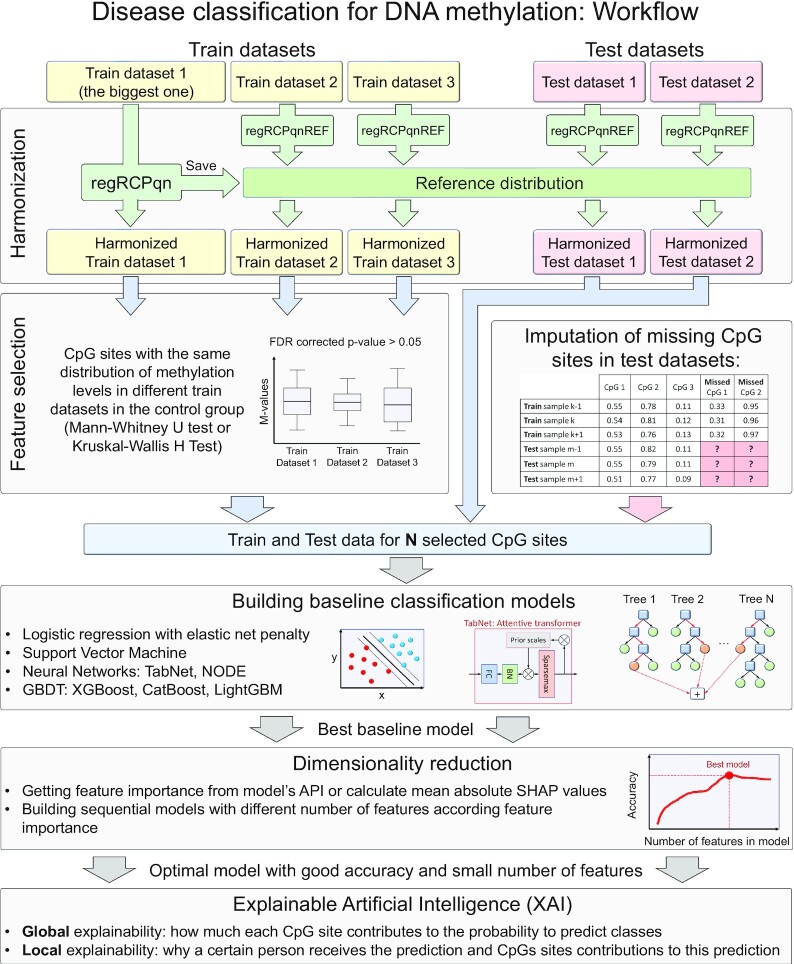
Workflow for classifying cases and controls of various diseases based on DNA methylation data proposed in this article.

Larger training sample sizes provide better quality of machine learning models. The currently available DNA methylation data sets do not exceed several thousand samples, and that could hardly change in the near future due to complexity and cost of study. Merging different datasets, therefore, appears a practical way to circumvent size limitations. However, it poses many challenges, such as the need to harmonize datasets collected under different conditions and preprocessed in different ways, the need to fill in missing values in a way that preserves patterns in the data, and the reduction of excessively high dimensionality of input variables with a relatively small number of samples. These issues have been addressed separately; below we report an integrated solution that brings together the data processing and analysis steps and the resulting methodologically complete workflow for solving the classification problem based on merging several independent DNA methylation data. A schematic representation of the proposed workflow is shown in Fig. 1.

### Datasets and machine learning tasks

We studied whole-blood DNA methylation datasets generated on patients with Parkinson’s disease or schizophrenia. We selected 3 datasets that contain samples from patients with Parkinson’s disease and healthy controls—GSE145361 [79], GSE111629 [80–82], and GSE72774 [80,81, 83]—and 4 datasets that contain samples from patients with schizophrenia and healthy controls: GSE152027 [84], GSE84727 [84, 85], GSE80417 [84, 85], and GSE116379 (nonfamine participants) [86]. Information about considered datasets is summarized in Table 1, in particular, the number of cases and controls, whether the dataset has been used as a train or test, the original preprocessing type, and the number of CpGs.

**Table 1.** Main characteristics of considered datasets. For each disease, the bold row represents the reference dataset for the harmonization. Number of CpGs is common for train datasets in each disease. Three largest datasets for schizophrenia, GSE84727, GSE80417, and GSE152024, have the same preprocessing.

Disease	Dataset	Number of cases	Number of controls	Train or test subset	Raw IDAT available?	Number of CpGs	Original preprocessing
Parkinson’sdisease	**GSE145361**	**959**	**930**	**Train**	**Yes**	**411,761**	**Data processing: Genome Studio software**
	GSE111629	334	237	Train	Yes		Data processing: R software v3.4.2Functional normalization: minfi R package
	GSE72774	289	219	Test	No	411,979	Data processing: BeadStudio software v3.2
Schizophrenia	**GSE84727**	**414**	**433**	**Train**	**No**	**399,625**	**Importing: methylumi R package** **methylumIDAT function** **Preprocessing: watermelon R package** **pfilter and dasen functions**
	GSE80417	353	322	Train	No		Importing: methylumi R packagemethylumIDAT functionPreprocessing: watermelon R packagepfilter and dasen functions
	GSE152027	290	203	Test	No	411,901	Importing: methylumi R packagemethylumIDAT functionPreprocessing: watermelon R packagepfilter and dasen functions
	GSE116379	51	54	Test	No	407,781	Removed: X and Y chromosomes,nonspecific binding probes,failed probes based on a detection *P*value>0.001 and bead count <5, probes withsingle-nucleotide polymorphisms of minor allele frequency >5% within10 base pairs of the primerFunctional normalization: minfi R package

For each disease, we built machine learning models to classify cases versus controls. Some of these datasets are used as train data for building the model, and the rest is used to test the model. For each disease, we selected a reference dataset, against which harmonization was performed. As can be seen from Table 1, the original preprocessing is the same for the majority of the considered datasets with schizophrenia patients, but it varies considerably among the different datasets for Parkinson’s disease. To reduce the influence of the laboratory-specific data collection and processing conditions on classification results, harmonization is necessary.

### Meta-analysis and harmonization

**Figure 2.**
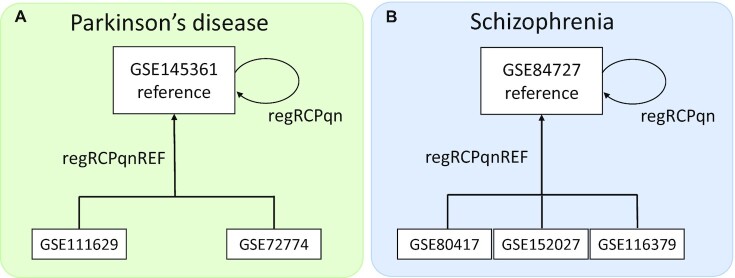
Schematic representation of harmonization procedure for (A) Parkinson’s disease and (B) schizophrenia datasets.

Combining different DNA methylation datasets can improve the statistical power to test hypotheses and identify epigenetic signatures by meta-analysis. However, such meta-analysis also poses serious problems related to data harmonization, which is often not considered [87]. This is especially true for DNA methylation, where data are often only available in the preprocessed rather than raw form, and where diverse preprocessing pipelines are used [49]. Developed in [49], approach regRCPqn (regional regression on correlated probes with quantile normalization) allows for meta-analysis even if the raw data are not available. Importantly, as emerging datasets are aligned, the already treated datasets do not require renormalization. Therefore, we apply this approach to harmonization with reference. The largest dataset for each disease is taken as the reference, and other datasets are harmonized relative to it. The schematic representation of the harmonization process is shown in Fig. 2.

**Figure 3.**
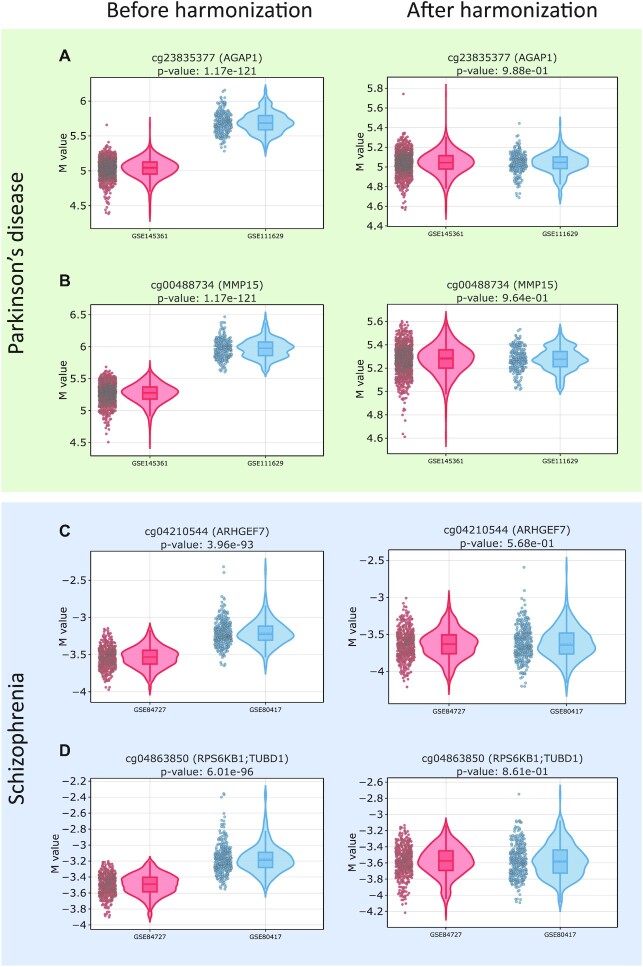
Examples of M-value methylation level distribution for control groups before and after harmonization for Parkinson’s disease examples (A) cg23835377 and (B) cg00488734 and schizophrenia examples (C) cg04210544 and (D) cg04863850.

For machine learning models, we used only those CpG sites that have the same distribution of methylation levels in different train datasets in the control group (methylation levels in the case group typically have greater variability because of disease heterogeneity). We used the Mann–Whitney *U* test [88] to compare DNA methylation values of healthy participants from the considered train datasets before and after harmonization. After harmonization, the number of CpG sites with the adjusted *P* > 0.05 (not significantly different between healthy subjects from the considered train datasets) increased from 43,019 to 50,911 for Parkinson’s disease and from 35,145 to 110,137 for schizophrenia. Fig. 3 illustrates the change in the distributions of methylation level values before and after harmonization. In particular, CpG sites whose methylation level distributions differed significantly before harmonization (false discovery rate [FDR]–corrected *P* < 0.05) manifest similar distributions after harmonization (FDR-corrected *P* > 0.05).

### Classification models

The most common type of data representation for machine learning is tabular, and DNA methylation data fulfill it. Typically, the rows refer to participants, the columns refer to CpG sites, and the cells of the table contain the methylation levels of each CpG site for each participant. There are many machine learning models designed to work with tabular data: logistic regression with elastic net penalty [89], support vector machine [90], XGBoost [91], CatBoost [92], LightGBM [93], TabNet [94], and NODE [95]. Main characteristics of the models are summarized in Table 2.

**Table 2.** Main characteristics of the considered classification models

Model	Type	Feature importance API
Logistic regression	Generalized linear model	Yes
Support vector machine	Supervised learning model constructing the separating manifold	Only for linear kernel
XGBoost	Gradient-boosted decision tree ensemble	Yes
CatBoost	Gradient-boosted decision tree ensemble	Yes
LightGBM	Gradient-boosted decision tree ensemble	Yes
TabNet	Deep neural network	Yes
NODE	Gradient-boosted decision tree ensemble	No

For each disease, all considered datasets were divided into training and test ones (as stated in Table 1). We trained all models on 2 training datasets and then tested on the remaining datasets. Accuracy with weighted averaging was the main quality metric, as it can handle situations with possible imbalance of the classes (the number of participants in different classes varies significantly). As discussed above, the approach fulfills the requirement that the model does not have to be trained again as the new data set is considered. Moreover, the models must be trained to classify biological differences in methylation data rather than traces of different experimental conditions in different laboratories. Accordingly, we do not mix train and test datasets and do not perform cross-validation. To find the optimal combination of model parameters that provides the best accuracy, we used a hyperparametric grid search (the values are presented in Supplementary Table S1).

Newly introduced datasets may lack some CpG sites that are present in already trained models; in this case, various imputation methods are applied (cf. Imputation of Missing Values and Methods sections for more details). Models for Parkinson’s disease are trained on 43,019 CpG sites for nonharmonized data and on 50,911 CpG sites for harmonized data. Among these, the Parkinson’s disease test dataset GSE72774 lacks 38 CpG sites in the nonharmonized data and 34 CpG sites in the harmonized data. Models for schizophrenia train on 35,145 CpG sites for nonharmonized data and on 11,0137 CpG sites for harmonized data. The first test dataset for schizophrenia, GSE152027, lacks 9 CpG sites in the nonharmonized data and 36 CpG sites in the harmonized data. The second test dataset for schizophrenia, GSE116379, lacks 268 CpG sites in the nonharmonized data and 609 CpG sites in the harmonized data. These missed CpG sites are imputed using K nearest neighbor (KNN) methods with K = 1. However, this imputation does not have a significant effect on the result because, as will be shown later, all the missed CpG sites are not at the top of the features in terms of importance.

Table 3 shows the results of cases versus controls classification by baseline models based on nonharmonized and harmonized whole-blood methylation data for Parkinson’s disease and schizophrenia on test datasets. For each combination of harmonization type, disease, and test dataset, the best weighted accuracy values for all constructed models are given. All imputation methods described in the Imputation of Missing Values section do not significantly change the quality of the resulting models, because all missed CpGs in test datasets have a very low value of feature importance in models with corresponding API.

**Table 3.** Binary classification results of baseline models for nonharmonized and harmonized data. For Parkinson’s disease and schizophrenia, results comparing the accuracy of different models for nonharmonized and harmonized methylation data are shown.

Model	Parkinson’s disease		Schizophrenia			
	GSE72774		GSE152027		GSE116379	
	Nonharmonized	Harmonized	Nonharmonized	Harmonized	Nonharmonized	Harmonized
Logistic regression	0.71	0.93	0.63	0.66	0.56	0.66
Support vector machine	0.67	0.92	0.62	0.66	0.58	0.65
XGBoost	0.72	0.95	0.67	0.71	0.56	0.66
CatBoost	0.71	0.94	0.68	0.72	0.59	0.71
LightGBM	0.76	0.97	0.68	0.71	0.58	0.67
TabNet	0.69	0.93	0.63	0.66	0.58	0.65
NODE	0.71	0.92	0.62	0.66	0.56	0.65

The results confirm that harmonization must be applied and is most efficient for the datasets with different preprocessing methods. In particular, for Parkinson’s disease, all datasets have different original preprocessing, and the best model trained on such data shows a result of 76%. When these data are harmonized, accuracy improves dramatically to 97%. For both nonharmonized and harmonized data, for Parkinson’s disease, the best model in terms of weighted accuracy is LightGBM. For schizophrenia, only 1 of 4 datasets has a different preprocessing (GSE116379, Table 1). Then, harmonization does not significantly affect the quality of the built models if the datasets have the same preprocessing (68% without harmonization, 72% with harmonization in the best models). The best model is LightGBM for nonharmonized data and CatBoost for harmonized data. However, applying models trained on nonharmonized data to data with a different preprocessing gives a poor result for binary classification: 59%. Harmonization of data improves the performance of the trained models, making them close to the best results obtained for schizophrenia in terms of quality: 71%. It is also worth noting that the overall classification quality for these 2 diseases on methylation data is very different, possibly due to the different etiology and molecular mechanisms involved in the 2 diseases.

**Figure 4.**
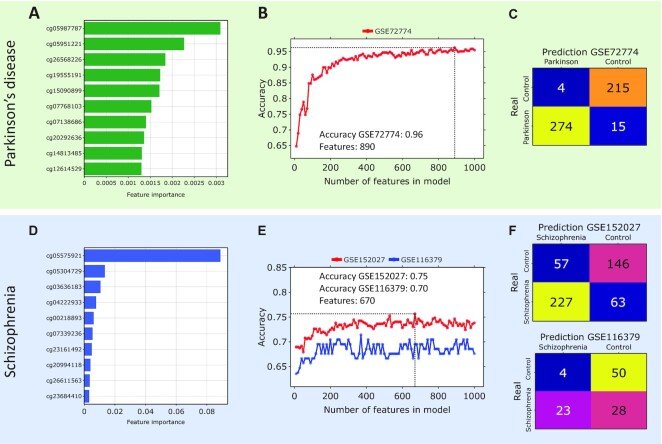
Dimensionality reduction for the best classification models. Parkinson’s disease: (A) Top 10 features for the best classification model LightGBM with the normalized importance values. (B) Dependence of the weighted accuracy on the number of features in the model. Dotted line corresponds to the optimal small model with accuracy value of 0.96 and 890 features. (C) Confusion matrix for optimal model. Schizophrenia: (D) Top 10 features for the best classification model CatBoost with the normalized importance values. (E) Dependence of the weighted accuracy on the number of features in the model. Dotted line corresponds to the optimal small model with accuracy value of 0.75 for GSE152027, 0.70 for GSE116379, and 670 features. (F) Confusion matrix for optimal models.

Best accuracy models allow us to extract importance values for all features. The ranking of the most important features for these models for Parkinson’s disease and schizophrenia is shown in Fig. 4. It is worth noting that for schizophrenia, there is 1 outstanding CpG with the highest importance for classification, while the others have much lower values. For Parkinson’s disease, the situation is more uniform. These rankings can be used for the dimensionality reduction of the built models.

### Dimensionality reduction

As a result of applying different baseline models to methylation data to classify cases versus controls, the ones with an API for feature extraction showed the best accuracy. Based on the obtained ranking of the features (Fig. 4), we performed dimensionality reduction of the constructed models. Most common epigenetic models comprise few CpG sites, no more than several hundred (for example, those used to predict epigenetic age like Horvath clocks and Hannum clocks). First, models based on few features show significantly better performance while maintaining similar classification accuracy. Second, such models are less memory-consuming.

Along these lines, we reduced the dimensionality of the model, leaving only the most important features for classification. Fig. 4 shows the dependence of weighted classification accuracy on the number of features in the model for Parkinson’s disease and for schizophrenia. It first increases, until reaching a certain optimal number of features, and then changes weakly. For Parkinson’s disease, the best weighted accuracy of 96% is observed for 890 CpG sites, with an accuracy value changed by only 1% compared to the full data (50,911 CpG sites). For schizophrenia, the best weighted accuracy of 75% is observed for 670 CpG sites for the test dataset GSE152027 and 70% for the test dataset GSE116379. The optimal model for schizophrenia was chosen as the one for the GSE152027. The accuracy values changed by no more than 3% compared to the full data (110,137 CpG sites). The list of CpG sites that make up these small models, as well as basic information about them (gene, chromosome, relation to the CpG island), is presented in Supplementary Table S2. The resulting CpG lists were compared with previously published lists of biomarkers associated with Parkinson’s disease [80, 96, 97] and schizophrenia [84, 98]. Interestingly, for Parkinson’s disease, there is practically no overlap with the previous results, except for 1 CpG site from [96]. This CpG belongs to the gene *DYNC1H1*, which is associated with neurological and neurodegenerative diseases [99, 100]. For schizophrenia, only 15 CpG sites are common with [84]. Some of the connected genes like *PRKCZ*, *SHANK2*, *ZNF608*, and *PRDM16* were also identified as schizophrenia risk factors [101–104]. Genes, corresponding to CpG sites, from the optimal small model for Parkinson’s disease were enriched in several gene ontologies related to neuronal and metabolic processes, whereas genes from schizophrenia models were enriched in gene ontologies related to cell development processes (Supplementary Table S3).

### Imputation of missing values

For trained machine learning models (both large and small), it is important that there are no missing values in the upcoming test data. Since it is impossible to guarantee their absence, various imputation methods are used to fill them in. Since not all models support data imputation, we use the most popular of them: mean, median, mode, random, chained equations, expectation maximization, and KNN with different numbers of neighbors (from 1 to 3). To study the effect of these imputation methods on the classification accuracy, we consider the following simulation experiment. For each disease, we consider only the best small models, obtained at the previous step (LightGBM with 890 CpG sites for Parkinson’s disease and CatBoost with 670 CpG sites for schizophrenia). For these models, we “remove” 100 CpG sites with the highest importance values and impute them. The number of CpG sites was chosen to induce a significant drop in accuracy and to sharpen the differences in efficiency between the imputation methods. The missing CpG sites do not take part in the construction of small optimal models, so the actually existing CpG sites are removed from consideration. Table 4 shows results for the considered test datasets. For Parkinson’s disease, KNN with 1 neighbor kept the classification accuracy at the baseline level of the data without missing values. Imputation with mode also showed good results, losing only 3%. The other methods achieved an accuracy of no more than 90%. For schizophrenia, none of the approaches achieved the baseline accuracy for data without missing values. This may be explained by the critical importance of specific features for classification. KNN with 1 neighbor for both datasets shows one of the best imputation results; for GSE152027, chained equation and expectation maximization perform better than KNN by 3% and 2%, respectively. Median and random values methods show unsatisfactory results in all experiments.

**Table 4.** Comparison of different missing value imputation methods and their effect on weighted classification accuracy for Parkinson’s disease and schizophrenia. In all cases, 100 CpG sites with the highest importance values were dropped.

Model	Parkinson’s disease	Schizophrenia	
	GSE72774	GSE152027	GSE116379
**Original (n** **o missed data)**	**0.96**	**0.75**	**0.7**
Mean	0.78	0.65	0.67
Median	0.81	0.59	0.55
Mode	0.93	0.55	0.54
Random	0.86	0.62	0.54
Chained equation	0.89	0.68	0.61
Expectation maximization	0.87	0.67	0.54
KNN (K = 1)	0.96	0.65	0.67
KNN (K = 2)	0.9	0.65	0.67
KNN (K = 3)	0.86	0.65	0.67

### Explainable artificial intelligence

Even the most accurate machine learning models make mistakes on upcoming data. It presents a major challenge for those models that work as “black boxes” with unknown principles behind made decisions. SHapley Additive exPlanations (SHAP) help to understand why the model makes its predictions from the global and local points of view [105].

**Figure 5.**
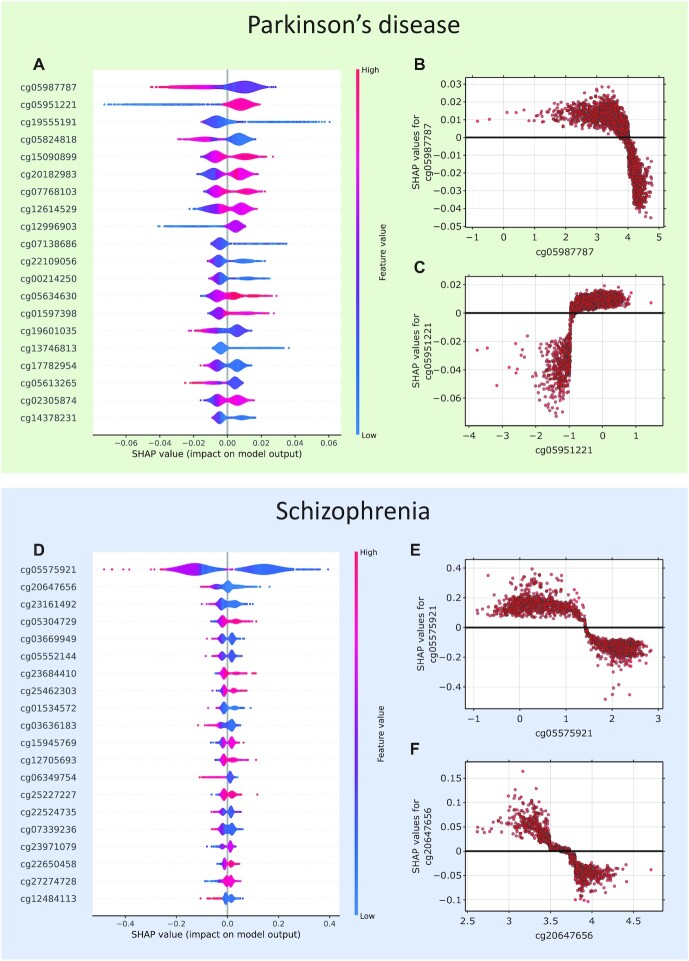
Global explainability based on SHAP values. Parkinson’s disease: (A) Beeswarm plots show the dependence of SHAP values for each CpG site on their methylation levels. Each dot represents 1 participant. (B) Dependence of SHAP values on methylation M-values for cg05987787. The black line separates the areas of negative and positive influence of SHAP values on the probability of predicting Parkinson’s disease. (C) Dependence of SHAP values on methylation M-values for cg05951221. Schizophrenia: (D) Beeswarm plots show the dependence of SHAP values for each CpG site on their methylation levels. Each dot represents 1 participant. (E) Dependence of SHAP values on methylation M-values for cg05575921. (F) Dependence of SHAP values on methylation M-values for cg20647656.

The global explainability of the constructed models on the training data for Parkinson’s disease and schizophrenia is illustrated in Fig. 5. Beeswarm plots show the relationship between SHAP values and methylation levels for the most important CpG sites. For each CpG site, the distributions of methylation levels for all participants are shown. In particular, for Parkinson’s disease, most of the participants in the CpG site cg05987787 have low methylation levels, which positively affects the probability of predicting the disease. The scatterplots show in detail the distribution of methylation levels in different participants and SHAP values. We can see that M-values below 4 have a positive effect on the probability of predicting disease, while M-values above 4 have a negative effect. The black line divides the areas of positive and negative influence of SHAP values on the prediction of disease probability. The opposite situation is observed for the CpG site cg05951221. M-values below −1 have a negative effect on the probability of predicting disease, while M-values above −1 have a positive effect. Similar plots are shown for schizophrenia. The beeswarm plot shows that there is one most important CpG site, cg05575921, that contributes the most to the probability of predicting disease, as previously shown, and the other CpG sites have a much smaller effect.

**Figure 6.**
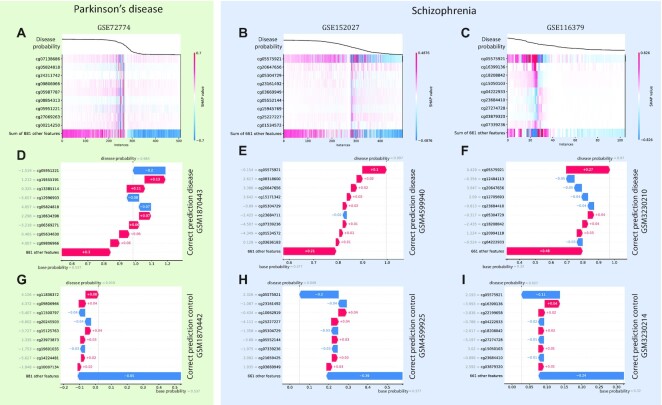
Local explainability based on SHAP values. (A) Black lines show the probability of predicting Parkinson’s disease for different participants. Heatmaps in color show the contribution of SHAP values to the probability of predicting Parkinson’s disease for different participants and CpG sites for GSE72774. (B) Probability of predicting schizophrenia and heatmaps of the contribution for GSE152027. (C) Probability of predicting schizophrenia and heatmaps of the contribution for GSE116379. (D) Waterfall plot for participant GSM1870443 with Parkinson’s disease, showing the contribution of individual CpG sites to changes in the probability of predicting disease. (E) Waterfall plot for participant GSM4599940 with schizophrenia. (F) Waterfall plot for participant GSM3230210 with schizophrenia. In all cases, the models’ confidence is over 97%. (G) Waterfall plot for control participant GSM1870442 showing the contribution of individual CpG sites to the change in probability of predicting disease. (H) Waterfall plot for control participant GSM4599925. (I) Waterfall plot for control participant GSM3230214. In all cases, the probability of predicting disease is below 5%.

The local explainability of predictions on the test data is shown in Fig. 6. The top row presents heatmaps with participants on the x-axis, CpG sites on the y-axis, and SHAP values encoded on a color scale. The participants are ordered based on the probability to predict the disease. Model output is shown above the heatmap matrix. The black line represents the probability of predicting the disease for each participant. It follows that for Parkinson’s disease, where the model works with high accuracy, this line is quite smooth and similar to the softmax function. Whereas for schizophrenia, for which the models have much lower accuracy, these probability plots are more fragmented. As shown earlier, for schizophrenia, 1 CpG site is the most important, so it has the highest absolute SHAP values and appears the brightest in the heatmaps. The center and bottom lines represent waterfall plots for participants with the disease and controls, respectively. They allow for explaining the model output for each participant separately. The bottom part of the waterfall plot shows the base probability of the model to predict disease, and then each line shows how a positive (red) or negative (blue) contribution from each CpG site moves the probability to the model output for that prediction. The output of the model is the probability of predicting disease. If the probability is greater than 50%, the model identifies the participant as a case; otherwise, it identifies the participant as a control. The baseline probability is the average probability of the model predicting on the test data. Because baseline probability is a characteristic of the model, it depends on the quality of the model. If the model has reasonably good accuracy, then the base probability is close to the proportion of participants in a particular class. In the examples from middle line of the Fig. 6 for all participants with diseases, the probability of predicting disease in the examples was above 97% (models identify them as cases almost for sure); for control participants (bottom line of Fig. 6), the probability of predicting disease was below 5% (models identify them as controls almost for sure).

## Discussion

### Conclusion

We developed a multifunctional workflow for applying machine learning models to classify cases and controls for different diseases based on DNA methylation data. Specifically, we considered Parkinson’s disease and schizophrenia as examples of complex diseases requiring early diagnosis. In addition, for these diseases, there are large publicly available whole-blood DNA methylation datasets. In this article, the task of classifying cases and controls based on whole-blood DNA methylation data with harmonization, missing value imputation, model dimensionality reduction, and application of XAI approaches is solved for the first time for Parkinson’s disease and schizophrenia. In [106], the problem of pairwise classification of neurodegenerative diseases cases (in particular, for Parkinson’s disease and schizophrenia) was solved. The authors did not use methylation levels; instead, they used methylation-derived profile scores as features; however, we do not compare diseases with each other in this work. In [40], the problem of classifying cases and controls for schizophrenia was solved. In that work, instead of methylation levels, other metrics were also used: CoRSIV probes with a polygenic risk score. The article presents the results of positive predictive values only for cases, not for controls, so it is not possible to directly compare the results.

The first step of the workflow is to harmonize the data according to the chosen reference dataset. We have shown that harmonization works well also when the available datasets were preprocessed using different pipelines and tools, as it often occurs. Harmonization can increase the classification accuracy by up to 20%. This is fully consistent with the original study, which proposed the harmonization method regRCPqn [49]. When the preprocessing of training and test data is the same, harmonization has almost no effect on the final classification accuracy. It is impossible to guarantee that all new datasets on which the model will be tested will have the same preprocessing. Even with tools such as limma [107] and ComBat [108], it may not be possible to remove technical signal when batches are mixed with variables of interest. Applying ComBat to high-throughput data with an uneven study design may actually result in false signals [109]. To solve the classification problem, different models were tested: classical ones (logistic regression and support vector machine), different gradient-boosted decision trees, and deep neural networks. For all models, a hyperparametric search was performed to select the optimal set of parameters. The best results of weighted accuracy were obtained with tree ensembles, which is consistent with other works [110]. For Parkinson’s disease, the accuracy of classifying patients and healthy controls was higher than 95%. The accuracy for schizophrenia was much lower, only >70%. Unlike Parkinson’s disease, schizophrenia is a complex disease characterized by a variety of different symptoms. A large number of different causes and molecular patterns determining the development of this pathology are being identified. The etiology of schizophrenia is multifactorial and reflects an interaction between genetic vulnerability and environmental influences. Environmental risk factors, such as complications of pregnancy and childbirth, childhood trauma, migration, social isolation, urban life, and substance abuse, either singly or in combination over time, affect the likelihood of an individual developing the disorder. A lot of genes have been previously identified as responsible for the risk of developing this pathology, so the personal molecular landscape is highly individualized and complicated by interactions between genes and the environment. These reasons may be related to the relatively low accuracy for schizophrenia in our study compared to Parkinson’s disease [111–113]. Another reason that affects the result may be the number of samples in the training set. The training set for schizophrenia has fewer samples compared to Parkinson’s disease. In this case, the data may not be representative enough to fully describe the disease characteristics for the model. Since the best models allow us to obtain the importance values of each feature for classification, we constructed a CpG site ranking for each of the diseases. Based on this ranking, we reduced the dimensionality of the classification models, since many of the most common epigenetic models (such as the epigenetic clocks) contain relatively few CpG sites (up to 1,000). To find optimal small models, we performed a series of experiments for an increasing number of features from 10 to 1,000. Based on the dependence of the weighted classification accuracy on the number of features, we determined the optimal models, with the accuracy for both diseases changing not much, while the number of features decreased significantly. Optimal small models contain 890 CpG sites for Parkinson’s disease and 670 CpG sites for schizophrenia. Such small models are much less memory-consuming and have better computational performance. For machine learning models (both small and large), missing values are critical. A model trained on a particular set of features must necessarily be tested on the same set. If, for certain reasons (data collection or processing errors, low signal intensities, or other problems), some CpG sites are missing from the new data for testing, imputation methods are applied. The best imputation methods recover almost the same classification accuracy as for the original data. Since machine learning models work as a “black box,” special methods must be used to explain exactly how the model makes predictions. Otherwise, the model cannot be trusted, and it is impossible to identify the nature of errors. If the predictive capabilities of the model can be explained, it can help to discover complex relationships between biomarkers. The calculation of SHAP values allowed us to obtain both global and local explainability. Globally, the prediction of a healthy control or patient with Parkinson’s disease is affected by several CpG sites, and there are examples of both types of influence, positive and negative. Global explainability for healthy controls and patients with schizophrenia confirmed the strongest influence of 1 CpG site, cg05575921, while the others had little effect on model prediction. This CpG site has previously been reported to be associated with schizophrenia and posttraumatic stress disorder [85, 114]. Examples of local explainability—specific predictions for certain participants— are also given.

Thus, we propose a methodologically valid and complete approach for classifying healthy people and patients with various diseases, which allows to harmonize DNA methylation data from different sources, impute missing values, reduce dimensionality of the models, and apply explainable artificial intelligence approaches. The proposed algorithm works better for Parkinson’s disease than for schizophrenia, which is characterized by a variety of different symptoms. Further work may include expanding the pool of considered diseases, enriching the library of methods at different stages of the workflow. Another future challenge is advancing from explainability to interpretability. The former, currently implemented, uncovers the internal “mechanics” of a system. The yet missing interpretability would predict the outcome of changing the input or algorithmic parameters.

### Limitations

The proposed approach has several limitations relevant at different steps. First, it should be noted that the accuracy gain from harmonization will be limited if the preprocessing of training and test data is the same. Second, the constructed classification models may not be globally optimal in terms of quality metrics, because we are considering a limited number of parameters to vary within a hyperparametric search, each varying within a limited range of values near defaults. Third, besides choosing the top best features to reduce dimensionality, it might be better to consider different combinations of them. However, in this case, the number of considered models would increase dramatically. Fourth, using schizophrenia as an example, it was shown that if there are some most important CpG sites significantly overcoming the importance of the other, and if they are missed, imputation cannot help to improve the result.

## Methods

### Datasets

We reviewed publicly available whole-blood DNA methylation datasets in the GEO repository [115], which include the largest ones from patients with Parkinson’s disease and schizophrenia, with at least 50 participants in each group. The following datasets comprise whole-blood samples from subjects with Parkinson’s disease and healthy controls: GSE145361 [79], GSE111629 [80–82], and GSE72774 [80, 81, 83]. The following datasets comprise whole-blood samples from patients with schizophrenia and healthy controls: GSE152027 [84], GSE84727 [84, 85], GSE80417 [84, 85], and GSE116379 (only nonfamine participants) [86]. We remove from the analysis non-CpG probes [116], single-nucelotide polymorphism–related probes [117], multihit probes [118], and probes on chromosomes X and Y. We consider only common CpGs in train datasets, and the number of CpGs in test datasets can be different. The remaining number of CpGs after all filtration procedures is shown in Table 1.

### Data harmonization

Meta-analysis can be done in different ways: some approaches first analyze different datasets separately and then combine the results into a final estimate; others first combine data from all sets and then analyze the combined data using a single model. The first class of approaches includes aggregated data and 2-step meta-analysis of individual participant data (IPD) [119]. The major advantage of these approaches is their relatively low implementation complexity, while the major disadvantage is the need for raw data. This approach often uses not all available data, but only a subset (usually differentially methylated positions [DMPs] or differentially methylated regions [DMRs]). The second class of approaches is called single-step IPD meta-analysis. Although single-step IPD approaches are expected to behave similarly to 2-step IPD [119], they provide additional flexibility (e.g., no need to start with raw data) and enable comparison between different models [119]. An important assumption of single-step IPD meta-analysis is the comparability of variables measured in different datasets [120], and therefore data harmonization is crucial to ensure that methylation samples of the same type (same tissue, health status, age, gender, etc.) from different datasets can be compared.

We use the approach to data harmonization proposed in [49], a 1-step IPD approach for systematically assessing the impact of different preprocessing methods on the meta-analysis. It has been shown that data preprocessing by different algorithms has a significant impact. RegRCPqn (regional regression on correlated probes with quantile normalization) does not require raw idat files and can be applied to datasets with only β-values or M-values available, which is a common scenario from real life [49]. RCP [121] is a within-array normalization that uses the spatial correlation of DNA methylation at CpG sites to estimate the calibration transformation between type I and type II intensities. The regRCPqn procedure improves the RCP algorithm by including 3 functions to solve the problem under study. First, it calculates the RCP normalization separately for each type of genomic region (i.e., for CpG belonging to islands, shores, shelves, or open seas) because the distribution of DNA methylation values is different in each of these types of regions [122]. It then performs a quantile normalization between samples, in which CpG values for all samples are normalized separately for each CpG region and for type I and type II probes. Finally, it introduces the possibility of storing the reference distribution and using it to perform quantile normalization of samples from another dataset based on the reference. The reference distribution is calculated separately for each area type and for type I and type II probes. When possible, this distribution is used by regRCPqn to perform normalization based on the reference, again separately for each region and probe type. The dataset with the highest number of participants for each disease is used as the reference, and the others are harmonized relative to it. We consider CpG sites whose distribution of methylation levels does not differ in different train datasets in the control group. To find them, we performed the Mann–Whitney *U*test [88] for all CpG sites included in the train datasets for the control group and took CpG sites for which the *P* value adjusted according to the Benjamini–Hochberg procedure [123] was >0.05. The Mann–Whitney *U*test was performed using the scipy package version 1.8.0.

### Classification models

The most common type of data for machine learning and deep learning tasks is tabular data, which comprise samples (rows) with the same set of features (columns). DNA methylation is an example of this type of data. Tabular data, unlike image or speech data, are heterogeneous, resulting in dense numerical and sparse categorical features. In addition, the correlation between features is weaker than the spatial or semantic relationship in image or speech data [124]. Variables can be correlated or independent, and features have no positional information. Consequently, it is necessary to detect and use correlation without relying on spatial information [94,95]. During the past decade, traditional machine learning methods such as gradient-boosted decision trees (GBDT) [91] have continued to dominate tabular data modeling and have demonstrated better performance than deep learning [110]. GBDT trains a series of weak learners to predict the outcome. In GBDT, the weak learner is a standard decision tree that lacks differentiability. Despite their differences, their performance on many problems is similar [92]. When deep neural networks are applied to tabular data, many problems arise, such as lack of locality, missing values, mixed object types (numeric, ordinal, and categorical), and lack of prior knowledge about the structure of the data. Tree ensemble algorithms are considered a recommended option for real-world problems with tabular data [91, 92, 125]. The XGBoost algorithm [91] is an extendible gradient boosting tree algorithm that achieves state-of-the-art results on many tabular datasets [126, 127].

We consider the following classification models: logistic regression with elastic net penalty [89], support vector machine [90], XGBoost (XGBoost, RRID:SCR_021361) [91], CatBoost (Catboost, RRID:SCR_021694) [92], LightGBM (LightGBM, RRID:SCR_021697) [93], TabNet [94], and NODE [95]. Despite the name, logistic regression is used to solve the binary classification problem. It is a generalized linear model, showing good results for linearly separable data. Support vector machine is a supervised learning model whose main goal is to construct a separating manifold. This method allows the use of different kernel functions to achieve the best results. XGBoost (Extreme Gradient Boosting) is a scalable, distributed GBDT machine learning library. GBDT iteratively trains an ensemble of shallow decision trees, with each iteration using error residuals from the previous model to fit the next model. The final prediction is a weighted sum of all tree predictions. XGBoost has one of the best combinations of prediction performance and processing time. CatBoost is an open-source gradient boosting algorithm, which builds symmetric (balanced) trees. At each step, the leaves of the previous tree are separated by the same condition. A feature-split pair is selected and used for all nodes, which provides the least losses. This balanced tree architecture reduces prediction time and controls overfitting. LightGBM is a fast, distributed, high-performance gradient boosting platform that supports the decision tree algorithm. It splits the tree by leaf with the simplest fit, whereas other boosting algorithms split the tree by depth or by level rather than by leaf. Thus, when growing on an equivalent leaf in LightGBM, a leaf-based algorithm can reduce more losses than a level-based algorithm and therefore lead to greater accuracy. TabNet is a deep neural network designed to handle tabular data. TabNet inputs raw tabular data with no preprocessing and is trained using gradient descent-based optimization. It uses sequential attention to select features at each decision step, providing interpretability and better learning as the learning capability is used for the most useful features, with instance-specific feature selection. Neural Oblivious Decision Ensembles (NODE) is a deep learning architecture designed to handle tabular data. The NODE architecture generalizes ensembles of oblivious decision trees but benefits from both end-to-end gradient-based optimization and multilevel hierarchical learning capabilities. All of the above models can handle continuous variables (without categorical ones). For classification, we use only the DNA methylation levels of the different CpG sites, which are continuous variables. Parameter values of the trained models, which were found by hyperparametric search, can be found in Supplementary Table S1. All models have been trained for 2,000 epochs.

Each model was trained on 2 training datasets and then tested on the remaining independent datasets. Hyperparametric search was used to find optimal parameters for the models. There are a lot of quality metrics for the classification problem: accuracy, precision, recall, f1 score, Cohen’s kappa, Matthews correlation coefficient, AUROC (area under receiver operating characteristic curve), and so on. As the main metric, we choose accuracy with weighted averaging to take into account the possible imbalance of the classes. It is calculated according to the following formula:

(1) \begin{equation*} \frac{N_{cases}}{N} Accuracy_{cases} + \frac{N_{controls}}{N} Accuracy_{controls}, \end{equation*}

where *N*_*cases*_ is the total number of cases, *N*_*controls*_ is total the number of controls, and *N* is the total number of participants. The accuracy for each class is

(2) \begin{equation*} Accuracy = \frac{TP + TN}{N}, \end{equation*}

where *TP* is the number of true positives and *TN* is the number of true negatives. Adam optimizer and StepLR scheduler were used for the neural network models [128]. We used the following versions of software packages for the models: XGBoost 1.5.2 (XGBoost, RRID:SCR_021361), CatBoost 1.0.4 (Catboost, RRID:SCR_021694), LightGBM 3.3.2 (LightGBM, RRID:SCR_021697), TabNet 3.1.1, PyTorch 1.10.0 (PyTorch, RRID:SCR_018536), and PyTorch Lightning 1.6.0.

### Dimensionality reduction

Based on the features ranking, we performed dimensionality reduction of the models. We performed it, leaving only the most important CpGs for solving the classification problem. For this purpose, we built a series of models with different numbers of features. First, for each disease, we choose the top 10 most important features, and a new model is built for them (the type of model is chosen beforehand—it is the best in terms of accuracy for the full data). Then new models are built on the number of features from 10 to 1,000 in increments of 10, and for each such model, the weighted classification accuracy is calculated. For all these models, hyperparametric search was performed. According to the dependence of weighted classification accuracy on the number of features, we chose as optimal the number of features for which the highest weighted classification accuracy is observed for the considered diseases.

### Imputation of missing values

Missing data can be divided into 3 classes [129]: (i) missing completely at random (MCAR) values, if the probability of absence is completely independent of both observed and unobserved variables; (ii) missing at random (MAR) values, if the probability of absence is independent of the value itself but may depend on observed variables; and (iii) missing not at random (MNAR), if the probability of absence depends on the missing value itself. There is currently no statistical way to determine which category the specific missing data fall into. Assumptions are usually made based on knowledge of the data and the data collection and processing procedure. It is assumed that the missing values represent MCAR/MAR due to random experimental and technology-related errors [53]. It has been shown that missing values lying at the midrange methylation level are more difficult to impute than missing values close to the extremes of the range [54]. This is probably a consequence of the higher variance of methylation values in the middle ranges. Such a scenario could have a profound effect in terms of performance expectations, assuming that many missing values in the data are of the MNAR type and, in particular, lie in the middle range of β values.

In general terms, imputation approaches can be divided into single imputation (SI) and multiple imputation (MI) methods. SI methods replace a missing value with a single acceptable value. MI methods perform multiple SIs and average parameter estimates over multiple imputations to produce a single estimate. Under MCAR/MAR assumptions, the most common imputation methods like mean, median, or mode can handle missing data [130]. Such simple imputation methods are used often [131], but they can lead to systematic error or unrealistic results for multivariate datasets. In addition, for large data, this method often performs poorly [132]. The expectation maximization method is an iterative method for handling missing values in numerical datasets, and the algorithm uses an “impute, estimate, and iterate until convergence” approach. Each iteration involves 2 steps: expectation and maximization. Expectation estimates the missing values given the observed data, while maximization uses the current estimated values to maximize the probability of all data [133–135]. Besides classical methods, there are approaches to multiple imputation, for example, chained equation for big data [132]. Hot-deck imputation handles missing values by matching missing values with other values in the dataset for several other key variables that have complete values [136,137]. However, this method does not account for the variability of the missing data. One of the common hot-deck methods is KNN [138]. The KNN algorithm works by classifying the nearest neighbors of missing values and using those neighbors for imputation using a distance measure between instances [139]. Several distance measures can be used for KNN imputation, but Euclidean distance has been shown to provide efficiency and performance [140] and is therefore the most widely used distance measure. However, KNN imputation has weaknesses, such as poor accuracy when imputing variables and introducing false associations where none exists [141]. Another weakness of KNN imputation is that it scans the entire dataset, which increases computation time [142]. However, there are approaches developed in the literature to improve the KNN imputation algorithm [143–149]. All imputation methods that can deal with continuous variables are suitable for imputing DNA methylation data [53]. To study the effect of these methods on classification accuracy, we removed from consideration 100 CpG sites with the highest importance values for each disease and tried to fill them in. We used previously constructed small models for both diseases. Imputation methods were applied by impyute package version 0.0.8.

### Explainable artificial intelligence

Modern machine learning–based artificial intelligence systems are usually treated as black boxes. However, every decision must be made available for verification by a human expert [150]. One important aspect of model explainability is the ability to verify the system. For example, in health care, the use of models that can be interpreted and verified by medical experts is an absolute necessity [151]. Another aspect is to improve the system. The first step to improving the AI system is to understand its weaknesses. Performing weakness analysis on black box models is more difficult than on models that can be interpreted. Furthermore, model interpretability can be useful when comparing different models or architectures [152–154]. It can be argued that the better we understand what models do (and why they sometimes fail), the easier it becomes to improve them [150]. The next important aspect of explainability is the ability to learn from the system: since modern AI systems learn from millions of examples, they can observe patterns in the data that are inaccessible to humans, who can only learn from a limited number of examples [155,156]. Explainability is also important for other machine learning methods beyond neural networks [152].

One of the taxonomies to classify explanatory methods is global and local methods [64,65, 150]. Local interpretable methods apply to a single model result; they can explain the reason for a particular prediction or result. In contrast, global methods try to explain the behavior of the model as a whole. Perturbation is the easiest way to analyze the effect of changing input features on the AI model outputs. This can be accomplished by removing or changing certain input features, running a forward pass, and measuring the difference with the original output data. The input characteristics that most affect the output are evaluated as the most important ones. This is computationally costly, since a direct pass must be run after perturbing each group of input features. Such a perturbation-based approach is Shapley value sampling, which computes approximate Shapley values by taking each input feature for a certain number of times. It is a method from game theory that describes a fair distribution of wins and losses between input functions [157]. As a result, it is not a practical method in its original form but has led to the development of methods based on game theory, such as Deep SHAP (RRID:SCR_021362) [105]. SHAP has an alternative kernel-based approach to estimating Shapley values inspired by local surrogate models. There is also TreeSHAP, an efficient approach to estimating tree models, as well as DeepExplainer, an enhanced version of the DeepLIFT algorithm for deep neural networks. For the constructed portable models, we applied SHAP to obtain global and local explainability. SHAP values were calculated using eponymous package version 0.40.0.

## Code Availability Statement

The source code for the analysis workflow presented in the article is publicly available.

- Project name: DNAmClassMeta
- Project home page: https://github.com/GillianGrayson/DNAmClassMeta
- Operating system(s): Platform independent
- Programming language: Python
- Other requirements: Python 3.8 or higher, pytorch-lightning 1.5.10 or higher, xgboost 1.6.0 or higher, catboost 1.0.5 or higher, lightgbm 3.3.2 or higher, scikit-learn 1.0.2 or higher. All requirements are listed in the requirements.txt file in the project homepage.
- License: MIT
- biotools:dnamclassmeta
- RRID:SCR_022680

## Data Availability

No new data were generated. Data used in this study are available from the GEO database (accession numbers GSE145361, GSE111629, GSE72774, GSE84727, GSE80417, GSE152027, GSE116379). All supporting data and materials are available in the GigaScience GigaDB database [158].

### Additional Files

**Supplementary Table S1**. Parameters and their values that were varied to optimize the models.

**Supplementary Table S2**. Information about CpG sites making up small models for considered diseases. Intersection with other published works.

**Supplementary Table S3**. Gene Ontology (GO) enrichment for genes, connected with CpGs from the optimal small models.

## Abbreviations

AI: artificial intelligence; CatBoost: categorical boosting; DMP: differentially methylated position; DMR: differentially methylated region; DNAm: DNA methylation; EWAS: epigenome-wide association study; FDR: false discovery rate; GBDT: gradient-boosted decision tree; IPD: individual participant data; KNN: K nearest neighbor; LightGBM: light gradient boosting machine; MAR: missing at random; MCAR: missing completely at random; MI: multiple imputation; MNAR: missing not at random; NODE: Neural Oblivious Decision Ensemble; RCP: regression on correlated probes; SHAP: SHapley Additive exPlanations; SI: single imputation; XAI: explainable artificial intelligence; XGBoost: extreme gradient boosting.

## Competing Interests

The authors declare that they have no competing interests.

## Funding

The research was supported by the Ministry of Science and Higher Education of the Russian Federation, Grant for Major Research Projects in Priority Areas of Scientific and Technological Development No. 075-15-2020-808, grant recipient: Lobachevsky State University.

## Authors’ Contributions

Conceptualization: A.K., I.Y., M.G.B., M.I.; formal analysis: A.K., I.Y.; methodology: A.K., I.Y., M.G.B.; software: A.K., I.Y.; supervision: M.G.B., C.F., M.V., M.I.; visualization: A.K., I.Y.; writing—original draft: A.K., I.Y.; writing—review & editing: A.K., I.Y., M.G.B., C.F., M.V., M.I.

## Supplementary Material

giac097_GIGA-D-22-00134_Original_Submission

giac097_GIGA-D-22-00134_Revision_1

giac097_GIGA-D-22-00134_Revision_2

giac097_Response_to_Reviewer_Comments_Original_Submission

giac097_Response_to_Reviewer_Comments_Revision_1

giac097_Reviewer_1_Report_Original_Submission Liang Yu -- 6/25/2022 Reviewed

giac097_Reviewer_1_Report_Revision_1 Liang Yu -- 8/8/2022 Reviewed

giac097_Reviewer_2_Report_Original_Submission Giulia De Riso -- 6/29/2022 Reviewed

giac097_Reviewer_2_Report_Revision_1 Giulia De Riso -- 8/12/2022 Reviewed

giac097_Supplemental_Tables
