# Supplementary material for: Disease classification for whole-blood DNA methylation: Meta-analysis, missing values imputation, and XAI
Source: Gigascience. 2022 Oct 19;11:giac097. doi: 10.1093/gigascience/giac097 (PMC9718659; doi:10.1093/gigascience/giac097)
Supplement: giac097_GIGA-D-22-00134_Revision_1 [file giac097_giga-d-22-00134_revision_1.pdf]

GigaScience

Disease classification for whole blood DNA methylation: meta-analysis, missing values imputation, and XAI

--Manuscript Draft--

**Manuscript Number:**	GIGA-D-22-00134R1
**Full Title:**	Disease classification for whole blood DNA methylation: meta-analysis, missing values imputation, and XAI
**Article Type:**	Research
**Funding Information:**	Ministry of Science and Higher Education of the Russian Federation (075-15-2020-808) — Not applicable
**Abstract:**	Background: DNA methylation has a significant effect on gene expression and can be associated with various diseases. Meta-analysis of available DNA methylation datasets requires development of a specific workflow for joint data processing.<br>Results: We propose a comprehensive approach of combined DNA methylation datasets to classify controls and patients. The solution includes data harmonization, construction of machine learning classification models, dimensionality reduction of models, imputation of missing values, and explanation of model predictions by explainable artificial intelligence (XAI) algorithms. We show that harmonization can improve classification accuracy by up to 20% when preprocessing methods of the training and test datasets are different. The best accuracy results were obtained with tree ensembles, reaching above 95% for Parkinson's disease. Dimensionality reduction can substantially decrease the number of features, without detriment to the classification accuracy. The best imputation methods achieve almost the same classification accuracy for data with missing values as for the original data. XAI approaches have allowed us to explain model predictions from both populational and individual perspectives.<br>Conclusions: We propose a methodologically valid and comprehensive approach to the classification of healthy individuals and patients with various diseases based on whole blood DNA methylation data using Parkinson's disease and schizophrenia as examples. The proposed algorithm works better for the former pathology, characterized by a complex set of symptoms. It allows to solve data harmonization problems for meta-analysis of many different datasets, impute missing values, and build classification models of small dimensionality.
**Corresponding Author:**	Alena Kalyakulina<br>Lobachevsky State University of Nizhny Novgorod: Nacional'nyj issledovatel'skij Nizegorodskij gosudarstvennyj universitet imeni N I Lobacevskogo<br>Nizhny Novgorod, RUSSIAN FEDERATION
**Corresponding Author Secondary Information:**	
**Corresponding Author's Institution:**	Lobachevsky State University of Nizhny Novgorod: Nacional'nyj issledovatel'skij Nizegorodskij gosudarstvennyj universitet imeni N I Lobacevskogo
**Corresponding Author's Secondary Institution:**	
**First Author:**	Alena Kalyakulina
**First Author Secondary Information:**	
**Order of Authors:**	Alena Kalyakulina<br>Igor Yusipov<br>Maria Giulia Bacalini<br>Claudio Franceschi<br>Maria Vedunova<br>Mikhail Ivanchenko

Powered by Editorial Manager® and ProduXion Manager® from Aries Systems Corporation

**Order of Authors Secondary Information:**	
**Response to Reviewers:**	Dear Editor,<br>We were pleased to receive a thorough and helpful feedback from both reviewers with the generally positive evaluation of the manuscript. Having carefully addressed their comments and suggestions, we resubmit the revised manuscript. We hope that it warrants publication in the present form.<br>The point-to-point reply to the reviewers' comments follows.<br><br>On behalf of all authors,<br>Alena Kalyakulina<br><br><br>Reviewer 1<br>The paper by Kalyakulina et al. described the disease classification for whole blood DNA methylation. The author proposed a comprehensive approach of combined DNA methylation datasets to classify controls and patients. The solution includes data harmonization, construction of machine learning classification models, dimensionality reduction of models, imputation of missing values, and explanation of model predictions by explainable artificial intelligence algorithms. For Parkinson's disease and schizophrenia, the author also demonstrates that a method for classifying healthy individuals and patients with various disorders based on whole blood DNA methylation data is an efficient and comprehensive approach. Overall, the manuscript is well organized. I have some suggestions for the authors to improve their work:<br><br>Comment 1. The manuscript has constructed different models for the prediction study of CpG sites for different types of data. It is suggested to add a flowchart of the whole model construction process to the manuscript so that readers can understand the study more clearly.<br><br>Our reply:<br>We have added Figure 1 with the schematic representation of all main steps of the proposed workflow.<br><br>Comment 2. In Figure 4, the author only shows the top 10 important features and marks the highest accuracy and number of features with black lines in the figure. It is recommended to show the relevant data (optimal accuracy and number of features) in the figure. For the three subplots included in the figure, please label them separately, e.g., A, B, and C to indicate them separately.)<br><br>Our reply:<br>We followed the suggestion and added letter labels to the subplots. In Figure 4, the parameters of the optimal small models (accuracy and number of features) are introduced in subplots B and E.<br><br>Comment 3. Remark concerns model performance evaluation: author should provide standard deviations of the obtained values.<br><br>Our reply:<br>In this paper, we consider a common case (dictated by the practice), when the model is trained on certain data sets and tested on completely different data sets, which are new and unfamiliar to it. In this setup cross-validation is not applicable, and therefore it is not possible to calculate the standard deviation on different folds. It is worth mentioning that for the situation we are considering, a common problem is the absence of some features in the test data and the need to impute them. The built model should always accept certain attributes, on which the training has been performed, even if they are missing in the test data for some reason (this problem is covered in the subsections "Imputation of missing values" of Results and Methods sections). In particular, the best-known epigenetic clock models of Horvath or Hannum are built on a strictly defined number of CpG sites (353 and 71 CpG sites, respectively). In independent test datasets, some of these CpG sites may be missing due to, for example, failed quality control checks. The epigenetic clock of Horvath and Hannum imputes missing values either by simple methods (mean) or KNN with 10 neighbors. The simplest methods within our work do not show better results, nor does increasing the number of neighbors in KNN lead to better results for all the test datasets we

consider.
It could be possible to combine all datasets together and apply cross-validation, but in this case one has to ensure that the most important features in the resulting model are not caused by the batch effects of certain datasets (which can be most evident in the case of non-harmonized data). Such features can be dataset-specific and not biologically relevant. They can have a strong impact on the quality of the model when validating it on completely new data. This task may be the subject of further research beyond the context of this study.

Comment 4. In this manuscript, the author used graphs to present the results and suggested that a table summarizing the performance results of the model would be intuitive.

Our reply:
Figures with the results of classification and imputation have been replaced by tables.

Comment 5. I didn't find how the authors optimize the hyper-parameters, usually using grid search.

Our reply:
To find the optimal combination of model parameters that provides the best accuracy, we made use of hyperparametric grid search (the corresponding values are presented in Supplementary Table 1). Previously, this was only mentioned in the Classification Models subsection of the Methods section. We also added information about hyperparametric search in the Classification Models subsection of Results. Supplementary tables have been renumbered according to this change.

Comment 6. The authors do not adequately address how their method outperforms existing methods in the discussion section.

Our reply:
The task of classifying cases and controls for Parkinson's disease and schizophrenia based on whole blood DNA methylation data is underrepresented in the literature. For Parkinson's disease, the task of classifying different neurodegenerative diseases using DNA methylation-derived profile scores was solved (Nabais et.al., 2021). For Parkinson's disease in this study, the best result was an AUROC value of 0.7 for binary classification of two different datasets with this disease as well as for binary classification of Parkinson's disease and rheumatoid arthritis. In the same study for schizophrenia, the best result was an AUROC value of 0.78 for the binary classification of schizophrenia and rheumatoid arthritis. For schizophrenia, the task of classifying cases and controls was considered, but only for CoRSIV probes with polygenic risk score (Gunasekara et. al., 2021). The paper does not provide accuracy values, but only positive predictive values for cases (not controls) with the best result of 86%. It is worth noting that this work uses the dataset GSE84727 for model training and GSE80417 for the testing. These 2 datasets use the same data preprocessing (as we mentioned in Table 1), which can affect the result. Both works did not use data harmonization, did not consider the problem of missing value imputation, and did not explain the model outputs.
Thus, in this formulation, the task of classifying cases and controls from whole blood DNA methylation data with harmonization, imputation of missing values, and application of XAI approaches is solved for the first time. We added references to the works mentioned above in Discussion.

Comment 7. The "Dimensionality reduction" section: I think this section is more appropriately called "feature selection", a sequence forward search method. First sort the features according to their importance values, then add or remove features from a candidate subset while evaluating the criterion.

Our reply:
We appreciate the suggestion, but would refrain from renaming "Dimensionality reduction" to "Feature selection" due to possible confusion. The initial feature selection is done in the "Meta-analysis and harmonization" subsection (workflow step). For each disease and for all CpG sites in the training datasets, we perform the Mann-Whitney U-test for the control group only. CpG sites with an adjusted p-value >0.05 have similar

Powered by Editorial Manager® and ProduXion Manager® from Aries Systems Corporation

distributions of methylation levels among controls in different datasets. These CpG sites are used later for machine learning models. We do not use cases because their methylation levels may have high variability due to disease heterogeneity.
In the "Dimensionality reduction" subsection, we build multiple models for the best method with a varying number of features (from 10 to 1000 in increments of 10) based on the importance rating. The resulting best model has a much smaller number of features as compared to the original one, without much loss in accuracy. This can indeed be called a second feature selection, but we believe that it does not need to be renamed so as not to be confused with the feature selection based on the equality of methylation level distributions in the control group. We first perform feature selection to build the model and then reduce the dimensionality of the best model already built.

Reviewer 2
In this study, a workflow is presented to generate classification models from DNA methylation data. Methods to deal with harmonization and missing data imputation are presented and the benefit of adopting them for classification tasks is tested on case-control datasets of schizophrenia and Parkinson disease. The authors support this workflow with source code.
Although mostly based on already known methodologies, the present study may help orient studies aimed at building and applying DNA methylation based models. However, some major concerns can be raised:

Majors:
Comment 1. In different points of the manuscript, the authors refer to their approach as a pipeline. Indeed, this approach should be composed of sequential modules, in which the output of a module becomes the input of the next one. Although the modules are clearly distinguishable, their organization in the pipeline is less straightforward (also considering that modules can be adopted both to build a model and to use it on new data). The authors could think to draw a scheme of the pipeline, or to adopt a different term to refer to the presented approach.

Our reply:
We renamed "pipeline" to "workflow" because, in our opinion, this term better reflects the essence of the proposed approach - a sequence of modules executed in a certain order, where the output of one module is the input of the next one. We have also added Figure 1 with an illustration of the proposed workflow.

Comment 2. From the model performance perspective, the ML models poorly perform for schizophrenia. The authors point to inner characteristics of the disease as a possible reason for this. However, this point should be better commented in the Discussion section. Besides this, the impact of the smaller number of samples included in the training set and the higher proportion of imputed features compared to Parkinson disease on the classification accuracy should be discussed. In addition, since the authors provided the code, is there a way to select samples to include in training/test sets based on random choice (classical 70-30% splitting) instead of source dataset?

Our reply:
The requested comments supported by the related literature have been added to Discussion. In particular, it is pointed out that schizophrenia is a complex disease characterized by many different symptoms, with a variety of causes and molecular patterns. The individual molecular landscape can vary considerably among patients with schizophrenia. Fewer samples in the training sample for schizophrenia may also affect the final result. Insufficient data may not fully describe the disease characteristics for the model.
The experiment with imputation of missing values in our case was synthetic. For both Parkinson's disease and schizophrenia, the test data sets did not contain missing values in those CpG sites that participated in the construction of the best small models. For each disease, we removed 100 CpG sites, which were top-ranked by importance value. These 100 CpG sites were then imputed using various methods, and their results were compared with the original model. Despite the fact that in our case there were no missing data, in real life such a factor as the number of missing values (and, accordingly, the quality of their filling) can have a significant impact on the final result. Classic cross-validation can be applied in this case. This may have an impact on the

Powered by Editorial Manager® and ProduXion Manager® from Aries Systems Corporation

result, since the training set will include samples from different datasets. Here, it is critical to make sure that the most important features in the resulting model are not due to batch effects or different preprocessing of specific datasets (which is most obvious in the case of non-harmonized data). Such attributes can affect the quality of the model when tested on entirely new data. However, the focus of our work is not the case where we have all the data on hand at once (and no new data are expected), but a closer to reality case in which the model is trained on some data and tested later on others (which may have a different preprocessing, bias, or batch effect). It is important that the model is not re-trained. In this formulation of the problem, we do not use cross-validation, so as not to mix training and test sets. Therefore, the harmonization step is particularly important.

Comment 3. "For machine learning models, we used only those CpG sites that have the same distribution of methylation levels in different datasets in the control group (methylation levels in the case group typically have greater variability because of disease heterogeneity).": is this filtering performed only on the datasets included in the training set, or also on the test set? It seems the former, but the authors should clearly state this point.

Our reply:
We selected those CpG sites that have the same methylation level distributions in different train datasets in the control group only. An appropriate correction has been added to the "Meta-analysis and harmonization" subsection of the Results section and to the "Data harmonization" subsection of the Methods section.

Comment 4. Accuracy with weighted averaging should be defined with a formula in the methods section

Our reply:
We added a formula for accuracy with weighted averaging to the "Classification models" subsection of the Methods section.

Comment 5. Regarding the ML models, the authors chose different types of decision-trees ensemble, along with a deep learning one. They should contextualize this choice (why different models from the same family?). In addition, ML models built on DNA methylation are often based on elastic net or Support-Vector Machines, which are not accounted for in this work. The authors should comment on this aspect in limitations, and state whether the code they provided for their approach could be customized to adopt different models from the ones they presented.

Our reply:
Gradient-boosting decision trees (GBDT) have recently become state-of-the-art models for solving classification and regression problems on tabular data. GBDTs train multiple decision trees to predict the outcome. Although the three considered models, XGBoost, CatBoost, and LightGBM, belong to the same group of methods, they all have structural differences. XGBoost iteratively trains an ensemble of shallow decision trees, with each iteration using error residuals from the previous model to fit the next model. The final prediction is a weighted sum of all tree predictions. CatBoost builds symmetric trees. At each step, the leaves of the previous tree are separated by the same condition. A feature-split pair is selected and used for all nodes, which provides the least losses. LightGBM splits the tree by leaf with the simplest fit, whereas other boosting algorithms split the tree by depth or by level rather than by leaf. The Neural Oblivious Decision Ensembles (NODE) architecture generalizes ensembles of oblivious decision trees, but benefits from both end-to-end gradient-based optimization and multilevel hierarchical learning capabilities. Information about the differences between the considered models is presented in the "Classification models" subsection of the Methods section. In our experiments, these models demonstrate different performance. For example, for non-harmonized Parkinson's disease data, the LightGBM model shows the best performance, exceeding the accuracy of the CatBoost model by 5%. At the same time, for harmonized schizophrenia data, the CatBoost model's result exceeds the NODE model's result by 6%.
The Elastic Net model is indeed often applied to DNA methylation data, especially for the epigenetic clock. However, this approach in the classical sense is used to solve the regression problem, while logistic regression with the elastic net penalty is used for

classification.
We added the Logistic Regression and Support-Vector Machine (SVM) models to consideration. We applied both methods to both diseases, Parkinson's disease and schizophrenia, to non-harmonized and harmonized data. We found that these classical approaches do not outperform the GBDT models. These methods have also been added to the code repository.

Comment 6. Regarding the Imputation Method column in Table 2, the meaning is not clear. Are the different imputation methods described in the Imputation of missing values section paired with the ML models presented in Table 2? If yes, some of the methods (like KNN) are missing.

Our reply:
Table 2 provides information about the missing value imputation methods integrated into the considered models and provided by their API. Since not all models perform imputation themselves, we consider it as a separate necessary step in the described workflow. To avoid misunderstanding, we removed the Imputation method column from Table 2.

Comment 7. In the harmonization section, Models for case-control classification are trained on different numbers and sets of CpGs. To assess the effect of harmonization alone, the number of CpGs should be instead fixed. This is especially critical for schizophrenia, when the number of features for the non-harmonized data is 35145 whereas the one for harmonized data is 110,137.

Our reply:
The first step in the described workflow is data harmonization. The regRCPqn approach is used (regional regression on correlated probes with quantile normalization). One of the goals is to compare classification results using data without and with harmonization. However, not all CpG sites are used to build classification models.
Let us first consider Parkinson's disease. The two training datasets are GSE145361 and GSE111629. Only the control group is considered. For all CpG sites in these datasets, the Mann-Whitney U-test is performed for the control group to compare the distributions of methylation levels. We select only those CpG sites for which the adjusted p-value is >0.05. If the data are not harmonized, there will be 43019 such CpG sites. In the case of harmonized data, the condition is satisfied for 50911 CpG sites. Classification models are constructed for these selected CpG sites. Similarly for schizophrenia. The two training datasets are GSE84727 and GSE80417. In the control group, the Mann-Whitney U-test is performed for these datasets for all CpG sites. For non-harmonized data, the condition for the adjusted p-value is satisfied for 35145 CpG sites, and for harmonized data, the condition for 110137 CpG sites is satisfied. Thus, we consider all CpG sites that passed the test for p-value for each case.
The intersection of the resulting CpG sites for non-harmonized and harmonized data contains 13370 CpG sites for Parkinson's disease and 4539 CpG sites for schizophrenia. The overlap is not too great. This may be due to the fact that for non-harmonized data, CpG sites may have a similar distribution of methylation levels for a variety of reasons related to batch-related variability, chip position in batches, different preprocessing, and other non-biological reasons. Whereas, the same methylation level distributions for the harmonized data are devoid of dependence on the specific dataset and largely reflect biologically relevant causes.
We performed classification experiments for the best models: LightGBM for Parkinson's disease and CatBoost for schizophrenia. CpG sites from the intersection of lists for non-harmonized and harmonized data were used as features. Thus, the model for Parkinson's disease was trained on 13370 features, and the model for schizophrenia was trained on 4539 features. For GSE72774 (Parkinson’s disease) LightGBM has reached accuracy of 0.75 for the non-harmonized data and 0.94 for harmonized data. For GSE152027 (Schizophrenia) CatBoost has reached accuracy of 0.67 for non-harmonized data and 0.71 for harmonized data. For GSE116379 (Schizophrenia) CatBoost has reached accuracy of 0.58 for non-harmonized data and 0.67 for harmonized data. The results retain the trend found for the complete data. Harmonization works well for Parkinson's disease; for schizophrenia, improved accuracy is observed for the test dataset with different preprocessing. However, the overall accuracy values were lower than those described in the paper.

Powered by Editorial Manager® and ProduXion Manager® from Aries Systems Corporation

Our results for non-harmonized and harmonized data show that harmonization either leaves the result the same or improves it. Therefore, in practice, when working with multiple test datasets within our workflow, it is preferable to perform harmonization. In this case, adding information related to non-harmonized data does not bring substantial benefit.

Comment 8. Dimensionality reduction section: are the models from imputed and not-imputed data trained only on harmonized data? And how the set of 50911CpG sites for Parkinson and 110137 CpG sites for schizophrenia is selected?

Our reply:
For the best models in terms of accuracy, all the following steps (dimensionality reduction, imputation of missing values, XAI) are performed using only harmonized data. Models for Parkinson's are trained on 50911 harmonized CpGs; models for schizophrenia are trained on 110137 harmonized CpGs. They are chosen based on distributions of methylation levels in controls. For the harmonized data, the Mann-Whitney U-test is performed, and CpG sites are selected for which the p-value >0.05. Such CpG sites have the same distribution of methylation levels in the training data sets from controls.
This approach avoids the situation in which the machine learning model selects CpG sites specific to a particular dataset as the most important features. These CpG sites will be useless for other datasets, where the distribution of methylation levels for them may be different.
This approach minimizes the risk of a situation where the model implicitly tries to solve separate binary classification problems in each individual dataset and finally solves a new multiclass classification problem whose classes correlate with specific datasets (instead of the "case vs control" problem, the "case_dataset_1 vs control_dataset_1 vs case_dataset_2 vs control_dataset_2 vs ..." problem would be solved implicitly).

Comment 9. Imputation of missing values section: it is not clear on which CpGs and on which samples imputation is performed. Also, it is not clear whether the imputation has been tested on the best-performing model.

Our reply:
The small optimal models built in the "Dimensionality reduction" subsection (for harmonized data) are used for the imputation stage. The LightGBM model with 890 CpG sites is used for Parkinson's disease, and the CatBoost model with 670 CpG sites is used for schizophrenia.
For each model, 100 CpG sites with the highest values are excluded. The number of CpG sites was chosen to cause a significant drop in accuracy and to emphasize the differences in efficiency between the imputation methods. There are no missing features in the resulting small optimal models, so real CpG sites are excluded from consideration.
The different imputation methods are tested on the same datasets as before: GSE72774 for Parkinson's disease, GSE152027, and GSE116379 for schizophrenia. We examine how the accuracy of the "cases vs controls" classification changes when different imputation methods are used relative to the baseline model on data without missing features.
The text of the subsection "Imputation of missing values" is rephrased accordingly.

Minors:
Comment 1. Page 1, line 2: "DNA methylation is associated with epigenetic modification". DNA methylation is an epigenetic mark itself. Do the authors mean histone marks?

Our reply:
Indeed, DNA methylation is an epigenetic modification. This wording is a typo, and it has been corrected.

Comment 2. Page 1, from line 7: "DNA methylation consists of binding a methyl group to cytosine in the cytosine-guanine dinucleotides (CpG sites). Hypermethylation of CpG sites near the gene promoter is known to repress transcription, while hypermethylation in the gene body appears to have an opposite, also less pronounced effect.": references should be added

Powered by Editorial Manager® and ProduXion Manager® from Aries Systems Corporation

	Our reply:<br>We followed the suggestion and added references that mention this statement: (Jones, 2012) and (Jjino et. al., 2012).<br><br>Comment 3. Page 2, from line 2 : "Current epigenome-wide association studies (EWAS) test DNAm associations with human phenotypes, health conditions and diseases.": references should be added<br><br>Our reply:<br>We followed the suggestion and added references supporting this statement: (Rakyan et. al., 2011), (Liu et. al., 2019) and (Birney et. al., 2016).<br><br>Comment 4. Page 3: "In most cases, an increase in dimensionality does not provide significant benefits, since lower dimensionality data may contain more relevant information".<br>This point could be presented in a reverse way (higher dimensionality data may contain redundant information), introducing the collinearity issue.<br>In addition, this issue could be introduced before the missing values and imputation section.<br><br>Our reply:<br>We have rephrased the statement about the relationship between the dimensionality of the data and the information it contains.<br><br>Comment 5. Page 3: references for "Modern machine-learning-based artificial intelligence systems are powerful and promising tools" could be more specific for the field of epigenetics and DNA methylation.<br><br>Our reply:<br>We have rephrased the statement and added references to works related to the analysis of biomedical data and, in particular, DNA methylation. In this paragraph, we wanted to point out the problem of explainability from a global point of view, as this is a separate problem for any models, not only for DNA methylation analysis.
**Additional Information:**	
**Question**	**Response**
Are you submitting this manuscript to a special series or article collection?	No
**Experimental design and statistics**<br><br>Full details of the experimental design and statistical methods used should be given in the Methods section, as detailed in our Minimum Standards Reporting Checklist. Information essential to interpreting the data presented should be made available in the figure legends.<br><br>Have you included all the information requested in your manuscript?	Yes
**Resources**	Yes

A description of all resources used, including antibodies, cell lines, animals and software tools, with enough information to allow them to be uniquely identified, should be included in the Methods section. Authors are strongly encouraged to cite Research Resource Identifiers (RRIDs) for antibodies, model organisms and tools, where possible.<br><br>Have you included the information requested as detailed in our Minimum Standards Reporting Checklist?	
**Availability of data and materials**<br><br>All datasets and code on which the conclusions of the paper rely must be either included in your submission or deposited in publicly available repositories (where available and ethically appropriate), referencing such data using a unique identifier in the references and in the "Availability of Data and Materials" section of your manuscript.<br><br>Have you have met the above requirement as detailed in our Minimum Standards Reporting Checklist?	Yes

LaTeX main file (Overleaf GigaScience template)

Click here to access/download;LaTeX - Main Document (TeX file);main.tex

OXFORD (GIGA)^n SCIENCE

GigaScience, 2022, 1–20

doi: xx.xxxx/xxxx
Manuscript in Preparation
Research

RESEARCH

Disease classification for whole blood DNA methylation: meta-analysis, missing values imputation, and XAI

Alena Kalyakulina[1,*,†], Igor Yusipov[1,†], Maria Giulia Bacalini[2], Claudio Franceschi[1], Maria Vedunova[3] and Mikhail Ivanchenko[1]

[1]Institute of Information Technologies, Mathematics and Mechanics, Lobachevsky State University, Nizhny Novgorod, Russia and [2]IRCCS Istituto delle Scienze Neurologiche di Bologna, Bologna, Italy and [3]Institute of Biology and Biomedicine, Lobachevsky State University, Nizhny Novgorod, Russia

[*]kalyakulina.alena@gmail.com
[†]Contributed equally

Abstract

Background: DNA methylation has a significant effect on gene expression and can be associated with various diseases. Meta-analysis of available DNA methylation datasets requires development of a specific workflow for joint data processing.
Results: We propose a comprehensive approach of combined DNA methylation datasets to classify controls and patients. The solution includes data harmonization, construction of machine learning classification models, dimensionality reduction of models, imputation of missing values, and explanation of model predictions by explainable artificial intelligence (XAI) algorithms. We show that harmonization can improve classification accuracy by up to 20% when preprocessing methods of the training and test datasets are different. The best accuracy results were obtained with tree ensembles, reaching above 95% for Parkinson's disease. Dimensionality reduction can substantially decrease the number of features, without detriment to the classification accuracy. The best imputation methods achieve almost the same classification accuracy for data with missing values as for the original data. XAI approaches have allowed us to explain model predictions from both populational and individual perspectives.
Conclusions: We propose a methodologically valid and comprehensive approach to the classification of healthy individuals and patients with various diseases based on whole blood DNA methylation data using Parkinson's disease and schizophrenia as examples. The proposed algorithm works better for the former pathology, characterized by a complex set of symptoms. It allows to solve data harmonization problems for meta-analysis of many different datasets, impute missing values, and build classification models of small dimensionality.

Key words: DNA methylation, machine learning, data harmonization, explainable artificial intelligence

Introduction

Background

DNA methylation (DNAm) plays an important role in human development and is associated with gene expression, genomic imprinting, and other biological processes without altering the DNA sequence [1, 2, 3, 4, 5, 6, 7, 8]. Abnormal methylation patterns can lead to numerous diseases [9]. DNA methylation consists of binding a methyl group to cytosine in the cytosine-guanine dinucleotides (CpG sites). Hypermethyla-

Compiled on: August 1, 2022.
Draft manuscript prepared by the author.

tion of CpG sites near the gene promoter is known to repress transcription, while hypermethylation in the gene body appears to have an opposite, also less pronounced effect [10, 11]. Changes in DNAm patterns are associated with aging and environmental exposures [12, 13]. Current epigenome-wide association studies (EWAS) test DNAm associations with human phenotypes, health conditions and diseases [14, 15, 16]. Microarray-based technologies, such as the Illumina HumanMethylation450 (450K) and HumanMethylationEPIC (850K) arrays [17] are based on the hybridization of bisulfite-converted DNA to 50-mer probes and for each CpG site included in the design allow to estimate the fraction of methylated DNA copies. Two metrics are used to represent methylation levels: the β-value, ranging from 0 to 1, and the M-value, the log2 ratio of the intensities of methylated versus unmethylated probes [18, 19, 20]. M-values are more robust quantifiers since β-values close to 0 and 1 suffer from substantial heteroscedasticity [18].

Nowadays, machine learning has become a broadly applicable method for data modeling and analysis in a wide range of applications. The availability of large data sets and a variety of unreinforced generative methods make these approaches more accurate, simple, and relevant in bioinformatics, in particular, for transcriptomic and epigenetic data analysis [21, 22, 23, 24, 25, 26]. DNA methylation data are often used for classification tasks. One of the most common examples is the classification of different types of cancer using the TCGA repository [27]. Such classifiers usually demonstrate high accuracy [26, 28, 29, 30, 31, 32, 33, 34], based on both cancer-induced changes in methylation and the differences in methylation of various tumor tissues [28, 35]. Classifying different human conditions - phenotypes or pathologies - using DNA methylation data from a single tissue is more difficult. Phenotype classification can question smoking or obesity status, although existing results suggest that such conditions may not be clearly reflected in DNA methylation [26, 36, 37]. Classification of cases and controls for certain diseases is also performed using DNA methylation data. Examples of machine learning applications using epigenetic data include classification of coronary heart disease, neurodevelopmental syndromes, schizophrenia, Alzheimer's disease, psychiatric disorders and others [38, 39, 40, 41, 42, 43, 44].

One of the main challenges is that methylation datasets are limited in the number of samples. Increasing the amount of data requires combining many datasets collected under different conditions and then performing analysis for the merged data, which can cause a variety of problems. There are many factors that lead to significant differences in methylation data that are not directly related to the development of pathological conditions, to name the effect of the laboratory batch, different experimental conditions, normalization, and other [45].

Methylation levels can be affected by systematic variation due to biosample processing, i.e., batch-related variability (a subset of samples processed simultaneously), chip position in batches, and sample position within the chip [46, 47]. Batch effects can dramatically reduce the accuracy of measurements and produce false positive effects if the sample distribution is not uniform [48]. Most of the existing works avoid the question of the applicability of obtained models to new data. A central issue of meta-analysis is data harmonization. Ref. [49] developed an approach to systematically assess the impact of different preprocessing methods on meta-analysis. Its main advantage is the possibility of harmonization of the newly introduced datasets that does not require corrections to the previously analyzed datasets, employed for training the machine learning model.

Making use of new datasets to validate the model raises another problem, that is missing values in the data and the need to fill them in. New (test) datasets can lack information about some relatively small number of CpG sites on which the model was built. Experimental methylation data often contain missing values due to failing quality control checks, which can affect subsequent analysis. Since such missing CpG sites are necessary input parameters for the model, their values must be imputed. Examples include epigenetic clocks, which estimate biological age from small sets of pre-selected age-correlated CpG sites [5, 50, 51, 52], sensitive to small deviations in methylation levels [53]. Consequently, accurate imputation of missing data is required to improve the quality of DNA methylation analysis [54].

Dimensionality presents yet another problem. High data dimensionality is often associated with various undesirable consequences: increased computational effort, retraining, and visualization difficulties [55]. High-dimensional data may contain redundant information and introduce noise, while low-dimensional data may be sufficient for comprehensive data characterization. Since methylation data is multivariate, continuous, with nonlinear dependencies, traditional approaches often encounter the problem of multiple hypothesis testing and multicollinearity [26]. In addition, the most common epigenetic models [5, 51, 56, 57] contain a small number of variables to simplify data processing, for better interpretation of the results and for the possibility of applying these models in real life. It is also worth noting that small DNA methylation panels are significantly less costly [58], which is an undeniable advantage for the possibility of widespread use.

Modern artificial intelligence systems based on machine learning are powerful and promising tools in a wide range of applications from computer vision, machine translation and speech recognition [59, 60, 61] to the analysis of biomedical data, in particular DNA methylation [26, 62, 63]. However, while these models provide impressive predictive accuracy, their nonlinear structure makes them poorly interpretable, i.e. it is hard to explain what information in the input data leads AI to particular outputs. The need for trustworthy solutions has recently attracted much attention to methods that would "open" black box models [64, 65, 66, 67, 68, 69, 70, 71, 72, 73, 74]. This includes developing methods to help better understand what the model has learned [75, 76] as well as methods to explain individual predictions [65, 66, 67, 77, 78].

In summary, individual DNA methylation datasets contain an insufficient number of samples to apply machine learning approaches, so there is a need to combine and harmonize different datasets. Problems that arise on the way include tackling batch effects in individual datasets, missing values for certain samples, and high data dimensionality. Here, we analyze several existing fragmented solutions to these problems, develop a generalized unifying approach integrated in a workflow, validate it and demonstrate its efficiency.

Study design and novelty

Our primary goal is to offer a methodologically complete workflow for building machine learning models, classifying cases and controls for various diseases from whole blood DNA methylation data on many datasets, ranging from data harmonization to explainable artificial intelligence models. DNA methylation data are taken from different human body tissues, but the most widespread is whole blood methylation, the least invasive analysis and, therefore, of broad diagnostic prospects. We restrict our analysis to this kind of data. Our workflow solves a problem of harmonization of methylation data from different datasets. They are collected in different laboratories, with different setups and experimental conditions. In general, the data are of different quality, and have been preprocessed differently.

Harmonization is used to eliminate the unavoidable bias between the data and to minimize the associated machine learning model errors. The proposed workflow uses harmonization with the selection of a reference dataset, in which case all other datasets are aligned with the reference one, so that when a new dataset is introduced, there is no need to renormalize the training data and hence rebuild the model. The workflow uses the generally recognized types of machine learning models for classification on methylation data in tabular representation, in particular gradient-boosted decision trees. A hyperparametric search for the optimal combination of the parameters of these models is performed to ensure the best classification accuracy. Next, the dimensionality of the feature space is reduced to build portable models. In such models, the number of features has the same order as the most popular epigenetic models, such as the Horvath clock (353 CpG sites) [5], Hannum clock (71 CpG sites) [51], DNAm PhenoAge (513 CpG sites) [56], DNAm GrimAge (1030 unique CpGs were used to predict plasma protein levels) [57]. Such portable models allow them to be used for early diagnosis of various diseases - analysis of small CpG panels is much cheaper than full-genome analyses. Reducing the dimensionality of the data can also help discard noisy features that do not carry relevant information for classifiers. Also, the proposed approach includes the possibility of imputing missing values (CpG sites), and different approaches are used for this purpose. This is especially important when testing the model on new data, where some CpG sites critical for the model may be missed (e.g., because of technical errors in data acquisition and processing or failing quality checks). For the best models in terms of accuracy, explainable artificial intelligence (XAI) methods are applied to explore both the global influence of individual CpG sites on model predictions and to get explanations of how the methylation level values of individual CpG sites for specific subjects shape their individual predictions. Lists of the most important CpG sites in terms of machine learning models are compared with lists of CpG sites (and their corresponding genes) from existing studies associated with the considered diseases. Biological pathways of diseases based on these lists are identified and investigated.

Results

Larger training sample sizes provide better quality of machine learning models. The currently available DNA methylation data sets do not exceed several thousand samples, and that could hardly change in the near future due to complexity and cost of study. Merging different data sets, therefore, appears a practical way to circumvent size limitations. However, it poses many challenges, such as the need to harmonize datasets collected under different conditions and pre-processed in different ways, the need to fill in missing values in a way that preserves patterns in the data, the reduction of excessively high dimensionality of input variables with a relatively small number of samples. These issues have been addressed separately; below we report an integrated solution that brings together the data processing and analysis steps and the resulting methodologically complete workflow for solving the classification problem based on merging several independent DNA methylation data. A schematic representation of the proposed workflow is shown in Figure 1.

Datasets and machine learning tasks

We studied whole blood DNA methylation datasets generated on subjects with Parkinson's disease or schizophrenia. We selected 3 datasets that contain samples from subjects with Parkinson's disease and healthy controls: GSE145361 [79], GSE111629 [80, 81, 82], GSE72774 [80, 81, 83] and 4 datasets that contain samples from subjects with schizophrenia and healthy controls: GSE152027 [84], GSE84727 [84, 85], GSE80417 [84, 85], GSE116379 (non-famine participants) [86]. Information about considered datasets is summarized in Table 1, in particular, the number of cases and controls, whether the dataset has been used as train or test, the original preprocessing type, the number of CpGs.

For each disease, we built machine learning models to classify cases vs. controls. Some of these datasets are used as train data for building the model, and the rest is used to test the model. For each disease we selected a reference dataset, against which harmonization was performed. As it can be seen from Table 1, the original preprocessing is the same for the majority of the considered datasets with schizophrenia patients, but it varies considerably among the different datasets for Parkinson's disease. To reduce the influence of the laboratory-specific data collection and processing conditions on classification results, harmonization is necessary.

Meta-analysis and harmonization

Combining different DNA methylation datasets can improve the statistical power to test hypotheses and identify epigenetic signatures by meta-analysis. However, such meta-analysis also poses serious problems related to data harmonization, which is often not considered [87]. This is especially true for DNA methylation, where data is often only available in the preprocessed rather than raw form, and where diverse preprocessing pipelines are used [49]. Developed in [49] approach regRCPqn (regional regression on correlated probes with quantile normalization) allows for meta-analysis even if the raw data are not available. Importantly, as emerging datasets are aligned, the already treated datasets do not require renormalization. Therefore, we apply this approach to harmonization with reference. The largest dataset for each disease is taken as the reference, and other datasets are harmonized relative to it. The schematic representation of the harmonization process is shown in Figure 2.

For machine learning models, we used only those CpG sites that have the same distribution of methylation levels in different train datasets in the control group (methylation levels in the case group typically have greater variability because of disease heterogeneity). We used the Mann-Whitney U-test [88] to compare DNA methylation values of healthy participants from the considered train datasets before and after harmonization. After harmonization, the number of CpG sites with the adjusted p-value >0.05 (not significantly different between healthy subjects from the considered train datasets) increased from 43019 to 50911 for Parkinson's disease and from 35145 to 110137 for schizophrenia. Figure 3 illustrates the change in the distributions of methylation level values before and after harmonization. In particular, CpG sites whose methylation level distributions differed significantly before harmonization (FDR-corrected p-values<0.05) manifest similar distributions after harmonization (FDR-corrected p-values>0.05).

Classification models

The most common type of data representation for machine learning is tabular, and DNA methylation data fulfills it. Typically, the rows refer to participants, the columns refer to CpG sites, and the cells of the table contain the methylation levels of each CpG site for each participant. There are many machine learning models designed to work with tabular data: Logistic Regression with elastic net penalty [89], Support-Vector Ma-

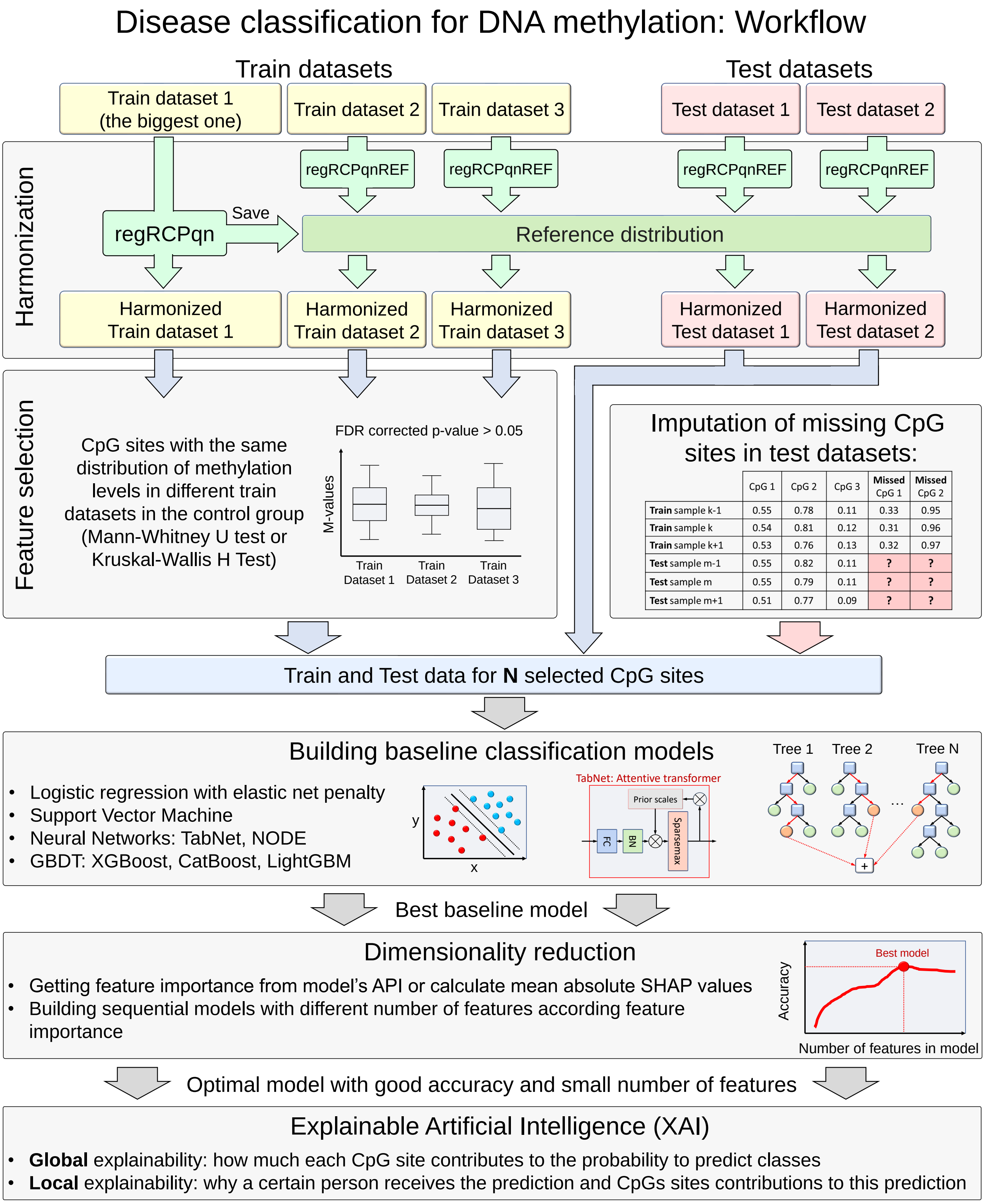

Figure 1. Workflow for classifying cases and controls of various diseases based on DNA methylation data proposed in this paper.

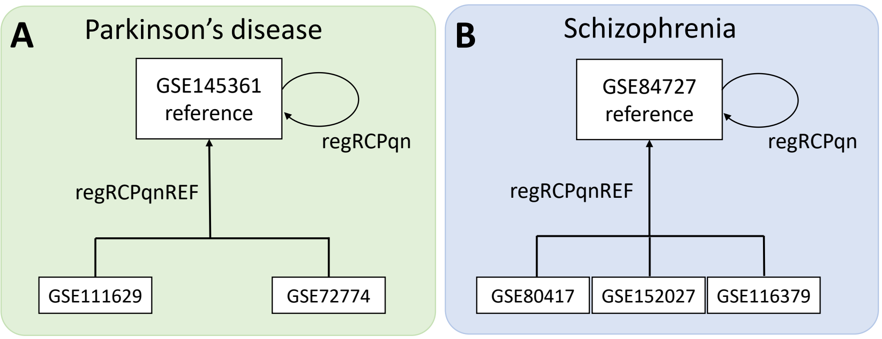

Figure 2. Schematic representation of harmonization procedure for (A) Parkinson's disease and (B) Schizophrenia datasets.

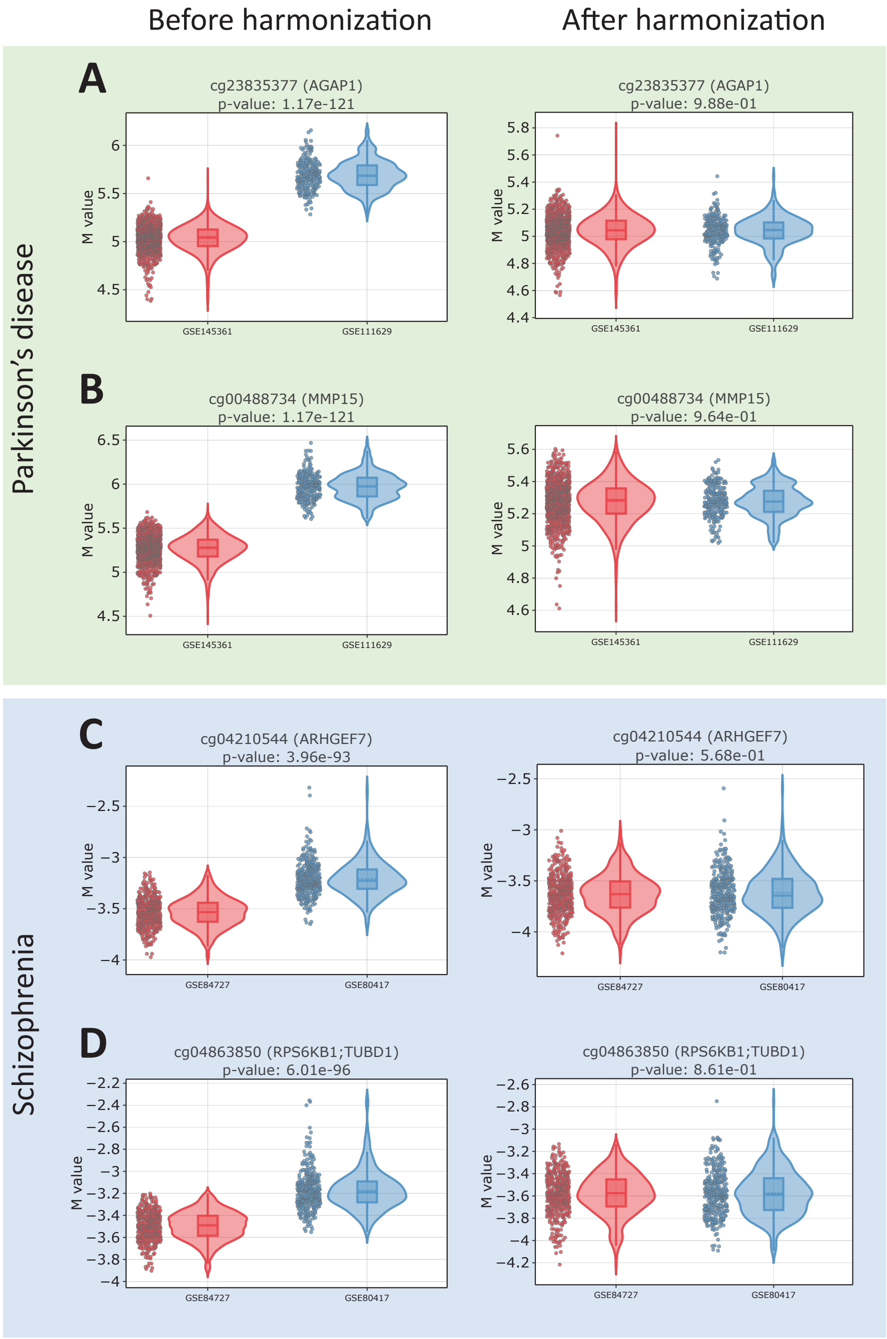

Figure 3. Examples of M-values methylation levels distribution for control groups before and after harmonization for Parkinson's disease examples (A) cg23835377 and (B) cg00488734, Schizophrenia examples (C) cg04210544 and (D) cg04863850.

Table 1. Main characteristics of considered datasets. For each disease, the bold row represents the reference dataset for the harmonization. Number of CpGs is common for train datasets in each disease. Three largest datasets for schizophrenia, GSE84727, GSE80417 and GSE152024, have the same preprocessing.

Disease	Dataset	Number of cases	Number of controls	Train or Test subset	Raw IDAT available?	Number of CpGs	Original preprocessing
Parkinson's disease	**GSE145361**	**959**	**930**	**Train**	**Yes**	**411761**	**Data processing: Genome Studio software**
	GSE111629	334	237	Train	Yes		Data processing: R software v3.4.2<br>Functional normalization: minfi R package
	GSE72774	289	219	Test	No	411979	Data processing: BeadStudio software v3.2
Schizophrenia	**GSE84727**	**414**	**433**	**Train**	**No**	**399625**	**Importing: methylumi R package methylumIDAT function<br>Preprocessing: watermelon R package pfilter and dasen functions**
	GSE80417	353	322	Train	No		Importing: methylumi R package methylumIDAT function<br>Preprocessing: watermelon R package pfilter and dasen functions
	GSE152027	290	203	Test	No	411901	Importing: methylumi R package methylumIDAT function<br>Preprocessing: watermelon R package pfilter and dasen functions
	GSE116379	51	54	Test	No	407781	Removed: X and Y chromosome, non-specific binding probes, failed probes based on a detection p-value > 0.001 and bead count < 5, probes with SNPs of Minor Allele Frequency > 5% within 10 base pairs of the primer<br>Functional normalization: minfi R package

Table 2. Main characteristics of the considered classification models.

Model	Type	Feature importance API
Logistic Regression	Generalized linear model	Yes
Support-Vector Machine	Supervised learning model constructing the separating manifold	Only for linear kernel
XGBoost	Gradient-boosted decision tree ensemble	Yes
CatBoost	Gradient-boosted decision tree ensemble	Yes
LightGBM	Gradient-boosted decision tree ensemble	Yes
TabNet	Deep neural network	Yes
NODE	Gradient-boosted decision tree ensemble	No

chine [90], XGBoost [91], CatBoost [92], LightGBM [93], TabNet [94], NODE [95]. Main characteristics of the models are summarized in Table 2.

For each disease, all considered datasets were divided into training and test ones (as stated in Table 1). We trained all models on two training datasets and then tested on the remaining datasets. Accuracy with weighted averaging was the main quality metric, as it can handle situations with possible imbalance of the classes (the number of participants in different classes varies significantly). As discussed in the above, the approach fulfills the requirement that the model does not have to be trained again as the new data set is considered. Moreover, the models must be trained to classify biological differences in methylation data rather than traces of different experimental conditions in different laboratories. Accordingly, we do not mix train and test datasets and do not perform cross-validation. To find the optimal combination of model parameters that provides the best accuracy, we used a hyperparametric grid search (the values are presented in Supplementary Table S1).

Newly introduced datasets may lack some CpG sites that are present in already trained models; in this case various imputation methods are applied (cf. Sections Imputation of missing values in Results and Methods for more details). Models for Parkinson's disease for non-harmonized data are trained on 43019 CpG sites, for harmonized data on 50911 CpG sites. Among these, the Parkinson's disease test dataset GSE72774 lacks 38 CpG sites in the non-harmonized data and 34 CpG sites in the harmonized data. Models for schizophrenia for non-harmonized data train on 35145 CpG sites, for harmonized data train on 110137 CpG sites. The first test dataset for schizophrenia GSE152027 lacks 9 CpG sites in the non-harmonized data and 36 CpG sites in the harmonized data. The second test dataset for schizophrenia GSE116379 lacks 268 CpG sites in the non-harmonized data and 609 CpG sites in the harmonized data. These missed CpG sites are imputed using KNN methods with K=1. However, this imputation does not have a significant effect on the result, because, as will be shown later, all the missed CpG sites are not at the top of the features in terms of importance.

Table 3 shows the results of cases vs. controls classifica-

Table 3. Binary classification results of baseline models for non-harmonized and harmonized data. For Parkinson's disease (green background) and schizophrenia (blue background), results comparing the accuracy of different models for non-harmonized and harmonized methylation data are shown.

Model	Parkinson's disease		Schizophrenia			
	GSE72774		GSE152027		GSE116379	
	Non-harmonized	Harmonized	Non-harmonized	Harmonized	Non-harmonized	Harmonized
Logistic Regression	0.71	0.93	0.63	0.66	0.56	0.66
Support-Vector Machine	0.67	0.92	0.62	0.66	0.58	0.65
XGBoost	0.72	0.95	0.67	0.71	0.56	0.66
CatBoost	0.71	0.94	0.68	0.72	0.59	0.71
LightGBM	0.76	0.97	0.68	0.71	0.58	0.67
TabNet	0.69	0.93	0.63	0.66	0.58	0.65
NODE	0.71	0.92	0.62	0.66	0.56	0.65

tion by baseline models based on non-harmonized and harmonized whole blood methylation data for Parkinson's disease and schizophrenia on test datasets. For each combination of harmonization type, disease, and test dataset, the best weighted accuracy values for all constructed models is given. All imputation methods described in Section Imputation of missing values do not significantly change the quality of the resulting models, because all missed CpGs in test datasets have a very low value of feature importance in models with corresponding API.

The results confirm that harmonization must be applied and is most efficient for the datasets with different preprocessing methods. In particular, for Parkinson's disease, all datasets have different original preprocessing, and the best model trained on such data shows a result of 76%. When these data are harmonized, accuracy improves dramatically to 97%. For both non-harmonized and harmonized data, for Parkinson's disease, the best model in terms of weighted accuracy is LightGBM. For schizophrenia, only one of 4 datasets has a different preprocessing (GSE116379, Table 1). Then, harmonization does not significantly affect the quality of the built models if the datasets have the same preprocessing (68% without harmonization, 72% with harmonization in the best models). The best model is LightGBM for non-harmonized data and CatBoost for harmonized data. However, applying models trained on non-harmonized data to data with a different preprocessing gives a poor result for binary classification - 59%. Harmonization of data improves the performance of the trained models, making them close to the best results obtained for schizophrenia in terms of quality - 71%. It is also worth noting that the overall classification quality for these two diseases on methylation data is very different, possibly due to the different etiology and molecular mechanisms involved in the two diseases.

Best accuracy models allow us to extract importance values for all features. The ranking of the most important features for these models for Parkinson's disease and schizophrenia is shown in Figure 4. It is worth noting that for schizophrenia, there is one outstanding CpG with the highest importance for classification, while the others have much lower values. For Parkinson's disease, the situation is more uniform. These rankings can be used for the dimensionality reduction of the built models.

Dimensionality reduction

As a result of applying different baseline models to methylation data to classify cases vs controls, the ones with an API for feature extraction showed the best accuracy. Based on the obtained ranking of the features (Figure 4), we performed dimensionality reduction of the constructed models. Most common epigenetic models comprise few CpG sites, no more than several hundred (for example, those used to predict epigenetic age like Horvath' clocks and Hannum clocks). First, models based on few features show significantly better performance while maintaining similar classification accuracy. Second, such models are less memory-consuming.

Along these lines, we reduced the dimensionality of the model, leaving only the most important features for classification. Figure 4 shows the dependence of weighted classification accuracy on the number of features in the model for Parkinson's disease and for schizophrenia. It first increases, until reaching a certain optimal number of features, and then changes weakly. For Parkinson's disease, the best weighted accuracy of 96% is observed for 890 CpG sites, with an accuracy value changed by only 1% compared to the full data (50911 CpG sites). For schizophrenia, the best weighted accuracy of 75% is observed for 670 CpG sites for the test dataset GSE152027 and 70% for the test dataset GSE116379. The optimal model for schizophrenia was chosen as the one for the GSE152027. The accuracy values changed by no more than 3% compared to the full data (110137 CpG sites). The list of CpG sites that make up these small models, as well as basic information about them (gene, chromosome, relation to the CpG island) is presented in Supplementary Table S2. The resulting CpG lists were compared with previously published lists of biomarkers associated with Parkinson's disease [80, 96, 97] and schizophrenia [84, 98]. Interestingly, for Parkinson's disease, there is practically no overlap with the previous results, except for one CpG site from [96]. This CpG belongs to the gene DYNC1H1, which is associated with neurological and neurodegenerative diseases [99, 100]. For schizophrenia, only 15 CpG sites are common with [84]. Some of the connected genes like PRKCZ, SHANK2, ZNF608, PRDM16 were also identified as schizophrenia risk factors [101, 102, 103, 104]. Genes, corresponding to CpG sites, from the optimal small model for Parkinson's disease were enriched in several gene ontologies related to neuronal and metabolic processes, whereas genes from schizophrenia models were enriched in gene ontologies related to cell development processes (Supplementary Table S3).

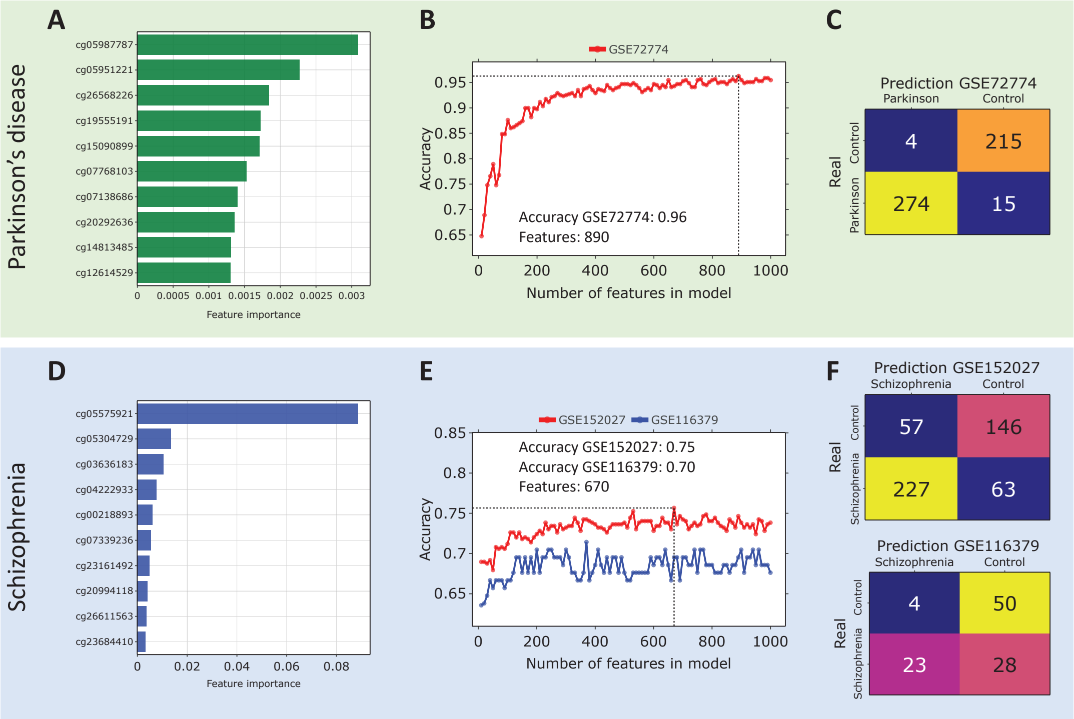

Figure 4. Dimensionality reduction for the best classification models. Parkinson's disease: (A) Top-10 features for the best classification model LightGBM with the normalized importance values. (B) Dependence of the weighted accuracy on the number of features in the model. Dotted line corresponds to the optimal small model with accuracy value 0.96 and 890 features. (C) Confusion matrix for optimal model. Schizophrenia: (D) Top-10 features for the best classification model CatBoost with the normalized importance values. (E) Dependence of the weighted accuracy on the number of features in the model. Dotted line corresponds to the optimal small model with accuracy value 0.75 for GSE152027, 0.70 for GSE116379 and 670 features. (F) Confusion matrix for optimal models.

Imputation of missing values

For trained machine learning models (both large and small), it is important that there are no missing values in the upcoming test data. Since it is impossible to guarantee their absence, various imputation methods are used to fill them in. Since not all models support data imputation, we use the most popular of them: mean, median, mode, random, chained equations, expectation maximization, KNN with different numbers of neighbors (from 1 to 3). To study the effect of these imputation methods on the classification accuracy, we consider the following simulation experiment. For each disease, we consider only the best small models, obtained at the previous step (LightGBM with 890 CpG sites for Parkinson's disease and CatBoost with 670 CpG sites for schizophrenia). For these models we "remove" 100 CpG sites with the highest importance values and impute them. The number of CpG sites was chosen to induce a significant drop in accuracy and to sharpen the differences in efficiency between the imputation methods. The missing CpG sites do not take part in the construction of small optimal models, so the actually existing CpG sites are removed from consideration. Table 4 shows results for the considered test datasets. For Parkinson's disease, KNN with one neighbor kept the classification accuracy at the baseline level of the data without missing values. Imputation with mode also showed good results, losing only 3%. The other methods achieved an accuracy of no more than 90%. For schizophrenia, none of the approaches achieved the baseline accuracy for data without missing values. This may be explained by the critical importance of specific features for classification. KNN with one neighbor for both datasets shows one of the best imputation results, for GSE152027 chained equation and expectation maximization perform better than KNN by 3% and 2%, respectively. Median and random values methods show unsatisfactory results in all experiments.

Explainable artificial intelligence

Even the most accurate machine learning models make mistakes on upcoming data. It presents a major challenge for those models that work as "black boxes" with unknown principles behind made decisions. SHapley Additive exPlanations (SHAP) help to understand why the model makes its predictions from the global and local points of view [105].

The global explainability of the constructed models on the training data for Parkinson's disease and schizophrenia is illustrated in Figure 5. Beeswarm plots show the relationship between SHAP values and methylation levels for the most important CpG sites. For each CpG site, the distributions of methylation levels for all participants are shown. In particular, for Parkinson's disease, most of the participants in the CpG site cg05987787 have low methylation levels, which positively affects the probability of predicting the disease. The scatter plots show in detail the distribution of methylation levels in different participants and SHAP values. We can see that M-values below 4 have a positive effect on the probability of predicting disease, while M-values above 4 have a negative effect. The black line divides the areas of positive and negative influence of SHAP values on the prediction of disease probability. The opposite situation is observed for the CpG site cg05951221. M-values below -1 have a negative effect on the probability of predicting disease, while M-values above -1 have a positive effect. Similar plots are shown for schizophrenia. The beeswarm plot shows that there is one most important CpG site, cg05575921, that contributes the most to the probability of predicting disease, as previously shown, and the other CpG sites have a much smaller effect.

The local explainability of predictions on the test data is

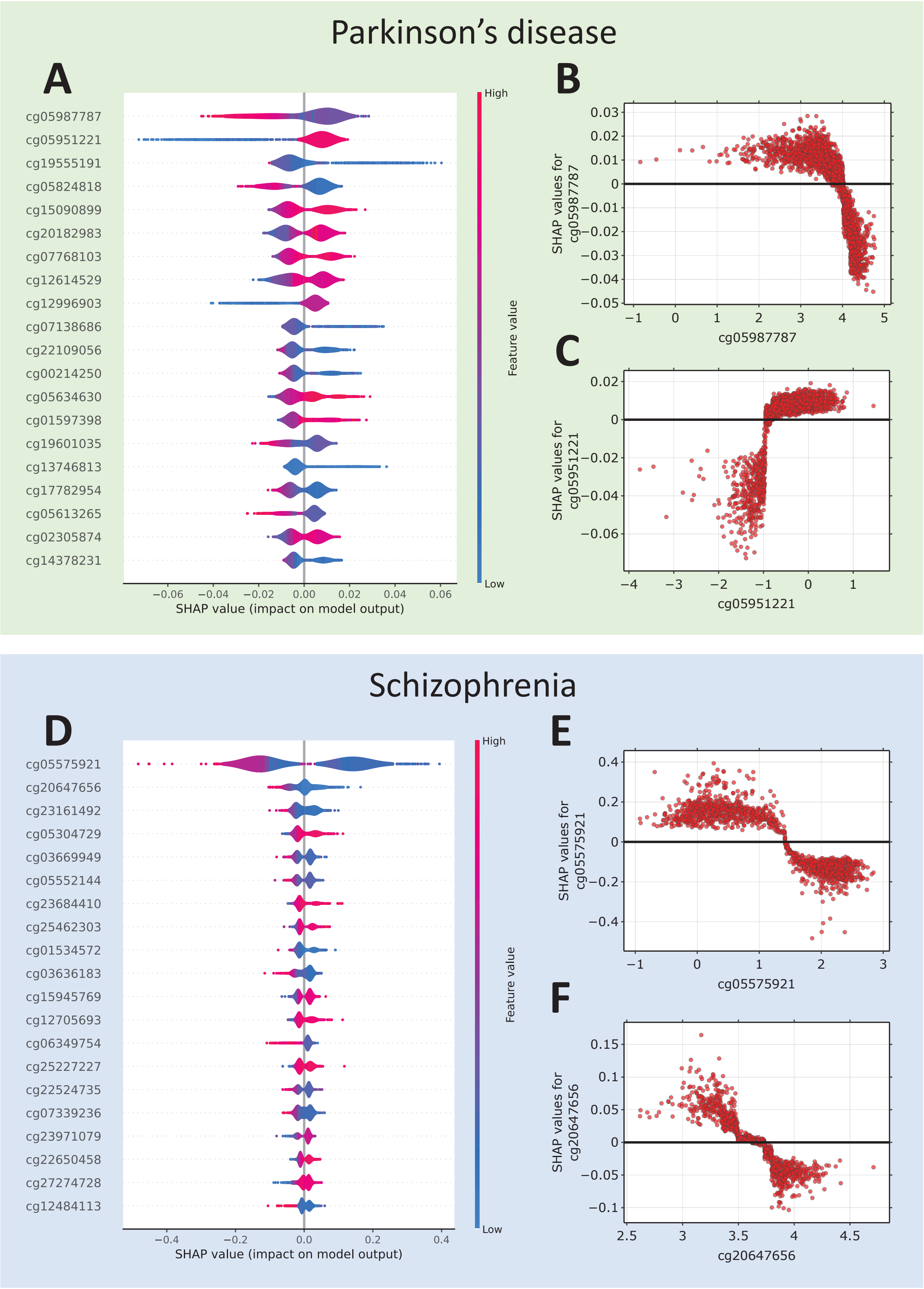

Figure 5. Global explainability based on SHAP values. Parkinson's disease: (A) Beeswarm plots show the dependence of SHAP values for each CpG site on their methylation levels. Each dot represents one participant. (B) Dependence of SHAP values on methylation M-values for cg05987787. The black line separates the areas of negative and positive influence of SHAP values on the probability of predicting Parkinson's disease. (C) Dependence of SHAP values on methylation M-values for cg05951221. Schizophrenia: (D) Beeswarm plots show the dependence of SHAP values for each CpG site on their methylation levels. Each dot represents one participant. (E) Dependence of SHAP values on methylation M-values for cg05575921. (F) Dependence of SHAP values on methylation M-values for cg20647656.

Table 4. Comparison of different missing value imputation methods and their effect on weighted classification accuracy for Parkinson's disease (green background) and schizophrenia (blue background). In all cases, 100 CpG sites with the highest importance values were dropped.

Model	Parkinson's disease	Schizophrenia	
	GSE72774	GSE152027	GSE116379
**Original (no missed data)**	**0.96**	**0.75**	**0.7**
Mean	0.78	0.65	0.67
Median	0.81	0.59	0.55
Mode	0.93	0.55	0.54
Random	0.86	0.62	0.54
Chained equation	0.89	0.68	0.61
Expectation maximization	0.87	0.67	0.54
KNN (K = 1)	0.96	0.65	0.67
KNN (K = 2)	0.9	0.65	0.67
KNN (K = 3)	0.86	0.65	0.67

shown in Figure 6. The top row presents heatmaps with participants on the x-axis, CpG sites on the y-axis, and SHAP values encoded on a color scale. The participants are ordered based on the probability to predict the disease. Model output is shown above the heatmap matrix. The black line represents the probability of predicting the disease for each participant. It follows that for Parkinson's disease, where the model works with high accuracy, this line is quite smooth and similar to the softmax function. Whereas for schizophrenia, for which the models have much lower accuracy, these probability plots are more fragmented. As shown earlier, for schizophrenia, one CpG site is the most important, so it has the highest absolute SHAP values and appears the brightest in the heatmaps. The center and bottom lines represent waterfall plots for participants with the disease and controls, respectively. They allow for explaining the model output for each participant separately. The bottom part of the waterfall plot shows the base probability of the model to predict disease, and then each line shows how a positive (red) or negative (blue) contribution from each CpG site moves the probability to the model output for that prediction. The output of the model is the probability of predicting disease. If the probability is greater than 50%, the model identifies the participant as a case, otherwise it identifies the participant as a control. The baseline probability is the average probability of the model predicting on the test data. Because baseline probability is a characteristic of the model, it depends on the quality of the model. If the model has reasonably good accuracy, then the base probability is close to the proportion of participants in a particular class. In the examples from middle line of the Figure 6 for all participants with diseases, the probability of predicting disease in the examples was above 97% (models identify them as cases almost for sure); for control participants (bottom line of Figure 6), the probability of predicting disease was below 5% (models identify them as controls almost for sure).

Discussion

Conclusion

We developed a multifunctional workflow for applying machine learning models to classify cases and controls for different diseases based on DNA methylation data. Specifically, we considered Parkinson's disease and schizophrenia as examples of complex diseases requiring early diagnosis. In addition, for these diseases there are large publicly available whole blood DNA methylation datasets. In this paper, the task of classifying cases and controls based on whole blood DNA methylation data with harmonization, missing value imputation, model dimensionality reduction, and application of XAI approaches is solved for the first time for Parkinson's disease and schizophrenia. In [106] the problem of pairwise classification of neurodegenerative diseases cases (in particular, for Parkinson's disease and schizophrenia) was solved. The authors did not use methylation levels; instead, they used methylation-derived profile scores as features; however, we do not compare diseases with each other in this work. In [40] the problem of classifying cases and controls for schizophrenia is solved. In that work, instead of methylation levels, other metrics were also used: CoRSIV probes with a polygenic risk score. The paper presents the results of positive predictive values only for cases, not for controls, so it is not possible to directly compare the results.

The first step of the workflow is to harmonize the data according to the chosen reference dataset. We have shown that harmonization works well also when the available datasets were preprocessed using different pipelines and tools as it often occurs. Harmonization can increase the classification accuracy by up to 20%. This is fully consistent with the original paper, which proposed the harmonization method regRCPqn [49]. When the preprocessing of training and test data is the same, harmonization has almost no effect on the final classification accuracy. It is impossible to guarantee that all new datasets on which the model will be tested will have the same preprocessing. Even with tools such as limma [107], ComBat [108], it may not be possible to remove technical signal when batches are mixed with variables of interest. Applying ComBat to high-throughput data with an uneven study design may actually result in false signals [109]. To solve the classification problem, different models were tested, classical ones (logistic regression and support-vector machine), different gradient-boosted decision trees and deep neural networks. For all models, a hyperparametric search was performed to select the optimal set of parameters. The best results of weighted accuracy were obtained with tree ensembles, which is consistent with other works [110]. For Parkinson's disease, the accuracy of classifying patients and healthy controls was higher than 95%. The accuracy for schizophrenia was much lower, only >70%. Unlike Parkinson's disease, schizophrenia is a complex disease characterized by a variety of different symptoms. A large number of different causes and molecular patterns determining the development of this pathology are being identified. The etiology of schizophrenia is multifactorial and reflects an interaction between genetic vulnerability and environmental influences. Environmental risk factors, such as complications of pregnancy and childbirth, childhood trauma, migration, social isolation, urban life, and substance abuse, either singly or in combination over time, affect the likelihood of an individual developing the disorder. A lot of genes have been previously identified as responsible for the risk of developing this pathology, so the personal molecular landscape is highly individualized and complicated by interactions between genes and the environment. These reasons may be related to the relatively low accuracy for schizophrenia in our study compared to Parkinson's disease [111, 112, 113]. Another reason that affects the

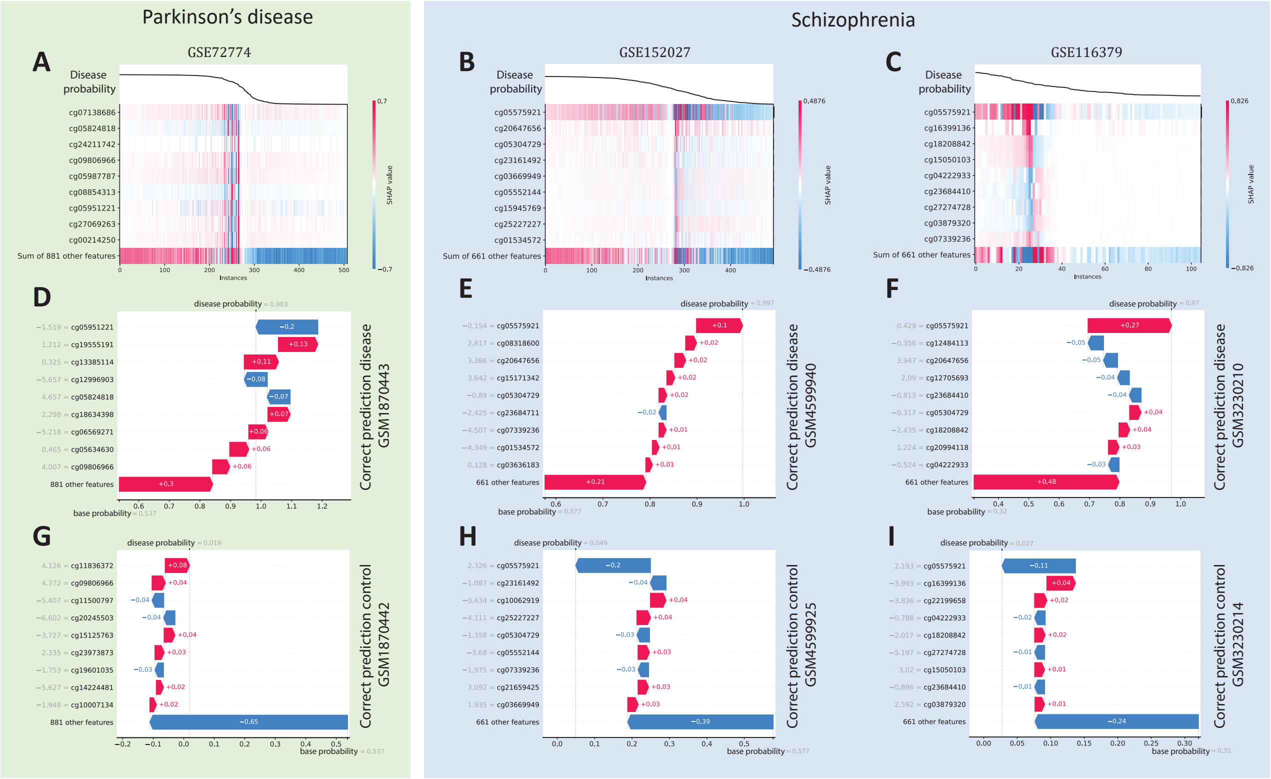

Figure 6. Local explainability based on SHAP values. (A) Black lines show the probability of predicting Parkinson's disease for different participants. Heatmaps in color show the contribution of SHAP values to the probability of predicting Parkinson's disease for different participants and CpG sites for GSE72774. (B) Probability of predicting schizophrenia and heatmaps of contribution for GSE152027. (C) Probability of predicting schizophrenia and heatmaps of contribution for GSE116379. (D) Waterfall plot for participant GSM1870443 with Parkinson's disease, showing the contribution of individual CpG sites to changes in the probability of predicting disease. (E) Waterfall plot for participant GSM4599940 with schizophrenia. (F) Waterfall plot for participant GSM3230210 with schizophrenia. In all cases, the models' confidence is over 97%. (G) Waterfall plot for control participant GSM1870442 showing the contribution of individual CpG sites to the change in probability of predicting disease. (H) Waterfall plot for control participant GSM4599925. (I) Waterfall plot for control participant GSM3230214. In all cases, the probability of predicting disease is below 5%.

result may be the number of samples in the training set. The training set for schizophrenia has fewer samples compared to Parkinson's disease. In this case, the data may not be representative enough to fully describe the disease characteristics for the model. Since the best models allow us to obtain the importance values of each feature for classification, we constructed a CpG site ranking for each of the diseases. Based on this ranking, we reduced the dimensionality of the classification models, since many of the most common epigenetic models (such as the epigenetic clocks) contain relatively few CpG sites (up to 1000). To find optimal small models, we performed a series of experiments for an increasing number of features from 10 to 1000. Based on the dependence of the weighted classification accuracy on the number of features, we determined the optimal models, with the accuracy for both diseases changing not much, while the number of features decreased significantly. Optimal small models contain 890 CpG sites for Parkinson's disease and 670 CpG sites for Schizophrenia. Such small models are much less memory-consuming and have better computational performance. For machine learning models (both small and large), missing values are critical. A model trained on a particular set of features must necessarily be tested on the same set. If, for certain reasons (data collection or processing errors, low signal intensities, or other problems), some CpG sites are missing from the new data for testing, imputation methods are applied. The best imputation methods recover almost the same classification accuracy as for the original data. Since machine learning models work as a "black box", special methods must be used to explain exactly how the model makes predictions. Otherwise, the model cannot be trusted and it is impossible to identify the nature of errors. If the predictive capabilities of the model can be explained, it can help to discover complex relationships between biomarkers. The calculation of SHAP values allowed us to obtain both global and local explainability. Globally, the prediction of healthy control or patient with Parkinson's disease is affected by several CpG sites, there are examples of both types of influence, positive and negative. Global explainability for healthy controls and patients with schizophrenia confirmed the strongest influence of one CpG site, cg05575921, while the others had little effect on model prediction. This CpG site has previously been reported to be associated with schizophrenia and post-traumatic stress disorder (PTSD) [85, 114]. Examples of local explainability - specific predictions for certain participants - are also given.

Thus, we propose a methodologically valid and complete approach for classifying healthy people and patients with various diseases, which allows to harmonize DNA methylation data from different sources, impute missing values, reduce dimensionality of the models, and apply explainable artificial intelligence approaches. The proposed algorithm works better for Parkinson's disease than for schizophrenia, which is characterized by a variety of different symptoms. Further work may include expanding the pool of considered diseases, enriching the library of methods at different stages of the workflow. Another future challenge is advancing from explainability to interpretability. The former, currently implemented, uncovers the internal "mechanics" of a system. The yet missing interpretability would predict the outcome of changing the input or algorithmic parameters.

Limitations

The proposed approach has several limitations relevant at different steps. First, it should be noted that the accuracy gain from harmonization will be limited if the preprocessing of training and test data is the same. Second, the constructed classification models may not be globally optimal in terms of quality metrics, because we are considering a limited number of parameters to vary within a hyperparametric search, each varying within a limited range of values near defaults. Third, besides choosing the top best features to reduce dimensionality, it might be better to consider different combinations of them. However, in this case, the number of considered models would increase dramatically. Fourth, using schizophrenia as an example, it was shown that if there are some most important CpG sites significantly overcoming the importance of the other, and if they are missed, imputation can not help to improve the result.

Methods

Datasets

We reviewed publicly available whole blood DNA methylation datasets in the GEO repository [115], which include the largest ones from patients with Parkinson's disease and schizophrenia, with at least 50 participants in each group. The following datasets comprise whole blood samples from subjects with Parkinson's disease and healthy controls: GSE145361 [79], GSE111629 [80, 81, 82], GSE72774 [80, 81, 83]. The following datasets comprise whole blood samples from subjects with schizophrenia and healthy controls: GSE152027 [84], GSE84727 [84, 85], GSE80417 [84, 85], GSE116379 (only non famine participants) [86]. We remove from the analysis non-CpG probes [116], SNP-related probes [117], multi-hit probes [118], probes on chromosomes X and Y. We consider only common CpGs in train datasets, the number of CpGs in test datasets can be different.The remaining amount of CpGs after all filtration procedures is shown in Table 1.

Data harmonization

Meta-analysis can be done in different ways: some approaches first analyze different datasets separately and then combine the results into a final estimate; others first combine data from all sets and then analyze the combined data using a single model. The first class of approaches includes aggregated data and two-step meta-analysis of individual participant data (IPD) [119]. The major advantage of these approaches is their relatively low implementation complexity, while the major disadvantage is the need for raw data. This approach often uses not all available data, but only a subset (usually differentially methylated positions, DMPs, or differentially methylated regions, DMRs). The second class of approaches is called single-step IPD meta-analysis. Although single-step IPD approaches are expected to behave similarly to two-step IPD [119], they provide additional flexibility (e.g., no need to start with raw data) and enable comparison between different models [119]. An important assumption of single-step IPD meta-analysis is the comparability of variables measured in different datasets [120], and therefore data harmonization is crucial to ensure that methylation samples of the same type (same tissue, health status, age, gender, etc.) from different datasets can be compared.

We use the approach to data harmonization proposed in [49], a one-step IPD approach for systematically assessing the impact of different preprocessing methods on the meta-analysis. It has been shown that data preprocessing by different algorithms has a significant impact. RegRCPqn (regional regression on correlated probes with quantile normalization) does not require raw idat files and can be applied to datasets with only β-values or M-values available, which is a common scenario from real life [49]. RCP [121] is a within-array normalization that uses the spatial correlation of DNA methyla-

tion at CpG sites to estimate the calibration transformation between type I and type II intensities. The regRCPqn procedure improves the RCP algorithm by including three functions to solve the problem under study. First, it calculates the RCP normalization separately for each type of genomic region (i.e., for CpG belonging to islands, shores, shelves, or open seas) because the distribution of DNA methylation values is different in each of these types of regions [122]. It then performs a quantile normalization between samples, in which CpG values for all samples are normalized separately for each CpG region and for type I and type II probes. Finally, it introduces the possibility of storing the reference distribution and using it to perform quantile normalization of samples from another dataset based on the reference. The reference distribution is calculated separately for each area type and for type I and type II probes. When possible, this distribution is used by regRCPqn to perform normalization based on the reference, again separately for each region and probe type. The dataset with the highest number of participants for each disease is used as the reference, and the others are harmonized relative to it. We consider CpG sites whose distribution of methylation levels does not differ in different train datasets in the control group. To find them, we performed the Mann-Whitney U-test [88] for all CpG sites included in the train datasets for the control group and took CpG sites for which the p-value adjusted according to the Benjamini-Hochberg procedure [123] >0.05. The Mann-Whitney U-test was performed using the scipy package version 1.8.0.

Classification models

The most common type of data for machine learning and deep learning tasks is tabular data, which comprises samples (rows) with the same set of features (columns). DNA methylation is an example of this type of data. Tabular data, unlike image or speech data, is heterogeneous, resulting in dense numerical and sparse categorical features. In addition, the correlation between features is weaker than the spatial or semantic relationship in image or speech data [124]. Variables can be correlated or independent, and features have no positional information. Consequently, it is necessary to detect and use correlation without relying on spatial information [94, 95]. During the last decade, traditional machine learning methods such as gradient-boosted decision trees (GBDT) [91] have continued to dominate tabular data modeling and have demonstrated better performance than deep learning [110]. GBDT trains a series of weak learners to predict the outcome. In GBDT, the weak learner is a standard decision tree that lacks differentiability. Despite their differences, their performance on many problems is similar [92]. When deep neural networks are applied to tabular data, many problems arise, such as lack of locality, missing values, mixed object types (numeric, ordinal and categorical), lack of prior knowledge about the structure of the data. Tree ensemble algorithms are considered a recommended option for real-world problems with tabular data [91, 92, 125]. The XGBoost algorithm [91] is an extendible gradient boosting tree algorithm that achieves state-of-the-art results on many tabular datasets [126, 127].

We consider the following classification models: Logistic Regression with elastic net penalty [89], Support-Vector Machine [90], XGBoost [91], CatBoost [92], LightGBM [93], TabNet [94], NODE [95]. Despite the name, logistic regression is used to solve the binary classification problem. It is a generalized linear model, showing good results for linearly separable data. Support-vector machine is a supervised learning model whose main goal is to construct a separating manifold. This method allows the use of different kernel functions to achieve the best results. XGBoost (Extreme Gradient Boosting) is a scalable, distributed gradient-boosted decision tree (GBDT) machine learning library. GBDT iteratively trains an ensemble of shallow decision trees, with each iteration using error residuals from the previous model to fit the next model. The final prediction is a weighted sum of all tree predictions. XGBoost has one of the best combinations of prediction performance and processing time. CatBoost is an open-source gradient boosting algorithm, which builds symmetric (balanced) trees. At each step, the leaves of the previous tree are separated by the same condition. A feature-split pair is selected and used for all nodes, which provides the least losses. This balanced tree architecture reduces prediction time and controls overfitting. LightGBM is a fast, distributed, high-performance gradient boosting platform that supports the decision tree algorithm. It splits the tree by leaf with the simplest fit, whereas other boosting algorithms split the tree by depth or by level rather than by leaf. Thus, when growing on an equivalent leaf in LightGBM, a leaf-based algorithm can reduce more losses than a level-based algorithm, and therefore lead to greater accuracy. TabNet is a deep neural network designed to handle tabular data. TabNet inputs raw tabular data with no preprocessing and is trained using gradient descent-based optimization. It uses sequential attention to select features at each decision step, providing interpretability and better learning as the learning capability is used for the most useful features, with instance-specific feature selection. Neural Oblivious Decision Ensembles (NODE) is a deep learning architecture designed to handle tabular data. The NODE architecture generalizes ensembles of oblivious decision trees, but benefits from both end-to-end gradient-based optimization and multilevel hierarchical learning capabilities. All of the above models can handle continuous variables (without categorical ones). For classification, we use only the DNA methylation levels of the different CpG sites, which are continuous variables. Parameter values of the trained models which were found by hyperparametric search can be found in Supplementary Table S1. All models have been trained for 2,000 epochs.

Each model was trained on two training datasets and then tested on the remaining independent datasets. Hyperparametric search was used to find optimal parameters for the models. There are a lot of quality metrics for the classification problem: accuracy, precision, recall, f1 score, Cohen's kappa, Matthews correlation coefficient, AUROC, etc. As the main metric, we choose accuracy with weighted averaging to take into account the possible imbalance of the classes. It is calculated according to the formula:

$$\frac{N_{cases}}{N}Accuracy_{cases} + \frac{N_{controls}}{N}Accuracy_{controls}, \quad (1)$$

where N_{cases} is the total number of cases, $N_{controls}$ is total the number of controls, N is the total number of participants. The accuracy for each class is:

$$Accuracy = \frac{TP + TN}{N}, \quad (2)$$

where TP is the number of true positives and TN is the number of true negatives. Adam optimizer and StepLR scheduler were used for the neural network models [128]. Used versions of software packages for the models: XGBoost 1.5.2, CatBoost 1.0.4, LightGBM 3.3.2, TabNet 3.1.1, PyTorch 1.10.0, PyTorch Lightning 1.6.0.

Dimensionality reduction

Based on the features ranking, we performed dimensionality reduction of the models. We performed it, leaving only the most important CpGs for solving the classification problem. For this purpose, we built a series of models with different numbers of features. First, for each disease, we choose the top 10 most important features, and a new model is built for them (the type of model is chosen beforehand - it is the best in terms of accuracy for the full data). Then new models are built on the number of features from 10 to 1000 in increments of 10, and for each such model, the weighted classification accuracy is calculated. For all these models hyperparametric search was performed. According to the dependence of weighted classification accuracy on the number of features we chose as optimal the number of features for which the highest weighted classification accuracy is observed for the considered diseases.

Imputation of missing values

Missing data can be divided into three classes [129]: i) missing completely at random (MCAR) values, if the probability of absence is completely independent of both observed and unobserved variables; ii) missing at random (MAR) values, if the probability of absence is independent of the value itself, but may depend on observed variables; iii) missing not at random (MNAR), if the probability of absence depends on the missing value itself. There is currently no statistical way to determine which category the specific missing data falls into. Assumptions are usually made based on knowledge of the data and the data collection and processing procedure. It is assumed that the missing values represent MCAR/MAR due to random experimental and technology-related errors [53]. It has been shown that missing values lying at the midrange methylation level are more difficult to impute than missing values close to the extremes of the range [54]. This is probably a consequence of the higher variance of methylation values in the middle ranges. Such a scenario could have a profound effect in terms of performance expectations, assuming that many missing values in the data are of the MNAR type and, in particular, lie in the middle range of β values.

In general terms, imputation approaches can be divided into single (SI) and multiple imputation (MI) methods. SI methods replace a missing value with a single acceptable value. MI methods perform multiple SIs and average parameter estimates over multiple imputations to produce a single estimate. Under MCAR/MAR assumptions, the most common imputation methods like mean, median or mode can handle missing data [130]. Such simple imputation methods are used often [131], but they can lead to systematic error or unrealistic results for multivariate datasets. In addition, for large data, this method often performs poorly [132]. The expectation maximization method is an iterative method for handling missing values in numerical datasets, and the algorithm uses an "impute, estimate, and iterate until convergence" approach. Each iteration involves two steps: expectation and maximization. Expectation estimates the missing values given the observed data, while maximization uses the current estimated values to maximize the probability of all data [133, 134, 135]. Besides classical methods, there are approaches to multiple imputation, for example, chained equation for big data [132]. Hot-deck imputation handles missing values by matching missing values with other values in the dataset for several other key variables that have complete values [136, 137]. However, this method does not account for the variability of the missing data. One of the common hot-deck methods is K Nearest Neighbours (KNN) [138]. The KNN algorithm works by classifying the nearest neighbors of missing values and using those neighbors for imputation using a distance measure between instances [139]. Several distance measures can be used for KNN imputation, but Euclidean distance has been shown to provide efficiency and performance [140] and is therefore the most widely used distance measure. However, KNN imputation has weaknesses, such as poor accuracy when imputing variables and introducing false associations where none exists [141]. Another weakness of KNN imputation is that it scans the entire dataset, which increases computation time [142]. However, there are approaches developed in the literature to improve the KNN imputation algorithm [143, 144, 145, 146, 147, 148, 149]. All imputation methods that can deal with continuous variables are suitable for imputing DNA methylation data [53]. To study the effect of these methods on classification accuracy, we removed from consideration 100 CpG sites with the highest importance values for each disease and tried to fill them in. We used previously constructed small models for both diseases. Imputation methods were applied by impyute package version 0.0.8.

Explainable artificial intelligence

Modern machine-learning-based artificial intelligence systems are usually treated as black boxes. However, every decision must be made available for verification by a human expert [150]. One important aspect of model explainability is the ability to verify the system. For example, in healthcare, the use of models that can be interpreted and verified by medical experts is an absolute necessity [151]. Another aspect is to improve the system. The first step to improving the AI system is to understand its weaknesses. Performing weakness analysis on black box models is more difficult than on models that can be interpreted. Furthermore, model interpretability can be useful when comparing different models or architectures [152, 153, 154]. It can be argued that the better we understand what models do (and why they sometimes fail), the easier it becomes to improve them [150]. The next important aspect of explainability is the ability to learn from the system: since modern AI systems learn from millions of examples, they can observe patterns in the data that are inaccessible to humans, who can only learn from a limited number of examples [155, 156]. Explainability is also important for other machine learning methods beyond neural networks [152].

One of the taxonomies to classify explanatory methods is global and local methods [64, 65, 150]. Local interpretable methods apply to a single model result; they can explain the reason for a particular prediction or result. In contrast, global methods try to explain the behavior of the model as a whole. Perturbation is the easiest way to analyze the effect of changing input features on the AI model outputs. This can be accomplished by removing or changing certain input features, running a forward pass, and measuring the difference with the original output data. The input characteristics that most affect the output are evaluated as the most important ones. This is computationally costly, since a direct pass must be run after perturbing each group of input features. Such a perturbation-based approach is Shapley value sampling, which computes approximate Shapley values by taking each input feature for a certain number of times. It is a method from game theory that describes a fair distribution of wins and losses between input functions [157]. As a result, it is not a practical method in its original form, but has led to the development of methods based on game theory, such as Deep SHapley Additive exPlanations (SHAP) [105]. SHAP has an alternative kernel-based approach to estimating Shapley values inspired by local surrogate models. There is also TreeSHAP, an efficient approach to estimating tree models, as well as DeepExplainer, an enhanced version of

the DeepLIFT algorithm for deep neural networks. For the constructed portable models, we applied SHAP to obtain global and local explainability. SHAP values were calculated using eponymous package version 0.40.0.

Data availability statement

No new data was generated. Data used in this study are available from the GEO database (accession numbers GSE145361, GSE111629, GSE72774, GSE84727, GSE80417, GSE152027, GSE116379).

Code availability statement

The source code for the analysis workflow presented in the manuscript is publicly available.

- Project name: DNAmClassMeta
- Project home page: https://github.com/GillianGrayson/DNAmClassMeta
- Operating system(s): Platform independent
- Programming language: Python
- Other requirements: Python 3.8 or higher, pytorch-lightning 1.5.10 or higher, xgboost 1.6.0 or higher, catboost 1.0.5 or higher, lightgbm 3.3.2 or higher, scikit-learn 1.0.2 or higher. All requirements are listed in the requirements.txt file in the project home page.
- License: MIT

Abbreviations

AI: Artificial Intelligence; CatBoost: Categorical Boosting; DMP: Differentially Methylated Position; DMR: Differentially Methylated Region; DNAm: DNA methylation; EWAS: Epigenome-Wide Association Study; FDR: False Discovery Rate; GBDT: Gradient-Boosted Decision Tree; IPD: Individual Participant Data; KNN: K Nearest Neighbors; LightGBM: Light Gradient Boosting Machine; MAR: Missing At Random; MCAR: Missing Completely At Random; MI: Multiple Imputation; MNAR: Missing Not At Random; NODE: Neural Oblivious Decision Ensemble; PTSD: Post-Traumatic Stress Disorder; RCP: Regression on Correlated Probes; SHAP: Shapley Additive Explanations; SI: Single Imputation; XAI: Explainable Artificial Intelligence; XGBoost: Extreme Gradient Boosting.

Competing Interests

The authors declare that they have no competing interests.

Funding

The research was supported by the Ministry of Science and Higher Education of the Russian Federation, Grant for Major Research Projects in Priority Areas of Scientific and Technological Development No. 075-15-2020-808, grant recipient: Lobachevsky State University.

Author's Contributions

Conceptualization: A.K., I.Y., M.G.B., M.I.; Formal analysis: A.K., I.Y.; Methodology: A.K., I.Y., M.G.B.; Software: A.K., I.Y.; Supervision: M.G.B., C.F., M.V., M.I.; Visualization: A.K., I.Y.; Writing – original draft: A.K., I.Y.; Writing – review & editing: A.K., I.Y., M.G.B., C.F., M.V., M.I.

Acknowledgements

The authors acknowledge the use of computational resources provided by the "Lobachevsky" supercomputer.

References

1. Sasaki H, Matsui Y. Epigenetic Events in Mammalian Germ-Cell Development: Reprogramming and Beyond. Nature Reviews Genetics 2008 Feb;9(2):129–140.
2. Igarashi J, Muroi S, Kawashima H, Wang X, Shinojima Y, Kitamura E, et al. Quantitative Analysis of Human Tissue-Specific Differences in Methylation. Biochemical and Biophysical Research Communications 2008 Nov;376(4):658–664.
3. Zemach A, McDaniel IE, Silva P, Zilberman D. Genome-Wide Evolutionary Analysis of Eukaryotic DNA Methylation. Science 2010 May;328(5980):916–919.
4. Ziller MJ, Gu H, Müller F, Donaghey J, Tsai LTY, Kohlbacher O, et al. Charting a Dynamic DNA Methylation Landscape of the Human Genome. Nature 2013 Aug;500(7463):477–481.
5. Horvath S. DNA Methylation Age of Human Tissues and Cell Types. Genome Biology 2013;14(10):R115.
6. Orozco LD, Farrell C, Hale C, Rubbi L, Rinaldi A, Civelek M, et al. Epigenome-Wide Association in Adipose Tissue from the METSIM Cohort. Human Molecular Genetics 2018 Jul;27(14):2586–2586.
7. Smith ZD, Meissner A. DNA Methylation: Roles in Mammalian Development. Nature Reviews Genetics 2013 Mar;14(3):204–220.
8. Lim DHK, Maher ER. Genomic Imprinting Syndromes and Cancer. In: Advances in Genetics, vol. 70 Elsevier; 2010.p. 145–175.
9. Robertson KD. DNA Methylation and Human Disease. Nature Reviews Genetics 2005 Aug;6(8):597–610.
10. Jones PA. Functions of DNA Methylation: Islands, Start Sites, Gene Bodies and Beyond. Nature Reviews Genetics 2012 Jul;13(7):484–492.
11. Jjingo D, Conley AB, Yi SV, Lunyak VV, Jordan IK. On the Presence and Role of Human Gene-Body DNA Methylation. Oncotarget 2012 Apr;3(4):462–474.
12. Christensen BC, Houseman EA, Marsit CJ, Zheng S, Wrensch MR, Wiemels JL, et al. Aging and Environmental Exposures Alter Tissue-Specific DNA Methylation Dependent upon CpG Island Context. PLoS Genetics 2009 Aug;5(8):e1000602.
13. Bell CG, Lowe R, Adams PD, Baccarelli AA, Beck S, Bell JT, et al. DNA Methylation Aging Clocks: Challenges and Recommendations. Genome Biology 2019 Dec;20(1):249.
14. Rakyan VK, Down TA, Balding DJ, Beck S. Epigenome-Wide Association Studies for Common Human Diseases. Nature Reviews Genetics 2011 Jul;12(8):529–541.
15. Liu D, Zhao L, Wang Z, Zhou X, Fan X, Li Y, et al. EWASdb: Epigenome-Wide Association Study Database. Nucleic Acids Research 2019 Jan;47(D1):D989–D993.
16. Birney E, Smith GD, Greally JM. Epigenome-Wide Association Studies and the Interpretation of Disease -Omics. PLOS Genetics 2016 Jun;12(6):e1006105.
17. Moran S, Arribas C, Esteller M. Validation of a DNA Methylation Microarray for 850,000 CpG Sites of the Human Genome Enriched in Enhancer Sequences. Epigenomics 2016 Mar;8(3):389–399.
18. Bibikova M, Lin Z, Zhou L, Chudin E, Garcia EW, Wu

B, et al. High-Throughput DNA Methylation Profiling Using Universal Bead Arrays. Genome Research 2006 Mar;16(3):383–393.
19. Irizarry RA, Ladd-Acosta C, Carvalho B, Wu H, Brandenburg SA, Jeddeloh JA, et al. Comprehensive High-Throughput Arrays for Relative Methylation (CHARM). Genome Research 2008 May;18(5):780–790.
20. Du P, Zhang X, Huang CC, Jafari N, Kibbe WA, Hou L, et al. Comparison of Beta-value and M-value Methods for Quantifying Methylation Levels by Microarray Analysis. BMC Bioinformatics 2010 Dec;11(1):587.
21. Tian T, Wan J, Song Q, Wei Z. Clustering Single-Cell RNA-seq Data with a Model-Based Deep Learning Approach. Nature Machine Intelligence 2019 Apr;1(4):191–198.
22. Lopez R, Regier J, Cole MB, Jordan MI, Yosef N. Deep Generative Modeling for Single-Cell Transcriptomics. Nature Methods 2018 Dec;15(12):1053–1058.
23. Way GP, Greene CS. Extracting a Biologically Relevant Latent Space from Cancer Transcriptomes with Variational Autoencoders. Pacific Symposium on Biocomputing Pacific Symposium on Biocomputing 2018;23:80–91.
24. Titus AJ, Wilkins OM, Bobak CA, Christensen BC. Unsupervised Deep Learning with Variational Autoencoders Applied to Breast Tumor Genome-Wide DNA Methylation Data with Biologic Feature Extraction. Bioinformatics; 2018.
25. Ching T, Himmelstein DS, Beaulieu-Jones BK, Kalinin AA, Do BT, Way GP, et al. Opportunities and Obstacles for Deep Learning in Biology and Medicine. Journal of The Royal Society Interface 2018 Apr;15(141):20170387.
26. Levy JJ, Titus AJ, Petersen CL, Chen Y, Salas LA, Christensen BC. MethylNet: An Automated and Modular Deep Learning Approach for DNA Methylation Analysis. BMC Bioinformatics 2020 Dec;21(1):108.
27. The Cancer Genome Atlas Research Network, Weinstein JN, Collisson EA, Mills GB, Shaw KRM, Ozenberger BA, et al. The Cancer Genome Atlas Pan-Cancer Analysis Project. Nature Genetics 2013 Oct;45(10):1113–1120.
28. Ding W, Chen G, Shi T. Integrative Analysis Identifies Potential DNA Methylation Biomarkers for Pan-Cancer Diagnosis and Prognosis. Epigenetics 2019 Jan;14(1):67–80.
29. Celli F, Cumbo F, Weitschek E. Classification of Large DNA Methylation Datasets for Identifying Cancer Drivers. Big Data Research 2018 Sep;13:21–28.
30. Ma B, Meng F, Yan G, Yan H, Chai B, Song F. Diagnostic Classification of Cancers Using Extreme Gradient Boosting Algorithm and Multi-Omics Data. Computers in Biology and Medicine 2020 Jun;121:103761.
31. List M, Hauschild AC, Tan Q, Kruse TA, Baumbach J, Batra R. Classification of Breast Cancer Subtypes by Combining Gene Expression and DNA Methylation Data. Journal of Integrative Bioinformatics 2014 Jun;11(2):1–14.
32. Dong RZ, Yang X, Zhang XY, Gao PT, Ke AW, Sun Hc, et al. Predicting Overall Survival of Patients with Hepatocellular Carcinoma Using a Three-category Method Based on DNA Methylation and Machine Learning. Journal of Cellular and Molecular Medicine 2019 May;23(5):3369–3374.
33. Hao X, Luo H, Krawczyk M, Wei W, Wang W, Wang J, et al. DNA Methylation Markers for Diagnosis and Prognosis of Common Cancers. Proceedings of the National Academy of Sciences 2017 Jul;114(28):7414–7419.
34. Jurmeister P, Bockmayr M, Seegerer P, Bockmayr T, Treue D, Montavon G, et al. Machine Learning Analysis of DNA Methylation Profiles Distinguishes Primary Lung Squamous Cell Carcinomas from Head and Neck Metastases. Science Translational Medicine 2019 Sep;11(509):eaaw8513.
35. Wajed SA, Laird PW, DeMeester TR. DNA Methylation: An Alternative Pathway to Cancer:. Annals of Surgery 2001 Jul;234(1):10–20.
36. Bollepalli S, Korhonen T, Kaprio J, Anders S, Ollikainen M. EpiSmokEr: A Robust Classifier to Determine Smoking Status from DNA Methylation Data. Epigenomics 2019 Oct;11(13):1469–1486.
37. Lee YC, Christensen JJ, Parnell LD, Smith CE, Shao J, McKeown NM, et al. Using Machine Learning to Predict Obesity Based on Genome-Wide and Epigenome-Wide Gene–Gene and Gene–Diet Interactions. Frontiers in Genetics 2022 Jan;12:783845.
38. Aref-Eshghi E, Rodenhiser DI, Schenkel LC, Lin H, Skinner C, Ainsworth P, et al. Genomic DNA Methylation Signatures Enable Concurrent Diagnosis and Clinical Genetic Variant Classification in Neurodevelopmental Syndromes. The American Journal of Human Genetics 2018 Jan;102(1):156–174.
39. Dogan MV, Grumbach IM, Michaelson JJ, Philibert RA. Integrated Genetic and Epigenetic Prediction of Coronary Heart Disease in the Framingham Heart Study. PLOS ONE 2018 Jan;13(1):e0190549.
40. Gunasekara CJ, Hannon E, MacKay H, Coarfa C, McQuillin A, Clair DS, et al. A Machine Learning Case–Control Classifier for Schizophrenia Based on DNA Methylation in Blood. Translational Psychiatry 2021 Dec;11(1):412.
41. Jabari S, Kobow K, Pieper T, Hartlieb T, Kudernatsch M, Polster T, et al. DNA Methylation-Based Classification of Malformations of Cortical Development in the Human Brain. Acta Neuropathologica 2022 Jan;143(1):93–104.
42. Jo T, Nho K, Bice P, Saykin AJ, For The Alzheimer's Disease Neuroimaging Initiative. Deep Learning-Based Identification of Genetic Variants: Application to Alzheimer's Disease Classification. Briefings in Bioinformatics 2022 Mar;23(2):bbac022.
43. Haghshenas S, Bhai P, Aref-Eshghi E, Sadikovic B. Diagnostic Utility of Genome-Wide DNA Methylation Analysis in Mendelian Neurodevelopmental Disorders. International Journal of Molecular Sciences 2020 Dec;21(23):9303.
44. Xiong Z, Zhang X, Zhang M, Cao B. Predicting Features of Human Mental Disorders through Methylation Profile and Machine Learning Models. In: 2020 2nd International Conference on Machine Learning, Big Data and Business Intelligence (MLBDBI) Taiyuan, China: IEEE; 2020. p. 67–75.
45. Luo X, Wei Y. Batch Effects Correction with Unknown Subtypes. Journal of the American Statistical Association 2019 Apr;114(526):581–594.
46. Leek JT, Scharpf RB, Bravo HC, Simcha D, Langmead B, Johnson WE, et al. Tackling the Widespread and Critical Impact of Batch Effects in High-Throughput Data. Nature Reviews Genetics 2010 Oct;11(10):733–739.
47. Perrier F, Novoloaca A, Ambatipudi S, Baglietto L, Ghantous A, Perduca V, et al. Identifying and Correcting Epigenetics Measurements for Systematic Sources of Variation. Clinical Epigenetics 2018 Dec;10(1):38.
48. Zindler T, Frieling H, Neyazi A, Bleich S, Friedel E. Simulating ComBat: How Batch Correction Can Lead to the Systematic Introduction of False Positive Results in DNA Methylation Microarray Studies. BMC Bioinformatics 2020 Dec;21(1):271.
49. Sala C, Di Lena P, Fernandes Durso D, Prodi A, Castellani G, Nardini C. Evaluation of Pre-Processing on the Meta-Analysis of DNA Methylation Data from the Illumina HumanMethylation450 BeadChip Platform. PLOS ONE 2020 Mar;15(3):e0229763.
50. Garagnani P, Bacalini MG, Pirazzini C, Gori D, Giuliani C, Mari D, et al. Methylation of *ELOVL* 2 Gene as a New

Epigenetic Marker of Age. Aging Cell 2012 Dec;11(6):1132–1134.
51. Hannum G, Guinney J, Zhao L, Zhang L, Hughes G, Sadda S, et al. Genome-Wide Methylation Profiles Reveal Quantitative Views of Human Aging Rates. Molecular Cell 2013 Jan;49(2):359–367.
52. Weidner C, Lin Q, Koch C, Eisele L, Beier F, Ziegler P, et al. Aging of Blood Can Be Tracked by DNA Methylation Changes at Just Three CpG Sites. Genome Biology 2014;15(2):R24.
53. Di Lena P, Sala C, Prodi A, Nardini C. Missing Value Estimation Methods for DNA Methylation Data. Bioinformatics 2019 Oct;35(19):3786–3793.
54. Lena PD, Sala C, Prodi A, Nardini C. Methylation Data Imputation Performances under Different Representations and Missingness Patterns. BMC Bioinformatics 2020 Dec;21(1):268.
55. Venkat N. The Curse of Dimensionality: Inside Out 2018;http://rgdoi.net/10.13140/RG.2.2.29631.36006.
56. Levine ME, Lu AT, Quach A, Chen BH, Assimes TL, Bandinelli S, et al. An Epigenetic Biomarker of Aging for Lifespan and Healthspan. Aging 2018 Apr;10(4):573–591.
57. Lu AT, Quach A, Wilson JG, Reiner AP, Aviv A, Raj K, et al. DNA Methylation GrimAge Strongly Predicts Lifespan and Healthspan. Aging 2019 Jan;11(2):303–327.
58. Kurdyukov S, Bullock M. DNA Methylation Analysis: Choosing the Right Method. Biology 2016 Jan;5(1):3.
59. He K, Zhang X, Ren S, Sun J. Deep Residual Learning for Image Recognition. In: 2016 IEEE Conference on Computer Vision and Pattern Recognition (CVPR) Las Vegas, NV, USA: IEEE; 2016. p. 770–778.
60. Cho K, van Merrienboer B, Gulcehre C, Bahdanau D, Bougares F, Schwenk H, et al. Learning Phrase Representations Using RNN Encoder–Decoder for Statistical Machine Translation. In: Proceedings of the 2014 Conference on Empirical Methods in Natural Language Processing (EMNLP) Doha, Qatar: Association for Computational Linguistics; 2014. p. 1724–1734.
61. Deng L, Hinton G, Kingsbury B. New Types of Deep Neural Network Learning for Speech Recognition and Related Applications: An Overview. In: 2013 IEEE International Conference on Acoustics, Speech and Signal Processing Vancouver, BC, Canada: IEEE; 2013. p. 8599–8603.
62. Baldi P. Deep Learning in Biomedical Data Science. Annual Review of Biomedical Data Science 2018 Jul;1(1):181–205.
63. Galkin F, Mamoshina P, Kochetov K, Sidorenko D, Zhavoronkov A. DeepMAge: A Methylation Aging Clock Developed with Deep Learning. Aging and Disease 2021 Aug;12(5):1252–1262.
64. Baehrens D, Schroeter T, Harmeling S, Kawanabe M, Hansen K, Müller KR. How to Explain Individual Classification Decisions. The Journal of Machine Learning Research 2010 Aug;11:1803–1831.
65. Simonyan K, Vedaldi A, Zisserman A. Deep Inside Convolutional Networks: Visualising Image Classification Models and Saliency Maps. arXiv:13126034 [cs] 2014 Apr;.
66. Zeiler MD, Fergus R. Visualizing and Understanding Convolutional Networks. In: Fleet D, Pajdla T, Schiele B, Tuytelaars T, editors. Computer Vision – ECCV 2014, vol. 8689 Cham: Springer International Publishing; 2014.p. 818–833.
67. Bach S, Binder A, Montavon G, Klauschen F, Müller KR, Samek W. On Pixel-Wise Explanations for Non-Linear Classifier Decisions by Layer-Wise Relevance Propagation. PLOS ONE 2015 Jul;10(7):e0130140.
68. Shrikumar A, Greenside P, Shcherbina A, Kundaje A. Not Just a Black Box: Learning Important Features Through Propagating Activation Differences. arXiv:160501713 [cs] 2017 Apr;.
69. Mahendran A, Vedaldi A. Visualizing Deep Convolutional Neural Networks Using Natural Pre-images. International Journal of Computer Vision 2016 Dec;120(3):233–255.
70. Lipton ZC. The Mythos of Model Interpretability. arXiv:160603490 [cs, stat] 2017 Mar;.
71. Ribeiro MT, Singh S, Guestrin C. "Why Should I Trust You?": Explaining the Predictions of Any Classifier. arXiv:160204938 [cs, stat] 2016 Aug;.
72. Zintgraf LM, Cohen TS, Adel T, Welling M. Visualizing Deep Neural Network Decisions: Prediction Difference Analysis. arXiv:170204595 [cs] 2017 Feb;.
73. Doshi-Velez F, Kim B. Towards A Rigorous Science of Interpretable Machine Learning. arXiv:170208608 [cs, stat] 2017 Mar;.
74. Montavon G, Samek W, Müller KR. Methods for Interpreting and Understanding Deep Neural Networks. Digital Signal Processing 2018 Feb;73:1–15.
75. Mahendran A, Vedaldi A. Understanding Deep Image Representations by Inverting Them. arXiv:14120035 [cs] 2014 Nov;.
76. Nguyen A, Yosinski J, Clune J. Multifaceted Feature Visualization: Uncovering the Different Types of Features Learned By Each Neuron in Deep Neural Networks. arXiv:160203616 [cs] 2016 May;.
77. Landecker W, Thomure MD, Bettencourt LMA, Mitchell M, Kenyon GT, Brumby SP. Interpreting Individual Classifications of Hierarchical Networks. In: 2013 IEEE Symposium on Computational Intelligence and Data Mining (CIDM) Singapore, Singapore: IEEE; 2013. p. 32–38.
78. Montavon G, Lapuschkin S, Binder A, Samek W, Müller KR. Explaining Nonlinear Classification Decisions with Deep Taylor Decomposition. Pattern Recognition 2017 May;65:211–222.
79. Vallerga CL, Zhang F, Fowdar J, McRae AF, Qi T, Nabais MF, et al. Analysis of DNA Methylation Associates the Cystine–Glutamate Antiporter SLC7A11 with Risk of Parkinson's Disease. Nature Communications 2020 Dec;11(1):1238.
80. Chuang YH, Paul KC, Bronstein JM, Bordelon Y, Horvath S, Ritz B. Parkinson's Disease Is Associated with DNA Methylation Levels in Human Blood and Saliva. Genome Medicine 2017 Dec;9(1):76.
81. Horvath S, Ritz BR. Increased Epigenetic Age and Granulocyte Counts in the Blood of Parkinson's Disease Patients. Aging 2015 Dec;7(12):1130–1142.
82. Chuang YH, Lu AT, Paul KC, Folle AD, Bronstein JM, Bordelon Y, et al. Longitudinal Epigenome-Wide Methylation Study of Cognitive Decline and Motor Progression in Parkinson's Disease. Journal of Parkinson's Disease 2019 May;9(2):389–400.
83. Paul KC, Binder AM, Horvath S, Kusters C, Yan Q, Rosario ID, et al. Accelerated Hematopoietic Mitotic Aging Measured by DNA Methylation, Blood Cell Lineage, and Parkinson's Disease. BMC Genomics 2021 Dec;22(1):696.
84. Hannon E, Dempster EL, Mansell G, Burrage J, Bass N, Bohlken MM, et al. DNA Methylation Meta-Analysis Reveals Cellular Alterations in Psychosis and Markers of Treatment-Resistant Schizophrenia. eLife 2021 Feb;10:e58430.
85. Hannon E, Dempster E, Viana J, Burrage J, Smith AR, Macdonald R, et al. An Integrated Genetic-Epigenetic Analysis of Schizophrenia: Evidence for Co-Localization of Genetic Associations and Differential DNA Methylation. Genome Biology 2016 Dec;17(1):176.
86. Boks MP, Houtepen LC, Xu Z, He Y, Ursini G, Maihofer AX,

et al. Genetic Vulnerability to DUSP22 Promoter Hypermethylation Is Involved in the Relation between in Utero Famine Exposure and Schizophrenia. npj Schizophrenia 2018 Dec;4(1):16.
87. Rauschert S, Raubenheimer K, Melton PE, Huang RC. Machine Learning and Clinical Epigenetics: A Review of Challenges for Diagnosis and Classification. Clinical Epigenetics 2020 Dec;12(1):51.
88. Mann HB, Whitney DR. On a Test of Whether One of Two Random Variables Is Stochastically Larger than the Other. The Annals of Mathematical Statistics 1947 Mar;18(1):50–60.
89. Cox DR. The Regression Analysis of Binary Sequences. Journal of the Royal Statistical Society Series B (Methodological) 1958;20(2):215–242.
90. Cortes C, Vapnik V. Support-Vector Networks. Machine Learning 1995 Sep;20(3):273–297.
91. Chen T, Guestrin C. XGBoost: A Scalable Tree Boosting System. In: Proceedings of the 22nd ACM SIGKDD International Conference on Knowledge Discovery and Data Mining San Francisco California USA: ACM; 2016. p. 785–794.
92. Prokhorenkova L, Gusev G, Vorobev A, Dorogush AV, Gulin A. CatBoost: Unbiased Boosting with Categorical Features. arXiv:170609516 [cs] 2019 Jan;.
93. Ke G, Meng Q, Finley T, Wang T, Chen W, Ma W, et al. LightGBM: A Highly Efficient Gradient Boosting Decision Tree. In: Advances in Neural Information Processing Systems, vol. 30 Curran Associates, Inc.; 2017. .
94. Arik SO, Pfister T. TabNet: Attentive Interpretable Tabular Learning. arXiv:190807442 [cs, stat] 2020 Dec;.
95. Popov S, Morozov S, Babenko A. Neural Oblivious Decision Ensembles for Deep Learning on Tabular Data. arXiv:190906312 [cs, stat] 2019 Sep;.
96. Henderson-Smith A, Fisch KM, Hua J, Liu G, Ricciardelli E, Jepsen K, et al. DNA Methylation Changes Associated with Parkinson's Disease Progression: Outcomes from the First Longitudinal Genome-Wide Methylation Analysis in Blood. Epigenetics 2019 Apr;14(4):365–382.
97. Kaut O, Schmitt I, Tost J, Busato F, Liu Y, Hofmann P, et al. Epigenome-Wide DNA Methylation Analysis in Siblings and Monozygotic Twins Discordant for Sporadic Parkinson's Disease Revealed Different Epigenetic Patterns in Peripheral Blood Mononuclear Cells. neurogenetics 2017 Jan;18(1):7–22.
98. Walton E, Hass J, Liu J, Roffman JL, Bernardoni F, Roessner V, et al. Correspondence of DNA Methylation Between Blood and Brain Tissue and Its Application to Schizophrenia Research. Schizophrenia Bulletin 2016 Mar;42(2):406–414.
99. Hoang HT, Schlager MA, Carter AP, Bullock SL. DYNC1H1 Mutations Associated with Neurological Diseases Compromise Processivity of Dynein–Dynactin–Cargo Adaptor Complexes. Proceedings of the National Academy of Sciences 2017 Feb;114(9).
100. Chen XJ, Xu H, Cooper HM, Liu Y. Cytoplasmic Dynein: A Key Player in Neurodegenerative and Neurodevelopmental Diseases. Science China Life Sciences 2014 Apr;57(4):372–377.
101. Ma Y, Li J, Xu Y, Wang Y, Yao Y, Liu Q, et al. Identification of 34 Genes Conferring Genetic and Pharmacological Risk for the Comorbidity of Schizophrenia and Smoking Behaviors. Aging 2020 Feb;12(3):2169–2225.
102. Peykov S, Berkel S, Schoen M, Weiss K, Degenhardt F, Strohmaier J, et al. Identification and Functional Characterization of Rare SHANK2 Variants in Schizophrenia. Molecular Psychiatry 2015 Dec;20(12):1489–1498.
103. Chen X, Long F, Cai B, Chen X, Chen G. A Novel Relationship for Schizophrenia, Bipolar and Major Depressive Disorder Part 5: A Hint from Chromosome 5 High Density Association Screen. American Journal of Translational Research 2017;9(5):2473–2491.
104. Hindley G, Bahrami S, Steen NE, O'Connell KS, Frei O, Shadrin A, et al. Characterising the Shared Genetic Determinants of Bipolar Disorder, Schizophrenia and Risk-Taking. Translational Psychiatry 2021 Dec;11(1):466.
105. Chen H, Lundberg S, Lee SI. Explaining Models by Propagating Shapley Values of Local Components. arXiv:191111888 [cs, stat] 2019 Nov;.
106. the Australian Imaging Biomarkers and Lifestyle study, the Alzheimer's Disease Neuroimaging Initiative, Nabais MF, Laws SM, Lin T, Vallerga CL, et al. Meta-Analysis of Genome-Wide DNA Methylation Identifies Shared Associations across Neurodegenerative Disorders. Genome Biology 2021 Dec;22(1):90.
107. Smyth GK, Speed T. Normalization of cDNA Microarray Data. Methods 2003 Dec;31(4):265–273.
108. Johnson WE, Li C, Rabinovic A. Adjusting Batch Effects in Microarray Expression Data Using Empirical Bayes Methods. Biostatistics 2007 Jan;8(1):118–127.
109. Price EM, Robinson WP. Adjusting for Batch Effects in DNA Methylation Microarray Data, a Lesson Learned. Frontiers in Genetics 2018 Mar;9:83.
110. Shwartz-Ziv R, Armon A. Tabular Data: Deep Learning Is Not All You Need. Information Fusion 2022 May;81:84–90.
111. Stilo SA, Murray RM. Non-Genetic Factors in Schizophrenia. Current Psychiatry Reports 2019 Oct;21(10):100.
112. Häfner H, an der Heiden W. Epidemiology of Schizophrenia. The Canadian Journal of Psychiatry 1997 Mar;42(2):139–151.
113. Khan Z, Martin-Montañez E, Muly E. Schizophrenia: Causes and Treatments. Current Pharmaceutical Design 2013-09-31;19(36):6451–6461.
114. INTRuST Clinical Consortium, VA Mid-Atlantic MIRECC Workgroup, PGC PTSD Epigenetics Workgroup, Smith AK, Ratanatharathorn A, Maihofer AX, et al. Epigenome-Wide Meta-Analysis of PTSD across 10 Military and Civilian Cohorts Identifies Methylation Changes in AHRR. Nature Communications 2020 Dec;11(1):5965.
115. Barrett T, Troup DB, Wilhite SE, Ledoux P, Rudnev D, Evangelista C, et al. NCBI GEO: Archive for High-Throughput Functional Genomic Data. Nucleic Acids Research 2009 Jan;37(Database):D885–D890.
116. McCartney DL, Walker RM, Morris SW, McIntosh AM, Porteous DJ, Evans KL. Identification of Polymorphic and Off-Target Probe Binding Sites on the Illumina Infinium MethylationEPIC BeadChip. Genomics Data 2016 Sep;9:22–24.
117. Zhou W, Laird PW, Shen H. Comprehensive Characterization, Annotation and Innovative Use of Infinium DNA Methylation BeadChip Probes. Nucleic Acids Research 2016 Oct;p. gkw967.
118. Nordlund J, Bäcklin CL, Wahlberg P, Busche S, Berglund EC, Eloranta ML, et al. Genome-Wide Signatures of Differential DNA Methylation in Pediatric Acute Lymphoblastic Leukemia. Genome Biology 2013;14(9):r105.
119. Stewart GB, Altman DG, Askie LM, Duley L, Simmonds MC, Stewart LA. Statistical Analysis of Individual Participant Data Meta-Analyses: A Comparison of Methods and Recommendations for Practice. PLoS ONE 2012 Oct;7(10):e46042.
120. Smith-Warner SA, Spiegelman D, Ritz J, Albanes D, Beeson WL, Bernstein L, et al. Methods for Pooling Results of Epidemiologic Studies. American Journal of Epidemiology 2006 Jun;163(11):1053–1064.

121. Niu L, Xu Z, Taylor JA. RCP: A Novel Probe Design Bias Correction Method for Illumina Methylation BeadChip. Bioinformatics 2016 Sep;32(17):2659–2663.
122. Touleimat N, Tost J. Complete Pipeline for Infinium ® Human Methylation 450K BeadChip Data Processing Using Subset Quantile Normalization for Accurate DNA Methylation Estimation. Epigenomics 2012 Jun;4(3):325–341.
123. Benjamini Y, Hochberg Y. Controlling the False Discovery Rate: A Practical and Powerful Approach to Multiple Testing. Journal of the Royal Statistical Society Series B (Methodological) 1995;57(1):289–300.
124. Borisov V, Leemann T, Seßler K, Haug J, Pawelczyk M, Kasneci G. Deep Neural Networks and Tabular Data: A Survey. arXiv:211001889 [cs] 2022 Feb;.
125. Friedman JH. Greedy Function Approximation: A Gradient Boosting Machine. The Annals of Statistics 2001 Oct;29(5).
126. Zhao Y, Chetty G, Tran D. Deep Learning with XGBoost for Real Estate Appraisal. In: 2019 IEEE Symposium Series on Computational Intelligence (SSCI) Xiamen, China: IEEE; 2019. p. 1396–1401.
127. Santhanam R, Uzir N, Raman S, Banerjee S. Experimenting XGBoost Algorithm for Prediction and Classification of Different Datasets. In: National Conference on Recent Innovations in Software Engineering and Computer Technologies (NCRISECT) 2017; 2017. .
128. Kingma DP, Ba J. Adam: A Method for Stochastic Optimization. arXiv:14126980 [cs] 2017 Jan;.
129. Little RJA, Rubin DB. Statistical Analysis with Missing Data. 3rd edition ed. Wiley Series in Probability and Statistics, Hoboken, NJ: Wiley; 2020.
130. Bennett DA. How Can I Deal with Missing Data in My Study? Australian and New Zealand Journal of Public Health 2001 Oct;25(5):464–469.
131. Jerez JM, Molina I, García-Laencina PJ, Alba E, Ribelles N, Martín M, et al. Missing Data Imputation Using Statistical and Machine Learning Methods in a Real Breast Cancer Problem. Artificial Intelligence in Medicine 2010 Oct;50(2):105–115.
132. Khan SI, Hoque ASML. SICE: An Improved Missing Data Imputation Technique. Journal of Big Data 2020 Dec;7(1):37.
133. Lin WC, Tsai CF. Missing Value Imputation: A Review and Analysis of the Literature (2006–2017). Artificial Intelligence Review 2020 Feb;53(2):1487–1509.
134. Rubin LH, Witkiewitz K, Andre JS, Reilly S. Methods for Handling Missing Data in the Behavioral Neurosciences: Don't Throw the Baby Rat out with the Bath Water. Journal of undergraduate neuroscience education: JUNE: a publication of FUN, Faculty for Undergraduate Neuroscience 2007;5(2):A71–77.
135. Delalleau O, Courville A, Bengio Y. Efficient EM Training of Gaussian Mixtures with Missing Data. arXiv:12090521 [cs, stat] 2018 Jan;.
136. Andridge RR, Little RJA. A Review of Hot Deck Imputation for Survey Non-response. International Statistical Review 2010 Apr;78(1):40–64.
137. Cheema JR. A Review of Missing Data Handling Methods in Education Research. Review of Educational Research 2014 Dec;84(4):487–508.
138. Jonsson P, Wohlin C. An Evaluation of K-Nearest Neighbour Imputation Using Likert Data. In: 10th International Symposium on Software Metrics, 2004. Proceedings. Chicago, IL, USA: IEEE; 2004. p. 108–118.
139. Maillo J, Ramírez S, Triguero I, Herrera F. kNN-IS: An Iterative Spark-based Design of the k-Nearest Neighbors Classifier for Big Data. Knowledge-Based Systems 2017 Feb;117:3–15.
140. Amirteimoori A, Kordrostami S. A Euclidean Distance-Based Measure of Efficiency in Data Envelopment Analysis. Optimization 2010 Oct;59(7):985–996.
141. Beretta L, Santaniello A. Nearest Neighbor Imputation Algorithms: A Critical Evaluation. BMC Medical Informatics and Decision Making 2016 Jul;16(S3):74.
142. Acuña E, Rodriguez C. The Treatment of Missing Values and Its Effect on Classifier Accuracy. In: Banks D, McMorris FR, Arabie P, Gaul W, editors. Classification, Clustering, and Data Mining Applications Berlin, Heidelberg: Springer Berlin Heidelberg; 2004.p. 639–647.
143. Lee JY, Styczynski MP. NS-kNN: A Modified k-Nearest Neighbors Approach for Imputing Metabolomics Data. Metabolomics 2018 Dec;14(12):153.
144. Sun B, Ma L, Cheng W, Wen W, Goswami P, Bai G. An Improved K-Nearest Neighbours Method for Traffic Time Series Imputation. In: 2017 Chinese Automation Congress (CAC) Jinan: IEEE; 2017. p. 7346–7351.
145. Cheng D, Zhang S, Deng Z, Zhu Y, Zong M. kNN Algorithm with Data-Driven k Value. In: Luo X, Yu JX, Li Z, editors. Advanced Data Mining and Applications Lecture Notes in Computer Science, Cham: Springer International Publishing; 2014. p. 499–512.
146. Murti DMP, Pujianto U, Wibawa AP, Akbar MI. K-Nearest Neighbor (K-NN) Based Missing Data Imputation. In: 2019 5th International Conference on Science in Information Technology (ICSITech) Yogyakarta, Indonesia: IEEE; 2019. p. 83–88.
147. Huang J, Keung JW, Sarro F, Li YF, Yu YT, Chan WK, et al. Cross-Validation Based K Nearest Neighbor Imputation for Software Quality Datasets: An Empirical Study. Journal of Systems and Software 2017 Oct;132:226–252.
148. Zhu M, Xingbing Cheng. Iterative KNN Imputation Based on GRA for Missing Values in TPLMS. In: 2015 4th International Conference on Computer Science and Network Technology (ICCSNT) Harbin, China: IEEE; 2015. p. 94–99.
149. Zhang S, Li X, Zong M, Zhu X, Cheng D. Learning *k* for kNN Classification. ACM Transactions on Intelligent Systems and Technology 2017 Apr;8(3):1–19.
150. Samek W, Wiegand T, Müller KR. Explainable Artificial Intelligence: Understanding, Visualizing and Interpreting Deep Learning Models. arXiv:170808296 [cs, stat] 2017 Aug;.
151. Caruana R, Lou Y, Gehrke J, Koch P, Sturm M, Elhadad N. Intelligible Models for HealthCare: Predicting Pneumonia Risk and Hospital 30-Day Readmission. In: Proceedings of the 21th ACM SIGKDD International Conference on Knowledge Discovery and Data Mining Sydney NSW Australia: ACM; 2015. p. 1721–1730.
152. Lapuschkin S, Binder A, Montavon G, Muller KR, Samek W. Analyzing Classifiers: Fisher Vectors and Deep Neural Networks. In: 2016 IEEE Conference on Computer Vision and Pattern Recognition (CVPR) Las Vegas, NV, USA: IEEE; 2016. p. 2912–2920.
153. Arras L, Horn F, Montavon G, Müller KR, Samek W. Explaining Predictions of Non-Linear Classifiers in NLP. arXiv:160607298 [cs, stat] 2016 Jun;.
154. Arras L, Horn F, Montavon G, Müller KR, Samek W. "What Is Relevant in a Text Document?": An Interpretable Machine Learning Approach. PLOS ONE 2017 Aug;12(8):e0181142.
155. Schütt KT, Arbabzadah F, Chmiela S, Müller KR, Tkatchenko A. Quantum-Chemical Insights from Deep Tensor Neural Networks. Nature Communications 2017 Apr;8(1):13890.
156. Sturm I, Lapuschkin S, Samek W, Müller KR. Interpretable Deep Neural Networks for Single-Trial EEG Classification.

Journal of Neuroscience Methods 2016 Dec;274:141–145.
157. Lipovetsky S, Conklin M. Analysis of Regression in Game Theory Approach. Applied Stochastic Models in Business and Industry 2001 Oct;17(4):319–330.

Manuscript file Click here to access/download;Manuscript;Manuscript.pdf

OXFORD (GIGA)^n SCIENCE

GigaScience, 2022, 1–18

doi: xx.xxxx/xxxx
Manuscript in Preparation
Research

RESEARCH

Disease classification for whole blood DNA methylation: meta-analysis, missing values imputation, and XAI

Alena Kalyakulina[1,*,†], Igor Yusipov[1,†], Maria Giulia Bacalini[2], Claudio Franceschi[1], Maria Vedunova[3] and Mikhail Ivanchenko[1]

[1]Institute of Information Technologies, Mathematics and Mechanics, Lobachevsky State University, Nizhny Novgorod, Russia and [2]IRCCS Istituto delle Scienze Neurologiche di Bologna, Bologna, Italy and [3]Institute of Biology and Biomedicine, Lobachevsky State University, Nizhny Novgorod, Russia

[*]kalyakulina.alena@gmail.com
[†]Contributed equally

Abstract

Background: DNA methylation has a significant effect on gene expression and can be associated with various diseases. Meta-analysis of available DNA methylation datasets requires development of a specific workflow for joint data processing.
Results: We propose a comprehensive approach of combined DNA methylation datasets to classify controls and patients. The solution includes data harmonization, construction of machine learning classification models, dimensionality reduction of models, imputation of missing values, and explanation of model predictions by explainable artificial intelligence (XAI) algorithms. We show that harmonization can improve classification accuracy by up to 20% when preprocessing methods of the training and test datasets are different. The best accuracy results were obtained with tree ensembles, reaching above 95% for Parkinson's disease. Dimensionality reduction can substantially decrease the number of features, without detriment to the classification accuracy. The best imputation methods achieve almost the same classification accuracy for data with missing values as for the original data. XAI approaches have allowed us to explain model predictions from both populational and individual perspectives.
Conclusions: We propose a methodologically valid and comprehensive approach to the classification of healthy individuals and patients with various diseases based on whole blood DNA methylation data using Parkinson's disease and schizophrenia as examples. The proposed algorithm works better for the former pathology, characterized by a complex set of symptoms. It allows to solve data harmonization problems for meta-analysis of many different datasets, impute missing values, and build classification models of small dimensionality.

Key words: DNA methylation, machine learning, data harmonization, explainable artificial intelligence

Introduction

Background

DNA methylation (DNAm) plays an important role in human development and is associated with gene expression, genomic imprinting, and other biological processes without altering the DNA sequence [1, 2, 3, 4, 5, 6, 7, 8]. Abnormal methylation patterns can lead to numerous diseases [9]. DNA methylation consists of binding a methyl group to cytosine in the cytosine-guanine dinucleotides (CpG sites). Hypermethylation of CpG sites near the gene promoter is known to repress transcription, while hypermethylation in the gene body appears to have an opposite, also less pronounced effect [10, 11]. Changes in DNAm patterns are associated with aging and environmental exposures [12, 13]. Current epigenome-wide association studies (EWAS) test DNAm associations with human phenotypes, health conditions and diseases [14, 15, 16]. Microarray-based technologies, such as the Illumina HumanMethylation450 (450K) and HumanMethylationEPIC (850K) arrays [17] are based on the hybridization of bisulfite-converted DNA to 50-mer probes

Compiled on: August 1, 2022.
Draft manuscript prepared by the author.

and for each CpG site included in the design allow to estimate the fraction of methylated DNA copies. Two metrics are used to represent methylation levels: the β-value, ranging from 0 to 1, and the M-value, the log2 ratio of the intensities of methylated versus unmethylated probes [18, 19, 20]. M-values are more robust quantifiers since β-values close to 0 and 1 suffer from substantial heteroscedasticity [18].

Nowadays, machine learning has become a broadly applicable method for data modeling and analysis in a wide range of applications. The availability of large data sets and a variety of unreinforced generative methods make these approaches more accurate, simple, and relevant in bioinformatics, in particular, for transcriptomic and epigenetic data analysis [21, 22, 23, 24, 25, 26]. DNA methylation data are often used for classification tasks. One of the most common examples is the classification of different types of cancer using the TCGA repository [27]. Such classifiers usually demonstrate high accuracy [26, 28, 29, 30, 31, 32, 33, 34], based on both cancer-induced changes in methylation and the differences in methylation of various tumor tissues [28, 35]. Classifying different human conditions - phenotypes or pathologies - using DNA methylation data from a single tissue is more difficult. Phenotype classification can question smoking or obesity status, although existing results suggest that such conditions may not be clearly reflected in DNA methylation [26, 36, 37]. Classification of cases and controls for certain diseases is also performed using DNA methylation data. Examples of machine learning applications using epigenetic data include classification of coronary heart disease, neurodevelopmental syndromes, schizophrenia, Alzheimer's disease, psychiatric disorders and others [38, 39, 40, 41, 42, 43, 44].

One of the main challenges is that methylation datasets are limited in the number of samples. Increasing the amount of data requires combining many datasets collected under different conditions and then performing analysis for the merged data, which can cause a variety of problems. There are many factors that lead to significant differences in methylation data that are not directly related to the development of pathological conditions, to name the effect of the laboratory batch, different experimental conditions, normalization, and other [45].

Methylation levels can be affected by systematic variation due to biosample processing, i.e., batch-related variability (a subset of samples processed simultaneously), chip position in batches, and sample position within the chip [46, 47]. Batch effects can dramatically reduce the accuracy of measurements and produce false positive effects if the sample distribution is not uniform [48]. Most of the existing works avoid the question of the applicability of obtained models to new data. A central issue of meta-analysis is data harmonization. Ref. [49] developed an approach to systematically assess the impact of different preprocessing methods on meta-analysis. Its main advantage is the possibility of harmonization of the newly introduced datasets that does not require corrections to the previously analyzed datasets, employed for training the machine learning model.

Making use of new datasets to validate the model raises another problem, that is missing values in the data and the need to fill them in. New (test) datasets can lack information about some relatively small number of CpG sites on which the model was built. Experimental methylation data often contain missing values due to failing quality control checks, which can affect subsequent analysis. Since such missing CpG sites are necessary input parameters for the model, their values must be imputed. Examples include epigenetic clocks, which estimate biological age from small sets of pre-selected age-correlated CpG sites [5, 50, 51, 52], sensitive to small deviations in methylation levels [53]. Consequently, accurate imputation of missing data is required to improve the quality of DNA methylation analysis [54].

Dimensionality presents yet another problem. High data dimensionality is often associated with various undesirable consequences: increased computational effort, retraining, and visualization difficulties [55]. High-dimensional data may contain redundant information and introduce noise, while low-dimensional data may be sufficient for comprehensive data characterization. Since methylation data is multivariate, continuous, with nonlinear dependencies, traditional approaches often encounter the problem of multiple hypothesis testing and multicollinearity [26]. In addition, the most common epigenetic models [5, 51, 56, 57] contain a small number of variables to simplify data processing, for better interpretation of the results and for the possibility of applying these models in real life. It is also worth noting that small DNA methylation panels are significantly less costly [58], which is an undeniable advantage for the possibility of widespread use.

Modern artificial intelligence systems based on machine learning are powerful and promising tools in a wide range of applications from computer vision, machine translation and speech recognition [59, 60, 61] to the analysis of biomedical data, in particular DNA methylation [26, 62, 63]. However, while these models provide impressive predictive accuracy, their nonlinear structure makes them poorly interpretable, i.e. it is hard to explain what information in the input data leads AI to particular outputs. The need for trustworthy solutions has recently attracted much attention to methods that would "open" black box models [64, 65, 66, 67, 68, 69, 70, 71, 72, 73, 74]. This includes developing methods to help better understand what the model has learned [75, 76] as well as methods to explain individual predictions [65, 66, 67, 77, 78].

In summary, individual DNA methylation datasets contain an insufficient number of samples to apply machine learning approaches, so there is a need to combine and harmonize different datasets. Problems that arise on the way include tackling batch effects in individual datasets, missing values for certain samples, and high data dimensionality. Here, we analyze several existing fragmented solutions to these problems, develop a generalized unifying approach integrated in a workflow, validate it and demonstrate its efficiency.

Study design and novelty

Our primary goal is to offer a methodologically complete workflow for building machine learning models, classifying cases and controls for various diseases from whole blood DNA methylation data on many datasets, ranging from data harmonization to explainable artificial intelligence models. DNA methylation data are taken from different human body tissues, but the most widespread is whole blood methylation, the least invasive analysis and, therefore, of broad diagnostic prospects. We restrict our analysis to this kind of data. Our workflow solves a problem of harmonization of methylation data from different datasets. They are collected in different laboratories, with different setups and experimental conditions. In general, the data are of different quality, and have been preprocessed differently. Harmonization is used to eliminate the unavoidable bias between the data and to minimize the associated machine learning model errors. The proposed workflow uses harmonization with the selection of a reference dataset, in which case all other datasets are aligned with the reference one, so that when a new dataset is introduced, there is no need to renormalize the training data and hence rebuild the model. The workflow uses the generally recognized types of machine learning models for classification on methylation data in tabular representation, in particular gradient-boosted decision trees. A hyperparametric search for the optimal combination of the parameters of these models is performed to ensure the best classification accuracy. Next, the dimensionality of the feature space is reduced to build portable models. In such models, the number of features has the same order as the most popular epigenetic models, such as the Horvath clock (353 CpG sites) [5], Hannum clock (71 CpG sites) [51], DNAm PhenoAge (513 CpG sites) [56], DNAm GrimAge (1030 unique CpGs were used to predict plasma protein levels) [57]. Such portable models allow them to be

used for early diagnosis of various diseases - analysis of small CpG panels is much cheaper than full-genome analyses. Reducing the dimensionality of the data can also help discard noisy features that do not carry relevant information for classifiers. Also, the proposed approach includes the possibility of imputing missing values (CpG sites), and different approaches are used for this purpose. This is especially important when testing the model on new data, where some CpG sites critical for the model may be missed (e.g., because of technical errors in data acquisition and processing or failing quality checks). For the best models in terms of accuracy, explainable artificial intelligence (XAI) methods are applied to explore both the global influence of individual CpG sites on model predictions and to get explanations of how the methylation level values of individual CpG sites for specific subjects shape their individual predictions. Lists of the most important CpG sites in terms of machine learning models are compared with lists of CpG sites (and their corresponding genes) from existing studies associated with the considered diseases. Biological pathways of diseases based on these lists are identified and investigated.

Results

Larger training sample sizes provide better quality of machine learning models. The currently available DNA methylation data sets do not exceed several thousand samples, and that could hardly change in the near future due to complexity and cost of study. Merging different data sets, therefore, appears a practical way to circumvent size limitations. However, it poses many challenges, such as the need to harmonize datasets collected under different conditions and pre-processed in different ways, the need to fill in missing values in a way that preserves patterns in the data, the reduction of excessively high dimensionality of input variables with a relatively small number of samples. These issues have been addressed separately; below we report an integrated solution that brings together the data processing and analysis steps and the resulting methodologically complete workflow for solving the classification problem based on merging several independent DNA methylation data. A schematic representation of the proposed workflow is shown in Figure 1.

Datasets and machine learning tasks

We studied whole blood DNA methylation datasets generated on subjects with Parkinson's disease or schizophrenia. We selected 3 datasets that contain samples from subjects with Parkinson's disease and healthy controls: GSE145361 [79], GSE111629 [80, 81, 82], GSE72774 [80, 81, 83] and 4 datasets that contain samples from subjects with schizophrenia and healthy controls: GSE152027 [84], GSE84727 [84, 85], GSE80417 [84, 85], GSE116379 (non-famine participants) [86]. Information about considered datasets is summarized in Table 1, in particular, the number of cases and controls, whether the dataset has been used as train or test, the original preprocessing type, the number of CpGs.

For each disease, we built machine learning models to classify cases vs. controls. Some of these datasets are used as train data for building the model, and the rest is used to test the model. For each disease we selected a reference dataset, against which harmonization was performed. As it can be seen from Table 1, the original preprocessing is the same for the majority of the considered datasets with schizophrenia patients, but it varies considerably among the different datasets for Parkinson's disease. To reduce the influence of the laboratory-specific data collection and processing conditions on classification results, harmonization is necessary.

Meta-analysis and harmonization

Combining different DNA methylation datasets can improve the statistical power to test hypotheses and identify epigenetic signatures by meta-analysis. However, such meta-analysis also poses serious problems related to data harmonization, which is often not considered [87]. This is especially true for DNA methylation, where data is often only available in the preprocessed rather than raw form, and where diverse preprocessing pipelines are used [49]. Developed in [49] approach regRCPqn (regional regression on correlated probes with quantile normalization) allows for meta-analysis even if the raw data are not available. Importantly, as emerging datasets are aligned, the already treated datasets do not require renormalization. Therefore, we apply this approach to harmonization with reference. The largest dataset for each disease is taken as the reference, and other datasets are harmonized relative to it. The schematic representation of the harmonization process is shown in Figure 2.

For machine learning models, we used only those CpG sites that have the same distribution of methylation levels in different train datasets in the control group (methylation levels in the case group typically have greater variability because of disease heterogeneity). We used the Mann-Whitney U-test [88] to compare DNA methylation values of healthy participants from the considered train datasets before and after harmonization. After harmonization, the number of CpG sites with the adjusted p-value >0.05 (not significantly different between healthy subjects from the considered train datasets) increased from 43019 to 50911 for Parkinson's disease and from 35145 to 110137 for schizophrenia. Figure 3 illustrates the change in the distributions of methylation level values before and after harmonization. In particular, CpG sites whose methylation level distributions differed significantly before harmonization (FDR-corrected p-values<0.05) manifest similar distributions after harmonization (FDR-corrected p-values>0.05).

Classification models

The most common type of data representation for machine learning is tabular, and DNA methylation data fulfills it. Typically, the rows refer to participants, the columns refer to CpG sites, and the cells of the table contain the methylation levels of each CpG site for each participant. There are many machine learning models designed to work with tabular data: Logistic Regression with elastic net penalty [89], Support-Vector Machine [90], XGBoost [91], CatBoost [92], LightGBM [93], TabNet [94], NODE [95]. Main characteristics of the models are summarized in Table 2.

For each disease, all considered datasets were divided into training and test ones (as stated in Table 1). We trained all models on two training datasets and then tested on the remaining datasets. Accuracy with weighted averaging was the main quality metric, as it can handle situations with possible imbalance of the classes (the number of participants in different classes varies significantly). As discussed in the above, the approach fulfills the requirement that the model does not have to be trained again as the new data set is considered. Moreover, the models must be trained to classify biological differences in methylation data rather than traces of different experimental conditions in different laboratories. Accordingly, we do not mix train and test datasets and do not perform cross-validation. To find the optimal combination of model parameters that provides the best accuracy, we used a hyperparametric grid search (the values are presented in Supplementary Table S1).

Newly introduced datasets may lack some CpG sites that are present in already trained models; in this case various imputation methods are applied (cf. Sections Imputation of missing values in Results and Methods for more details). Models for Parkinson's disease for non-harmonized data are trained on 43019 CpG sites, for harmonized data on 50911 CpG sites. Among these, the Parkinson's disease test dataset GSE72774 lacks 38 CpG sites in the non-

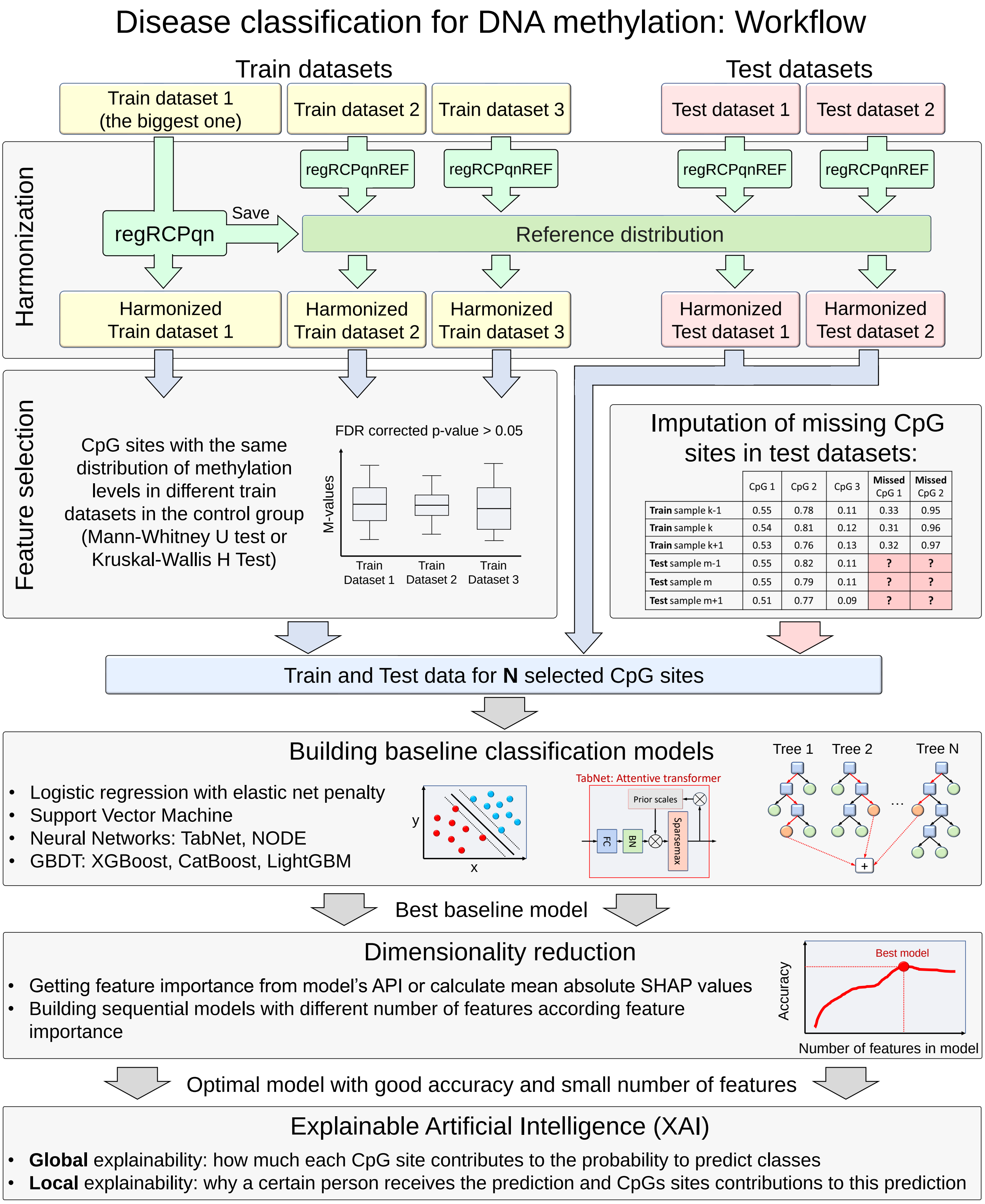

Figure 1. Workflow for classifying cases and controls of various diseases based on DNA methylation data proposed in this paper.

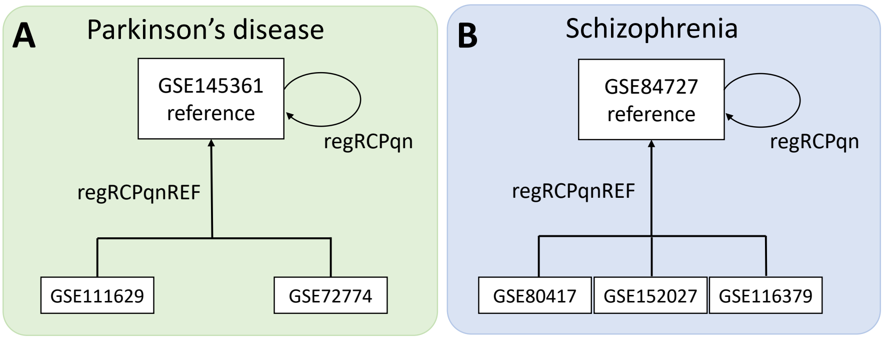

Figure 2. Schematic representation of harmonization procedure for (A) Parkinson's disease and (B) Schizophrenia datasets.

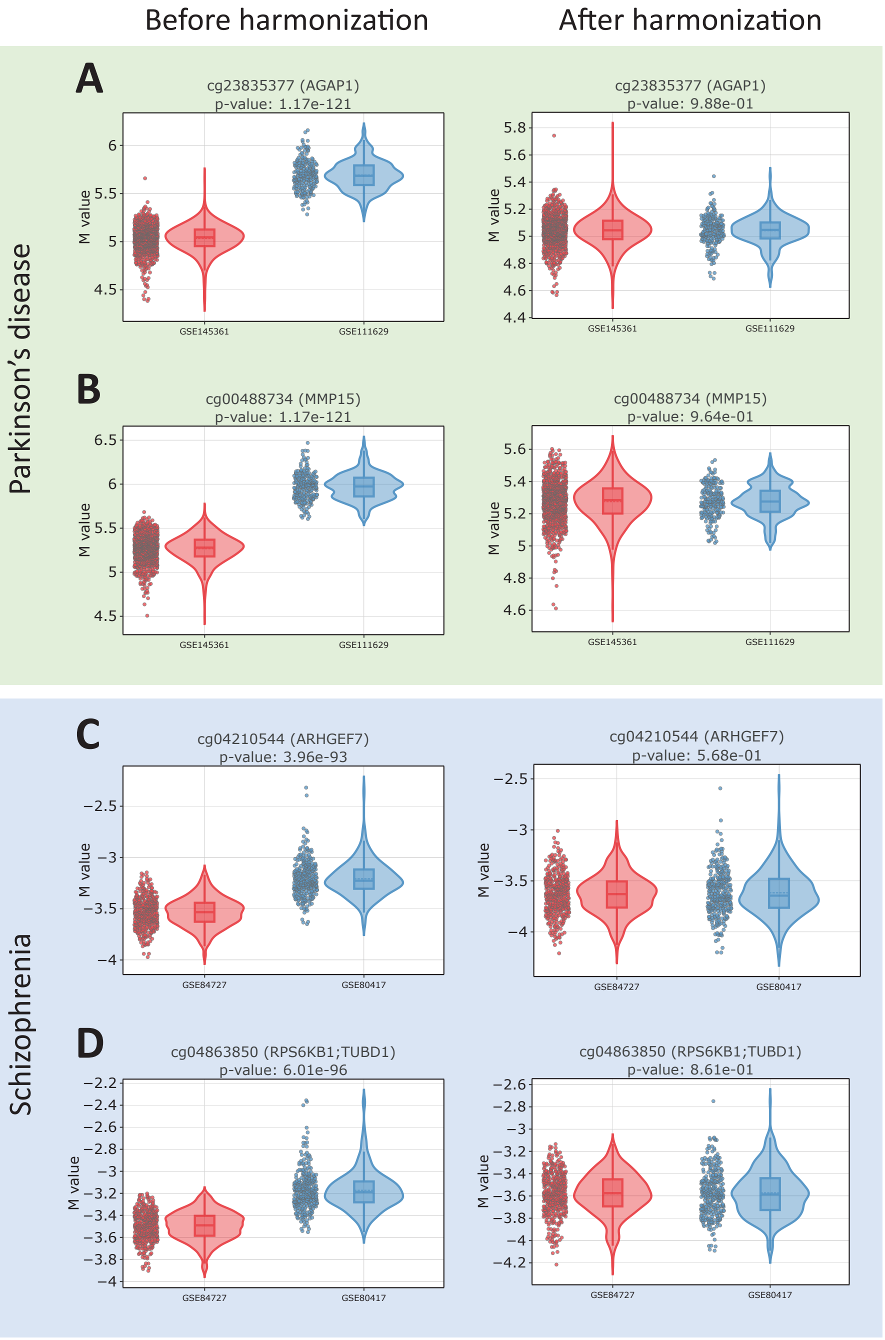

Figure 3. Examples of M-values methylation levels distribution for control groups before and after harmonization for Parkinson's disease examples (A) cg23835377 and (B) cg00488734, Schizophrenia examples (C) cg04210544 and (D) cg04863850.

Table 1. Main characteristics of considered datasets. For each disease, the bold row represents the reference dataset for the harmonization. Number of CpGs is common for train datasets in each disease. Three largest datasets for schizophrenia, GSE84727, GSE80417 and GSE152024, have the same preprocessing.

Disease	Dataset	Number of cases	Number of controls	Train or Test subset	Raw IDAT available?	Number of CpGs	Original preprocessing
Parkinson's disease	**GSE145361**	**959**	**930**	**Train**	**Yes**	**411761**	**Data processing: Genome Studio software**
	GSE111629	334	237	Train	Yes		Data processing: R software v3.4.2<br>Functional normalization: minfi R package
	GSE72774	289	219	Test	No	411979	Data processing: BeadStudio software v3.2
Schizophrenia	**GSE84727**	**414**	**433**	**Train**	**No**	**399625**	**Importing: methylumi R package<br>methylumIDAT function<br>Preprocessing: watermelon R package<br>pfilter and dasen functions**
	GSE80417	353	322	Train	No		Importing: methylumi R package<br>methylumIDAT function<br>Preprocessing: watermelon R package<br>pfilter and dasen functions
	GSE152027	290	203	Test	No	411901	Importing: methylumi R package<br>methylumIDAT function<br>Preprocessing: watermelon R package<br>pfilter and dasen functions
	GSE116379	51	54	Test	No	407781	Removed: X and Y chromosome,<br>non-specific binding probes,<br>failed probes based on a detection p-value<br>> 0.001 and bead count < 5, probes with<br>SNPs of Minor Allele Frequency > 5% within<br>10 base pairs of the primer<br>Functional normalization: minfi R package

Table 2. Main characteristics of the considered classification models.

Model	Type	Feature importance API
Logistic Regression	Generalized linear model	Yes
Support-Vector Machine	Supervised learning model constructing the separating manifold	Only for linear kernel
XGBoost	Gradient-boosted decision tree ensemble	Yes
CatBoost	Gradient-boosted decision tree ensemble	Yes
LightGBM	Gradient-boosted decision tree ensemble	Yes
TabNet	Deep neural network	Yes
NODE	Gradient-boosted decision tree ensemble	No

harmonized data and 34 CpG sites in the harmonized data. Models for schizophrenia for non-harmonized data train on 35145 CpG sites, for harmonized data train on 110137 CpG sites. The first test dataset for schizophrenia GSE152027 lacks 9 CpG sites in the non-harmonized data and 36 CpG sites in the harmonized data. The second test dataset for schizophrenia GSE116379 lacks 268 CpG sites in the non-harmonized data and 609 CpG sites in the harmonized data. These missed CpG sites are imputed using KNN methods with K=1. However, this imputation does not have a significant effect on the result, because, as will be shown later, all the missed CpG sites are not at the top of the features in terms of importance.

Table 3 shows the results of cases vs. controls classification by baseline models based on non-harmonized and harmonized whole blood methylation data for Parkinson's disease and schizophrenia on test datasets. For each combination of harmonization type, disease, and test dataset, the best weighted accuracy values for all constructed models is given. All imputation methods described in Section Imputation of missing values do not significantly change the quality of the resulting models, because all missed CpGs in test datasets have a very low value of feature importance in models with corresponding API.

The results confirm that harmonization must be applied and is most efficient for the datasets with different preprocessing methods. In particular, for Parkinson's disease, all datasets have different original preprocessing, and the best model trained on such data shows a result of 76%. When these data are harmonized, accuracy improves dramatically to 97%. For both non-harmonized and harmonized data, for Parkinson's disease, the best model in terms of weighted accuracy is LightGBM. For schizophrenia, only one of 4 datasets has a different preprocessing (GSE116379, Table 1). Then, harmonization does not significantly affect the quality of the built models if the datasets have the same preprocessing (68% without harmonization, 72% with harmonization in the best models). The best model is LightGBM for non-harmonized data and CatBoost for harmonized data. However, applying models trained on non-harmonized data to data with a different preprocessing gives a poor result for binary classification - 59%. Harmonization of data improves the performance of the trained models, making them close to the best results obtained for schizophrenia in terms of quality - 71%. It is also worth noting that the overall classification quality for

Table 3. Binary classification results of baseline models for non-harmonized and harmonized data. For Parkinson's disease (green background) and schizophrenia (blue background), results comparing the accuracy of different models for non-harmonized and harmonized methylation data are shown.

Model	Parkinson's disease		Schizophrenia			
	GSE72774		GSE152027		GSE116379	
	Non-harmonized	Harmonized	Non-harmonized	Harmonized	Non-harmonized	Harmonized
Logistic Regression	0.71	0.93	0.63	0.66	0.56	0.66
Support-Vector Machine	0.67	0.92	0.62	0.66	0.58	0.65
XGBoost	0.72	0.95	0.67	0.71	0.56	0.66
CatBoost	0.71	0.94	0.68	0.72	0.59	0.71
LightGBM	0.76	0.97	0.68	0.71	0.58	0.67
TabNet	0.69	0.93	0.63	0.66	0.58	0.65
NODE	0.71	0.92	0.62	0.66	0.56	0.65

these two diseases on methylation data is very different, possibly due to the different etiology and molecular mechanisms involved in the two diseases.

Best accuracy models allow us to extract importance values for all features. The ranking of the most important features for these models for Parkinson's disease and schizophrenia is shown in Figure 4. It is worth noting that for schizophrenia, there is one outstanding CpG with the highest importance for classification, while the others have much lower values. For Parkinson's disease, the situation is more uniform. These rankings can be used for the dimensionality reduction of the built models.

Dimensionality reduction

As a result of applying different baseline models to methylation data to classify cases vs controls, the ones with an API for feature extraction showed the best accuracy. Based on the obtained ranking of the features (Figure 4), we performed dimensionality reduction of the constructed models. Most common epigenetic models comprise few CpG sites, no more than several hundred (for example, those used to predict epigenetic age like Horvath' clocks and Hannum clocks). First, models based on few features show significantly better performance while maintaining similar classification accuracy. Second, such models are less memory-consuming.

Along these lines, we reduced the dimensionality of the model, leaving only the most important features for classification. Figure 4 shows the dependence of weighted classification accuracy on the number of features in the model for Parkinson's disease and for schizophrenia. It first increases, until reaching a certain optimal number of features, and then changes weakly. For Parkinson's disease, the best weighted accuracy of 96% is observed for 890 CpG sites, with an accuracy value changed by only 1% compared to the full data (50911 CpG sites). For schizophrenia, the best weighted accuracy of 75% is observed for 670 CpG sites for the test dataset GSE152027 and 70% for the test dataset GSE116379. The optimal model for schizophrenia was chosen as the one for the GSE152027. The accuracy values changed by no more than 3% compared to the full data (110137 CpG sites). The list of CpG sites that make up these small models, as well as basic information about them (gene, chromosome, relation to the CpG island) is presented in Supplementary Table S2. The resulting CpG lists were compared with previously published lists of biomarkers associated with Parkinson's disease [80, 96, 97] and schizophrenia [84, 98]. Interestingly, for Parkinson's disease, there is practically no overlap with the previous results, except for one CpG site from [96]. This CpG belongs to the gene DYNC1H1, which is associated with neurological and neurodegenerative diseases [99, 100]. For schizophrenia, only 15 CpG sites are common with [84]. Some of the connected genes like PRKCZ, SHANK2, ZNF608, PRDM16 were also identified as schizophrenia risk factors [101, 102, 103, 104]. Genes, corresponding to CpG sites, from the optimal small model for Parkinson's disease were enriched in several gene ontologies related to neuronal and metabolic processes, whereas genes from schizophrenia models were enriched in gene ontologies related to cell development processes (Supplementary Table S3).

Imputation of missing values

For trained machine learning models (both large and small), it is important that there are no missing values in the upcoming test data. Since it is impossible to guarantee their absence, various imputation methods are used to fill them in. Since not all models support data imputation, we use the most popular of them: mean, median, mode, random, chained equations, expectation maximization, KNN with different numbers of neighbors (from 1 to 3). To study the effect of these imputation methods on the classification accuracy, we consider the following simulation experiment. For each disease, we consider only the best small models, obtained at the previous step (LightGBM with 890 CpG sites for Parkinson's disease and CatBoost with 670 CpG sites for schizophrenia). For these models we "remove" 100 CpG sites with the highest importance values and impute them. The number of CpG sites was chosen to induce a significant drop in accuracy and to sharpen the differences in efficiency between the imputation methods. The missing CpG sites do not take part in the construction of small optimal models, so the actually existing CpG sites are removed from consideration. Table 4 shows results for the considered test datasets. For Parkinson's disease, KNN with one neighbor kept the classification accuracy at the baseline level of the data without missing values. Imputation with mode also showed good results, losing only 3%. The other methods achieved an accuracy of no more than 90%. For schizophrenia, none of the approaches achieved the baseline accuracy for data without missing values. This may be explained by the critical importance of specific features for classification. KNN with one neighbor for both datasets shows one of the best imputation results, for GSE152027 chained equation and expectation maximization perform better than KNN by 3% and 2%, respectively. Median and random values methods show unsatisfactory results in all experiments.

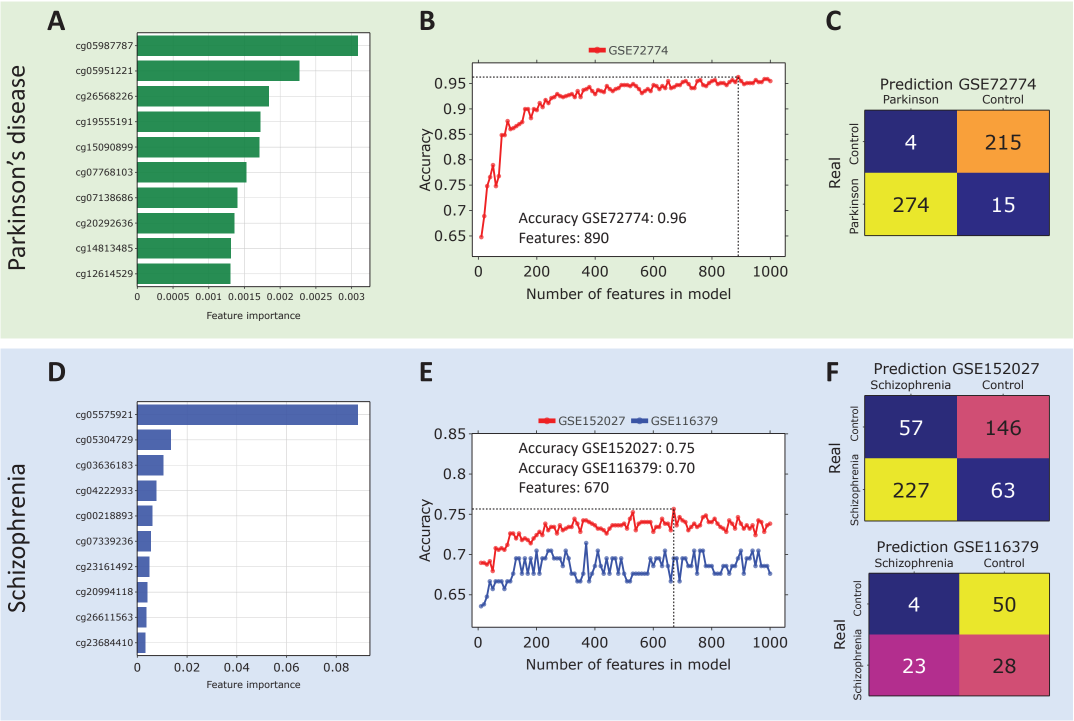

Figure 4. Dimensionality reduction for the best classification models. Parkinson's disease: (A) Top-10 features for the best classification model LightGBM with the normalized importance values. (B) Dependence of the weighted accuracy on the number of features in the model. Dotted line corresponds to the optimal small model with accuracy value 0.96 and 890 features. (C) Confusion matrix for optimal model. Schizophrenia: (D) Top-10 features for the best classification model CatBoost with the normalized importance values. (E) Dependence of the weighted accuracy on the number of features in the model. Dotted line corresponds to the optimal small model with accuracy value 0.75 for GSE152027, 0.70 for GSE116379 and 670 features. (F) Confusion matrix for optimal models.

Table 4. Comparison of different missing value imputation methods and their effect on weighted classification accuracy for Parkinson's disease (green background) and schizophrenia (blue background). In all cases, 100 CpG sites with the highest importance values were dropped.

Model	Parkinson's disease	Schizophrenia	
	GSE72774	GSE152027	GSE116379
**Original (no missed data)**	**0.96**	**0.75**	**0.7**
Mean	0.78	0.65	0.67
Median	0.81	0.59	0.55
Mode	0.93	0.55	0.54
Random	0.86	0.62	0.54
Chained equation	0.89	0.68	0.61
Expectation maximization	0.87	0.67	0.54
KNN (K = 1)	0.96	0.65	0.67
KNN (K = 2)	0.9	0.65	0.67
KNN (K = 3)	0.86	0.65	0.67

Explainable artificial intelligence

Even the most accurate machine learning models make mistakes on upcoming data. It presents a major challenge for those models that work as "black boxes" with unknown principles behind made decisions. SHapley Additive exPlanations (SHAP) help to understand why the model makes its predictions from the global and local points of view [105].

The global explainability of the constructed models on the training data for Parkinson's disease and schizophrenia is illustrated in Figure 5. Beeswarm plots show the relationship between SHAP values and methylation levels for the most important CpG sites. For each CpG site, the distributions of methylation levels for all participants are shown. In particular, for Parkinson's disease, most of the participants in the CpG site cg05987787 have low methylation levels, which positively affects the probability of predicting the disease. The scatter plots show in detail the distribution of methylation levels in different participants and SHAP values. We can see that M-values below 4 have a positive effect on the probability of predicting disease, while M-values above 4 have a negative effect. The black line divides the areas of positive and negative influence of SHAP values on the prediction of disease probability. The opposite situation is observed for the CpG site cg05951221. M-values below -1 have a negative effect on the probability of predicting disease, while M-values above -1 have a positive effect. Similar plots are shown for schizophrenia. The beeswarm plot shows that there is one most important CpG site, cg05575921, that contributes the most to the probability of predicting disease, as previously shown, and the other CpG sites have a much smaller effect.

The local explainability of predictions on the test data is shown in Figure 6. The top row presents heatmaps with participants on the x-axis, CpG sites on the y-axis, and SHAP values encoded on a color scale. The participants are ordered based on the probability to predict the disease. Model output is shown above the heatmap

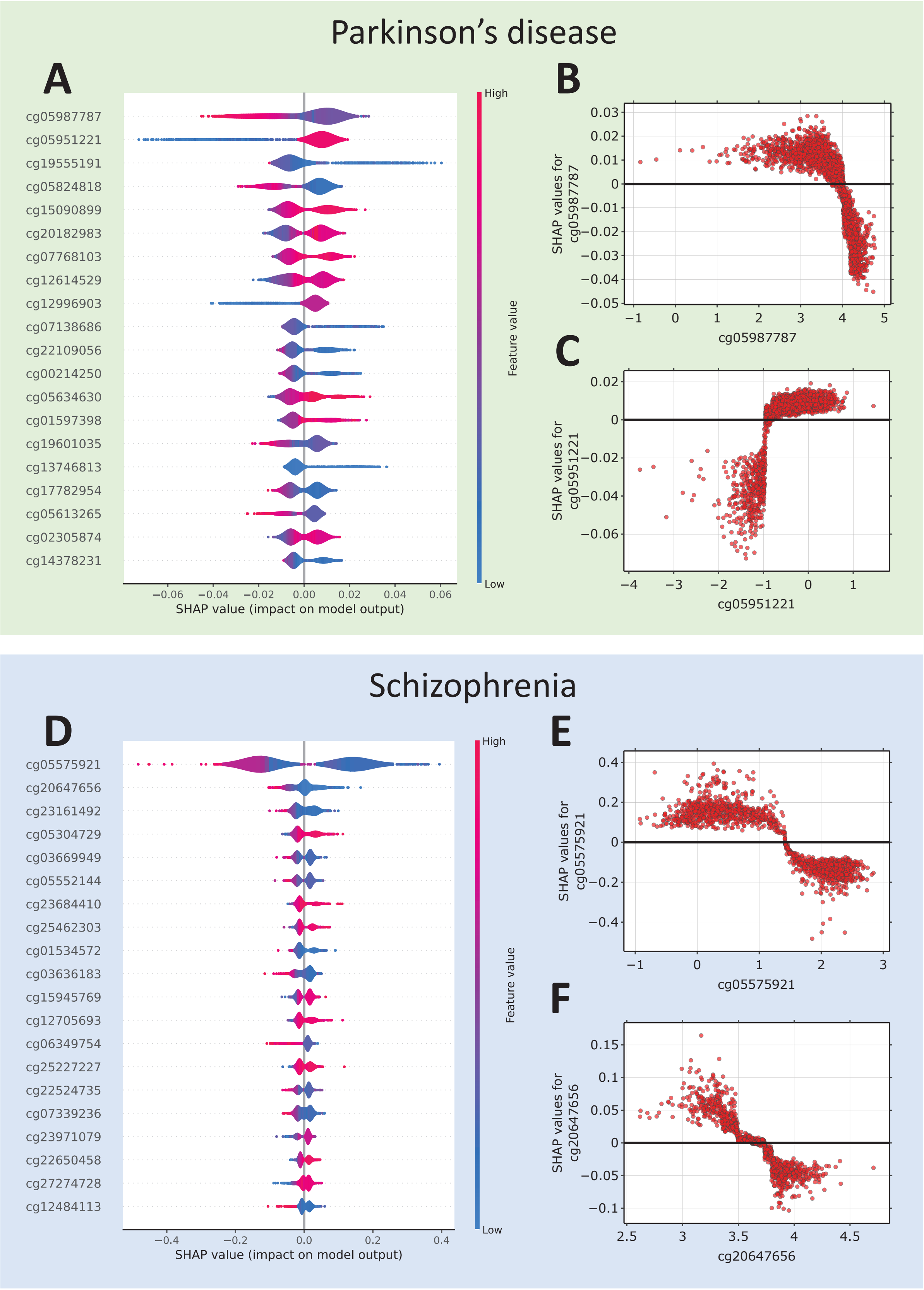

Figure 5. Global explainability based on SHAP values. Parkinson's disease: (A) Beeswarm plots show the dependence of SHAP values for each CpG site on their methylation levels. Each dot represents one participant. (B) Dependence of SHAP values on methylation M-values for cg05987787. The black line separates the areas of negative and positive influence of SHAP values on the probability of predicting Parkinson's disease. (C) Dependence of SHAP values on methylation M-values for cg05951221. Schizophrenia: (D) Beeswarm plots show the dependence of SHAP values for each CpG site on their methylation levels. Each dot represents one participant. (E) Dependence of SHAP values on methylation M-values for cg05575921. (F) Dependence of SHAP values on methylation M-values for cg20647656.

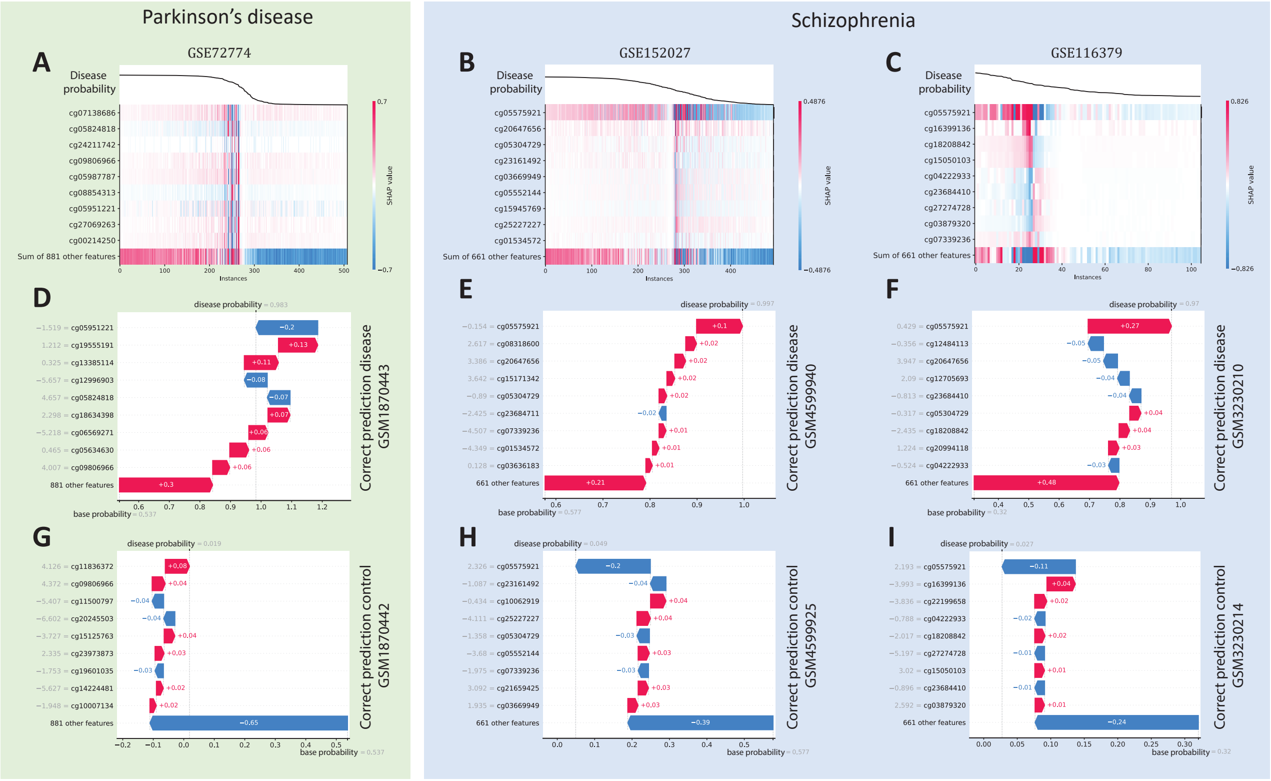

Figure 6. Local explainability based on SHAP values. (A) Black lines show the probability of predicting Parkinson's disease for different participants. Heatmaps in color show the contribution of SHAP values to the probability of predicting Parkinson's disease for different participants and CpG sites for GSE72774. (B) Probability of predicting schizophrenia and heatmaps of contribution for GSE152027. (C) Probability of predicting schizophrenia and heatmaps of contribution for GSE116379. (D) Waterfall plot for participant GSM1870443 with Parkinson's disease, showing the contribution of individual CpG sites to changes in the probability of predicting disease. (E) Waterfall plot for participant GSM4599940 with schizophrenia. (F) Waterfall plot for participant GSM3230210 with schizophrenia. In all cases, the models' confidence is over 97%. (G) Waterfall plot for control participant GSM1870442 showing the contribution of individual CpG sites to the change in probability of predicting disease. (H) Waterfall plot for control participant GSM4599925. (I) Waterfall plot for control participant GSM3230214. In all cases, the probability of predicting disease is below 5%.

matrix. The black line represents the probability of predicting the disease for each participant. It follows that for Parkinson's disease, where the model works with high accuracy, this line is quite smooth and similar to the softmax function. Whereas for schizophrenia, for which the models have much lower accuracy, these probability plots are more fragmented. As shown earlier, for schizophrenia, one CpG site is the most important, so it has the highest absolute SHAP values and appears the brightest in the heatmaps. The center and bottom lines represent waterfall plots for participants with the disease and controls, respectively. They allow for explaining the model output for each participant separately. The bottom part of the waterfall plot shows the base probability of the model to predict disease, and then each line shows how a positive (red) or negative (blue) contribution from each CpG site moves the probability to the model output for that prediction. The output of the model is the probability of predicting disease. If the probability is greater than 50%, the model identifies the participant as a case, otherwise it identifies the participant as a control. The baseline probability is the average probability of the model predicting on the test data. Because baseline probability is a characteristic of the model, it depends on the quality of the model. If the model has reasonably good accuracy, then the base probability is close to the proportion of participants in a particular class. In the examples from middle line of the Figure 6 for all participants with diseases, the probability of predicting disease in the examples was above 97% (models identify them as cases almost for sure); for control participants (bottom line of Figure 6), the probability of predicting disease was below 5% (models identify them as controls almost for sure).

Discussion

Conclusion

We developed a multifunctional workflow for applying machine learning models to classify cases and controls for different diseases based on DNA methylation data. Specifically, we considered Parkinson's disease and schizophrenia as examples of complex diseases requiring early diagnosis. In addition, for these diseases there are large publicly available whole blood DNA methylation datasets. In this paper, the task of classifying cases and controls based on whole blood DNA methylation data with harmonization, missing value imputation, model dimensionality reduction, and application of XAI approaches is solved for the first time for Parkinson's disease and schizophrenia. In [106] the problem of pairwise classification of neurodegenerative diseases cases (in particular, for Parkinson's disease and schizophrenia) was solved. The authors did not use methylation levels; instead, they used methylation-derived profile scores as features; however, we do not compare diseases with each other in this work. In [40] the problem of classifying cases and controls for schizophrenia is solved. In that work, instead of methylation levels, other metrics were also used: CoRSIV probes with a polygenic risk score. The paper presents the results of positive predictive values only for cases, not for controls, so it is not possible to directly compare the results.

The first step of the workflow is to harmonize the data according to the chosen reference dataset. We have shown that harmonization works well also when the available datasets were preprocessed using different pipelines and tools as it often occurs. Harmonization can increase the classification accuracy by up to 20%. This is fully

consistent with the original paper, which proposed the harmonization method regRCPqn [49]. When the preprocessing of training and test data is the same, harmonization has almost no effect on the final classification accuracy. It is impossible to guarantee that all new datasets on which the model will be tested will have the same preprocessing. Even with tools such as limma [107], ComBat [108], it may not be possible to remove technical signal when batches are mixed with variables of interest. Applying ComBat to high-throughput data with an uneven study design may actually result in false signals [109]. To solve the classification problem, different models were tested, classical ones (logistic regression and support-vector machine), different gradient-boosted decision trees and deep neural networks. For all models, a hyperparametric search was performed to select the optimal set of parameters. The best results of weighted accuracy were obtained with tree ensembles, which is consistent with other works [110]. For Parkinson's disease, the accuracy of classifying patients and healthy controls was higher than 95%. The accuracy for schizophrenia was much lower, only >70%. Unlike Parkinson's disease, schizophrenia is a complex disease characterized by a variety of different symptoms. A large number of different causes and molecular patterns determining the development of this pathology are being identified. The etiology of schizophrenia is multifactorial and reflects an interaction between genetic vulnerability and environmental influences. Environmental risk factors, such as complications of pregnancy and childbirth, childhood trauma, migration, social isolation, urban life, and substance abuse, either singly or in combination over time, affect the likelihood of an individual developing the disorder. A lot of genes have been previously identified as responsible for the risk of developing this pathology, so the personal molecular landscape is highly individualized and complicated by interactions between genes and the environment. These reasons may be related to the relatively low accuracy for schizophrenia in our study compared to Parkinson's disease [111, 112, 113]. Another reason that affects the result may be the number of samples in the training set. The training set for schizophrenia has fewer samples compared to Parkinson's disease. In this case, the data may not be representative enough to fully describe the disease characteristics for the model. Since the best models allow us to obtain the importance values of each feature for classification, we constructed a CpG site ranking for each of the diseases. Based on this ranking, we reduced the dimensionality of the classification models, since many of the most common epigenetic models (such as the epigenetic clocks) contain relatively few CpG sites (up to 1000). To find optimal small models, we performed a series of experiments for an increasing number of features from 10 to 1000. Based on the dependence of the weighted classification accuracy on the number of features, we determined the optimal models, with the accuracy for both diseases changing not much, while the number of features decreased significantly. Optimal small models contain 890 CpG sites for Parkinson's disease and 670 CpG sites for Schizophrenia. Such small models are much less memory-consuming and have better computational performance. For machine learning models (both small and large), missing values are critical. A model trained on a particular set of features must necessarily be tested on the same set. If, for certain reasons (data collection or processing errors, low signal intensities, or other problems), some CpG sites are missing from the new data for testing, imputation methods are applied. The best imputation methods recover almost the same classification accuracy as for the original data. Since machine learning models work as a "black box", special methods must be used to explain exactly how the model makes predictions. Otherwise, the model cannot be trusted and it is impossible to identify the nature of errors. If the predictive capabilities of the model can be explained, it can help to discover complex relationships between biomarkers. The calculation of SHAP values allowed us to obtain both global and local explainability. Globally, the prediction of healthy control or patient with Parkinson's disease is affected by several CpG sites, there are examples of both types of influence, positive and negative. Global explainability for healthy controls and patients with schizophrenia confirmed the strongest influence of one CpG site, cg05575921, while the others had little effect on model prediction. This CpG site has previously been reported to be associated with schizophrenia and post-traumatic stress disorder (PTSD) [85, 114]. Examples of local explainability - specific predictions for certain participants - are also given.

Thus, we propose a methodologically valid and complete approach for classifying healthy people and patients with various diseases, which allows to harmonize DNA methylation data from different sources, impute missing values, reduce dimensionality of the models, and apply explainable artificial intelligence approaches. The proposed algorithm works better for Parkinson's disease than for schizophrenia, which is characterized by a variety of different symptoms. Further work may include expanding the pool of considered diseases, enriching the library of methods at different stages of the workflow. Another future challenge is advancing from explainability to interpretability. The former, currently implemented, uncovers the internal "mechanics" of a system. The yet missing interpretability would predict the outcome of changing the input or algorithmic parameters.

Limitations

The proposed approach has several limitations relevant at different steps. First, it should be noted that the accuracy gain from harmonization will be limited if the preprocessing of training and test data is the same. Second, the constructed classification models may not be globally optimal in terms of quality metrics, because we are considering a limited number of parameters to vary within a hyperparametric search, each varying within a limited range of values near defaults. Third, besides choosing the top best features to reduce dimensionality, it might be better to consider different combinations of them. However, in this case, the number of considered models would increase dramatically. Fourth, using schizophrenia as an example, it was shown that if there are some most important CpG sites significantly overcoming the importance of the other, and if they are missed, imputation can not help to improve the result.

Methods

Datasets

We reviewed publicly available whole blood DNA methylation datasets in the GEO repository [115], which include the largest ones from patients with Parkinson's disease and schizophrenia, with at least 50 participants in each group. The following datasets comprise whole blood samples from subjects with Parkinson's disease and healthy controls: GSE145361 [79], GSE111629 [80, 81, 82], GSE72774 [80, 81, 83]. The following datasets comprise whole blood samples from subjects with schizophrenia and healthy controls: GSE152027 [84], GSE84727 [84, 85], GSE80417 [84, 85], GSE116379 (only non famine participants) [86]. We remove from the analysis non-CpG probes [116], SNP-related probes [117], multi-hit probes [118], probes on chromosomes X and Y. We consider only common CpGs in train datasets, the number of CpGs in test datasets can be different.The remaining amount of CpGs after all filtration procedures is shown in Table 1.

Data harmonization

Meta-analysis can be done in different ways: some approaches first analyze different datasets separately and then combine the results into a final estimate; others first combine data from all sets and then analyze the combined data using a single model. The

first class of approaches includes aggregated data and two-step meta-analysis of individual participant data (IPD) [119]. The major advantage of these approaches is their relatively low implementation complexity, while the major disadvantage is the need for raw data. This approach often uses not all available data, but only a subset (usually differentially methylated positions, DMPs, or differentially methylated regions, DMRs). The second class of approaches is called single-step IPD meta-analysis. Although single-step IPD approaches are expected to behave similarly to two-step IPD [119], they provide additional flexibility (e.g., no need to start with raw data) and enable comparison between different models [119]. An important assumption of single-step IPD meta-analysis is the comparability of variables measured in different datasets [120], and therefore data harmonization is crucial to ensure that methylation samples of the same type (same tissue, health status, age, gender, etc.) from different datasets can be compared.

We use the approach to data harmonization proposed in [49], a one-step IPD approach for systematically assessing the impact of different preprocessing methods on the meta-analysis. It has been shown that data preprocessing by different algorithms has a significant impact. RegRCPqn (regional regression on correlated probes with quantile normalization) does not require raw idat files and can be applied to datasets with only β-values or M-values available, which is a common scenario from real life [49]. RCP [121] is a within-array normalization that uses the spatial correlation of DNA methylation at CpG sites to estimate the calibration transformation between type I and type II intensities. The regRCPqn procedure improves the RCP algorithm by including three functions to solve the problem under study. First, it calculates the RCP normalization separately for each type of genomic region (i.e., for CpG belonging to islands, shores, shelves, or open seas) because the distribution of DNA methylation values is different in each of these types of regions [122]. It then performs a quantile normalization between samples, in which CpG values for all samples are normalized separately for each CpG region and for type I and type II probes. Finally, it introduces the possibility of storing the reference distribution and using it to perform quantile normalization of samples from another dataset based on the reference. The reference distribution is calculated separately for each area type and for type I and type II probes. When possible, this distribution is used by regRCPqn to perform normalization based on the reference, again separately for each region and probe type. The dataset with the highest number of participants for each disease is used as the reference, and the others are harmonized relative to it. We consider CpG sites whose distribution of methylation levels does not differ in different train datasets in the control group. To find them, we performed the Mann-Whitney U-test [88] for all CpG sites included in the train datasets for the control group and took CpG sites for which the p-value adjusted according to the Benjamini-Hochberg procedure [123] >0.05. The Mann-Whitney U-test was performed using the scipy package version 1.8.0.

Classification models

The most common type of data for machine learning and deep learning tasks is tabular data, which comprises samples (rows) with the same set of features (columns). DNA methylation is an example of this type of data. Tabular data, unlike image or speech data, is heterogeneous, resulting in dense numerical and sparse categorical features. In addition, the correlation between features is weaker than the spatial or semantic relationship in image or speech data [124]. Variables can be correlated or independent, and features have no positional information. Consequently, it is necessary to detect and use correlation without relying on spatial information [94, 95]. During the last decade, traditional machine learning methods such as gradient-boosted decision trees (GBDT) [91] have continued to dominate tabular data modeling and have demonstrated better performance than deep learning [110]. GBDT trains a series of weak learners to predict the outcome. In GBDT, the weak learner is a standard decision tree that lacks differentiability. Despite their differences, their performance on many problems is similar [92]. When deep neural networks are applied to tabular data, many problems arise, such as lack of locality, missing values, mixed object types (numeric, ordinal and categorical), lack of prior knowledge about the structure of the data. Tree ensemble algorithms are considered a recommended option for real-world problems with tabular data [91, 92, 125]. The XGBoost algorithm [91] is an extendible gradient boosting tree algorithm that achieves state-of-the-art results on many tabular datasets [126, 127].

We consider the following classification models: Logistic Regression with elastic net penalty [89], Support-Vector Machine [90], XGBoost [91], CatBoost [92], LightGBM [93], TabNet [94], NODE [95]. Despite the name, logistic regression is used to solve the binary classification problem. It is a generalized linear model, showing good results for linearly separable data. Support-vector machine is a supervised learning model whose main goal is to construct a separating manifold. This method allows the use of different kernel functions to achieve the best results. XGBoost (Extreme Gradient Boosting) is a scalable, distributed gradient-boosted decision tree (GBDT) machine learning library. GBDT iteratively trains an ensemble of shallow decision trees, with each iteration using error residuals from the previous model to fit the next model. The final prediction is a weighted sum of all tree predictions. XGBoost has one of the best combinations of prediction performance and processing time. CatBoost is an open-source gradient boosting algorithm, which builds symmetric (balanced) trees. At each step, the leaves of the previous tree are separated by the same condition. A feature-split pair is selected and used for all nodes, which provides the least losses. This balanced tree architecture reduces prediction time and controls overfitting. LightGBM is a fast, distributed, high-performance gradient boosting platform that supports the decision tree algorithm. It splits the tree by leaf with the simplest fit, whereas other boosting algorithms split the tree by depth or by level rather than by leaf. Thus, when growing on an equivalent leaf in LightGBM, a leaf-based algorithm can reduce more losses than a level-based algorithm, and therefore lead to greater accuracy. TabNet is a deep neural network designed to handle tabular data. TabNet inputs raw tabular data with no preprocessing and is trained using gradient descent-based optimization. It uses sequential attention to select features at each decision step, providing interpretability and better learning as the learning capability is used for the most useful features, with instance-specific feature selection. Neural Oblivious Decision Ensembles (NODE) is a deep learning architecture designed to handle tabular data. The NODE architecture generalizes ensembles of oblivious decision trees, but benefits from both end-to-end gradient-based optimization and multilevel hierarchical learning capabilities. All of the above models can handle continuous variables (without categorical ones). For classification, we use only the DNA methylation levels of the different CpG sites, which are continuous variables. Parameter values of the trained models which were found by hyperparametric search can be found in Supplementary Table S1. All models have been trained for 2,000 epochs.

Each model was trained on two training datasets and then tested on the remaining independent datasets. Hyperparametric search was used to find optimal parameters for the models. There are a lot of quality metrics for the classification problem: accuracy, precision, recall, f1 score, Cohen's kappa, Matthews correlation coefficient, AUROC, etc. As the main metric, we choose accuracy with weighted averaging to take into account the possible imbalance of the classes. It is calculated according to the formula:

$$\frac{N_{cases}}{N} Accuracy_{cases} + \frac{N_{controls}}{N} Accuracy_{controls}, \quad (1)$$

where N_{cases} is the total number of cases, $N_{controls}$ is total the number of controls, N is the total number of participants. The accuracy for each class is:

$$Accuracy = \frac{TP + TN}{N}, \quad (2)$$

where TP is the number of true positives and TN is the number of true negatives. Adam optimizer and StepLR scheduler were used for the neural network models [128]. Used versions of software packages for the models: XGBoost 1.5.2, CatBoost 1.0.4, LightGBM 3.3.2, TabNet 3.1.1, PyTorch 1.10.0, PyTorch Lightning 1.6.0.

Dimensionality reduction

Based on the features ranking, we performed dimensionality reduction of the models. We performed it, leaving only the most important CpGs for solving the classification problem. For this purpose, we built a series of models with different numbers of features. First, for each disease, we choose the top 10 most important features, and a new model is built for them (the type of model is chosen beforehand - it is the best in terms of accuracy for the full data). Then new models are built on the number of features from 10 to 1000 in increments of 10, and for each such model, the weighted classification accuracy is calculated. For all these models hyperparametric search was performed. According to the dependence of weighted classification accuracy on the number of features we chose as optimal the number of features for which the highest weighted classification accuracy is observed for the considered diseases.

Imputation of missing values

Missing data can be divided into three classes [129]: i) missing completely at random (MCAR) values, if the probability of absence is completely independent of both observed and unobserved variables; ii) missing at random (MAR) values, if the probability of absence is independent of the value itself, but may depend on observed variables; iii) missing not at random (MNAR), if the probability of absence depends on the missing value itself. There is currently no statistical way to determine which category the specific missing data falls into. Assumptions are usually made based on knowledge of the data and the data collection and processing procedure. It is assumed that the missing values represent MCAR/MAR due to random experimental and technology-related errors [53]. It has been shown that missing values lying at the midrange methylation level are more difficult to impute than missing values close to the extremes of the range [54]. This is probably a consequence of the higher variance of methylation values in the middle ranges. Such a scenario could have a profound effect in terms of performance expectations, assuming that many missing values in the data are of the MNAR type and, in particular, lie in the middle range of β values.

In general terms, imputation approaches can be divided into single (SI) and multiple imputation (MI) methods. SI methods replace a missing value with a single acceptable value. MI methods perform multiple SIs and average parameter estimates over multiple imputations to produce a single estimate. Under MCAR/MAR assumptions, the most common imputation methods like mean, median or mode can handle missing data [130]. Such simple imputation methods are used often [131], but they can lead to systematic error or unrealistic results for multivariate datasets. In addition, for large data, this method often performs poorly [132]. The expectation maximization method is an iterative method for handling missing values in numerical datasets, and the algorithm uses an "impute, estimate, and iterate until convergence" approach. Each iteration involves two steps: expectation and maximization. Expectation estimates the missing values given the observed data, while maximization uses the current estimated values to maximize the probability of all data [133, 134, 135]. Besides classical methods, there are approaches to multiple imputation, for example, chained equation for big data [132]. Hot-deck imputation handles missing values by matching missing values with other values in the dataset for several other key variables that have complete values [136, 137]. However, this method does not account for the variability of the missing data. One of the common hot-deck methods is K Nearest Neighbours (KNN) [138]. The KNN algorithm works by classifying the nearest neighbors of missing values and using those neighbors for imputation using a distance measure between instances [139]. Several distance measures can be used for KNN imputation, but Euclidean distance has been shown to provide efficiency and performance [140] and is therefore the most widely used distance measure. However, KNN imputation has weaknesses, such as poor accuracy when imputing variables and introducing false associations where none exists [141]. Another weakness of KNN imputation is that it scans the entire dataset, which increases computation time [142]. However, there are approaches developed in the literature to improve the KNN imputation algorithm [143, 144, 145, 146, 147, 148, 149]. All imputation methods that can deal with continuous variables are suitable for imputing DNA methylation data [53]. To study the effect of these methods on classification accuracy, we removed from consideration 100 CpG sites with the highest importance values for each disease and tried to fill them in. We used previously constructed small models for both diseases. Imputation methods were applied by impyute package version 0.0.8.

Explainable artificial intelligence

Modern machine-learning-based artificial intelligence systems are usually treated as black boxes. However, every decision must be made available for verification by a human expert [150]. One important aspect of model explainability is the ability to verify the system. For example, in healthcare, the use of models that can be interpreted and verified by medical experts is an absolute necessity [151]. Another aspect is to improve the system. The first step to improving the AI system is to understand its weaknesses. Performing weakness analysis on black box models is more difficult than on models that can be interpreted. Furthermore, model interpretability can be useful when comparing different models or architectures [152, 153, 154]. It can be argued that the better we understand what models do (and why they sometimes fail), the easier it becomes to improve them [150]. The next important aspect of explainability is the ability to learn from the system: since modern AI systems learn from millions of examples, they can observe patterns in the data that are inaccessible to humans, who can only learn from a limited number of examples [155, 156]. Explainability is also important for other machine learning methods beyond neural networks [152].

One of the taxonomies to classify explanatory methods is global and local methods [64, 65, 150]. Local interpretable methods apply to a single model result; they can explain the reason for a particular prediction or result. In contrast, global methods try to explain the behavior of the model as a whole. Perturbation is the easiest way to analyze the effect of changing input features on the AI model outputs. This can be accomplished by removing or changing certain input features, running a forward pass, and measuring the difference with the original output data. The input characteristics that most affect the output are evaluated as the most important ones. This is computationally costly, since a direct pass must be run after perturbing each group of input features. Such a perturbation-based approach is Shapley value sampling, which computes approximate Shapley values by taking each input feature for a certain number of times. It is a method from game theory that describes a fair distribution of wins and losses between input functions [157]. As a result, it is not a practical method in its original form, but has led to the development of methods based on game theory, such as

Deep SHapley Additive exPlanations (SHAP) [105]. SHAP has an alternative kernel-based approach to estimating Shapley values inspired by local surrogate models. There is also TreeSHAP, an efficient approach to estimating tree models, as well as DeepExplainer, an enhanced version of the DeepLIFT algorithm for deep neural networks. For the constructed portable models, we applied SHAP to obtain global and local explainability. SHAP values were calculated using eponymous package version 0.40.0.

Data availability statement

No new data was generated. Data used in this study are available from the GEO database (accession numbers GSE145361, GSE111629, GSE72774, GSE84727, GSE80417, GSE152027, GSE116379).

Code availability statement

The source code for the analysis workflow presented in the manuscript is publicly available.

- Project name: DNAmClassMeta
- Project home page: https://github.com/GillianGrayson/DNAmClassMeta
- Operating system(s): Platform independent
- Programming language: Python
- Other requirements: Python 3.8 or higher, pytorch-lightning 1.5.10 or higher, xgboost 1.6.0 or higher, catboost 1.0.5 or higher, lightgbm 3.3.2 or higher, scikit-learn 1.0.2 or higher. All requirements are listed in the requirements.txt file in the project home page.
- License: MIT

Abbreviations

AI: Artificial Intelligence; CatBoost: Categorical Boosting; DMP: Differentially Methylated Position; DMR: Differentially Methylated Region; DNAm: DNA methylation; EWAS: Epigenome-Wide Association Study; FDR: False Discovery Rate; GBDT: Gradient-Boosted Decision Tree; IPD: Individual Participant Data; KNN: K Nearest Neighbors; LightGBM: Light Gradient Boosting Machine; MAR: Missing At Random; MCAR: Missing Completely At Random; MI: Multiple Imputation; MNAR: Missing Not At Random; NODE: Neural Oblivious Decision Ensemble; PTSD: Post-Traumatic Stress Disorder; RCP: Regression on Correlated Probes; SHAP: Shapley Additive Explanations; SI: Single Imputation; XAI: Explainable Artificial Intelligence; XGBoost: Extreme Gradient Boosting.

Competing Interests

The authors declare that they have no competing interests.

Funding

The research was supported by the Ministry of Science and Higher Education of the Russian Federation, Grant for Major Research Projects in Priority Areas of Scientific and Technological Development No. 075-15-2020-808, grant recipient: Lobachevsky State University.

Author's Contributions

Conceptualization: A.K., I.Y., M.G.B., M.I.; Formal analysis: A.K., I.Y.; Methodology: A.K., I.Y., M.G.B.; Software: A.K., I.Y.; Supervision: M.G.B., C.F., M.V., M.I.; Visualization: A.K., I.Y.; Writing – original draft: A.K., I.Y.; Writing – review & editing: A.K., I.Y., M.G.B., C.F., M.V., M.I.

Acknowledgements

The authors acknowledge the use of computational resources provided by the "Lobachevsky" supercomputer.

References

1. Sasaki H, Matsui Y. Epigenetic Events in Mammalian Germ-Cell Development: Reprogramming and Beyond. Nature Reviews Genetics 2008 Feb;9(2):129–140.
2. Igarashi J, Muroi S, Kawashima H, Wang X, Shinojima Y, Kitamura E, et al. Quantitative Analysis of Human Tissue-Specific Differences in Methylation. Biochemical and Biophysical Research Communications 2008 Nov;376(4):658–664.
3. Zemach A, McDaniel IE, Silva P, Zilberman D. Genome-Wide Evolutionary Analysis of Eukaryotic DNA Methylation. Science 2010 May;328(5980):916–919.
4. Ziller MJ, Gu H, Müller F, Donaghey J, Tsai LTY, Kohlbacher O, et al. Charting a Dynamic DNA Methylation Landscape of the Human Genome. Nature 2013 Aug;500(7463):477–481.
5. Horvath S. DNA Methylation Age of Human Tissues and Cell Types. Genome Biology 2013;14(10):R115.
6. Orozco LD, Farrell C, Hale C, Rubbi L, Rinaldi A, Civelek M, et al. Epigenome-Wide Association in Adipose Tissue from the METSIM Cohort. Human Molecular Genetics 2018 Jul;27(14):2586–2586.
7. Smith ZD, Meissner A. DNA Methylation: Roles in Mammalian Development. Nature Reviews Genetics 2013 Mar;14(3):204–220.
8. Lim DHK, Maher ER. Genomic Imprinting Syndromes and Cancer. In: Advances in Genetics, vol. 70 Elsevier; 2010.p. 145–175.
9. Robertson KD. DNA Methylation and Human Disease. Nature Reviews Genetics 2005 Aug;6(8):597–610.
10. Jones PA. Functions of DNA Methylation: Islands, Start Sites, Gene Bodies and Beyond. Nature Reviews Genetics 2012 Jul;13(7):484–492.
11. Jjingo D, Conley AB, Yi SV, Lunyak VV, Jordan IK. On the Presence and Role of Human Gene-Body DNA Methylation. Oncotarget 2012 Apr;3(4):462–474.
12. Christensen BC, Houseman EA, Marsit CJ, Zheng S, Wrensch MR, Wiemels JL, et al. Aging and Environmental Exposures Alter Tissue-Specific DNA Methylation Dependent upon CpG Island Context. PLoS Genetics 2009 Aug;5(8):e1000602.
13. Bell CG, Lowe R, Adams PD, Baccarelli AA, Beck S, Bell JT, et al. DNA Methylation Aging Clocks: Challenges and Recommendations. Genome Biology 2019 Dec;20(1):249.
14. Rakyan VK, Down TA, Balding DJ, Beck S. Epigenome-Wide Association Studies for Common Human Diseases. Nature Reviews Genetics 2011 Jul;12(8):529–541.
15. Liu D, Zhao L, Wang Z, Zhou X, Fan X, Li Y, et al. EWASdb: Epigenome-Wide Association Study Database. Nucleic Acids Research 2019 Jan;47(D1):D989–D993.
16. Birney E, Smith GD, Greally JM. Epigenome-Wide Association Studies and the Interpretation of Disease -Omics. PLOS Genetics 2016 Jun;12(6):e1006105.
17. Moran S, Arribas C, Esteller M. Validation of a DNA Methylation Microarray for 850,000 CpG Sites of the Human Genome Enriched in Enhancer Sequences. Epigenomics 2016 Mar;8(3):389–399.
18. Bibikova M, Lin Z, Zhou L, Chudin E, Garcia EW, Wu B, et al. High-Throughput DNA Methylation Profiling Using Universal

Bead Arrays. Genome Research 2006 Mar;16(3):383–393.

19. Irizarry RA, Ladd-Acosta C, Carvalho B, Wu H, Brandenburg SA, Jeddeloh JA, et al. Comprehensive High-Throughput Arrays for Relative Methylation (CHARM). Genome Research 2008 May;18(5):780–790.
20. Du P, Zhang X, Huang CC, Jafari N, Kibbe WA, Hou L, et al. Comparison of Beta-value and M-value Methods for Quantifying Methylation Levels by Microarray Analysis. BMC Bioinformatics 2010 Dec;11(1):587.
21. Tian T, Wan J, Song Q, Wei Z. Clustering Single-Cell RNA-seq Data with a Model-Based Deep Learning Approach. Nature Machine Intelligence 2019 Apr;1(4):191–198.
22. Lopez R, Regier J, Cole MB, Jordan MI, Yosef N. Deep Generative Modeling for Single-Cell Transcriptomics. Nature Methods 2018 Dec;15(12):1053–1058.
23. Way GP, Greene CS. Extracting a Biologically Relevant Latent Space from Cancer Transcriptomes with Variational Autoencoders. Pacific Symposium on Biocomputing Pacific Symposium on Biocomputing 2018;23:80–91.
24. Titus AJ, Wilkins OM, Bobak CA, Christensen BC. Unsupervised Deep Learning with Variational Autoencoders Applied to Breast Tumor Genome-Wide DNA Methylation Data with Biologic Feature Extraction. Bioinformatics; 2018.
25. Ching T, Himmelstein DS, Beaulieu-Jones BK, Kalinin AA, Do BT, Way GP, et al. Opportunities and Obstacles for Deep Learning in Biology and Medicine. Journal of The Royal Society Interface 2018 Apr;15(141):20170387.
26. Levy JJ, Titus AJ, Petersen CL, Chen Y, Salas LA, Christensen BC. MethylNet: An Automated and Modular Deep Learning Approach for DNA Methylation Analysis. BMC Bioinformatics 2020 Dec;21(1):108.
27. The Cancer Genome Atlas Research Network, Weinstein JN, Collisson EA, Mills GB, Shaw KRM, Ozenberger BA, et al. The Cancer Genome Atlas Pan-Cancer Analysis Project. Nature Genetics 2013 Oct;45(10):1113–1120.
28. Ding W, Chen G, Shi T. Integrative Analysis Identifies Potential DNA Methylation Biomarkers for Pan-Cancer Diagnosis and Prognosis. Epigenetics 2019 Jan;14(1):67–80.
29. Celli F, Cumbo F, Weitschek E. Classification of Large DNA Methylation Datasets for Identifying Cancer Drivers. Big Data Research 2018 Sep;13:21–28.
30. Ma B, Meng F, Yan G, Yan H, Chai B, Song F. Diagnostic Classification of Cancers Using Extreme Gradient Boosting Algorithm and Multi-Omics Data. Computers in Biology and Medicine 2020 Jun;121:103761.
31. List M, Hauschild AC, Tan Q, Kruse TA, Baumbach J, Batra R. Classification of Breast Cancer Subtypes by Combining Gene Expression and DNA Methylation Data. Journal of Integrative Bioinformatics 2014 Jun;11(2):1–14.
32. Dong RZ, Yang X, Zhang XY, Gao PT, Ke AW, Sun Hc, et al. Predicting Overall Survival of Patients with Hepatocellular Carcinoma Using a Three-category Method Based on DNA Methylation and Machine Learning. Journal of Cellular and Molecular Medicine 2019 May;23(5):3369–3374.
33. Hao X, Luo H, Krawczyk M, Wei W, Wang W, Wang J, et al. DNA Methylation Markers for Diagnosis and Prognosis of Common Cancers. Proceedings of the National Academy of Sciences 2017 Jul;114(28):7414–7419.
34. Jurmeister P, Bockmayr M, Seegerer P, Bockmayr T, Treue D, Montavon G, et al. Machine Learning Analysis of DNA Methylation Profiles Distinguishes Primary Lung Squamous Cell Carcinomas from Head and Neck Metastases. Science Translational Medicine 2019 Sep;11(509):eaaw8513.
35. Wajed SA, Laird PW, DeMeester TR. DNA Methylation: An Alternative Pathway to Cancer:. Annals of Surgery 2001 Jul;234(1):10–20.
36. Bollepalli S, Korhonen T, Kaprio J, Anders S, Ollikainen M. EpiSmokEr: A Robust Classifier to Determine Smoking Status from DNA Methylation Data. Epigenomics 2019 Oct;11(13):1469–1486.
37. Lee YC, Christensen JJ, Parnell LD, Smith CE, Shao J, McKeown NM, et al. Using Machine Learning to Predict Obesity Based on Genome-Wide and Epigenome-Wide Gene–Gene and Gene–Diet Interactions. Frontiers in Genetics 2022 Jan;12:783845.
38. Aref-Eshghi E, Rodenhiser DI, Schenkel LC, Lin H, Skinner C, Ainsworth P, et al. Genomic DNA Methylation Signatures Enable Concurrent Diagnosis and Clinical Genetic Variant Classification in Neurodevelopmental Syndromes. The American Journal of Human Genetics 2018 Jan;102(1):156–174.
39. Dogan MV, Grumbach IM, Michaelson JJ, Philibert RA. Integrated Genetic and Epigenetic Prediction of Coronary Heart Disease in the Framingham Heart Study. PLOS ONE 2018 Jan;13(1):e0190549.
40. Gunasekara CJ, Hannon E, MacKay H, Coarfa C, McQuillin A, Clair DS, et al. A Machine Learning Case–Control Classifier for Schizophrenia Based on DNA Methylation in Blood. Translational Psychiatry 2021 Dec;11(1):412.
41. Jabari S, Kobow K, Pieper T, Hartlieb T, Kudernatsch M, Polster T, et al. DNA Methylation-Based Classification of Malformations of Cortical Development in the Human Brain. Acta Neuropathologica 2022 Jan;143(1):93–104.
42. Jo T, Nho K, Bice P, Saykin AJ, For The Alzheimer's Disease Neuroimaging Initiative. Deep Learning-Based Identification of Genetic Variants: Application to Alzheimer's Disease Classification. Briefings in Bioinformatics 2022 Mar;23(2):bbac022.
43. Haghshenas S, Bhai P, Aref-Eshghi E, Sadikovic B. Diagnostic Utility of Genome-Wide DNA Methylation Analysis in Mendelian Neurodevelopmental Disorders. International Journal of Molecular Sciences 2020 Dec;21(23):9303.
44. Xiong Z, Zhang X, Zhang M, Cao B. Predicting Features of Human Mental Disorders through Methylation Profile and Machine Learning Models. In: 2020 2nd International Conference on Machine Learning, Big Data and Business Intelligence (MLBDBI) Taiyuan, China: IEEE; 2020. p. 67–75.
45. Luo X, Wei Y. Batch Effects Correction with Unknown Subtypes. Journal of the American Statistical Association 2019 Apr;114(526):581–594.
46. Leek JT, Scharpf RB, Bravo HC, Simcha D, Langmead B, Johnson WE, et al. Tackling the Widespread and Critical Impact of Batch Effects in High-Throughput Data. Nature Reviews Genetics 2010 Oct;11(10):733–739.
47. Perrier F, Novoloaca A, Ambatipudi S, Baglietto L, Ghantous A, Perduca V, et al. Identifying and Correcting Epigenetics Measurements for Systematic Sources of Variation. Clinical Epigenetics 2018 Dec;10(1):38.
48. Zindler T, Frieling H, Neyazi A, Bleich S, Friedel E. Simulating ComBat: How Batch Correction Can Lead to the Systematic Introduction of False Positive Results in DNA Methylation Microarray Studies. BMC Bioinformatics 2020 Dec;21(1):271.
49. Sala C, Di Lena P, Fernandes Durso D, Prodi A, Castellani G, Nardini C. Evaluation of Pre-Processing on the Meta-Analysis of DNA Methylation Data from the Illumina HumanMethylation450 BeadChip Platform. PLOS ONE 2020 Mar;15(3):e0229763.
50. Garagnani P, Bacalini MG, Pirazzini C, Gori D, Giuliani C, Mari D, et al. Methylation of *ELOVL* 2 Gene as a New Epigenetic Marker of Age. Aging Cell 2012 Dec;11(6):1132–1134.
51. Hannum G, Guinney J, Zhao L, Zhang L, Hughes G, Sadda S, et al. Genome-Wide Methylation Profiles Reveal Quantitative Views of Human Aging Rates. Molecular Cell 2013 Jan;49(2):359–367.
52. Weidner C, Lin Q, Koch C, Eisele L, Beier F, Ziegler P, et al. Aging of Blood Can Be Tracked by DNA Methylation Changes at Just Three CpG Sites. Genome Biology 2014;15(2):R24.
53. Di Lena P, Sala C, Prodi A, Nardini C. Missing Value Estima-

tion Methods for DNA Methylation Data. Bioinformatics 2019 Oct;35(19):3786–3793.
54. Lena PD, Sala C, Prodi A, Nardini C. Methylation Data Imputation Performances under Different Representations and Missingness Patterns. BMC Bioinformatics 2020 Dec;21(1):268.
55. Venkat N. The Curse of Dimensionality: Inside Out 2018;http://rgdoi.net/10.13140/RG.2.2.29631.36006.
56. Levine ME, Lu AT, Quach A, Chen BH, Assimes TL, Bandinelli S, et al. An Epigenetic Biomarker of Aging for Lifespan and Healthspan. Aging 2018 Apr;10(4):573–591.
57. Lu AT, Quach A, Wilson JG, Reiner AP, Aviv A, Raj K, et al. DNA Methylation GrimAge Strongly Predicts Lifespan and Healthspan. Aging 2019 Jan;11(2):303–327.
58. Kurdyukov S, Bullock M. DNA Methylation Analysis: Choosing the Right Method. Biology 2016 Jan;5(1):3.
59. He K, Zhang X, Ren S, Sun J. Deep Residual Learning for Image Recognition. In: 2016 IEEE Conference on Computer Vision and Pattern Recognition (CVPR) Las Vegas, NV, USA: IEEE; 2016. p. 770–778.
60. Cho K, van Merrienboer B, Gulcehre C, Bahdanau D, Bougares F, Schwenk H, et al. Learning Phrase Representations Using RNN Encoder–Decoder for Statistical Machine Translation. In: Proceedings of the 2014 Conference on Empirical Methods in Natural Language Processing (EMNLP) Doha, Qatar: Association for Computational Linguistics; 2014. p. 1724–1734.
61. Deng L, Hinton G, Kingsbury B. New Types of Deep Neural Network Learning for Speech Recognition and Related Applications: An Overview. In: 2013 IEEE International Conference on Acoustics, Speech and Signal Processing Vancouver, BC, Canada: IEEE; 2013. p. 8599–8603.
62. Baldi P. Deep Learning in Biomedical Data Science. Annual Review of Biomedical Data Science 2018 Jul;1(1):181–205.
63. Galkin F, Mamoshina P, Kochetov K, Sidorenko D, Zhavoronkov A. DeepMAge: A Methylation Aging Clock Developed with Deep Learning. Aging and Disease 2021 Aug;12(5):1252–1262.
64. Baehrens D, Schroeter T, Harmeling S, Kawanabe M, Hansen K, Müller KR. How to Explain Individual Classification Decisions. The Journal of Machine Learning Research 2010 Aug;11:1803–1831.
65. Simonyan K, Vedaldi A, Zisserman A. Deep Inside Convolutional Networks: Visualising Image Classification Models and Saliency Maps. arXiv:13126034 [cs] 2014 Apr;.
66. Zeiler MD, Fergus R. Visualizing and Understanding Convolutional Networks. In: Fleet D, Pajdla T, Schiele B, Tuytelaars T, editors. Computer Vision – ECCV 2014, vol. 8689 Cham: Springer International Publishing; 2014.p. 818–833.
67. Bach S, Binder A, Montavon G, Klauschen F, Müller KR, Samek W. On Pixel-Wise Explanations for Non-Linear Classifier Decisions by Layer-Wise Relevance Propagation. PLOS ONE 2015 Jul;10(7):e0130140.
68. Shrikumar A, Greenside P, Shcherbina A, Kundaje A. Not Just a Black Box: Learning Important Features Through Propagating Activation Differences. arXiv:160501713 [cs] 2017 Apr;.
69. Mahendran A, Vedaldi A. Visualizing Deep Convolutional Neural Networks Using Natural Pre-images. International Journal of Computer Vision 2016 Dec;120(3):233–255.
70. Lipton ZC. The Mythos of Model Interpretability. arXiv:160603490 [cs, stat] 2017 Mar;.
71. Ribeiro MT, Singh S, Guestrin C. "Why Should I Trust You?": Explaining the Predictions of Any Classifier. arXiv:160204938 [cs, stat] 2016 Aug;.
72. Zintgraf LM, Cohen TS, Adel T, Welling M. Visualizing Deep Neural Network Decisions: Prediction Difference Analysis. arXiv:170204595 [cs] 2017 Feb;.
73. Doshi-Velez F, Kim B. Towards A Rigorous Science of Interpretable Machine Learning. arXiv:170208608 [cs, stat] 2017 Mar;.
74. Montavon G, Samek W, Müller KR. Methods for Interpreting and Understanding Deep Neural Networks. Digital Signal Processing 2018 Feb;73:1–15.
75. Mahendran A, Vedaldi A. Understanding Deep Image Representations by Inverting Them. arXiv:14120035 [cs] 2014 Nov;.
76. Nguyen A, Yosinski J, Clune J. Multifaceted Feature Visualization: Uncovering the Different Types of Features Learned By Each Neuron in Deep Neural Networks. arXiv:160203616 [cs] 2016 May;.
77. Landecker W, Thomure MD, Bettencourt LMA, Mitchell M, Kenyon GT, Brumby SP. Interpreting Individual Classifications of Hierarchical Networks. In: 2013 IEEE Symposium on Computational Intelligence and Data Mining (CIDM) Singapore, Singapore: IEEE; 2013. p. 32–38.
78. Montavon G, Lapuschkin S, Binder A, Samek W, Müller KR. Explaining Nonlinear Classification Decisions with Deep Taylor Decomposition. Pattern Recognition 2017 May;65:211–222.
79. Vallerga CL, Zhang F, Fowdar J, McRae AF, Qi T, Nabais MF, et al. Analysis of DNA Methylation Associates the Cystine–Glutamate Antiporter SLC7A11 with Risk of Parkinson's Disease. Nature Communications 2020 Dec;11(1):1238.
80. Chuang YH, Paul KC, Bronstein JM, Bordelon Y, Horvath S, Ritz B. Parkinson's Disease Is Associated with DNA Methylation Levels in Human Blood and Saliva. Genome Medicine 2017 Dec;9(1):76.
81. Horvath S, Ritz BR. Increased Epigenetic Age and Granulocyte Counts in the Blood of Parkinson's Disease Patients. Aging 2015 Dec;7(12):1130–1142.
82. Chuang YH, Lu AT, Paul KC, Folle AD, Bronstein JM, Bordelon Y, et al. Longitudinal Epigenome-Wide Methylation Study of Cognitive Decline and Motor Progression in Parkinson's Disease. Journal of Parkinson's Disease 2019 May;9(2):389–400.
83. Paul KC, Binder AM, Horvath S, Kusters C, Yan Q, Rosario ID, et al. Accelerated Hematopoietic Mitotic Aging Measured by DNA Methylation, Blood Cell Lineage, and Parkinson's Disease. BMC Genomics 2021 Dec;22(1):696.
84. Hannon E, Dempster EL, Mansell G, Burrage J, Bass N, Bohlken MM, et al. DNA Methylation Meta-Analysis Reveals Cellular Alterations in Psychosis and Markers of Treatment-Resistant Schizophrenia. eLife 2021 Feb;10:e58430.
85. Hannon E, Dempster E, Viana J, Burrage J, Smith AR, Macdonald R, et al. An Integrated Genetic-Epigenetic Analysis of Schizophrenia: Evidence for Co-Localization of Genetic Associations and Differential DNA Methylation. Genome Biology 2016 Dec;17(1):176.
86. Boks MP, Houtepen LC, Xu Z, He Y, Ursini G, Maihofer AX, et al. Genetic Vulnerability to DUSP22 Promoter Hypermethylation Is Involved in the Relation between in Utero Famine Exposure and Schizophrenia. npj Schizophrenia 2018 Dec;4(1):16.
87. Rauschert S, Raubenheimer K, Melton PE, Huang RC. Machine Learning and Clinical Epigenetics: A Review of Challenges for Diagnosis and Classification. Clinical Epigenetics 2020 Dec;12(1):51.
88. Mann HB, Whitney DR. On a Test of Whether One of Two Random Variables Is Stochastically Larger than the Other. The Annals of Mathematical Statistics 1947 Mar;18(1):50–60.
89. Cox DR. The Regression Analysis of Binary Sequences. Journal of the Royal Statistical Society Series B (Methodological) 1958;20(2):215–242.
90. Cortes C, Vapnik V. Support-Vector Networks. Machine Learning 1995 Sep;20(3):273–297.
91. Chen T, Guestrin C. XGBoost: A Scalable Tree Boosting System. In: Proceedings of the 22nd ACM SIGKDD International Conference on Knowledge Discovery and Data Mining San Francisco California USA: ACM; 2016. p. 785–794.
92. Prokhorenkova L, Gusev G, Vorobev A, Dorogush AV, Gulin A. CatBoost: Unbiased Boosting with Categorical Features.

arXiv:170609516 [cs] 2019 Jan;.
93. Ke G, Meng Q, Finley T, Wang T, Chen W, Ma W, et al. LightGBM: A Highly Efficient Gradient Boosting Decision Tree. In: Advances in Neural Information Processing Systems, vol. 30 Curran Associates, Inc.; 2017. .
94. Arik SO, Pfister T. TabNet: Attentive Interpretable Tabular Learning. arXiv:190807442 [cs, stat] 2020 Dec;.
95. Popov S, Morozov S, Babenko A. Neural Oblivious Decision Ensembles for Deep Learning on Tabular Data. arXiv:190906312 [cs, stat] 2019 Sep;.
96. Henderson-Smith A, Fisch KM, Hua J, Liu G, Ricciardelli E, Jepsen K, et al. DNA Methylation Changes Associated with Parkinson's Disease Progression: Outcomes from the First Longitudinal Genome-Wide Methylation Analysis in Blood. Epigenetics 2019 Apr;14(4):365–382.
97. Kaut O, Schmitt I, Tost J, Busato F, Liu Y, Hofmann P, et al. Epigenome-Wide DNA Methylation Analysis in Siblings and Monozygotic Twins Discordant for Sporadic Parkinson's Disease Revealed Different Epigenetic Patterns in Peripheral Blood Mononuclear Cells. neurogenetics 2017 Jan;18(1):7–22.
98. Walton E, Hass J, Liu J, Roffman JL, Bernardoni F, Roessner V, et al. Correspondence of DNA Methylation Between Blood and Brain Tissue and Its Application to Schizophrenia Research. Schizophrenia Bulletin 2016 Mar;42(2):406–414.
99. Hoang HT, Schlager MA, Carter AP, Bullock SL. DYNC1H1 Mutations Associated with Neurological Diseases Compromise Processivity of Dynein–Dynactin–Cargo Adaptor Complexes. Proceedings of the National Academy of Sciences 2017 Feb;114(9).
100. Chen XJ, Xu H, Cooper HM, Liu Y. Cytoplasmic Dynein: A Key Player in Neurodegenerative and Neurodevelopmental Diseases. Science China Life Sciences 2014 Apr;57(4):372–377.
101. Ma Y, Li J, Xu Y, Wang Y, Yao Y, Liu Q, et al. Identification of 34 Genes Conferring Genetic and Pharmacological Risk for the Comorbidity of Schizophrenia and Smoking Behaviors. Aging 2020 Feb;12(3):2169–2225.
102. Peykov S, Berkel S, Schoen M, Weiss K, Degenhardt F, Strohmaier J, et al. Identification and Functional Characterization of Rare SHANK2 Variants in Schizophrenia. Molecular Psychiatry 2015 Dec;20(12):1489–1498.
103. Chen X, Long F, Cai B, Chen X, Chen G. A Novel Relationship for Schizophrenia, Bipolar and Major Depressive Disorder Part 5: A Hint from Chromosome 5 High Density Association Screen. American Journal of Translational Research 2017;9(5):2473–2491.
104. Hindley G, Bahrami S, Steen NE, O'Connell KS, Frei O, Shadrin A, et al. Characterising the Shared Genetic Determinants of Bipolar Disorder, Schizophrenia and Risk-Taking. Translational Psychiatry 2021 Dec;11(1):466.
105. Chen H, Lundberg S, Lee SI. Explaining Models by Propagating Shapley Values of Local Components. arXiv:191111888 [cs, stat] 2019 Nov;.
106. the Australian Imaging Biomarkers and Lifestyle study, the Alzheimer's Disease Neuroimaging Initiative, Nabais MF, Laws SM, Lin T, Vallerga CL, et al. Meta-Analysis of Genome-Wide DNA Methylation Identifies Shared Associations across Neurodegenerative Disorders. Genome Biology 2021 Dec;22(1):90.
107. Smyth GK, Speed T. Normalization of cDNA Microarray Data. Methods 2003 Dec;31(4):265–273.
108. Johnson WE, Li C, Rabinovic A. Adjusting Batch Effects in Microarray Expression Data Using Empirical Bayes Methods. Biostatistics 2007 Jan;8(1):118–127.
109. Price EM, Robinson WP. Adjusting for Batch Effects in DNA Methylation Microarray Data, a Lesson Learned. Frontiers in Genetics 2018 Mar;9:83.
110. Shwartz-Ziv R, Armon A. Tabular Data: Deep Learning Is Not All You Need. Information Fusion 2022 May;81:84–90.
111. Stilo SA, Murray RM. Non-Genetic Factors in Schizophrenia. Current Psychiatry Reports 2019 Oct;21(10):100.
112. Häfner H, an der Heiden W. Epidemiology of Schizophrenia. The Canadian Journal of Psychiatry 1997 Mar;42(2):139–151.
113. Khan Z, Martin-Montañez E, Muly E. Schizophrenia: Causes and Treatments. Current Pharmaceutical Design 2013-09-31;19(36):6451–6461.
114. INTRuST Clinical Consortium, VA Mid-Atlantic MIRECC Workgroup, PGC PTSD Epigenetics Workgroup, Smith AK, Ratanatharathorn A, Maihofer AX, et al. Epigenome-Wide Meta-Analysis of PTSD across 10 Military and Civilian Cohorts Identifies Methylation Changes in AHRR. Nature Communications 2020 Dec;11(1):5965.
115. Barrett T, Troup DB, Wilhite SE, Ledoux P, Rudnev D, Evangelista C, et al. NCBI GEO: Archive for High-Throughput Functional Genomic Data. Nucleic Acids Research 2009 Jan;37(Database):D885–D890.
116. McCartney DL, Walker RM, Morris SW, McIntosh AM, Porteous DJ, Evans KL. Identification of Polymorphic and Off-Target Probe Binding Sites on the Illumina Infinium MethylationEPIC BeadChip. Genomics Data 2016 Sep;9:22–24.
117. Zhou W, Laird PW, Shen H. Comprehensive Characterization, Annotation and Innovative Use of Infinium DNA Methylation BeadChip Probes. Nucleic Acids Research 2016 Oct;p. gkw967.
118. Nordlund J, Bäcklin CL, Wahlberg P, Busche S, Berglund EC, Eloranta ML, et al. Genome-Wide Signatures of Differential DNA Methylation in Pediatric Acute Lymphoblastic Leukemia. Genome Biology 2013;14(9):r105.
119. Stewart GB, Altman DG, Askie LM, Duley L, Simmonds MC, Stewart LA. Statistical Analysis of Individual Participant Data Meta-Analyses: A Comparison of Methods and Recommendations for Practice. PLoS ONE 2012 Oct;7(10):e46042.
120. Smith-Warner SA, Spiegelman D, Ritz J, Albanes D, Beeson WL, Bernstein L, et al. Methods for Pooling Results of Epidemiologic Studies. American Journal of Epidemiology 2006 Jun;163(11):1053–1064.
121. Niu L, Xu Z, Taylor JA. RCP: A Novel Probe Design Bias Correction Method for Illumina Methylation BeadChip. Bioinformatics 2016 Sep;32(17):2659–2663.
122. Touleimat N, Tost J. Complete Pipeline for Infinium ® Human Methylation 450K BeadChip Data Processing Using Subset Quantile Normalization for Accurate DNA Methylation Estimation. Epigenomics 2012 Jun;4(3):325–341.
123. Benjamini Y, Hochberg Y. Controlling the False Discovery Rate: A Practical and Powerful Approach to Multiple Testing. Journal of the Royal Statistical Society Series B (Methodological) 1995;57(1):289–300.
124. Borisov V, Leemann T, Seßler K, Haug J, Pawelczyk M, Kasneci G. Deep Neural Networks and Tabular Data: A Survey. arXiv:211001889 [cs] 2022 Feb;.
125. Friedman JH. Greedy Function Approximation: A Gradient Boosting Machine. The Annals of Statistics 2001 Oct;29(5).
126. Zhao Y, Chetty G, Tran D. Deep Learning with XGBoost for Real Estate Appraisal. In: 2019 IEEE Symposium Series on Computational Intelligence (SSCI) Xiamen, China: IEEE; 2019. p. 1396–1401.
127. Santhanam R, Uzir N, Raman S, Banerjee S. Experimenting XGBoost Algorithm for Prediction and Classification of Different Datasets. In: National Conference on Recent Innovations in Software Engineering and Computer Technologies (NCRISECT) 2017; 2017. .
128. Kingma DP, Ba J. Adam: A Method for Stochastic Optimization. arXiv:14126980 [cs] 2017 Jan;.
129. Little RJA, Rubin DB. Statistical Analysis with Missing Data. 3rd edition ed. Wiley Series in Probability and Statistics, Hoboken, NJ: Wiley; 2020.
130. Bennett DA. How Can I Deal with Missing Data in My Study? Australian and New Zealand Journal of Public Health 2001 Oct;25(5):464–469.

131. Jerez JM, Molina I, García-Laencina PJ, Alba E, Ribelles N, Martín M, et al. Missing Data Imputation Using Statistical and Machine Learning Methods in a Real Breast Cancer Problem. Artificial Intelligence in Medicine 2010 Oct;50(2):105–115.
132. Khan SI, Hoque ASML. SICE: An Improved Missing Data Imputation Technique. Journal of Big Data 2020 Dec;7(1):37.
133. Lin WC, Tsai CF. Missing Value Imputation: A Review and Analysis of the Literature (2006–2017). Artificial Intelligence Review 2020 Feb;53(2):1487–1509.
134. Rubin LH, Witkiewitz K, Andre JS, Reilly S. Methods for Handling Missing Data in the Behavioral Neurosciences: Don't Throw the Baby Rat out with the Bath Water. Journal of undergraduate neuroscience education: JUNE: a publication of FUN, Faculty for Undergraduate Neuroscience 2007;5(2):A71–77.
135. Delalleau O, Courville A, Bengio Y. Efficient EM Training of Gaussian Mixtures with Missing Data. arXiv:12090521 [cs, stat] 2018 Jan;.
136. Andridge RR, Little RJA. A Review of Hot Deck Imputation for Survey Non-response. International Statistical Review 2010 Apr;78(1):40–64.
137. Cheema JR. A Review of Missing Data Handling Methods in Education Research. Review of Educational Research 2014 Dec;84(4):487–508.
138. Jonsson P, Wohlin C. An Evaluation of K-Nearest Neighbour Imputation Using Likert Data. In: 10th International Symposium on Software Metrics, 2004. Proceedings. Chicago, IL, USA: IEEE; 2004. p. 108–118.
139. Maillo J, Ramírez S, Triguero I, Herrera F. kNN-IS: An Iterative Spark-based Design of the k-Nearest Neighbors Classifier for Big Data. Knowledge-Based Systems 2017 Feb;117:3–15.
140. Amirteimoori A, Kordrostami S. A Euclidean Distance-Based Measure of Efficiency in Data Envelopment Analysis. Optimization 2010 Oct;59(7):985–996.
141. Beretta L, Santaniello A. Nearest Neighbor Imputation Algorithms: A Critical Evaluation. BMC Medical Informatics and Decision Making 2016 Jul;16(S3):74.
142. Acuña E, Rodriguez C. The Treatment of Missing Values and Its Effect on Classifier Accuracy. In: Banks D, McMorris FR, Arabie P, Gaul W, editors. Classification, Clustering, and Data Mining Applications Berlin, Heidelberg: Springer Berlin Heidelberg; 2004.p. 639–647.
143. Lee JY, Styczynski MP. NS-kNN: A Modified k-Nearest Neighbors Approach for Imputing Metabolomics Data. Metabolomics 2018 Dec;14(12):153.
144. Sun B, Ma L, Cheng W, Wen W, Goswami P, Bai G. An Improved K-Nearest Neighbours Method for Traffic Time Series Imputation. In: 2017 Chinese Automation Congress (CAC) Jinan: IEEE; 2017. p. 7346–7351.
145. Cheng D, Zhang S, Deng Z, Zhu Y, Zong M. kNN Algorithm with Data-Driven k Value. In: Luo X, Yu JX, Li Z, editors. Advanced Data Mining and Applications Lecture Notes in Computer Science, Cham: Springer International Publishing; 2014. p. 499–512.
146. Murti DMP, Pujianto U, Wibawa AP, Akbar MI. K-Nearest Neighbor (K-NN) Based Missing Data Imputation. In: 2019 5th International Conference on Science in Information Technology (ICSITech) Yogyakarta, Indonesia: IEEE; 2019. p. 83–88.
147. Huang J, Keung JW, Sarro F, Li YF, Yu YT, Chan WK, et al. Cross-Validation Based K Nearest Neighbor Imputation for Software Quality Datasets: An Empirical Study. Journal of Systems and Software 2017 Oct;132:226–252.
148. Zhu M, Xingbing Cheng. Iterative KNN Imputation Based on GRA for Missing Values in TPLMS. In: 2015 4th International Conference on Computer Science and Network Technology (ICCSNT) Harbin, China: IEEE; 2015. p. 94–99.
149. Zhang S, Li X, Zong M, Zhu X, Cheng D. Learning *k* for kNN Classification. ACM Transactions on Intelligent Systems and Technology 2017 Apr;8(3):1–19.
150. Samek W, Wiegand T, Müller KR. Explainable Artificial Intelligence: Understanding, Visualizing and Interpreting Deep Learning Models. arXiv:170808296 [cs, stat] 2017 Aug;.
151. Caruana R, Lou Y, Gehrke J, Koch P, Sturm M, Elhadad N. Intelligible Models for HealthCare: Predicting Pneumonia Risk and Hospital 30-Day Readmission. In: Proceedings of the 21th ACM SIGKDD International Conference on Knowledge Discovery and Data Mining Sydney NSW Australia: ACM; 2015. p. 1721–1730.
152. Lapuschkin S, Binder A, Montavon G, Muller KR, Samek W. Analyzing Classifiers: Fisher Vectors and Deep Neural Networks. In: 2016 IEEE Conference on Computer Vision and Pattern Recognition (CVPR) Las Vegas, NV, USA: IEEE; 2016. p. 2912–2920.
153. Arras L, Horn F, Montavon G, Müller KR, Samek W. Explaining Predictions of Non-Linear Classifiers in NLP. arXiv:160607298 [cs, stat] 2016 Jun;.
154. Arras L, Horn F, Montavon G, Müller KR, Samek W. "What Is Relevant in a Text Document?": An Interpretable Machine Learning Approach. PLOS ONE 2017 Aug;12(8):e0181142.
155. Schütt KT, Arbabzadah F, Chmiela S, Müller KR, Tkatchenko A. Quantum-Chemical Insights from Deep Tensor Neural Networks. Nature Communications 2017 Apr;8(1):13890.
156. Sturm I, Lapuschkin S, Samek W, Müller KR. Interpretable Deep Neural Networks for Single-Trial EEG Classification. Journal of Neuroscience Methods 2016 Dec;274:141–145.
157. Lipovetsky S, Conklin M. Analysis of Regression in Game Theory Approach. Applied Stochastic Models in Business and Industry 2001 Oct;17(4):319–330.

Word file for manuscript with comments corresponding to changes Click here to access/download;Manuscript;Draft Revision with Comments.docx

Disease classification for whole blood DNA methylation: meta-analysis, missing values imputation, and XAI

Alena Kalyakulina[1*†], Igor Yusipov[1†], Maria Giulia Bacalini[2], Claudio Franceschi[1], Maria Vedunova[3], Mikhail Ivanchenko[1]
[1] Institute of Information Technologies, Mathematics and Mechanics, Lobachevsky State University, Nizhny Novgorod, Russia
[2] IRCCS Istituto delle Scienze Neurologiche di Bologna, Bologna, Italy
[3] Institute of Biology and Biomedicine, Lobachevsky State University, Nizhny Novgorod, Russia
[*] kalyakulina.alena@gmail.com
[†] co-first authorship

Abstract

Background

DNA methylation has a significant effect on gene expression and can be associated with various diseases. Meta-analysis of available DNA methylation datasets requires development of a specific workflow for joint data processing.

Results

We propose a comprehensive approach of combined DNA methylation datasets to classify controls and patients. The solution includes data harmonization, construction of machine learning classification models, dimensionality reduction of models, imputation of missing values, and explanation of model predictions by explainable artificial intelligence (XAI) algorithms. We show that harmonization can improve classification accuracy by up to 20% when preprocessing methods of the training and test datasets are different. The best accuracy results were obtained with tree ensembles, reaching above 95% for Parkinson's disease. Dimensionality reduction can substantially decrease the number of features, without detriment to the classification accuracy. The best imputation methods achieve almost the same classification accuracy for data with missing values as for the original data. XAI approaches have allowed us to explain model predictions from both populational and individual perspectives.

Conclusions

We propose a methodologically valid and comprehensive approach to the classification of healthy individuals and patients with various diseases based on whole blood DNA methylation data using Parkinson's disease and schizophrenia as examples. The proposed algorithm works better for the

Commented [1]: "Pipeline" is renamed to "workflow" according to Reviewer's 2 Comment 1.

Commented [2]: Added the the above-introduced acronym.

former pathology, characterized by a complex set of symptoms. It allows to solve data harmonization problems for meta-analysis of many different datasets, impute missing values, and build classification models of small dimensionality.

Keywords: DNA methylation, machine learning, data harmonization, explainable artificial intelligence

1. Introduction

1.1. Background

DNA methylation (DNAm) plays an important role in human development and is associated with gene expression, genomic imprinting, and other biological processes without altering the DNA sequence [1–8]. Abnormal methylation patterns can lead to numerous diseases [9]. DNA methylation consists of binding a methyl group to cytosine in the cytosine-guanine dinucleotides (CpG sites). Hypermethylation of CpG sites near the gene promoter is known to repress transcription, while hypermethylation in the gene body appears to have an opposite, also less pronounced effect [10, 11]. Changes in DNAm patterns are associated with aging and environmental exposures [12, 13]. Current epigenome-wide association studies (EWAS) test DNAm associations with human phenotypes, health conditions and diseases [14–16]. Microarray-based technologies, such as the Illumina HumanMethylation450 (450K) and HumanMethylationEPIC (850K) arrays [17] are based on the hybridization of bisulfite-converted DNA to 50-mer probes and for each CpG site included in the design allow to estimate the fraction of methylated DNA copies. Two metrics are used to represent methylation levels: the β-value, ranging from 0 to 1, and the M-value, the log2 ratio of the intensities of methylated versus unmethylated probes [18–20]. M-values are more robust quantifiers since β-values close to 0 and 1 suffer from substantial heteroscedasticity [18].

Commented [3]: Typo removed according to Reviewer's 2 Minor Comment 1.

Commented [4]: References added according to Reviewer's 2 Minor Comment 2.

Commented [5]: References added according to Reviewer's 2 Minor Comment 3.

Nowadays, machine learning has become a broadly applicable method for data modeling and analysis in a wide range of applications. The availability of large data sets and a variety of unreinforced generative methods make these approaches more accurate, simple, and relevant in bioinformatics, in particular, for transcriptomic and epigenetic data analysis [21–26]. DNA methylation data are often used for classification tasks. One of the most common examples is the classification of different types of cancer using the TCGA repository [27]. Such classifiers usually demonstrate high accuracy [26, 28–34], based on both cancer-induced changes in methylation and the differences in methylation of various tumor tissues [28, 35]. Classifying different human conditions - phenotypes or pathologies - using DNA methylation data from a single tissue is more difficult. Phenotype classification can question smoking or obesity status, although existing results suggest that such conditions may not be clearly reflected in DNA methylation [26, 36, 37]. Classification of cases and controls for certain diseases is also performed using DNA methylation data. Examples of machine learning applications using epigenetic data include classification of

coronary heart disease, neurodevelopmental syndromes, schizophrenia, Alzheimer's disease, psychiatric disorders and others [38–44].

One of the main challenges is that methylation datasets are limited in the number of samples. Increasing the amount of data requires combining many datasets collected under different conditions and then performing analysis for the merged data, which can cause a variety of problems. There are many factors that lead to significant differences in methylation data that are not directly related to the development of pathological conditions, to name the effect of the laboratory batch, different experimental conditions, normalization, and other [45].

Methylation levels can be affected by systematic variation due to biosample processing, i.e., batch-related variability (a subset of samples processed simultaneously), chip position in batches, and sample position within the chip [46, 47]. Batch effects can dramatically reduce the accuracy of measurements and produce false positive effects if the sample distribution is not uniform [48]. Most of the existing works avoid the question of the applicability of obtained models to new data. A central issue of meta-analysis is data harmonization. Ref. [49] developed an approach to systematically assess the impact of different preprocessing methods on meta-analysis. Its main advantage is the possibility of harmonization of the newly introduced datasets that does not require corrections to the previously analyzed datasets, employed for training the machine learning model.

Making use of new datasets to validate the model raises another problem, that is missing values in the data and the need to fill them in. New (test) datasets can lack information about some relatively small number of CpG sites on which the model was built. Experimental methylation data often contain missing values due to failing quality control checks, which can affect subsequent analysis. Since such missing CpG sites are necessary input parameters for the model, their values must be imputed. Examples include epigenetic clocks, which estimate biological age from small sets of pre-selected age-correlated CpG sites [5, 50–52], sensitive to small deviations in methylation levels [53]. Consequently, accurate imputation of missing data is required to improve the quality of DNA methylation analysis [54].

Dimensionality presents yet another problem. High data dimensionality is often associated with various undesirable consequences: increased computational effort, retraining, and visualization difficulties [55]. High-dimensional data may contain redundant information and introduce noise, while low-dimensional data may be sufficient for comprehensive data characterization. Since methylation data is multivariate, continuous, with nonlinear dependencies, traditional approaches often encounter the problem of multiple hypothesis testing and multicollinearity [26]. In addition, the most common epigenetic models [5, 51, 56, 57] contain a small number of variables to simplify data processing, for better interpretation of the results and for the possibility of applying these models in real life. It is also worth noting that small DNA methylation panels are significantly less costly [58], which is an undeniable advantage for the possibility of widespread use.

Modern artificial intelligence systems based on machine learning are powerful and promising tools in a wide range of applications from computer vision, machine translation and speech recognition [59–61] to the analysis of biomedical data, in particular DNA methylation [26, 62, 63].

Commented [6]: Rephrased according to Reviewer's 2 Minor Comment 4.

Commented [7]: Rephrased according to Reviewer's 2 Minor Comment 5.

However, while these models provide impressive predictive accuracy, their nonlinear structure makes them poorly interpretable, i.e. it is hard to explain what information in the input data leads AI to particular outputs. The need for trustworthy solutions has recently attracted much attention to methods that would "open" black box models [64–74]. This includes developing methods to help better understand what the model has learned [75, 76] as well as methods to explain individual predictions [65–67, 77, 78].

In summary, individual DNA methylation datasets contain an insufficient number of samples to apply machine learning approaches, so there is a need to combine and harmonize different datasets. Problems that arise on the way include tackling batch effects in individual datasets, missing values for certain samples, and high data dimensionality. Here, we analyze several existing fragmented solutions to these problems, develop a generalized unifying approach integrated in a workflow, validate it and demonstrate its efficiency.

Commented [8]: "Pipeline" is renamed to "workflow" according to Reviewer's 2 Comment 1.

1.2. Study design and novelty

Our primary goal is to offer a methodologically complete workflow for building machine learning models, classifying cases and controls for various diseases from whole blood DNA methylation data on many datasets, ranging from data harmonization to explainable artificial intelligence models. DNA methylation data are taken from different human body tissues, but the most widespread is whole blood methylation, the least invasive analysis and, therefore, of broad diagnostic prospects. We restrict our analysis to this kind of data. Our workflow solves a problem of harmonization of methylation data from different datasets. They are collected in different laboratories, with different setups and experimental conditions. In general, the data are of different quality, and have been preprocessed differently. Harmonization is used to eliminate the unavoidable bias between the data and to minimize the associated machine learning model errors. The proposed workflow uses harmonization with the selection of a reference dataset, in which case all other datasets are aligned with the reference one, so that when a new dataset is introduced, there is no need to renormalize the training data and hence rebuild the model. The workflow uses the generally recognized types of machine learning models for classification on methylation data in tabular representation, in particular gradient-boosted decision trees. A hyperparametric search for the optimal combination of the parameters of these models is performed to ensure the best classification accuracy. Next, the dimensionality of the feature space is reduced to build portable models. In such models, the number of features has the same order as the most popular epigenetic models, such as the Horvath clock (353 CpG sites) [5], Hannum clock (71 CpG sites) [51], DNAm PhenoAge (513 CpG sites) [56], DNAm GrimAge (1030 unique CpGs were used to predict plasma protein levels) [57]. Such portable models allow them to be used for early diagnosis of various diseases - analysis of small CpG panels is much cheaper than full-genome analyses. Reducing the dimensionality of the data can also help discard noisy features that do not carry relevant information for classifiers. Also, the proposed approach includes the possibility of imputing missing values (CpG sites), and different approaches are used for this purpose. This is especially important when testing the model on new data, where some CpG sites critical for the model may

Commented [9]: "Pipeline" is renamed to "workflow" according to Reviewer's 2 Comment 1.

Commented [10]: "Pipeline" is renamed to "workflow" according to Reviewer's 2 Comment 1.

Commented [11]: "Pipeline" is renamed to "workflow" according to Reviewer's 2 Comment 1.

Commented [12]: "Pipeline" is renamed to "workflow" according to Reviewer's 2 Comment 1.

be missed (e.g., because of technical errors in data acquisition and processing or failing quality checks). For the best models in terms of accuracy, explainable artificial intelligence (XAI) methods are applied to explore both the global influence of individual CpG sites on model predictions and to get explanations of how the methylation level values of individual CpG sites for specific subjects shape their individual predictions. Lists of the most important CpG sites in terms of machine learning models are compared with lists of CpG sites (and their corresponding genes) from existing studies associated with the considered diseases. Biological pathways of diseases based on these lists are identified and investigated.

2. Results

Larger training sample sizes provide better quality of machine learning models. The currently available DNA methylation data sets do not exceed several thousand samples, and that could hardly change in the near future due to complexity and cost of study. Merging different data sets, therefore, appears a practical way to circumvent size limitations. However, it poses many challenges, such as the need to harmonize datasets collected under different conditions and pre-processed in different ways, the need to fill in missing values in a way that preserves patterns in the data, the reduction of excessively high dimensionality of input variables with a relatively small number of samples. These issues have been addressed separately; below we report an integrated solution that brings together the data processing and analysis steps and the resulting methodologically complete workflow for solving the classification problem based on merging several independent DNA methylation data. A schematic representation of the proposed workflow is shown in Figure 1.

Commented [13]: "Pipeline" is renamed to "workflow" according to Reviewer's 2 Comment 1.

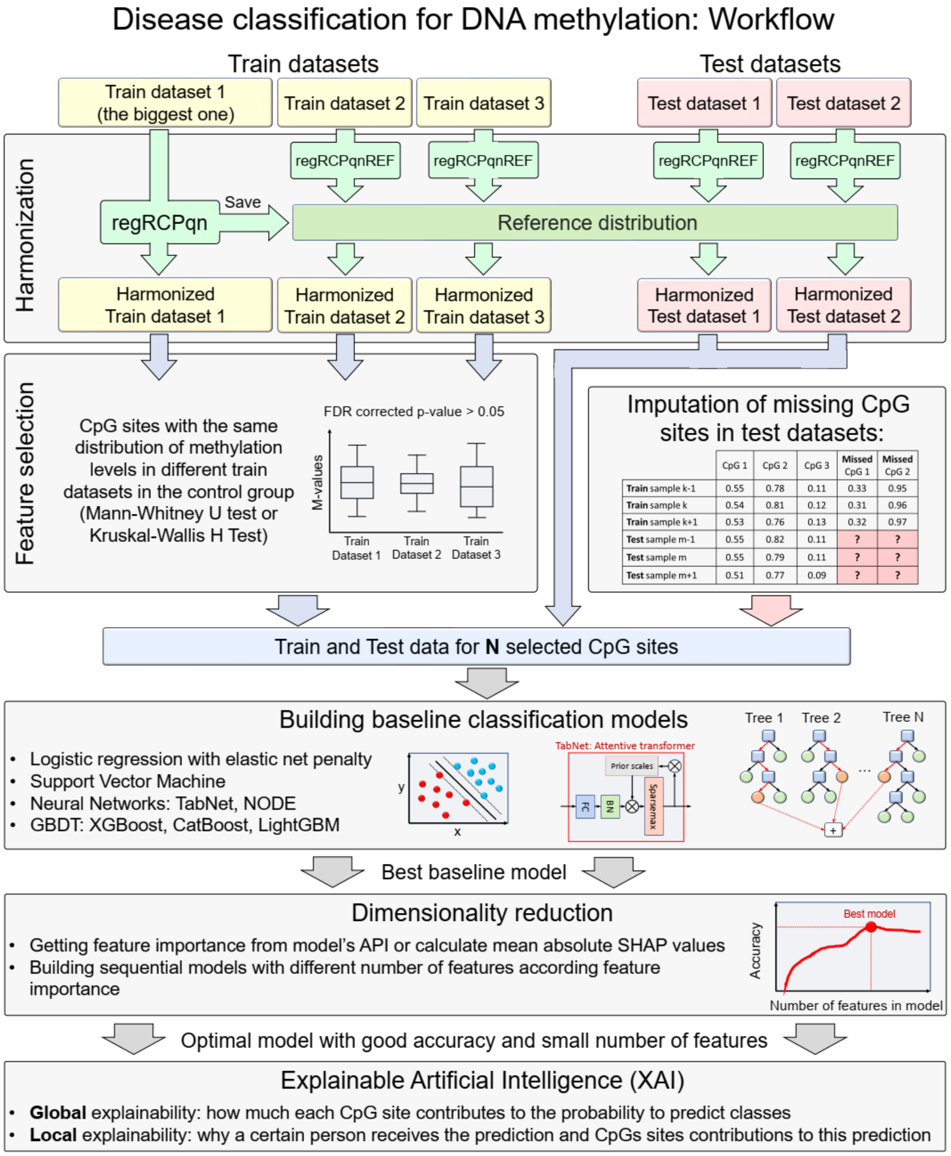

	CpG 1	CpG 2	CpG 3	Missed CpG 1	Missed CpG 2
**Train** sample k-1	0.55	0.78	0.11	0.33	0.95
**Train** sample k	0.54	0.81	0.12	0.31	0.96
**Train** sample k+1	0.53	0.76	0.13	0.32	0.97
**Test** sample m-1	0.55	0.82	0.11	?	?
**Test** sample m	0.55	0.79	0.11	?	?
**Test** sample m+1	0.51	0.77	0.09	?	?

Figure 1. Workflow for classifying cases and controls of various diseases based on DNA methylation data proposed in this paper.

Commented [14]: Added schematic representation of the workflow according to Reviewer's 1 Comment 1 and Reviewer's 2 Comment 1.

2.1. Datasets and machine learning tasks

We studied whole blood DNA methylation datasets generated on subjects with Parkinson's disease or schizophrenia. We selected 3 datasets that contain samples from subjects with Parkinson's disease and healthy controls: GSE145361 [79], GSE111629 [80–82], GSE72774 [80, 81, 83] and 4 datasets that contain samples from subjects with schizophrenia and healthy controls: GSE152027 [84], GSE84727 [84, 85], GSE80417 [84, 85], GSE116379 (non-famine participants) [86]. Information about considered datasets is summarized in Table 1, in particular, the number of cases and controls, whether the dataset has been used as train or test, the original preprocessing type, the number of CpGs.

For each disease, we built machine learning models to classify cases vs. controls. Some of these datasets are used as train data for building the model, and the rest is used to test the model. For each disease we selected a reference dataset, against which harmonization was performed. As it can be seen from Table 1, the original preprocessing is the same for the majority of the considered datasets with schizophrenia patients, but it varies considerably among the different datasets for Parkinson's disease. To reduce the influence of the laboratory-specific data collection and processing conditions on classification results, harmonization is necessary.

Table 1. Main characteristics of considered datasets. For each disease, the bold row represents the reference dataset for the harmonization. Number of CpGs is common for train datasets in each disease. Three largest datasets for schizophrenia, GSE84727, GSE80417 and GSE152024, have the same preprocessing.

Disease	Dataset	Number of cases	Number of controls	Train or Test subset	Raw IDAT available?	Number of CpGs	Original preprocessing
Parkinson's disease	**GSE145361**	**959**	**930**	**Train**	**Yes**	**411761**	**Data processing: Genome Studio software**
	GSE111629	334	237	Train	Yes		Data processing: R software v3.4.2<br>Functional normalization: minfi R package
	GSE72774	289	219	Test	No	411979	Data processing: BeadStudio software v3.2
Schizophrenia	**GSE84727**	**414**	**433**	**Train**	**No**	**399625**	**Importing: methylumi R package methylumIDAT function<br>Preprocessing: watermelon R package pfilter and dasen functions**
	GSE80417	353	322	Train	No		Importing: methylumi R package methylumIDAT function<br>Preprocessing: watermelon R package pfilter and dasen functions
	GSE152027	290	203	Test	No	411901	Importing: methylumi R package methylumIDAT function<br>Preprocessing: watermelon R package pfilter and dasen functions
	GSE116379	51	54	Test	No	407781	Removed: X and Y chromosome, non-specific binding probes, failed probes based on a detection p-value larger than 0.001 and bead count lower than 5, probes with SNPs of Minor Allele Frequency larger than 5 percent within 10 base pairs of the primer<br>Functional normalization: minfi R package

2.2. Meta-analysis and harmonization

Combining different DNA methylation datasets can improve the statistical power to test hypotheses and identify epigenetic signatures by meta-analysis. However, such meta-analysis also poses serious problems related to data harmonization, which is often not considered [87]. This is especially true for DNA methylation, where data is often only available in the preprocessed rather than raw form, and where diverse preprocessing pipelines are used [49]. Developed in [49] approach regRCPqn (regional regression on correlated probes with quantile normalization) allows for meta-analysis even if the raw data are not available. Importantly, as emerging datasets are aligned, the already treated datasets do not require renormalization. Therefore, we apply this approach to harmonization with reference. The largest dataset for each disease is taken as the reference, and other datasets are harmonized relative to it. The schematic representation of the harmonization process is shown in Figure 2.

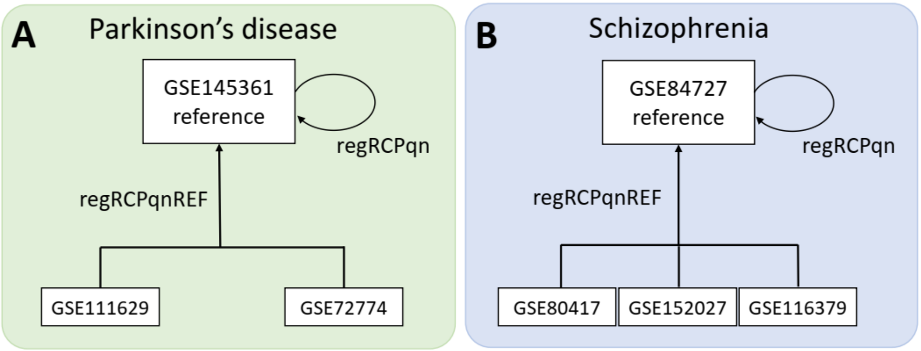

Figure 2. Schematic representation of harmonization procedure for (A) Parkinson's disease and (B) Schizophrenia datasets.

For machine learning models, we used only those CpG sites that have the same distribution of methylation levels in different train datasets in the control group (methylation levels in the case group typically have greater variability because of disease heterogeneity). We used the Mann-Whitney U-test [88] to compare DNA methylation values of healthy participants from the considered train datasets before and after harmonization. After harmonization, the number of CpG sites with the adjusted p-value >0.05 (not significantly different between healthy subjects from the considered train datasets) increased from 43019 to 50911 for Parkinson's disease and from 35145 to 110137 for schizophrenia. Figure 3 illustrates the change in the distributions of methylation level values before and after harmonization. In particular, CpG sites whose methylation level distributions differed significantly before harmonization (FDR-corrected p-values<0.05) manifest similar distributions after harmonization (FDR-corrected p-values>0.05).

Commented [15]: Added A and B letters for subplots according to Reviewer's 1 Comment 2.

Commented [16]: Added according to Reviewer's 2 Comment 3.

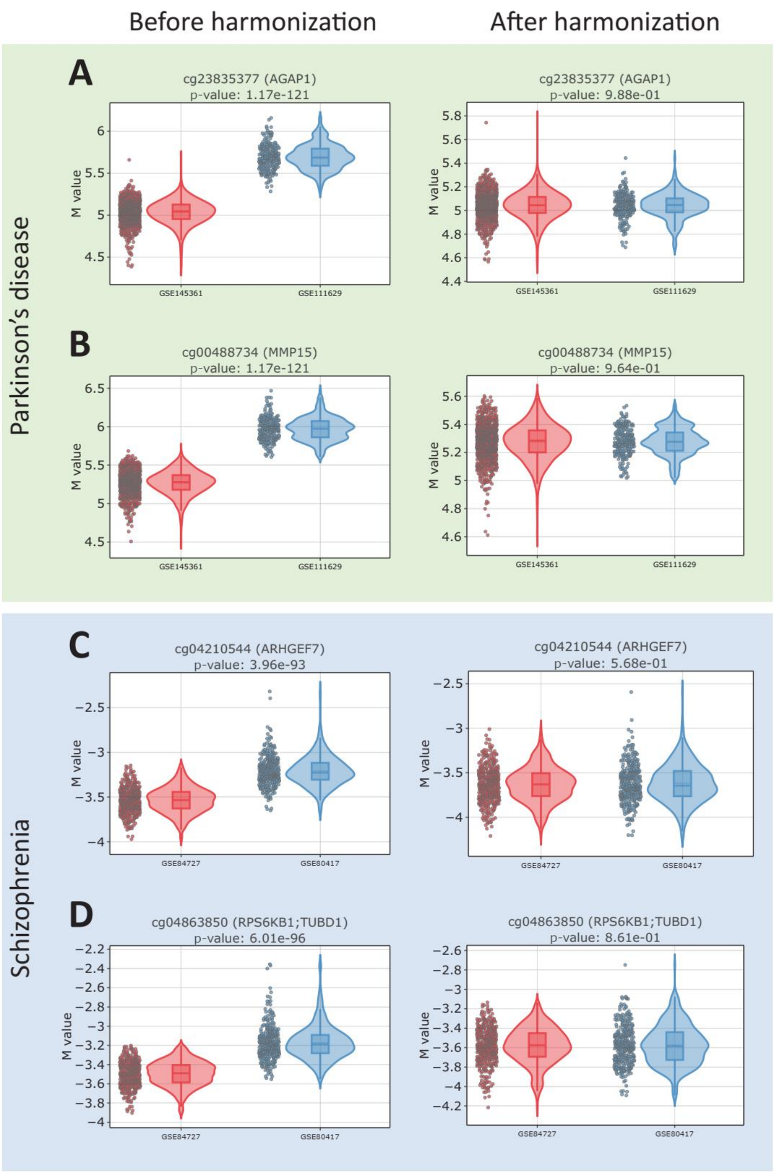

Figure 3. Examples of M-values methylation levels distribution for control groups before and after harmonization for Parkinson's disease examples (A) cg23835377 and (B) cg00488734, Schizophrenia examples (C) cg04210544 and (D) cg04863850.

Commented [17]: Added A, B, C, D letters for subplots according to Reviewer's 1 Comment 2.

2.3. Classification models

The most common type of data representation for machine learning is tabular, and DNA methylation data fulfills it. Typically, the rows refer to participants, the columns refer to CpG sites, and the cells of the table contain the methylation levels of each CpG site for each participant. There are many machine learning models designed to work with tabular data: Logistic Regression with elastic net penalty [89], Support-Vector Machine [90], XGBoost [91], CatBoost [92], LightGBM [93], TabNet [94], NODE [95]. Main characteristics of the models are summarized in Table 2.

Commented [18]: Added Logistic Regression and SVM according to Reviewer's 2 Comment 5.

Table 2. Main characteristics of the considered classification models.

Commented [19]: Added Logistic Regression and SVM according to Reviewer's 2 Comment 5.

Commented [20]: Imputation method column was removed according to Reviewer's 2 Comment 6.

Model	Type	Feature importance API
Logistic Regression	Generalized linear model	Yes
Support-Vector Machine	Supervised learning model constructing the separating manifold	Only for linear kernel
XGBoost	Gradient-boosted decision tree ensemble	Yes
CatBoost	Gradient-boosted decision tree ensemble	Yes
LightGBM	Gradient-boosted decision tree ensemble	Yes
TabNet	Deep neural network	Yes
NODE	Gradient-boosted decision tree ensemble	No

For each disease, all considered datasets were divided into training and test ones (as stated in Table 1). We trained all models on two training datasets and then tested on the remaining datasets. Accuracy with weighted averaging was the main quality metric, as it can handle situations with possible imbalance of the classes (the number of participants in different classes varies significantly). As discussed in the above, the approach fulfills the requirement that the model does not have to be trained again as the new data set is considered. Moreover, the models must be trained to classify biological differences in methylation data rather than traces of different experimental conditions in different laboratories. Accordingly, we do not mix train and test datasets and do not perform cross-validation. To find the optimal combination of model parameters that provides the best accuracy, we used a hyperparametric grid search (the values are presented in Supplementary Table 1).

Commented [21]: Added information about hyperparametric search according to Reviewer's 1 Comment 5

Newly introduced datasets may lack some CpG sites that are present in already trained models; in this case various imputation methods are applied (cf. Sections 2.5, 4.5 for more details). Models for Parkinson's disease for non-harmonized data are trained on 43019 CpG sites, for harmonized data on 50911 CpG sites. Among these, the Parkinson's disease test dataset GSE72774 lacks 38 CpG sites in the non-harmonized data and 34 CpG sites in the harmonized data. Models for schizophrenia for non-harmonized data train on 35145 CpG sites, for harmonized data train on 110137 CpG sites. The first test dataset for schizophrenia GSE152027 lacks 9 CpG sites in the non-harmonized data and 36 CpG sites in the harmonized data. The second test dataset for

schizophrenia GSE116379 lacks 268 CpG sites in the non-harmonized data and 609 CpG sites in the harmonized data. These missed CpG sites are imputed using KNN methods with K=1. However, this imputation does not have a significant effect on the result, because, as will be shown later, all the missed CpG sites are not at the top of the features in terms of importance.

Commented [22]: Added information about imputation of missing values in baseline models.

Table 3. Binary classification results of baseline models for non-harmonized and harmonized data. For Parkinson's disease (green background) and schizophrenia (blue background), results comparing the accuracy of different models for non-harmonized and harmonized methylation data are shown.

Commented [23]: Figure with the classification results is replaced by the table according to the Reviewer's 1 Comment 4.

Model	Parkinson's disease		Schizophrenia			
	GSE72774		GSE152027		GSE116379	
	Non-harmonized	Harmonized	Non-harmonized	Harmonized	Non-harmonized	Harmonized
Logistic Regression	0.71	0.93	0.63	0.66	0.56	0.66
SVM	0.67	0.92	0.62	0.66	0.58	0.65
XGBoost	0.72	0.95	0.67	0.71	0.56	0.66
CatBoost	0.71	0.94	0.68	0.72	0.59	0.71
LightGBM	0.76	0.97	0.68	0.71	0.58	0.67
TabNet	0.69	0.93	0.63	0.66	0.58	0.65
NODE	0.71	0.92	0.62	0.66	0.56	0.65

Table 3 shows the results of cases vs. controls classification by baseline models based on non-harmonized and harmonized whole blood methylation data for Parkinson's disease and schizophrenia on test datasets. For each combination of harmonization type, disease, and test dataset, the best weighted accuracy values for all constructed models is given. All imputation methods described in Section 2.5 do not significantly change the quality of the resulting models, because all missed CpGs in test datasets have a very low value of feature importance in models with corresponding API.

The results confirm that harmonization must be applied and is most efficient for the datasets with different preprocessing methods. In particular, for Parkinson's disease, all datasets have different original preprocessing, and the best model trained on such data shows a result of 76%. When these data are harmonized, accuracy improves dramatically to 97%. For both non-harmonized and harmonized data, for Parkinson's disease, the best model in terms of weighted accuracy is LightGBM. For schizophrenia, only one of 4 datasets has a different preprocessing (GSE116379, Table 1). Then, harmonization does not significantly affect the quality of the built models if the datasets have the same preprocessing (68% without harmonization, 72% with harmonization in the best models). The best model is LightGBM for non-harmonized data and

CatBoost for harmonized data. However, applying models trained on non-harmonized data to data with a different preprocessing gives a poor result for binary classification - 59%. Harmonization of data improves the performance of the trained models, making them close to the best results obtained for schizophrenia in terms of quality - 71%. It is also worth noting that the overall classification quality for these two diseases on methylation data is very different, possibly due to the different etiology and molecular mechanisms involved in the two diseases.

Best accuracy models allow us to extract importance values for all features. The ranking of the most important features for these models for Parkinson's disease and schizophrenia is shown in Figure 4. It is worth noting that for schizophrenia, there is one outstanding CpG with the highest importance for classification, while the others have much lower values. For Parkinson's disease, the situation is more uniform. These rankings can be used for the dimensionality reduction of the built models.

2.4. Dimensionality reduction

As a result of applying different baseline models to methylation data to classify cases vs controls, the ones with an API for feature extraction showed the best accuracy. Based on the obtained ranking of the features (Figure 4), we performed dimensionality reduction of the constructed models. Most common epigenetic models comprise few CpG sites, no more than several hundred (for example, those used to predict epigenetic age like Horvath' clock and Hannum clock). First, models based on few features show significantly better performance while maintaining similar classification accuracy. Second, such models are less memory-consuming.

Along these lines, we reduced the dimensionality of the model, leaving only the most important features for classification. Figure 4 shows the dependence of weighted classification accuracy on the number of features in the model for Parkinson's disease and for schizophrenia. It first increases, until reaching a certain optimal number of features, and then changes weakly. For Parkinson's disease, the best weighted accuracy of 96% is observed for 890 CpG sites, with an accuracy value changed by only 1% compared to the full data (50911 CpG sites). For schizophrenia, the best weighted accuracy of 75% is observed for 670 CpG sites for the test dataset GSE152027 and 70% for the test dataset GSE116379. The optimal model for schizophrenia was chosen as the one for the GSE152027. The accuracy values changed by no more than 3% compared to the full data (110137 CpG sites). The list of CpG sites that make up these small models, as well as basic information about them (gene, chromosome, relation to the CpG island) is presented in Supplementary Table 2. The resulting CpG lists were compared with previously published lists of biomarkers associated with Parkinson's disease [80, 96, 97] and schizophrenia [84, 98]. Interestingly, for Parkinson's disease, there is practically no overlap with the previous results, except for one CpG site from [96]. This CpG belongs to the gene DYNC1H1, which is associated with neurological and neurodegenerative diseases [99, 100]. For schizophrenia, only 15 CpG sites are common with [84]. Some of the connected genes like PRKCZ, SHANK2, ZNF608, PRDM16 were also identified as schizophrenia risk factors [101–104]. Genes, corresponding to CpG sites, from the optimal small model for Parkinson's disease were enriched in several gene ontologies

related to neuronal and metabolic processes, whereas genes from schizophrenia models were enriched in gene ontologies related to cell development processes (Supplementary Table 3).

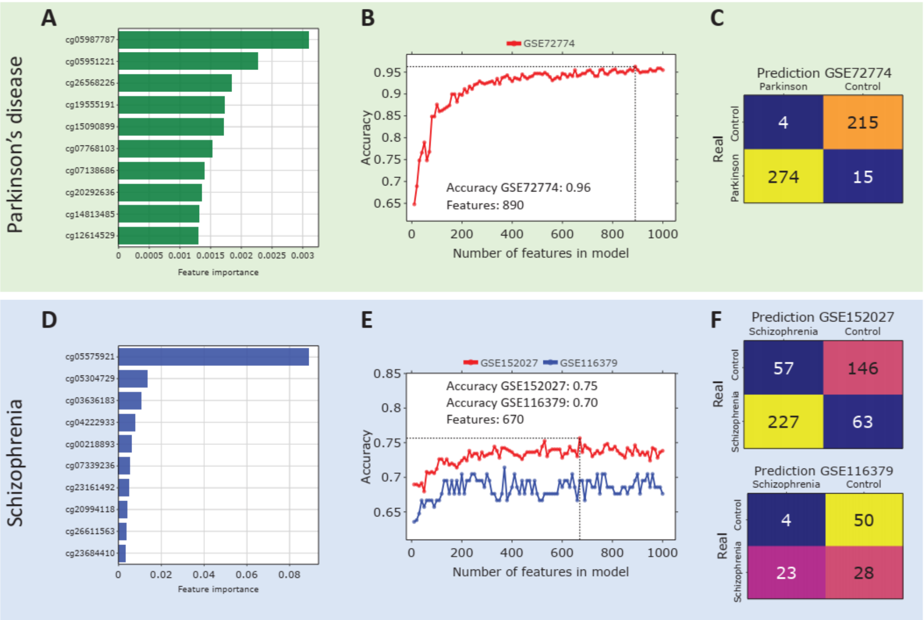

Figure 4. Dimensionality reduction for the best classification models. Parkinson's disease: (A) Top-10 features for the best classification model LightGBM with the normalized importance values. (B) Dependence of the weighted accuracy on the number of features in the model. Dotted line corresponds to the optimal small model with accuracy value 0.96 and 890 features. (C) Confusion matrix for optimal model. Schizophrenia: (D) Top-10 features for the best classification model CatBoost with the normalized importance values. (E) Dependence of the weighted accuracy on the number of features in the model. Dotted line corresponds to the optimal small model with accuracy value 0.75 for GSE152027, 0.70 for GSE116379 and 670 features. (F) Confusion matrix for optimal models.

2.5. Imputation of missing values

For trained machine learning models (both large and small), it is important that there are no missing values in the upcoming test data. Since it is impossible to guarantee their absence, various imputation methods are used to fill them in. Since not all models support data imputation, we use the most popular of them: mean, median, mode, random, chained equations, expectation maximization, KNN with different numbers of neighbors (from 1 to 3). To study the effect of these imputation methods on the classification accuracy, we consider the following simulation experiment. For each disease, we consider only the best small models, obtained at the previous

Commented [24]: Added A, B, C, D, E, F letters for subplots according to Reviewer's 1 Comment 2.

step (LightGBM with 890 CpG sites for Parkinson's disease and CatBoost with 670 CpG sites for schizophrenia). For these models we "remove" 100 CpG sites with the highest importance values and impute them. The number of CpG sites was chosen to induce a significant drop in accuracy and to sharpen the differences in efficiency between the imputation methods. The missing CpG sites do not take part in the construction of small optimal models, so the actually existing CpG sites are removed from consideration. Table 4 shows results for the considered test datasets. For Parkinson's disease, KNN with one neighbor kept the classification accuracy at the baseline level of the data without missing values. Imputation with mode also showed good results, losing only 3%. The other methods achieved an accuracy of no more than 90%. For schizophrenia, none of the approaches achieved the baseline accuracy for data without missing values. This may be explained by the critical importance of specific features for classification. KNN with one neighbor for both datasets shows one of the best imputation results, for GSE152027 chained equation and expectation maximization perform better than KNN by 3% and 2%, respectively. Median and random values methods show unsatisfactory results in all experiments.

Table 4. Comparison of different missing value imputation methods and their effect on weighted classification accuracy for Parkinson's disease (green background) and schizophrenia (blue background). In all cases, 100 CpG sites with the highest importance values were dropped.

Method	Parkinson's disease	Schizophrenia	
	GSE72774	GSE152027	GSE116379
**Original (no missed data)**	**0.96**	**0.75**	**0.7**
Mean	0.78	0.65	0.67
Median	0.81	0.59	0.55
Mode	0.93	0.55	0.54
Random	0.86	0.62	0.54
Chained equation	0.89	0.68	0.61
Expectation maximization	0.87	0.67	0.54
KNN (K = 1)	0.96	0.65	0.67
KNN (K = 2)	0.9	0.65	0.67
KNN (K = 3)	0.86	0.65	0.67

Commented [25]: Imputation experiment description rephrased according to Reviewer's 2 Comment 9.

Commented [26]: Figure with the imputation results is replaced by the table according to the Reviewer's 1 Comment 4.

Commented [27]: Figure with the imputation results is replaced by the table according to the Reviewer's 1 Comment 4.

2.6. Explainable artificial intelligence

Even the most accurate machine learning models make mistakes on upcoming data. It presents a major challenge for those models that work as “black boxes” with unknown principles behind made decisions. SHapley Additive exPlanations (SHAP) help to understand why the model makes its predictions from the global and local points of view [105].

The global explainability of the constructed models on the training data for Parkinson's disease and schizophrenia is illustrated in Figure 5. Beeswarm plots show the relationship between SHAP values and methylation levels for the most important CpG sites. For each CpG site, the distributions of methylation levels for all participants are shown. In particular, for Parkinson's disease, most of the participants in the CpG site cg05987787 have low methylation levels, which positively affects the probability of predicting the disease. The scatter plots show in detail the distribution of methylation levels in different participants and SHAP values. We can see that M-values below 4 have a positive effect on the probability of predicting disease, while M-values above 4 have a negative effect. The black line divides the areas of positive and negative influence of SHAP values on the prediction of disease probability. The opposite situation is observed for the CpG site cg05951221. M-values below -1 have a negative effect on the probability of predicting disease, while M-values above -1 have a positive effect. Similar plots are shown for schizophrenia. The beeswarm plot shows that there is one most important CpG site, cg05575921, that contributes the most to the probability of predicting disease, as previously shown, and the other CpG sites have a much smaller effect.

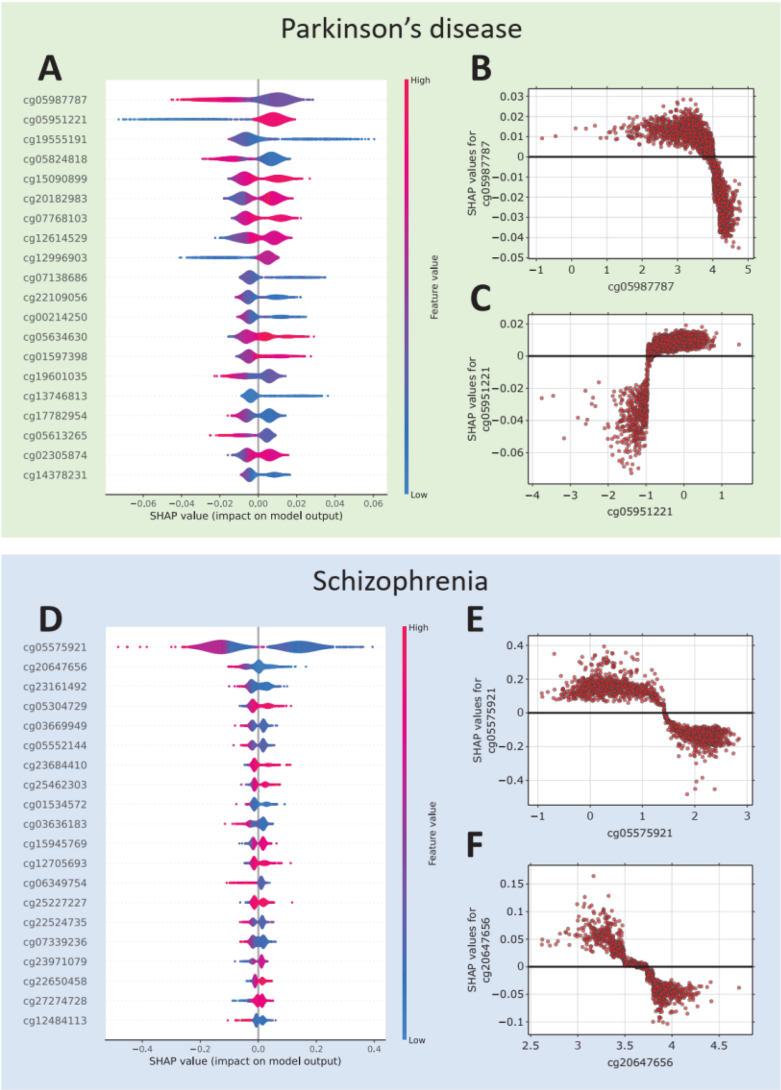

Figure 5. Global explainability based on SHAP values. Parkinson's disease: (A) Beeswarm plots show the dependence of SHAP values for each CpG site on their methylation levels. Each dot represents one participant. (B) Dependence of SHAP values on methylation M-values for cg05987787. The black line separates the areas of negative and positive influence of SHAP values on the probability of predicting Parkinson's disease. (C) Dependence of SHAP values on methylation M-values for cg05951221. Schizophrenia: (D) Beeswarm plots show the

dependence of SHAP values for each CpG site on their methylation levels. Each dot represents one participant. (E) Dependence of SHAP values on methylation M-values for cg05575921. (F) Dependence of SHAP values on methylation M-values for cg20647656.

Commented [28]: Added A, B, C, D, E, F letters for subplots according to Reviewer's 1 Comment 2.

The local explainability of predictions on the test data is shown in Figure 6. The top row presents heatmaps with participants on the x-axis, CpG sites on the y-axis, and SHAP values encoded on a color scale. The participants are ordered based on the probability to predict the disease. Model output is shown above the heatmap matrix. The black line represents the probability of predicting the disease for each participant. It follows that for Parkinson's disease, where the model works with high accuracy, this line is quite smooth and similar to the softmax function. Whereas for schizophrenia, for which the models have much lower accuracy, these probability plots are more fragmented. As shown earlier, for schizophrenia, one CpG site is the most important, so it has the highest absolute SHAP values and appears the brightest in the heatmaps. The center and bottom lines represent waterfall plots for participants with the disease and controls, respectively. They allow for explaining the model output for each participant separately. The bottom part of the waterfall plot shows the base probability of the model to predict disease, and then each line shows how a positive (red) or negative (blue) contribution from each CpG site moves the probability to the model output for that prediction. The output of the model is the probability of predicting disease. If the probability is greater than 50%, the model identifies the participant as a case, otherwise it identifies the participant as a control. The baseline probability is the average probability of the model predicting on the test data. Because baseline probability is a characteristic of the model, it depends on the quality of the model. If the model has reasonably good accuracy, then the base probability is close to the proportion of participants in a particular class. In the examples from middle line of the Figure 6 for all participants with diseases, the probability of predicting disease in the examples was above 97% (models identify them as cases almost for sure); for control participants (bottom line of Figure 6), the probability of predicting disease was below 5% (models identify them as controls almost for sure).

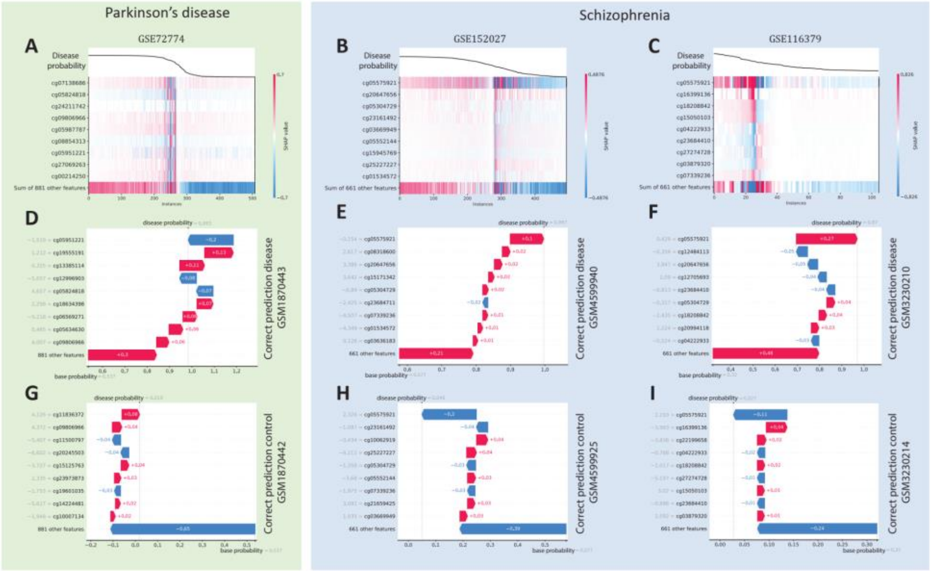

Figure 6. Local explainability based on SHAP values. (A) Black lines show the probability of predicting Parkinson's disease for different participants. Heatmaps in color show the contribution of SHAP values to the probability of predicting Parkinson's disease for different participants and CpG sites for GSE72774. (B) Probability of predicting schizophrenia and heatmaps of contribution for GSE152027. (C) Probability of predicting schizophrenia and heatmaps of contribution for GSE116379. (D) Waterfall plot for participant GSM1870443 with Parkinson's disease, showing the contribution of individual CpG sites to changes in the probability of predicting disease. (E) Waterfall plot for participant GSM4599940 with schizophrenia. (F) Waterfall plot for participant GSM3230210 with schizophrenia. In all cases, the models' confidence is over 97%. (G) Waterfall plot for control participant GSM1870442 showing the contribution of individual CpG sites to the change in probability of predicting disease. (H) Waterfall plot for control participant GSM4599925. (I) Waterfall plot for control participant GSM3230214. In all cases, the probability of predicting disease is below 5%.

Commented [29]: Added A, B, C, D, E, F, G, H, I letters for subplots according to Reviewer's 1 Comment 2.

3. Discussion

3.1. Conclusion

We developed a multifunctional workflow for applying machine learning models to classify cases and controls for different diseases based on DNA methylation data. Specifically, we considered Parkinson's disease and schizophrenia as examples of complex diseases requiring early diagnosis. In addition, for these diseases there are large publicly available whole blood DNA methylation datasets. In this paper, the task of classifying cases and controls based on whole blood DNA methylation data with harmonization, missing value imputation, model dimensionality reduction, and application of XAI approaches is solved for the first time for Parkinson's disease

Commented [30]: "Pipeline" is renamed to "workflow" according to Reviewer's 2 Comment 1.

and schizophrenia. In [106] the problem of pairwise classification of neurodegenerative diseases cases (in particular, for Parkinson's disease and schizophrenia) was solved. The authors did not use methylation levels; instead, they used methylation-derived profile scores as features; however, we do not compare diseases with each other in this work. In [40] the problem of classifying cases and controls for schizophrenia is solved. In that work, instead of methylation levels, other metrics were also used: CoRSIV probes with a polygenic risk score. The paper presents the results of positive predictive values only for cases, not for controls, so it is not possible to directly compare the results.

The first step of the workflow is to harmonize the data according to the chosen reference dataset. We have shown that harmonization works well also when the available datasets were preprocessed using different pipelines and tools, as it often occurs. Harmonization can increase the classification accuracy by up to 20%. This is fully consistent with the original paper, which proposed the harmonization method regRCPqn [49]. When the preprocessing of training and test data is the same, harmonization has almost no effect on the final classification accuracy. It is impossible to guarantee that all new datasets on which the model will be tested will have the same preprocessing. Even with tools such as limma [107], ComBat [108], it may not be possible to remove technical signal when batches are mixed with variables of interest. Applying ComBat to high-throughput data with an uneven study design may actually result in false signals [109]. To solve the classification problem, different models were tested, classical ones (logistic regression and support-vector machine), different gradient-boosted decision trees and deep neural networks. For all models, a hyperparametric search was performed to select the optimal set of parameters. The best results of weighted accuracy were obtained with tree ensembles, which is consistent with other works [110]. For Parkinson's disease, the accuracy of classifying patients and healthy controls was higher than 95%. The accuracy for schizophrenia was much lower, only >70%. Unlike Parkinson's disease, schizophrenia is a complex disease characterized by a variety of different symptoms. A large number of different causes and molecular patterns determining the development of this pathology are being identified. The etiology of schizophrenia is multifactorial and reflects an interaction between genetic vulnerability and environmental influences. Environmental risk factors, such as complications of pregnancy and childbirth, childhood trauma, migration, social isolation, urban life, and substance abuse, either singly or in combination over time, affect the likelihood of an individual developing the disorder. A lot of genes have been previously identified as responsible for the risk of developing this pathology, so the personal molecular landscape is highly individualized and complicated by interactions between genes and the environment. These reasons may be related to the relatively low accuracy for schizophrenia in our study compared to Parkinson's disease [111–113]. Another reason that affects the result may be the number of samples in the training set. The training set for schizophrenia has fewer samples compared to Parkinson's disease. In this case, the data may not be representative enough to fully describe the disease characteristics for the model. Since the best models allow us to obtain the importance values of each feature for classification, we constructed a CpG site ranking for each of the diseases. Based on this ranking, we reduced the dimensionality of the classification models,

Commented [31]: Added information about other papers according to Reviewer's 1 Comment 6

Commented [32]: "Pipeline" is renamed to "workflow" according to Reviewer's 2 Comment 1.

Commented [33]: Added Logistic Regression and SVM according to Reviewer's 2 Comment 5.

Commented [34]: Information on the differences between schizophrenia and Parkinson's disease has been added according to Reviewer's 2 Comment 2.

since many of the most common epigenetic models (such as the epigenetic clocks) contain relatively few CpG sites (up to 1000). To find optimal small models, we performed a series of experiments for an increasing number of features from 10 to 1000. Based on the dependence of the weighted classification accuracy on the number of features, we determined the optimal models, with the accuracy for both diseases changing not much, while the number of features decreased significantly. Optimal small models contain 890 CpG sites for Parkinson's disease and 670 CpG sites for Schizophrenia. Such small models are much less memory-consuming and have better computational performance. For machine learning models (both small and large), missing values are critical. A model trained on a particular set of features must necessarily be tested on the same set. If, for certain reasons (data collection or processing errors, low signal intensities, or other problems), some CpG sites are missing from the new data for testing, imputation methods are applied. The best imputation methods recover almost the same classification accuracy as for the original data. Since machine learning models work as a "black box", special methods must be used to explain exactly how the model makes predictions. Otherwise, the model cannot be trusted and it is impossible to identify the nature of errors. If the predictive capabilities of the model can be explained, it can help to discover complex relationships between biomarkers. The calculation of SHAP values allowed us to obtain both global and local explainability. Globally, the prediction of healthy control or patient with Parkinson's disease is affected by several CpG sites, there are examples of both types of influence, positive and negative. Global explainability for healthy controls and patients with schizophrenia confirmed the strongest influence of one CpG site, cg05575921, while the others had little effect on model prediction. This CpG site has previously been reported to be associated with schizophrenia and post-traumatic stress disorder (PTSD) [85, 114]. Examples of local explainability - specific predictions for certain participants - are also given.

Thus, we propose a methodologically valid and complete approach for classifying healthy people and patients with various diseases, which allows to harmonize DNA methylation data from different sources, impute missing values, reduce dimensionality of the models, and apply explainable artificial intelligence approaches. The proposed algorithm works better for Parkinson's disease than for schizophrenia, which is characterized by a variety of different symptoms. Further work may include expanding the pool of considered diseases, enriching the library of methods at different stages of the workflow. Another future challenge is advancing from explainability to interpretability. The former, currently implemented, uncovers the internal "mechanics" of a system. The yet missing interpretability would predict the outcome of changing the input or algorithmic parameters.

Commented [35]: "Pipeline" is renamed to "workflow" according to Reviewer's 2 Comment 1.

3.2. Limitations

The proposed approach has several limitations relevant at different steps. First, it should be noted that the accuracy gain from harmonization will be limited if the preprocessing of training and test data is the same. Second, the constructed classification models may not be globally optimal in terms of quality metrics, because we are considering a limited number of parameters to vary within a hyperparametric search, each varying within a limited range of values near defaults. Third,

besides choosing the top best features to reduce dimensionality, it might be better to consider different combinations of them. However, in this case, the number of considered models would increase dramatically. Fourth, using schizophrenia as an example, it was shown that if there are some most important CpG sites significantly overcoming the importance of the other, and if they are missed, imputation can not help to improve the result.

4. Methods

4.1. Datasets

We reviewed publicly available whole blood DNA methylation datasets in the GEO repository [115], which include the largest ones from patients with Parkinson's disease and schizophrenia, with at least 50 participants in each group. The following datasets comprise whole blood samples from subjects with Parkinson's disease and healthy controls: GSE145361 [79], GSE111629 [80–82], GSE72774 [80, 81, 83]. The following datasets comprise whole blood samples from subjects with schizophrenia and healthy controls: GSE152027 [84], GSE84727 [84, 85], GSE80417 [84, 85], GSE116379 (only non famine participants) [86]. We remove from the analysis non-CpG probes [116], SNP-related probes [117], multi-hit probes [118], probes on chromosomes X and Y. We consider only common CpGs in train datasets, the number of CpGs in test datasets can be different.The remaining amount of CpGs after all filtration procedures is shown in Table 1.

4.2. Data harmonization

Meta-analysis can be done in different ways: some approaches first analyze different datasets separately and then combine the results into a final estimate; others first combine data from all sets and then analyze the combined data using a single model. The first class of approaches includes aggregated data and two-step meta-analysis of individual participant data (IPD) [119]. The major advantage of these approaches is their relatively low implementation complexity, while the major disadvantage is the need for raw data. This approach often uses not all available data, but only a subset (usually differentially methylated positions, DMPs, or differentially methylated regions, DMRs). The second class of approaches is called single-step IPD meta-analysis. Although single-step IPD approaches are expected to behave similarly to two-step IPD [119], they provide additional flexibility (e.g., no need to start with raw data) and enable comparison between different models [119]. An important assumption of single-step IPD meta-analysis is the comparability of variables measured in different datasets [120], and therefore data harmonization is crucial to ensure that methylation samples of the same type (same tissue, health status, age, gender, etc.) from different datasets can be compared.

We use the approach to data harmonization proposed in [49], a one-step IPD approach for systematically assessing the impact of different preprocessing methods on the meta-analysis. It has been shown that data preprocessing by different algorithms has a significant impact. RegRCPqn (regional regression on correlated probes with quantile normalization) does not require raw idat files and can be applied to datasets with only β-values or M-values available, which is a common

scenario from real life [49]. RCP [121] is a within-array normalization that uses the spatial correlation of DNA methylation at CpG sites to estimate the calibration transformation between type I and type II intensities. The regRCPqn procedure improves the RCP algorithm by including three functions to solve the problem under study. First, it calculates the RCP normalization separately for each type of genomic region (i.e., for CpG belonging to islands, shores, shelves, or open seas) because the distribution of DNA methylation values is different in each of these types of regions [122]. It then performs a quantile normalization between samples, in which CpG values for all samples are normalized separately for each CpG region and for type I and type II probes. Finally, it introduces the possibility of storing the reference distribution and using it to perform quantile normalization of samples from another dataset based on the reference. The reference distribution is calculated separately for each area type and for type I and type II probes. When possible, this distribution is used by regRCPqn to perform normalization based on the reference, again separately for each region and probe type. The dataset with the highest number of participants for each disease is used as the reference, and the others are harmonized relative to it. We consider CpG sites whose distribution of methylation levels does not differ in different train datasets in the control group. To find them, we performed the Mann-Whitney U-test [88] for all CpG sites included in the train datasets for the control group and took CpG sites for which the p-value adjusted according to the Benjamini-Hochberg procedure [123] >0.05. The Mann-Whitney U-test was performed using the scipy package version 1.8.0.

Commented [36]: Added according to Reviewer's 2 Comment 3.

4.3. Classification models

The most common type of data for machine learning and deep learning tasks is tabular data, which comprises samples (rows) with the same set of features (columns). DNA methylation is an example of this type of data. Tabular data, unlike image or speech data, is heterogeneous, resulting in dense numerical and sparse categorical features. In addition, the correlation between features is weaker than the spatial or semantic relationship in image or speech data [124]. Variables can be correlated or independent, and features have no positional information. Consequently, it is necessary to detect and use correlation without relying on spatial information [94, 95]. During the last decade, traditional machine learning methods such as gradient-boosted decision trees (GBDT) [91] have continued to dominate tabular data modeling and have demonstrated better performance than deep learning [110]. GBDT trains a series of weak learners to predict the outcome. In GBDT, the weak learner is a standard decision tree that lacks differentiability. Despite their differences, their performance on many problems is similar [92]. When deep neural networks are applied to tabular data, many problems arise, such as lack of locality, missing values, mixed object types (numeric, ordinal and categorical), lack of prior knowledge about the structure of the data. Tree ensemble algorithms are considered a recommended option for real-world problems with tabular data [91, 92, 125]. The XGBoost algorithm [91] is an extendible gradient boosting tree algorithm that achieves state-of-the-art results on many tabular datasets [126, 127].

We consider the following classification models: Logistic Regression with elastic net penalty [89], Support-Vector Machine [90], XGBoost [91], CatBoost [92], LightGBM [93], TabNet [94],

Commented [37]: Added Logistic Regression and SVM according to Reviewer's 2 Comment 5.

NODE [95]. Despite the name, logistic regression is used to solve the binary classification problem. It is a generalized linear model, showing good results for linearly separable data. Support-vector machine is a supervised learning model whose main goal is to construct a separating manifold. This method allows the use of different kernel functions to achieve the best results. XGBoost (Extreme Gradient Boosting) is a scalable, distributed gradient-boosted decision tree (GBDT) machine learning library. GBDT iteratively trains an ensemble of shallow decision trees, with each iteration using error residuals from the previous model to fit the next model. The final prediction is a weighted sum of all tree predictions. XGBoost has one of the best combinations of prediction performance and processing time. CatBoost is an open-source gradient boosting algorithm, which builds symmetric (balanced) trees. At each step, the leaves of the previous tree are separated by the same condition. A feature-split pair is selected and used for all nodes, which provides the least losses. This balanced tree architecture reduces prediction time and controls overfitting. LightGBM is a fast, distributed, high-performance gradient boosting platform that supports the decision tree algorithm. It splits the tree by leaf with the simplest fit, whereas other boosting algorithms split the tree by depth or by level rather than by leaf. Thus, when growing on an equivalent leaf in LightGBM, a leaf-based algorithm can reduce more losses than a level-based algorithm, and therefore lead to greater accuracy. TabNet is a deep neural network designed to handle tabular data. TabNet inputs raw tabular data with no preprocessing and is trained using gradient descent-based optimization. It uses sequential attention to select features at each decision step, providing interpretability and better learning as the learning capability is used for the most useful features, with instance-specific feature selection. Neural Oblivious Decision Ensembles (NODE) is a deep learning architecture designed to handle tabular data. The NODE architecture generalizes ensembles of oblivious decision trees, but benefits from both end-to-end gradient-based optimization and multilevel hierarchical learning capabilities. All of the above models can handle continuous variables (without categorical ones). For classification, we use only the DNA methylation levels of the different CpG sites, which are continuous variables. Parameter values of the trained models which were found by hyperparametric search can be found in Supplementary Table 1. All models have been trained for 2,000 epochs.

Commented [38]: Added Logistic Regression and SVM according to Reviewer's 2 Comment 5.

Each model was trained on two training datasets and then tested on the remaining independent datasets. Hyperparametric search was used to find optimal parameters for the models. There are a lot of quality metrics for the classification problem: accuracy, precision, recall, f1 score, Cohen's kappa, Matthews correlation coefficient, AUROC, etc. As the main metric, we choose accuracy with weighted averaging to take into account the possible imbalance of the classes. It is calculated according to the formula:

$$\frac{N_{cases}}{N} Accuracy_{cases} + \frac{N_{controls}}{N} Accuracy_{controls}\ , \qquad (1)$$

where N_{cases} is the total number of cases, $N_{controls}$ is total the number of controls, N is the total number of participants. The accuracy for each class is:

$$Accuracy = \frac{TP+TN}{N}, \qquad (2)$$

where TP is the number of true positives and TN is the number of true negatives. Adam optimizer and StepLR scheduler were used for the neural network models [128]. Used versions of software packages for the models: XGBoost 1.5.2, CatBoost 1.0.4, LightGBM 3.3.2, TabNet 3.1.1, PyTorch 1.10.0, PyTorch Lightning 1.6.0.

Commented [39]: Formula for accuracy with weighted averaging is added according to Reviewer's 2 Comment 4.

4.4. Dimensionality reduction

Based on the features ranking, we performed dimensionality reduction of the models. We performed it, leaving only the most important CpGs for solving the classification problem. For this purpose, we built a series of models with different numbers of features. First, for each disease, we choose the top 10 most important features, and a new model is built for them (the type of model is chosen beforehand - it is the best in terms of accuracy for the full data). Then new models are built on the number of features from 10 to 1000 in increments of 10, and for each such model, the weighted classification accuracy is calculated. For all these models hyperparametric search was performed. According to the dependence of weighted classification accuracy on the number of features we chose as optimal the number of features for which the highest weighted classification accuracy is observed for the considered diseases.

4.5. Imputation of missing values

Missing data can be divided into three classes [129]: i) missing completely at random (MCAR) values, if the probability of absence is completely independent of both observed and unobserved variables; ii) missing at random (MAR) values, if the probability of absence is independent of the value itself, but may depend on observed variables; iii) missing not at random (MNAR), if the probability of absence depends on the missing value itself. There is currently no statistical way to determine which category the specific missing data falls into. Assumptions are usually made based on knowledge of the data and the data collection and processing procedure. It is assumed that the missing values represent MCAR/MAR due to random experimental and technology-related errors [53]. It has been shown that missing values lying at the midrange methylation level are more difficult to impute than missing values close to the extremes of the range [54]. This is probably a consequence of the higher variance of methylation values in the middle ranges. Such a scenario could have a profound effect in terms of performance expectations, assuming that many missing values in the data are of the MNAR type and, in particular, lie in the middle range of β values.

In general terms, imputation approaches can be divided into single (SI) and multiple imputation (MI) methods. SI methods replace a missing value with a single acceptable value. MI methods perform multiple SIs and average parameter estimates over multiple imputations to produce a single estimate. Under MCAR/MAR assumptions, the most common imputation methods like mean, median or mode can handle missing data [130]. Such simple imputation methods are used often [131], but they can lead to systematic error or unrealistic results for multivariate datasets. In addition, for large data, this method often performs poorly [132]. The expectation maximization method is an iterative method for handling missing values in numerical

datasets, and the algorithm uses an "impute, estimate, and iterate until convergence" approach. Each iteration involves two steps: expectation and maximization. Expectation estimates the missing values given the observed data, while maximization uses the current estimated values to maximize the probability of all data [133–135]. Besides classical methods, there are approaches to multiple imputation, for example, chained equation for big data [132]. Hot-deck imputation handles missing values by matching missing values with other values in the dataset for several other key variables that have complete values [136, 137]. However, this method does not account for the variability of the missing data. One of the common hot-deck methods is K Nearest Neighbours (KNN) [138]. The KNN algorithm works by classifying the nearest neighbors of missing values and using those neighbors for imputation using a distance measure between instances [139]. Several distance measures can be used for KNN imputation, but Euclidean distance has been shown to provide efficiency and performance [140] and is therefore the most widely used distance measure. However, KNN imputation has weaknesses, such as poor accuracy when imputing variables and introducing false associations where none exists [141]. Another weakness of KNN imputation is that it scans the entire dataset, which increases computation time [142]. However, there are approaches developed in the literature to improve the KNN imputation algorithm [143–149]. All imputation methods that can deal with continuous variables are suitable for imputing DNA methylation data [53]. To study the effect of these methods on classification accuracy, we removed from consideration 100 CpG sites with the highest importance values for each disease and tried to fill them in. We used previously constructed small models for both diseases. Imputation methods were applied by impyute package version 0.0.8.

4.6. Explainable artificial intelligence

Modern machine-learning-based artificial intelligence systems are usually treated as black boxes. However, every decision must be made available for verification by a human expert [150]. One important aspect of model explainability is the ability to verify the system. For example, in healthcare, the use of models that can be interpreted and verified by medical experts is an absolute necessity [151]. Another aspect is to improve the system. The first step to improving the AI system is to understand its weaknesses. Performing weakness analysis on black box models is more difficult than on models that can be interpreted. Furthermore, model interpretability can be useful when comparing different models or architectures [152–154]. It can be argued that the better we understand what models do (and why they sometimes fail), the easier it becomes to improve them [150]. The next important aspect of explainability is the ability to learn from the system: since modern AI systems learn from millions of examples, they can observe patterns in the data that are inaccessible to humans, who can only learn from a limited number of examples [155, 156]. Explainability is also important for other machine learning methods beyond neural networks [152].

One of the taxonomies to classify explanatory methods is global and local methods [64, 65, 150]. Local interpretable methods apply to a single model result; they can explain the reason for a particular prediction or result. In contrast, global methods try to explain the behavior of the model as a whole. Perturbation is the easiest way to analyze the effect of changing input features on the

AI model outputs. This can be accomplished by removing or changing certain input features, running a forward pass, and measuring the difference with the original output data. The input characteristics that most affect the output are evaluated as the most important ones. This is computationally costly, since a direct pass must be run after perturbing each group of input features. Such a perturbation-based approach is Shapley value sampling, which computes approximate Shapley values by taking each input feature for a certain number of times. It is a method from game theory that describes a fair distribution of wins and losses between input functions [157]. As a result, it is not a practical method in its original form, but has led to the development of methods based on game theory, such as Deep SHapley Additive exPlanations (SHAP) [105]. SHAP has an alternative kernel-based approach to estimating Shapley values inspired by local surrogate models. There is also TreeSHAP, an efficient approach to estimating tree models, as well as DeepExplainer, an enhanced version of the DeepLIFT algorithm for deep neural networks. For the constructed portable models, we applied SHAP to obtain global and local explainability. SHAP values were calculated using eponymous package version 0.40.0.

Data availability statement

No new data was generated. Data used in this study are available from the GEO database (accession numbers GSE145361, GSE111629, GSE72774, GSE84727, GSE80417, GSE152027, GSE116379).

Code availability statement

The source code for the analysis workflow presented in the manuscript is publicly available.

Project name: DNAmClassMeta

Project home page: https://github.com/GillianGrayson/DNAmClassMeta

Operating system(s): Platform independent

Programming language: Python

Other requirements: Python 3.8 or higher, pytorch-lightning 1.5.10 or higher, xgboost 1.6.0 or higher, catboost 1.0.5 or higher, lightgbm 3.3.2 or higher, scikit-learn 1.0.2 or higher. All requirements are listed in the requirements.txt file in the project home page.

License: MIT

Commented [40]: "Pipeline" is renamed to "workflow" according to Reviewer's 2 Comment 1.

Commented [41]: Added scikit-learn requirement.

Abbreviations

AI: Artificial Intelligence; CatBoost: Categorical Boosting; DMP: Differentially Methylated Position; DMR: Differentially Methylated Region; DNAm: DNA methylation; EWAS: Epigenome-Wide Association Study; FDR: False Discovery Rate; GBDT: Gradient-Boosted Decision Tree; IPD: Individual Participant Data; KNN: K Nearest Neighbors; LightGBM: Light Gradient Boosting Machine; MAR: Missing At Random; MCAR: Missing Completely At Random; MI: Multiple Imputation; MNAR: Missing Not At Random; NODE: Neural Oblivious

Decision Ensemble; PTSD: Post-Traumatic Stress Disorder; RCP: Regression on Correlated Probes; SHAP: Shapley Additive Explanations; SI: Single Imputation; XAI: Explainable Artificial Intelligence; XGBoost: Extreme Gradient Boosting.

Competing Interests

The authors declare that they have no competing interests.

Funding

The research was supported by the Ministry of Science and Higher Education of the Russian Federation, Grant for Major Research Projects in Priority Areas of Scientific and Technological Development No. 075-15-2020-808, grant recipient: Lobachevsky State University.

Commented [42]: Funding section formatted according to format: Funding body, Program/Award name, award ID, Recipient.

Author's Contributions

Conceptualization: A.K., I.Y., M.G.B., M.I.; Formal analysis: A.K., I.Y.; Methodology: A.K., I.Y., M.G.B.; Software: A.K., I.Y.; Supervision: M.G.B., C.F., M.V., M.I.; Visualization: A.K., I.Y.; Writing – original draft: A.K., I.Y.; Writing – review & editing: A.K., I.Y., M.G.B., C.F., M.V., M.I.

Acknowledgements

The authors acknowledge the use of computational resources provided by the "Lobachevsky" supercomputer.

References

1. Sasaki H, Matsui Y (2008) Epigenetic events in mammalian germ-cell development: reprogramming and beyond. Nat Rev Genet 9:129–140. https://doi.org/10.1038/nrg2295
2. Igarashi J, Muroi S, Kawashima H, Wang X, Shinojima Y, Kitamura E, Oinuma T, Nemoto N, Song F, Ghosh S, Held WA, Nagase H (2008) Quantitative analysis of human tissue-specific differences in methylation. Biochem Biophys Res Commun 376:658–664. https://doi.org/10.1016/j.bbrc.2008.09.044
3. Zemach A, McDaniel IE, Silva P, Zilberman D (2010) Genome-wide evolutionary analysis of eukaryotic DNA methylation. Science 328:916–919. https://doi.org/10.1126/science.1186366
4. Ziller MJ, Gu H, Müller F, Donaghey J, Tsai LT-Y, Kohlbacher O, De Jager PL, Rosen ED, Bennett DA, Bernstein BE, Gnirke A, Meissner A (2013) Charting a dynamic DNA methylation landscape of the human genome. Nature 500:477–481. https://doi.org/10.1038/nature12433
5. Horvath S (2013) DNA methylation age of human tissues and cell types. Genome Biol

14:R115. https://doi.org/10.1186/gb-2013-14-10-r115
6. Orozco LD, Farrell C, Hale C, Rubbi L, Rinaldi A, Civelek M, Pan C, Lam L, Montoya D, Edillor C, Seldin M, Boehnke M, Mohlke KL, Jacobsen S, Kuusisto J, Laakso M, Lusis AJ, Pellegrini M (2018) Epigenome-wide association in adipose tissue from the METSIM cohort. Hum Mol Genet 27:2586. https://doi.org/10.1093/hmg/ddy205
7. Smith ZD, Meissner A (2013) DNA methylation: roles in mammalian development. Nat Rev Genet 14:204–220. https://doi.org/10.1038/nrg3354
8. Lim DHK, Maher ER (2010) Genomic imprinting syndromes and cancer. Adv Genet 70:145–175. https://doi.org/10.1016/B978-0-12-380866-0.60006-X
9. Robertson KD (2005) DNA methylation and human disease. Nat Rev Genet 6:597–610. https://doi.org/10.1038/nrg1655
10. Jones PA (2012) Functions of DNA methylation: islands, start sites, gene bodies and beyond. Nat Rev Genet 13:484–492. https://doi.org/10.1038/nrg3230
11. Jjingo D, Conley AB, Yi SV, Lunyak VV, Jordan IK (2012) On the presence and role of human gene-body DNA methylation. Oncotarget 3:462–474. https://doi.org/10.18632/oncotarget.497
12. Christensen BC, Houseman EA, Marsit CJ, Zheng S, Wrensch MR, Wiemels JL, Nelson HH, Karagas MR, Padbury JF, Bueno R, Sugarbaker DJ, Yeh R-F, Wiencke JK, Kelsey KT (2009) Aging and environmental exposures alter tissue-specific DNA methylation dependent upon CpG island context. PLoS Genet 5:e1000602. https://doi.org/10.1371/journal.pgen.1000602
13. Bell CG, Lowe R, Adams PD, Baccarelli AA, Beck S, Bell JT, Christensen BC, Gladyshev VN, Heijmans BT, Horvath S, Ideker T, Issa J-PJ, Kelsey KT, Marioni RE, Reik W, Relton CL, Schalkwyk LC, Teschendorff AE, Wagner W, Zhang K, Rakyan VK (2019) DNA methylation aging clocks: challenges and recommendations. Genome Biol 20:249. https://doi.org/10.1186/s13059-019-1824-y
14. Rakyan VK, Down TA, Balding DJ, Beck S (2011) Epigenome-wide association studies for common human diseases. Nat Rev Genet 12:529–541. https://doi.org/10.1038/nrg3000
15. Liu D, Zhao L, Wang Z, Zhou X, Fan X, Li Y, Xu J, Hu S, Niu M, Song X, Li Y, Zuo L, Lei C, Zhang M, Tang G, Huang M, Zhang N, Duan L, Lv H, Zhang M, Li J, Xu L, Kong F, Feng R, Jiang Y (2019) EWASdb: epigenome-wide association study database. Nucleic Acids Res 47:D989–D993. https://doi.org/10.1093/nar/gky942
16. Birney E, Smith GD, Greally JM (2016) Epigenome-wide Association Studies and the Interpretation of Disease -Omics. PLoS Genet 12:e1006105. https://doi.org/10.1371/journal.pgen.1006105
17. Moran S, Arribas C, Esteller M (2016) Validation of a DNA methylation microarray for 850,000 CpG sites of the human genome enriched in enhancer sequences. Epigenomics 8:389–399. https://doi.org/10.2217/epi.15.114
18. Bibikova M, Lin Z, Zhou L, Chudin E, Garcia EW, Wu B, Doucet D, Thomas NJ, Wang Y, Vollmer E, Goldmann T, Seifart C, Jiang W, Barker DL, Chee MS, Floros J, Fan J-B (2006) High-throughput DNA methylation profiling using universal bead arrays. Genome Res 16:383–393. https://doi.org/10.1101/gr.4410706
19. Irizarry RA, Ladd-Acosta C, Carvalho B, Wu H, Brandenburg SA, Jeddeloh JA, Wen B, Feinberg AP (2008) Comprehensive high-throughput arrays for relative methylation (CHARM). Genome Res 18:780–790. https://doi.org/10.1101/gr.7301508
20. Du P, Zhang X, Huang C-C, Jafari N, Kibbe WA, Hou L, Lin SM (2010) Comparison of

Beta-value and M-value methods for quantifying methylation levels by microarray analysis. BMC Bioinformatics 11:587. https://doi.org/10.1186/1471-2105-11-587
21. Tian T, Wan J, Song Q, Wei Z (2019) Clustering single-cell RNA-seq data with a model-based deep learning approach. Nat Mach Intell 1:191–198. https://doi.org/10.1038/s42256-019-0037-0
22. Lopez R, Regier J, Cole MB, Jordan MI, Yosef N (2018) Deep generative modeling for single-cell transcriptomics. Nat Methods 15:1053–1058. https://doi.org/10.1038/s41592-018-0229-2
23. Way GP, Greene CS (2018) Extracting a biologically relevant latent space from cancer transcriptomes with variational autoencoders. Pac Symp Biocomput 23:80–91
24. Titus AJ, Wilkins OM, Bobak CA, Christensen BC (2018) Unsupervised deep learning with variational autoencoders applied to breast tumor genome-wide DNA methylation data with biologic feature extraction. Bioinformatics
25. Ching T, Himmelstein DS, Beaulieu-Jones BK, Kalinin AA, Do BT, Way GP, Ferrero E, Agapow P-M, Zietz M, Hoffman MM, Xie W, Rosen GL, Lengerich BJ, Israeli J, Lanchantin J, Woloszynek S, Carpenter AE, Shrikumar A, Xu J, Cofer EM, Lavender CA, Turaga SC, Alexandari AM, Lu Z, Harris DJ, DeCaprio D, Qi Y, Kundaje A, Peng Y, Wiley LK, Segler MHS, Boca SM, Swamidass SJ, Huang A, Gitter A, Greene CS (2018) Opportunities and obstacles for deep learning in biology and medicine. J R Soc Interface 15:20170387. https://doi.org/10.1098/rsif.2017.0387
26. Levy JJ, Titus AJ, Petersen CL, Chen Y, Salas LA, Christensen BC (2020) MethylNet: an automated and modular deep learning approach for DNA methylation analysis. BMC Bioinformatics 21:108. https://doi.org/10.1186/s12859-020-3443-8
27. The Cancer Genome Atlas Research Network, Weinstein JN, Collisson EA, Mills GB, Shaw KRM, Ozenberger BA, Ellrott K, Shmulevich I, Sander C, Stuart JM (2013) The Cancer Genome Atlas Pan-Cancer analysis project. Nat Genet 45:1113–1120. https://doi.org/10.1038/ng.2764
28. Ding W, Chen G, Shi T (2019) Integrative analysis identifies potential DNA methylation biomarkers for pan-cancer diagnosis and prognosis. Epigenetics 14:67–80. https://doi.org/10.1080/15592294.2019.1568178
29. Celli F, Cumbo F, Weitschek E (2018) Classification of Large DNA Methylation Datasets for Identifying Cancer Drivers. Big Data Research 13:21–28. https://doi.org/10.1016/j.bdr.2018.02.005
30. Ma B, Meng F, Yan G, Yan H, Chai B, Song F (2020) Diagnostic classification of cancers using extreme gradient boosting algorithm and multi-omics data. Computers in Biology and Medicine 121:103761. https://doi.org/10.1016/j.compbiomed.2020.103761
31. List M, Hauschild A-C, Tan Q, Kruse TA, Baumbach J, Batra R (2014) Classification of Breast Cancer Subtypes by combining Gene Expression and DNA Methylation Data. Journal of Integrative Bioinformatics 11:1–14. https://doi.org/10.1515/jib-2014-236
32. Dong R, Yang X, Zhang X, Gao P, Ke A, Sun H, Zhou J, Fan J, Cai J, Shi G (2019) Predicting overall survival of patients with hepatocellular carcinoma using a three-category method based on DNA methylation and machine learning. J Cell Mol Med 23:3369–3374. https://doi.org/10.1111/jcmm.14231
33. Hao X, Luo H, Krawczyk M, Wei W, Wang W, Wang J, Flagg K, Hou J, Zhang H, Yi S, Jafari M, Lin D, Chung C, Caughey BA, Li G, Dhar D, Shi W, Zheng L, Hou R, Zhu J, Zhao L, Fu X, Zhang E, Zhang C, Zhu J-K, Karin M, Xu R-H, Zhang K (2017) DNA

methylation markers for diagnosis and prognosis of common cancers. Proc Natl Acad Sci USA 114:7414–7419. https://doi.org/10.1073/pnas.1703577114
34. Jurmeister P, Bockmayr M, Seegerer P, Bockmayr T, Treue D, Montavon G, Vollbrecht C, Arnold A, Teichmann D, Bressem K, Schüller U, von Laffert M, Müller K-R, Capper D, Klauschen F (2019) Machine learning analysis of DNA methylation profiles distinguishes primary lung squamous cell carcinomas from head and neck metastases. Sci Transl Med 11:eaaw8513. https://doi.org/10.1126/scitranslmed.aaw8513
35. Wajed SA, Laird PW, DeMeester TR (2001) DNA Methylation: An Alternative Pathway to Cancer: Annals of Surgery 234:10–20. https://doi.org/10.1097/00000658-200107000-00003
36. Bollepalli S, Korhonen T, Kaprio J, Anders S, Ollikainen M (2019) EpiSmokEr: a robust classifier to determine smoking status from DNA methylation data. Epigenomics 11:1469–1486. https://doi.org/10.2217/epi-2019-0206
37. Lee Y-C, Christensen JJ, Parnell LD, Smith CE, Shao J, McKeown NM, Ordovás JM, Lai C-Q (2022) Using Machine Learning to Predict Obesity Based on Genome-Wide and Epigenome-Wide Gene–Gene and Gene–Diet Interactions. Front Genet 12:783845. https://doi.org/10.3389/fgene.2021.783845
38. Aref-Eshghi E, Rodenhiser DI, Schenkel LC, Lin H, Skinner C, Ainsworth P, Paré G, Hood RL, Bulman DE, Kernohan KD, Care4Rare Canada Consortium, Boycott KM, Campeau PM, Schwartz C, Sadikovic B (2018) Genomic DNA Methylation Signatures Enable Concurrent Diagnosis and Clinical Genetic Variant Classification in Neurodevelopmental Syndromes. Am J Hum Genet 102:156–174. https://doi.org/10.1016/j.ajhg.2017.12.008
39. Dogan MV, Grumbach IM, Michaelson JJ, Philibert RA (2018) Integrated genetic and epigenetic prediction of coronary heart disease in the Framingham Heart Study. PLoS One 13:e0190549. https://doi.org/10.1371/journal.pone.0190549
40. Gunasekara CJ, Hannon E, MacKay H, Coarfa C, McQuillin A, Clair DSt, Mill J, Waterland RA (2021) A machine learning case–control classifier for schizophrenia based on DNA methylation in blood. Transl Psychiatry 11:412. https://doi.org/10.1038/s41398-021-01496-3
41. Jabari S, Kobow K, Pieper T, Hartlieb T, Kudernatsch M, Polster T, Bien CG, Kalbhenn T, Simon M, Hamer H, Rössler K, Feucht M, Mühlebner A, Najm I, Peixoto-Santos JE, Gil-Nagel A, Delgado RT, Aledo-Serrano A, Hou Y, Coras R, von Deimling A, Blümcke I (2022) DNA methylation-based classification of malformations of cortical development in the human brain. Acta Neuropathol 143:93–104. https://doi.org/10.1007/s00401-021-02386-0
42. Jo T, Nho K, Bice P, Saykin AJ, for the Alzheimer's Neuroimaging Initiative (2021) Deep learning-based identification of genetic variants: Application to Alzheimer's disease classification. Genetic and Genomic Medicine
43. Haghshenas S, Bhai P, Aref-Eshghi E, Sadikovic B (2020) Diagnostic Utility of Genome-Wide DNA Methylation Analysis in Mendelian Neurodevelopmental Disorders. IJMS 21:9303. https://doi.org/10.3390/ijms21239303
44. Xiong Z, Zhang X, Zhang M, Cao B (2020) Predicting Features of Human Mental Disorders through Methylation Profile and Machine Learning Models. In: 2020 2nd International Conference on Machine Learning, Big Data and Business Intelligence (MLBDBI). IEEE, Taiyuan, China, pp 67–75

45. Luo X, Wei Y (2019) Batch Effects Correction with Unknown Subtypes. Journal of the American Statistical Association 114:581–594. https://doi.org/10.1080/01621459.2018.1497494
46. Leek JT, Scharpf RB, Bravo HC, Simcha D, Langmead B, Johnson WE, Geman D, Baggerly K, Irizarry RA (2010) Tackling the widespread and critical impact of batch effects in high-throughput data. Nat Rev Genet 11:733–739. https://doi.org/10.1038/nrg2825
47. Perrier F, Novoloaca A, Ambatipudi S, Baglietto L, Ghantous A, Perduca V, Barrdahl M, Harlid S, Ong KK, Cardona A, Polidoro S, Nøst TH, Overvad K, Omichessan H, Dollé M, Bamia C, Huerta JM, Vineis P, Herceg Z, Romieu I, Ferrari P (2018) Identifying and correcting epigenetics measurements for systematic sources of variation. Clin Epigenet 10:38. https://doi.org/10.1186/s13148-018-0471-6
48. Zindler T, Frieling H, Neyazi A, Bleich S, Friedel E (2020) Simulating ComBat: how batch correction can lead to the systematic introduction of false positive results in DNA methylation microarray studies. BMC Bioinformatics 21:271. https://doi.org/10.1186/s12859-020-03559-6
49. Sala C, Di Lena P, Fernandes Durso D, Prodi A, Castellani G, Nardini C (2020) Evaluation of pre-processing on the meta-analysis of DNA methylation data from the Illumina HumanMethylation450 BeadChip platform. PLoS One 15:e0229763. https://doi.org/10.1371/journal.pone.0229763
50. Garagnani P, Bacalini MG, Pirazzini C, Gori D, Giuliani C, Mari D, Di Blasio AM, Gentilini D, Vitale G, Collino S, Rezzi S, Castellani G, Capri M, Salvioli S, Franceschi C (2012) Methylation of ELOVL2 gene as a new epigenetic marker of age. Aging Cell 11:1132–1134. https://doi.org/10.1111/acel.12005
51. Hannum G, Guinney J, Zhao L, Zhang L, Hughes G, Sadda S, Klotzle B, Bibikova M, Fan J-B, Gao Y, Deconde R, Chen M, Rajapakse I, Friend S, Ideker T, Zhang K (2013) Genome-wide Methylation Profiles Reveal Quantitative Views of Human Aging Rates. Molecular Cell 49:359–367. https://doi.org/10.1016/j.molcel.2012.10.016
52. Weidner C, Lin Q, Koch C, Eisele L, Beier F, Ziegler P, Bauerschlag D, Jöckel K-H, Erbel R, Mühleisen T, Zenke M, Brümmendorf T, Wagner W (2014) Aging of blood can be tracked by DNA methylation changes at just three CpG sites. Genome Biol 15:R24. https://doi.org/10.1186/gb-2014-15-2-r24
53. Di Lena P, Sala C, Prodi A, Nardini C (2019) Missing value estimation methods for DNA methylation data. Bioinformatics 35:3786–3793. https://doi.org/10.1093/bioinformatics/btz134
54. Lena PD, Sala C, Prodi A, Nardini C (2020) Methylation data imputation performances under different representations and missingness patterns. BMC Bioinformatics 21:268. https://doi.org/10.1186/s12859-020-03592-5
55. Venkat N (2018) The Curse of Dimensionality: Inside Out. https://doi.org/10.13140/RG.2.2.29631.36006
56. Levine ME, Lu AT, Quach A, Chen BH, Assimes TL, Bandinelli S, Hou L, Baccarelli AA, Stewart JD, Li Y, Whitsel EA, Wilson JG, Reiner AP, Aviv A, Lohman K, Liu Y, Ferrucci L, Horvath S (2018) An epigenetic biomarker of aging for lifespan and healthspan. Aging 10:573–591. https://doi.org/10.18632/aging.101414
57. Lu AT, Quach A, Wilson JG, Reiner AP, Aviv A, Raj K, Hou L, Baccarelli AA, Li Y, Stewart JD, Whitsel EA, Assimes TL, Ferrucci L, Horvath S (2019) DNA methylation

GrimAge strongly predicts lifespan and healthspan. Aging 11:303–327. https://doi.org/10.18632/aging.101684
58. Kurdyukov S, Bullock M (2016) DNA Methylation Analysis: Choosing the Right Method. Biology (Basel) 5:E3. https://doi.org/10.3390/biology5010003
59. He K, Zhang X, Ren S, Sun J (2016) Deep Residual Learning for Image Recognition. In: 2016 IEEE Conference on Computer Vision and Pattern Recognition (CVPR). IEEE, Las Vegas, NV, USA, pp 770–778
60. Cho K, van Merrienboer B, Gulcehre C, Bahdanau D, Bougares F, Schwenk H, Bengio Y (2014) Learning Phrase Representations using RNN Encoder–Decoder for Statistical Machine Translation. In: Proceedings of the 2014 Conference on Empirical Methods in Natural Language Processing (EMNLP). Association for Computational Linguistics, Doha, Qatar, pp 1724–1734
61. Deng L, Hinton G, Kingsbury B (2013) New types of deep neural network learning for speech recognition and related applications: an overview. In: 2013 IEEE International Conference on Acoustics, Speech and Signal Processing. IEEE, Vancouver, BC, Canada, pp 8599–8603
62. Baldi P (2018) Deep Learning in Biomedical Data Science. Annu Rev Biomed Data Sci 1:181–205. https://doi.org/10.1146/annurev-biodatasci-080917-013343
63. Galkin F, Mamoshina P, Kochetov K, Sidorenko D, Zhavoronkov A (2021) DeepMAge: A Methylation Aging Clock Developed with Deep Learning. Aging and disease 12:1252. https://doi.org/10.14336/AD.2020.1202
64. Baehrens D, Schroeter T, Harmeling S, Kawanabe M, Hansen K, Müller K-R (2010) How to Explain Individual Classification Decisions. J Mach Learn Res 11:1803–1831
65. Simonyan K, Vedaldi A, Zisserman A (2014) Deep Inside Convolutional Networks: Visualising Image Classification Models and Saliency Maps. arXiv:13126034 [cs]
66. Zeiler MD, Fergus R (2014) Visualizing and Understanding Convolutional Networks. In: Fleet D, Pajdla T, Schiele B, Tuytelaars T (eds) Computer Vision – ECCV 2014. Springer International Publishing, Cham, pp 818–833
67. Bach S, Binder A, Montavon G, Klauschen F, Müller K-R, Samek W (2015) On Pixel-Wise Explanations for Non-Linear Classifier Decisions by Layer-Wise Relevance Propagation. PLoS ONE 10:e0130140. https://doi.org/10.1371/journal.pone.0130140
68. Shrikumar A, Greenside P, Shcherbina A, Kundaje A (2017) Not Just a Black Box: Learning Important Features Through Propagating Activation Differences. arXiv:160501713 [cs]
69. Mahendran A, Vedaldi A (2016) Visualizing Deep Convolutional Neural Networks Using Natural Pre-images. Int J Comput Vis 120:233–255. https://doi.org/10.1007/s11263-016-0911-8
70. Lipton ZC (2017) The Mythos of Model Interpretability. arXiv:160603490 [cs, stat]
71. Ribeiro MT, Singh S, Guestrin C (2016) “Why Should I Trust You?”: Explaining the Predictions of Any Classifier. arXiv:160204938 [cs, stat]
72. Zintgraf LM, Cohen TS, Adel T, Welling M (2017) Visualizing Deep Neural Network Decisions: Prediction Difference Analysis. arXiv:170204595 [cs]
73. Doshi-Velez F, Kim B (2017) Towards A Rigorous Science of Interpretable Machine Learning. arXiv:170208608 [cs, stat]
74. Montavon G, Samek W, Müller K-R (2018) Methods for interpreting and understanding deep neural networks. Digital Signal Processing 73:1–15.

https://doi.org/10.1016/j.dsp.2017.10.011
75. Mahendran A, Vedaldi A (2014) Understanding Deep Image Representations by Inverting Them. arXiv:14120035 [cs]
76. Nguyen A, Yosinski J, Clune J (2016) Multifaceted Feature Visualization: Uncovering the Different Types of Features Learned By Each Neuron in Deep Neural Networks. arXiv:160203616 [cs]
77. Landecker W, Thomure MD, Bettencourt LMA, Mitchell M, Kenyon GT, Brumby SP (2013) Interpreting individual classifications of hierarchical networks. In: 2013 IEEE Symposium on Computational Intelligence and Data Mining (CIDM). pp 32–38
78. Montavon G, Lapuschkin S, Binder A, Samek W, Müller K-R (2017) Explaining nonlinear classification decisions with deep Taylor decomposition. Pattern Recogn 65:211–222. https://doi.org/10.1016/j.patcog.2016.11.008
79. Vallerga CL, Zhang F, Fowdar J, McRae AF, Qi T, Nabais MF, Zhang Q, Kassam I, Henders AK, Wallace L, Montgomery G, Chuang Y-H, Horvath S, Ritz B, Halliday G, Hickie I, Kwok JB, Pearson J, Pitcher T, Kennedy M, Bentley SR, Silburn PA, Yang J, Wray NR, Lewis SJG, Anderson T, Dalrymple-Alford J, Mellick GD, Visscher PM, Gratten J (2020) Analysis of DNA methylation associates the cystine-glutamate antiporter SLC7A11 with risk of Parkinson’s disease. Nat Commun 11:1238. https://doi.org/10.1038/s41467-020-15065-7
80. Chuang Y-H, Paul KC, Bronstein JM, Bordelon Y, Horvath S, Ritz B (2017) Parkinson’s disease is associated with DNA methylation levels in human blood and saliva. Genome Med 9:76. https://doi.org/10.1186/s13073-017-0466-5
81. Horvath S, Ritz BR (2015) Increased epigenetic age and granulocyte counts in the blood of Parkinson’s disease patients. Aging (Albany NY) 7:1130–1142. https://doi.org/10.18632/aging.100859
82. Chuang Y-H, Lu AT, Paul KC, Folle AD, Bronstein JM, Bordelon Y, Horvath S, Ritz B (2019) Longitudinal Epigenome-Wide Methylation Study of Cognitive Decline and Motor Progression in Parkinson’s Disease. J Parkinsons Dis 9:389–400. https://doi.org/10.3233/JPD-181549
83. Paul KC, Binder AM, Horvath S, Kusters C, Yan Q, Rosario ID, Yu Y, Bronstein J, Ritz B (2021) Accelerated hematopoietic mitotic aging measured by DNA methylation, blood cell lineage, and Parkinson’s disease. BMC Genomics 22:696. https://doi.org/10.1186/s12864-021-08009-y
84. Hannon E, Dempster EL, Mansell G, Burrage J, Bass N, Bohlken MM, Corvin A, Curtis CJ, Dempster D, Di Forti M, Dinan TG, Donohoe G, Gaughran F, Gill M, Gillespie A, Gunasinghe C, Hulshoff HE, Hultman CM, Johansson V, Kahn RS, Kaprio J, Kenis G, Kowalec K, MacCabe J, McDonald C, McQuillin A, Morris DW, Murphy KC, Mustard CJ, Nenadic I, O’Donovan MC, Quattrone D, Richards AL, Rutten BP, St Clair D, Therman S, Toulopoulou T, Van Os J, Waddington JL, Wellcome Trust Case Control Consortium (WTCCC), CRESTAR consortium, Sullivan P, Vassos E, Breen G, Collier DA, Murray RM, Schalkwyk LS, Mill J (2021) DNA methylation meta-analysis reveals cellular alterations in psychosis and markers of treatment-resistant schizophrenia. Elife 10:e58430. https://doi.org/10.7554/eLife.58430
85. Hannon E, Dempster E, Viana J, Burrage J, Smith AR, Macdonald R, St Clair D, Mustard C, Breen G, Therman S, Kaprio J, Toulopoulou T, Hulshoff Pol HE, Bohlken MM, Kahn RS, Nenadic I, Hultman CM, Murray RM, Collier DA, Bass N, Gurling H, McQuillin A,

Schalkwyk L, Mill J (2016) An integrated genetic-epigenetic analysis of schizophrenia: evidence for co-localization of genetic associations and differential DNA methylation. Genome Biol 17:176. https://doi.org/10.1186/s13059-016-1041-x
86. Boks MP, Houtepen LC, Xu Z, He Y, Ursini G, Maihofer AX, Rajarajan P, Yu Q, Xu H, Wu Y, Wang S, Shi JP, Hulshoff Pol HE, Strengman E, Rutten BPF, Jaffe AE, Kleinman JE, Baker DG, Hol EM, Akbarian S, Nievergelt CM, De Witte LD, Vinkers CH, Weinberger DR, Yu J, Kahn RS (2018) Genetic vulnerability to DUSP22 promoter hypermethylation is involved in the relation between in utero famine exposure and schizophrenia. NPJ Schizophr 4:16. https://doi.org/10.1038/s41537-018-0058-4
87. Rauschert S, Raubenheimer K, Melton PE, Huang RC (2020) Machine learning and clinical epigenetics: a review of challenges for diagnosis and classification. Clin Epigenet 12:51. https://doi.org/10.1186/s13148-020-00842-4
88. Mann HB, Whitney DR (1947) On a Test of Whether one of Two Random Variables is Stochastically Larger than the Other. Ann Math Statist 18:50–60. https://doi.org/10.1214/aoms/1177730491
89. Cox DR (1958) The Regression Analysis of Binary Sequences. Journal of the Royal Statistical Society Series B (Methodological) 20:215–242
90. Cortes C, Vapnik V (1995) Support-vector networks. Mach Learn 20:273–297. https://doi.org/10.1007/BF00994018
91. Chen T, Guestrin C (2016) XGBoost: A Scalable Tree Boosting System. In: Proceedings of the 22nd ACM SIGKDD International Conference on Knowledge Discovery and Data Mining. ACM, San Francisco California USA, pp 785–794
92. Prokhorenkova L, Gusev G, Vorobev A, Dorogush AV, Gulin A (2018) CatBoost: unbiased boosting with categorical features. In: Bengio S, Wallach H, Larochelle H, Grauman K, Cesa-Bianchi N, Garnett R (eds) Advances in Neural Information Processing Systems. Curran Associates, Inc.
93. Ke G, Meng Q, Finley T, Wang T, Chen W, Ma W, Ye Q, Liu T-Y (2017) LightGBM: A Highly Efficient Gradient Boosting Decision Tree. Long Beach, CA, USA
94. Arik SO, Pfister T (2020) TabNet: Attentive Interpretable Tabular Learning. arXiv:190807442 [cs, stat]
95. Popov S, Morozov S, Babenko A (2019) Neural Oblivious Decision Ensembles for Deep Learning on Tabular Data. arXiv:190906312 [cs, stat]
96. Henderson-Smith A, Fisch KM, Hua J, Liu G, Ricciardelli E, Jepsen K, Huentelman M, Stalberg G, Edland SD, Scherzer CR, Dunckley T, Desplats P (2019) DNA methylation changes associated with Parkinson’s disease progression: outcomes from the first longitudinal genome-wide methylation analysis in blood. Epigenetics 14:365–382. https://doi.org/10.1080/15592294.2019.1588682
97. Kaut O, Schmitt I, Tost J, Busato F, Liu Y, Hofmann P, Witt SH, Rietschel M, Fröhlich H, Wüllner U (2017) Epigenome-wide DNA methylation analysis in siblings and monozygotic twins discordant for sporadic Parkinson’s disease revealed different epigenetic patterns in peripheral blood mononuclear cells. Neurogenetics 18:7–22. https://doi.org/10.1007/s10048-016-0497-x
98. Walton E, Hass J, Liu J, Roffman JL, Bernardoni F, Roessner V, Kirsch M, Schackert G, Calhoun V, Ehrlich S (2016) Correspondence of DNA Methylation Between Blood and Brain Tissue and Its Application to Schizophrenia Research. SCHBUL 42:406–414. https://doi.org/10.1093/schbul/sbv074

99. Hoang HT, Schlager MA, Carter AP, Bullock SL (2017) DYNC1H1 mutations associated with neurological diseases compromise processivity of dynein–dynactin–cargo adaptor complexes. Proc Natl Acad Sci USA 114:. https://doi.org/10.1073/pnas.1620141114
100. Chen X-J, Xu H, Cooper HM, Liu Y (2014) Cytoplasmic dynein: a key player in neurodegenerative and neurodevelopmental diseases. Sci China Life Sci 57:372–377. https://doi.org/10.1007/s11427-014-4639-9
101. Ma Y, Li J, Xu Y, Wang Y, Yao Y, Liu Q, Wang M, Zhao X, Fan R, Chen J, Zhang B, Cai Z, Han H, Yang Z, Yuan W, Zhong Y, Chen X, Ma JZ, Payne TJ, Xu Y, Ning Y, Cui W, Li MD (2020) Identification of 34 genes conferring genetic and pharmacological risk for the comorbidity of schizophrenia and smoking behaviors. Aging (Albany NY) 12:2169–2225. https://doi.org/10.18632/aging.102735
102. Peykov S, Berkel S, Schoen M, Weiss K, Degenhardt F, Strohmaier J, Weiss B, Proepper C, Schratt G, Nöthen MM, Boeckers TM, Rietschel M, Rappold GA (2015) Identification and functional characterization of rare SHANK2 variants in schizophrenia. Mol Psychiatry 20:1489–1498. https://doi.org/10.1038/mp.2014.172
103. Chen X, Long F, Cai B, Chen X, Chen G (2017) A novel relationship for schizophrenia, bipolar and major depressive disorder Part 5: a hint from chromosome 5 high density association screen. Am J Transl Res 9:2473–2491
104. Hindley G, Bahrami S, Steen NE, O'Connell KS, Frei O, Shadrin A, Bettella F, Rødevand L, Fan CC, Dale AM, Djurovic S, Smeland OB, Andreassen OA (2021) Characterising the shared genetic determinants of bipolar disorder, schizophrenia and risk-taking. Transl Psychiatry 11:466. https://doi.org/10.1038/s41398-021-01576-4
105. Chen H, Lundberg S, Lee S-I (2021) Explaining Models by Propagating Shapley Values of Local Components. In: Shaban-Nejad A, Michalowski M, Buckeridge DL (eds) Explainable AI in Healthcare and Medicine: Building a Culture of Transparency and Accountability. Springer International Publishing, Cham, pp 261–270
106. the Australian Imaging Biomarkers and Lifestyle study, the Alzheimer's Disease Neuroimaging Initiative, Nabais MF, Laws SM, Lin T, Vallerga CL, Armstrong NJ, Blair IP, Kwok JB, Mather KA, Mellick GD, Sachdev PS, Wallace L, Henders AK, Zwamborn RAJ, Hop PJ, Lunnon K, Pishva E, Roubroeks JAY, Soininen H, Tsolaki M, Mecocci P, Lovestone S, Kłoszewska I, Vellas B, Furlong S, Garton FC, Henderson RD, Mathers S, McCombe PA, Needham M, Ngo ST, Nicholson G, Pamphlett R, Rowe DB, Steyn FJ, Williams KL, Anderson TJ, Bentley SR, Dalrymple-Alford J, Fowder J, Gratten J, Halliday G, Hickie IB, Kennedy M, Lewis SJG, Montgomery GW, Pearson J, Pitcher TL, Silburn P, Zhang F, Visscher PM, Yang J, Stevenson AJ, Hillary RF, Marioni RE, Harris SE, Deary IJ, Jones AR, Shatunov A, Iacoangeli A, van Rheenen W, van den Berg LH, Shaw PJ, Shaw CE, Morrison KE, Al-Chalabi A, Veldink JH, Hannon E, Mill J, Wray NR, McRae AF (2021) Meta-analysis of genome-wide DNA methylation identifies shared associations across neurodegenerative disorders. Genome Biol 22:90. https://doi.org/10.1186/s13059-021-02275-5
107. Smyth GK, Speed T (2003) Normalization of cDNA microarray data. Methods 31:265–273. https://doi.org/10.1016/s1046-2023(03)00155-5
108. Johnson WE, Li C, Rabinovic A (2007) Adjusting batch effects in microarray expression data using empirical Bayes methods. Biostatistics 8:118–127. https://doi.org/10.1093/biostatistics/kxj037
109. Price EM, Robinson WP (2018) Adjusting for Batch Effects in DNA Methylation

Microarray Data, a Lesson Learned. Front Genet 9:83. https://doi.org/10.3389/fgene.2018.00083
110. Shwartz-Ziv R, Armon A (2022) Tabular data: Deep learning is not all you need. Information Fusion 81:84–90. https://doi.org/10.1016/j.inffus.2021.11.011
111. Stilo SA, Murray RM (2019) Non-Genetic Factors in Schizophrenia. Curr Psychiatry Rep 21:100. https://doi.org/10.1007/s11920-019-1091-3
112. Häfner H, an der Heiden W (1997) Epidemiology of Schizophrenia. Can J Psychiatry 42:139–151. https://doi.org/10.1177/070674379704200204
113. Khan Z, Martin-Montañez E, Muly E (2013) Schizophrenia: Causes and Treatments. CPD 19:6451–6461. https://doi.org/10.2174/1381612811319360006
114. INTRuST Clinical Consortium, VA Mid-Atlantic MIRECC Workgroup, PGC PTSD Epigenetics Workgroup, Smith AK, Ratanatharathorn A, Maihofer AX, Naviaux RK, Aiello AE, Amstadter AB, Ashley-Koch AE, Baker DG, Beckham JC, Boks MP, Bromet E, Dennis M, Galea S, Garrett ME, Geuze E, Guffanti G, Hauser MA, Katrinli S, Kilaru V, Kessler RC, Kimbrel NA, Koenen KC, Kuan P-F, Li K, Logue MW, Lori A, Luft BJ, Miller MW, Naviaux JC, Nugent NR, Qin X, Ressler KJ, Risbrough VB, Rutten BPF, Stein MB, Ursano RJ, Vermetten E, Vinkers CH, Wang L, Youssef NA, Uddin M, Nievergelt CM (2020) Epigenome-wide meta-analysis of PTSD across 10 military and civilian cohorts identifies methylation changes in AHRR. Nat Commun 11:5965. https://doi.org/10.1038/s41467-020-19615-x
115. Barrett T, Troup DB, Wilhite SE, Ledoux P, Rudnev D, Evangelista C, Kim IF, Soboleva A, Tomashevsky M, Marshall KA, Phillippy KH, Sherman PM, Muertter RN, Edgar R (2009) NCBI GEO: archive for high-throughput functional genomic data. Nucleic Acids Res 37:D885-890. https://doi.org/10.1093/nar/gkn764
116. McCartney DL, Walker RM, Morris SW, McIntosh AM, Porteous DJ, Evans KL (2016) Identification of polymorphic and off-target probe binding sites on the Illumina Infinium MethylationEPIC BeadChip. Genomics Data 9:22–24. https://doi.org/10.1016/j.gdata.2016.05.012
117. Zhou W, Laird PW, Shen H (2017) Comprehensive characterization, annotation and innovative use of Infinium DNA methylation BeadChip probes. Nucleic Acids Res 45:e22. https://doi.org/10.1093/nar/gkw967
118. Nordlund J, Bäcklin CL, Wahlberg P, Busche S, Berglund EC, Eloranta M-L, Flaegstad T, Forestier E, Frost B-M, Harila-Saari A, Heyman M, Jónsson ÓG, Larsson R, Palle J, Rönnblom L, Schmiegelow K, Sinnett D, Söderhäll S, Pastinen T, Gustafsson MG, Lönnerholm G, Syvänen A-C (2013) Genome-wide signatures of differential DNA methylation in pediatric acute lymphoblastic leukemia. Genome Biol 14:r105. https://doi.org/10.1186/gb-2013-14-9-r105
119. Stewart GB, Altman DG, Askie LM, Duley L, Simmonds MC, Stewart LA (2012) Statistical analysis of individual participant data meta-analyses: a comparison of methods and recommendations for practice. PLoS One 7:e46042. https://doi.org/10.1371/journal.pone.0046042
120. Smith-Warner SA, Spiegelman D, Ritz J, Albanes D, Beeson WL, Bernstein L, Berrino F, van den Brandt PA, Buring JE, Cho E, Colditz GA, Folsom AR, Freudenheim JL, Giovannucci E, Goldbohm RA, Graham S, Harnack L, Horn-Ross PL, Krogh V, Leitzmann MF, McCullough ML, Miller AB, Rodriguez C, Rohan TE, Schatzkin A, Shore R, Virtanen M, Willett WC, Wolk A, Zeleniuch-Jacquotte A, Zhang SM, Hunter DJ

(2006) Methods for pooling results of epidemiologic studies: the Pooling Project of Prospective Studies of Diet and Cancer. Am J Epidemiol 163:1053–1064. https://doi.org/10.1093/aje/kwj127
121. Niu L, Xu Z, Taylor JA (2016) RCP: a novel probe design bias correction method for Illumina Methylation BeadChip. Bioinformatics 32:2659–2663. https://doi.org/10.1093/bioinformatics/btw285
122. Touleimat N, Tost J (2012) Complete pipeline for Infinium(®) Human Methylation 450K BeadChip data processing using subset quantile normalization for accurate DNA methylation estimation. Epigenomics 4:325–341. https://doi.org/10.2217/epi.12.21
123. Benjamini Y, Hochberg Y (1995) Controlling the False Discovery Rate: A Practical and Powerful Approach to Multiple Testing. Journal of the Royal Statistical Society Series B (Methodological) 57:289–300
124. Borisov V, Leemann T, Seßler K, Haug J, Pawelczyk M, Kasneci G (2022) Deep Neural Networks and Tabular Data: A Survey. arXiv:211001889 [cs]
125. Friedman J (2001) Greedy Function Approximation: A Gradient Boosting Machine. The Annals of Statistics 20:1189–1232. https://doi.org/10.1214/aos/1013203451
126. Zhao Y, Chetty G, Tran D (2019) Deep Learning with XGBoost for Real Estate Appraisal. In: 2019 IEEE Symposium Series on Computational Intelligence (SSCI). pp 1396–1401
127. Santhanam R, Uzir N, Raman S, Banerjee S (2017) Experimenting XGBoost Algorithm for Prediction and Classification of Different Datasets
128. Kingma DP, Ba J (2017) Adam: A Method for Stochastic Optimization. arXiv:14126980 [cs]
129. Little RJA, Rubin DB (2020) Statistical analysis with missing data, Third edition. Wiley, Hoboken, NJ
130. Bennett DA (2001) How can I deal with missing data in my study? Aust N Z J Public Health 25:464–469
131. Jerez JM, Molina I, García-Laencina PJ, Alba E, Ribelles N, Martín M, Franco L (2010) Missing data imputation using statistical and machine learning methods in a real breast cancer problem. Artificial Intelligence in Medicine 50:105–115. https://doi.org/10.1016/j.artmed.2010.05.002
132. Khan SI, Hoque ASML (2020) SICE: an improved missing data imputation technique. J Big Data 7:37. https://doi.org/10.1186/s40537-020-00313-w
133. Lin W-C, Tsai C-F (2020) Missing value imputation: a review and analysis of the literature (2006–2017). Artif Intell Rev 53:1487–1509. https://doi.org/10.1007/s10462-019-09709-4
134. Rubin LH, Witkiewitz K, Andre JS, Reilly S (2007) Methods for Handling Missing Data in the Behavioral Neurosciences: Don’t Throw the Baby Rat out with the Bath Water. J Undergrad Neurosci Educ 5:A71-77
135. Delalleau O, Courville A, Bengio Y (2018) Efficient EM Training of Gaussian Mixtures with Missing Data. arXiv:12090521 [cs, stat]
136. Andridge RR, Little RJA (2010) A Review of Hot Deck Imputation for Survey Non-response. International Statistical Review 78:40–64. https://doi.org/10.1111/j.1751-5823.2010.00103.x
137. Cheema JR (2014) A Review of Missing Data Handling Methods in Education Research. Review of Educational Research 84:487–508. https://doi.org/10.3102/0034654314532697
138. Jonsson P, Wohlin C (2004) An evaluation of k-nearest neighbour imputation using likert

data. In: 10th International Symposium on Software Metrics, 2004. Proceedings. IEEE, Chicago, IL, USA, pp 108–118
139. Maillo J, Ramírez S, Triguero I, Herrera F (2017) kNN-IS: An Iterative Spark-based design of the k-Nearest Neighbors classifier for big data. Knowledge-Based Systems 117:3–15. https://doi.org/10.1016/j.knosys.2016.06.012
140. Amirteimoori A, Kordrostami S (2010) A Euclidean distance-based measure of efficiency in data envelopment analysis. Optimization 59:985–996. https://doi.org/10.1080/02331930902878333
141. Beretta L, Santaniello A (2016) Nearest neighbor imputation algorithms: a critical evaluation. BMC Medical Informatics and Decision Making 16:74. https://doi.org/10.1186/s12911-016-0318-z
142. Acuña E, Rodriguez C (2004) The Treatment of Missing Values and its Effect on Classifier Accuracy. In: Banks D, McMorris FR, Arabie P, Gaul W (eds) Classification, Clustering, and Data Mining Applications. Springer, Berlin, Heidelberg, pp 639–647
143. Lee JY, Styczynski MP (2018) NS-kNN: a modified k-nearest neighbors approach for imputing metabolomics data. Metabolomics 14:153. https://doi.org/10.1007/s11306-018-1451-8
144. Sun B, Ma L, Cheng W, Wen W, Goswami P, Bai G (2017) An improved k-nearest neighbours method for traffic time series imputation. In: 2017 Chinese Automation Congress (CAC). pp 7346–7351
145. Cheng D, Zhang S, Deng Z, Zhu Y, Zong M (2014) kNN Algorithm with Data-Driven k Value. In: Luo X, Yu JX, Li Z (eds) Advanced Data Mining and Applications. Springer International Publishing, Cham, pp 499–512
146. Murti DMP, Pujianto U, Wibawa AP, Akbar MI (2019) K-Nearest Neighbor (K-NN) based Missing Data Imputation. In: 2019 5th International Conference on Science in Information Technology (ICSITech). pp 83–88
147. Huang J, Keung JW, Sarro F, Li Y-F, Yu YT, Chan WK, Sun H (2017) Cross-validation based K nearest neighbor imputation for software quality datasets: An empirical study. Journal of Systems and Software 132:226–252. https://doi.org/10.1016/j.jss.2017.07.012
148. Zhu M, Cheng X (2015) Iterative KNN imputation based on GRA for missing values in TPLMS. In: 2015 4th International Conference on Computer Science and Network Technology (ICCSNT). pp 94–99
149. Zhang S, Li X, Zong M, Zhu X, Cheng D (2017) Learning *k* for kNN Classification. ACM Trans Intell Syst Technol 8:43:1-43:19. https://doi.org/10.1145/2990508
150. Samek W, Wiegand T, Müller K-R (2017) Explainable Artificial Intelligence: Understanding, Visualizing and Interpreting Deep Learning Models. arXiv:170808296 [cs, stat]
151. Caruana R, Lou Y, Gehrke J, Koch P, Sturm M, Elhadad N (2015) Intelligible Models for HealthCare: Predicting Pneumonia Risk and Hospital 30-day Readmission. In: Proceedings of the 21th ACM SIGKDD International Conference on Knowledge Discovery and Data Mining. Association for Computing Machinery, New York, NY, USA, pp 1721–1730
152. Lapuschkin S, Binder A, Montavon G, Muller KR, Samek W (2016) Analyzing Classifiers: 29th IEEE Conference on Computer Vision and Pattern Recognition, CVPR 2016. Proceedings - 29th IEEE Conference on Computer Vision and Pattern Recognition, CVPR 2016 2912–2920. https://doi.org/10.1109/CVPR.2016.318

153. Arras L, Horn F, Montavon G, Müller K-R, Samek W (2016) Explaining Predictions of Non-Linear Classifiers in NLP. In: Proceedings of the 1st Workshop on Representation Learning for NLP. Association for Computational Linguistics, Berlin, Germany, pp 1–7
154. Arras L, Horn F, Montavon G, Müller K-R, Samek W (2017) "What is relevant in a text document?": An interpretable machine learning approach. PLOS ONE 12:e0181142. https://doi.org/10.1371/journal.pone.0181142
155. Schütt KT, Arbabzadah F, Chmiela S, Müller KR, Tkatchenko A (2017) Quantum-chemical insights from deep tensor neural networks. Nat Commun 8:13890. https://doi.org/10.1038/ncomms13890
156. Sturm I, Lapuschkin S, Samek W, Müller K-R (2016) Interpretable deep neural networks for single-trial EEG classification. Journal of Neuroscience Methods 274:141–145. https://doi.org/10.1016/j.jneumeth.2016.10.008
157. Lipovetsky S, Conklin M (2001) Analysis of regression in game theory approach. Applied Stochastic Models in Business and Industry 17:319–330. https://doi.org/10.1002/asmb.446

Supplementary Table S1

Click here to access/download
Supplementary Material
TableS1 Supplementary Material.xlsx

Supplementary Table S2

Click here to access/download
Supplementary Material
TableS2 Supplementary Material.xlsx

Supplementary Table S3

Click here to access/download
Supplementary Material
TableS3 Supplementary Material.xlsx

Response to Reviewers Click here to access/download;Personal Cover;Response to Reviewers.docx

Dear Editor,

We were pleased to receive a thorough and helpful feedback from both reviewers with the generally positive evaluation of the manuscript. Having carefully addressed their comments and suggestions, we resubmit the revised manuscript. We hope that it warrants publication in the present form.
The point-to-point reply to the reviewers' comments follows.

On behalf of all authors,
Alena Kalyakulina

Reviewer 1

The paper by Kalyakulina et al. described the disease classification for whole blood DNA methylation. The author proposed a comprehensive approach of combined DNA methylation datasets to classify controls and patients. The solution includes data harmonization, construction of machine learning classification models, dimensionality reduction of models, imputation of missing values, and explanation of model predictions by explainable artificial intelligence algorithms. For Parkinson's disease and schizophrenia, the author also demonstrates that a method for classifying healthy individuals and patients with various disorders based on whole blood DNA methylation data is an efficient and comprehensive approach. Overall, the manuscript is well organized. I have some suggestions for the authors to improve their work:

Comment 1. The manuscript has constructed different models for the prediction study of CpG sites for different types of data. It is suggested to add a flowchart of the whole model construction process to the manuscript so that readers can understand the study more clearly.

Our reply:
We have added Figure 1 with the schematic representation of all main steps of the proposed workflow.

Comment 2. In Figure 4, the author only shows the top 10 important features and marks the highest accuracy and number of features with black lines in the figure. It is recommended to show the relevant data (optimal accuracy and number of features) in the figure. For the three subplots included in the figure, please label them separately, e.g., A, B, and C to indicate them separately.)

Our reply:
We followed the suggestion and added letter labels to the subplots. In Figure 4, the parameters of the optimal small models (accuracy and number of features) are introduced in subplots B and E.

Comment 3. Remark concerns model performance evaluation: author should provide standard deviations of the obtained values.

Our reply:
In this paper, we consider a common case (dictated by the practice), when the model is trained on certain data sets and tested on completely different data sets, which are new and unfamiliar to it. In this setup cross-validation is not applicable, and therefore it is not possible to calculate the standard deviation on different folds. It is worth mentioning that for the situation we are considering, a common problem is the absence of some features in the test data and the need to impute them. The built model should always accept certain attributes, on which the training has been performed, even if they are missing in the test data for some reason (this problem is covered in the subsections "Imputation of missing values" of Results and Methods sections). In particular, the best-known epigenetic clock models of Horvath or Hannum are built on a strictly defined number of CpG sites (353 and 71 CpG sites, respectively). In independent test datasets, some of these CpG sites may be missing due to, for example, failed quality control checks. The epigenetic clock of Horvath and Hannum imputes missing values either by simple methods (mean) or KNN with 10 neighbors. The simplest methods within our work do not show better results, nor does increasing the number of neighbors in KNN lead to better results for all the test datasets we consider.
It could be possible to combine all datasets together and apply cross-validation, but in this case one has to ensure that the most important features in the resulting model are not caused by the batch effects of certain datasets (which can be most evident in the case of non-harmonized data). Such features can be dataset-specific and not biologically relevant. They can have a strong impact on the quality of the model when validating it on completely new data. This task may be the subject of further research beyond the context of this study.

Comment 4. In this manuscript, the author used graphs to present the results and suggested that a table summarizing the performance results of the model would be intuitive.

Our reply:
Figures with the results of classification and imputation have been replaced by tables.

Comment 5. I didn't find how the authors optimize the hyper-parameters, usually using grid search.

Our reply:
To find the optimal combination of model parameters that provides the best accuracy, we made use of hyperparametric grid search (the corresponding values are presented in Supplementary Table 1). Previously, this was only mentioned in the Classification Models subsection of the Methods section. We also added information about hyperparametric search in the Classification Models subsection of Results. Supplementary tables have been renumbered according to this change.

Comment 6. The authors do not adequately address how their method outperforms existing methods in the discussion section.

Our reply:
The task of classifying cases and controls for Parkinson's disease and schizophrenia based on whole blood DNA methylation data is underrepresented in the literature. For Parkinson's disease, the task of classifying different neurodegenerative diseases using DNA methylation-derived profile scores was solved (Nabais et.al., 2021). For Parkinson's disease in this study, the best result was an AUROC value of 0.7 for binary classification of two different datasets with this disease as well as for binary classification of Parkinson's disease and rheumatoid arthritis. In the same study for schizophrenia, the best result was an AUROC value of 0.78 for the binary classification of schizophrenia and rheumatoid arthritis. For schizophrenia, the task of classifying cases and controls was considered, but only for CoRSIV probes with polygenic risk score (Gunasekara et. al., 2021). The paper does not provide accuracy values, but only positive predictive values for cases (not controls) with the best result of 86%. It is worth noting that this work uses the dataset GSE84727 for model training and GSE80417 for the testing. These 2 datasets use the same data preprocessing (as we mentioned in Table 1), which can affect the result. Both works did not use data harmonization, did not consider the problem of missing value imputation, and did not explain the model outputs.
Thus, in this formulation, the task of classifying cases and controls from whole blood DNA methylation data with harmonization, imputation of missing values, and application of XAI approaches is solved for the first time. We added references to the works mentioned above in Discussion.

Comment 7. The "Dimensionality reduction" section: I think this section is more appropriately called "feature selection", a sequence forward search method. First sort the features according to their importance values, then add or remove features from a candidate subset while evaluating the criterion.

Our reply:
We appreciate the suggestion, but would refrain from renaming "Dimensionality reduction" to "Feature selection" due to possible confusion. The initial feature selection is done in the "Meta-analysis and harmonization" subsection (workflow step). For each disease and for all CpG sites in the training datasets, we perform the Mann-Whitney U-test for the control group only. CpG sites with an adjusted p-value >0.05 have similar distributions of methylation levels among controls in different datasets. These CpG sites are used later for machine learning models. We do not use cases because their methylation levels may have high variability due to disease heterogeneity.
In the "Dimensionality reduction" subsection, we build multiple models for the best method with a varying number of features (from 10 to 1000 in increments of 10) based on the importance rating. The resulting best model has a much smaller number of features as compared to the original one, without much loss in accuracy. This can indeed be called a second feature selection, but we believe that it does not need to be renamed so as not to be confused with the feature selection based on the equality of methylation level distributions in the control group. We first perform feature selection to build the model and then reduce the dimensionality of the best model already built.

Reviewer 2

In this study, a workflow is presented to generate classification models from DNA methylation data. Methods to deal with harmonization and missing data imputation are presented and the benefit of adopting them for classification tasks is tested on case-control datasets of schizophrenia and Parkinson disease. The authors support this workflow with source code.
Although mostly based on already known methodologies, the present study may help orient studies aimed at building and applying DNA methylation based models. However, some major concerns can be raised:

Majors:

Comment 1. In different points of the manuscript, the authors refer to their approach as a pipeline. Indeed, this approach should be composed of sequential modules, in which the output of a module becomes the input of the next one. Although the modules are clearly distinguishable, their organization in the pipeline is less straightforward (also considering that modules can be adopted both to build a model and to use it on new data). The authors could think to draw a scheme of the pipeline, or to adopt a different term to refer to the presented approach.

Our reply:
We renamed "pipeline" to "workflow" because, in our opinion, this term better reflects the essence of the proposed approach - a sequence of modules executed in a certain order, where the output of one module is the input of the next one. We have also added Figure 1 with an illustration of the proposed workflow.

Comment 2. From the model performance perspective, the ML models poorly perform for schizophrenia. The authors point to inner characteristics of the disease as a possible reason for this. However, this point should be better commented in the Discussion section. Besides this, the impact of the smaller number of samples included in the training set and the higher proportion of imputed features compared to Parkinson disease on the classification accuracy should be discussed. In addition, since the authors provided the code, is there a way to select samples to include in training/test sets based on random choice (classical 70-30% splitting) instead of source dataset?

Our reply:
The requested comments supported by the related literature have been added to Discussion. In particular, it is pointed out that schizophrenia is a complex disease characterized by many different symptoms, with a variety of causes and molecular patterns. The individual molecular landscape can vary considerably among patients with schizophrenia. Fewer samples in the training sample for schizophrenia may also affect the final result. Insufficient data may not fully describe the disease characteristics for the model.
The experiment with imputation of missing values in our case was synthetic. For both Parkinson's disease and schizophrenia, the test data sets did not contain missing values in those CpG sites that participated in the construction of the best small models. For each disease, we removed 100 CpG sites, which were top-ranked by importance value. These 100 CpG sites

were then imputed using various methods, and their results were compared with the original model. Despite the fact that in our case there were no missing data, in real life such a factor as the number of missing values (and, accordingly, the quality of their filling) can have a significant impact on the final result.

Classic cross-validation can be applied in this case. This may have an impact on the result, since the training set will include samples from different datasets. Here, it is critical to make sure that the most important features in the resulting model are not due to batch effects or different preprocessing of specific datasets (which is most obvious in the case of non-harmonized data). Such attributes can affect the quality of the model when tested on entirely new data. However, the focus of our work is not the case where we have all the data on hand at once (and no new data are expected), but a closer to reality case in which the model is trained on some data and tested later on others (which may have a different preprocessing, bias, or batch effect). It is important that the model is not re-trained. In this formulation of the problem, we do not use cross-validation, so as not to mix training and test sets. Therefore, the harmonization step is particularly important.

Comment 3. "For machine learning models, we used only those CpG sites that have the same distribution of methylation levels in different datasets in the control group (methylation levels in the case group typically have greater variability because of disease heterogeneity).": is this filtering performed only on the datasets included in the training set, or also on the test set? It seems the former, but the authors should clearly state this point.

Our reply:

We selected those CpG sites that have the same methylation level distributions in different train datasets in the control group only. An appropriate correction has been added to the "Meta-analysis and harmonization" subsection of the Results section and to the "Data harmonization" subsection of the Methods section.

Comment 4. Accuracy with weighted averaging should be defined with a formula in the methods section

Our reply:

We added a formula for accuracy with weighted averaging to the "Classification models" subsection of the Methods section.

Comment 5. Regarding the ML models, the authors chose different types of decision-trees ensemble, along with a deep learning one. They should contextualize this choice (why different models from the same family?). In addition, ML models built on DNA methylation are often based on elastic net or Support-Vector Machines, which are not accounted for in this work. The authors should comment on this aspect in limitations, and state whether the code they provided for their approach could be customized to adopt different models from the ones they presented.

Our reply:

Gradient-boosting decision trees (GBDT) have recently become state-of-the-art models for solving classification and regression problems on tabular data. GBDTs train multiple decision

trees to predict the outcome. Although the three considered models, XGBoost, CatBoost, and LightGBM, belong to the same group of methods, they all have structural differences. XGBoost iteratively trains an ensemble of shallow decision trees, with each iteration using error residuals from the previous model to fit the next model. The final prediction is a weighted sum of all tree predictions. CatBoost builds symmetric trees. At each step, the leaves of the previous tree are separated by the same condition. A feature-split pair is selected and used for all nodes, which provides the least losses. LightGBM splits the tree by leaf with the simplest fit, whereas other boosting algorithms split the tree by depth or by level rather than by leaf. The Neural Oblivious Decision Ensembles (NODE) architecture generalizes ensembles of oblivious decision trees, but benefits from both end-to-end gradient-based optimization and multilevel hierarchical learning capabilities. Information about the differences between the considered models is presented in the "Classification models" subsection of the Methods section. In our experiments, these models demonstrate different performance. For example, for non-harmonized Parkinson's disease data, the LightGBM model shows the best performance, exceeding the accuracy of the CatBoost model by 5%. At the same time, for harmonized schizophrenia data, the CatBoost model's result exceeds the NODE model's result by 6%.
The Elastic Net model is indeed often applied to DNA methylation data, especially for the epigenetic clock. However, this approach in the classical sense is used to solve the regression problem, while logistic regression with the elastic net penalty is used for classification.
We added the Logistic Regression and Support-Vector Machine (SVM) models to consideration. We applied both methods to both diseases, Parkinson's disease and schizophrenia, to non-harmonized and harmonized data. We found that these classical approaches do not outperform the GBDT models. These methods have also been added to the code repository.

Comment 6. Regarding the Imputation Method column in Table 2, the meaning is not clear. Are the different imputation methods described in the Imputation of missing values section paired with the ML models presented in Table 2? If yes, some of the methods (like KNN) are missing.

Our reply:
Table 2 provides information about the missing value imputation methods integrated into the considered models and provided by their API. Since not all models perform imputation themselves, we consider it as a separate necessary step in the described workflow. To avoid misunderstanding, we removed the Imputation method column from Table 2.

Comment 7. In the harmonization section, Models for case-control classification are trained on different numbers and sets of CpGs. To assess the effect of harmonization alone, the number of CpGs should be instead fixed. This is especially critical for schizophrenia, when the number of features for the non-harmonized data is 35145 whereas the one for harmonized data is 110,137.

Our reply:
The first step in the described workflow is data harmonization. The regRCPqn approach is used (regional regression on correlated probes with quantile normalization). One of the goals is to

compare classification results using data without and with harmonization. However, not all CpG sites are used to build classification models.

Let us first consider Parkinson's disease. The two training datasets are GSE145361 and GSE111629. Only the control group is considered. For all CpG sites in these datasets, the Mann-Whitney U-test is performed for the control group to compare the distributions of methylation levels. We select only those CpG sites for which the adjusted p-value is >0.05. If the data are not harmonized, there will be 43019 such CpG sites. In the case of harmonized data, the condition is satisfied for 50911 CpG sites. Classification models are constructed for these selected CpG sites. Similarly for schizophrenia. The two training datasets are GSE84727 and GSE80417. In the control group, the Mann-Whitney U-test is performed for these datasets for all CpG sites. For non-harmonized data, the condition for the adjusted p-value is satisfied for 35145 CpG sites, and for harmonized data, the condition for 110137 CpG sites is satisfied. Thus, we consider all CpG sites that passed the test for p-value for each case.

The intersection of the resulting CpG sites for non-harmonized and harmonized data contains 13370 CpG sites for Parkinson's disease and 4539 CpG sites for schizophrenia. The overlap is not too great. This may be due to the fact that for non-harmonized data, CpG sites may have a similar distribution of methylation levels for a variety of reasons related to batch-related variability, chip position in batches, different preprocessing, and other non-biological reasons. Whereas, the same methylation level distributions for the harmonized data are devoid of dependence on the specific dataset and largely reflect biologically relevant causes.

We performed classification experiments for the best models: LightGBM for Parkinson's disease and CatBoost for schizophrenia. CpG sites from the intersection of lists for non-harmonized and harmonized data were used as features. Thus, the model for Parkinson's disease was trained on 13370 features, and the model for schizophrenia was trained on 4539 features. For GSE72774 (Parkinson's disease) LightGBM has reached accuracy of 0.75 for the non-harmonized data and 0.94 for harmonized data. For GSE152027 (Schizophrenia) CatBoost has reached accuracy of 0.67 for non-harmonized data and 0.71 for harmonized data. For GSE116379 (Schizophrenia) CatBoost has reached accuracy of 0.58 for non-harmonized data and 0.67 for harmonized data. The results retain the trend found for the complete data. Harmonization works well for Parkinson's disease; for schizophrenia, improved accuracy is observed for the test dataset with different preprocessing. However, the overall accuracy values were lower than those described in the paper.

Our results for non-harmonized and harmonized data show that harmonization either leaves the result the same or improves it. Therefore, in practice, when working with multiple test datasets within our workflow, it is preferable to perform harmonization. In this case, adding information related to non-harmonized data does not bring substantial benefit.

Comment 8. Dimensionality reduction section: are the models from imputed and not-imputed data trained only on harmonized data? And how the set of 50911CpG sites for Parkinson and 110137 CpG sites for schizophrenia is selected?

Our reply:

For the best models in terms of accuracy, all the following steps (dimensionality reduction, imputation of missing values, XAI) are performed using only harmonized data. Models for Parkinson's are trained on 50911 harmonized CpGs; models for schizophrenia are trained on

110137 harmonized CpGs. They are chosen based on distributions of methylation levels in controls. For the harmonized data, the Mann-Whitney U-test is performed, and CpG sites are selected for which the p-value >0.05. Such CpG sites have the same distribution of methylation levels in the training data sets from controls.
This approach avoids the situation in which the machine learning model selects CpG sites specific to a particular dataset as the most important features. These CpG sites will be useless for other datasets, where the distribution of methylation levels for them may be different.
This approach minimizes the risk of a situation where the model implicitly tries to solve separate binary classification problems in each individual dataset and finally solves a new multiclass classification problem whose classes correlate with specific datasets (instead of the "case vs control" problem, the "case_dataset_1 vs control_dataset_1 vs case_dataset_2 vs control_dataset_2 vs ..." problem would be solved implicitly).

Comment 9. Imputation of missing values section: it is not clear on which CpGs and on which samples imputation is performed. Also, it is not clear whether the imputation has been tested on the best-performing model.

Our reply:
The small optimal models built in the "Dimensionality reduction" subsection (for harmonized data) are used for the imputation stage. The LightGBM model with 890 CpG sites is used for Parkinson's disease, and the CatBoost model with 670 CpG sites is used for schizophrenia.
For each model, 100 CpG sites with the highest values are excluded. The number of CpG sites was chosen to cause a significant drop in accuracy and to emphasize the differences in efficiency between the imputation methods. There are no missing features in the resulting small optimal models, so real CpG sites are excluded from consideration.
The different imputation methods are tested on the same datasets as before: GSE72774 for Parkinson's disease, GSE152027, and GSE116379 for schizophrenia. We examine how the accuracy of the "cases vs controls" classification changes when different imputation methods are used relative to the baseline model on data without missing features.
The text of the subsection "Imputation of missing values" is rephrased accordingly.

Minors:

Comment 1. Page 1, line 2: "DNA methylation is associated with epigenetic modification". DNA methylation is an epigenetic mark itself. Do the authors mean histone marks?

Our reply:
Indeed, DNA methylation is an epigenetic modification. This wording is a typo, and it has been corrected.

Comment 2. Page 1, from line 7: "DNA methylation consists of binding a methyl group to cytosine in the cytosine-guanine dinucleotides (CpG sites). Hypermethylation of CpG sites near the gene promoter is known to repress transcription, while hypermethylation in the gene body appears to have an opposite, also less pronounced effect.": references should be added

Our reply:

We followed the suggestion and added references that mention this statement: (Jones, 2012) and (Jjino et. al., 2012).

Comment 3. Page 2, from line 2 : "Current epigenome-wide association studies (EWAS) test DNAm associations with human phenotypes, health conditions and diseases.": references should be added

Our reply:
We followed the suggestion and added references supporting this statement: (Rakyan et. al., 2011), (Liu et. al., 2019) and (Birney et. al., 2016).

Comment 4. Page 3: "In most cases, an increase in dimensionality does not provide significant benefits, since lower dimensionality data may contain more relevant information".
This point could be presented in a reverse way (higher dimensionality data may contain redundant information), introducing the collinearity issue.
In addition, this issue could be introduced before the missing values and imputation section.

Our reply:
We have rephrased the statement about the relationship between the dimensionality of the data and the information it contains.

Comment 5. Page 3: references for "Modern machine-learning-based artificial intelligence systems are powerful and promising tools" could be more specific for the field of epigenetics and DNA methylation.

Our reply:
We have rephrased the statement and added references to works related to the analysis of biomedical data and, in particular, DNA methylation. In this paragraph, we wanted to point out the problem of explainability from a global point of view, as this is a separate problem for any models, not only for DNA methylation analysis.
