# Supplementary material for: Disease classification for whole-blood DNA methylation: Meta-analysis, missing values imputation, and XAI
Source: Gigascience. 2022 Oct 19;11:giac097. doi: 10.1093/gigascience/giac097 (PMC9718659; doi:10.1093/gigascience/giac097)
Supplement: giac097_Response_to_Reviewer_Comments_Original_Submission [file giac097_response_to_reviewer_comments_original_submission.pdf]

Author's Response To Reviewer Comments

Close

Dear Editor,
We were pleased to receive a thorough and helpful feedback from both reviewers with the generally positive evaluation of the manuscript. Having carefully addressed their comments and suggestions, we resubmit the revised manuscript. We hope that it warrants publication in the present form.
The point-to-point reply to the reviewers’ comments follows.

On behalf of all authors,
Alena Kalyakulina

Reviewer 1
The paper by Kalyakulina et al. described the disease classification for whole blood DNA methylation. The author proposed a comprehensive approach of combined DNA methylation datasets to classify controls and patients. The solution includes data harmonization, construction of machine learning classification models, dimensionality reduction of models, imputation of missing values, and explanation of model predictions by explainable artificial intelligence algorithms. For Parkinson's disease and schizophrenia, the author also demonstrates that a method for classifying healthy individuals and patients with various disorders based on whole blood DNA methylation data is an efficient and comprehensive approach. Overall, the manuscript is well organized. I have some suggestions for the authors to improve their work:

Comment 1. The manuscript has constructed different models for the prediction study of CpG sites for different types of data. It is suggested to add a flowchart of the whole model construction process to the manuscript so that readers can understand the study more clearly.

Our reply:
We have added Figure 1 with the schematic representation of all main steps of the proposed workflow.

Comment 2. In Figure 4, the author only shows the top 10 important features and marks the highest accuracy and number of features with black lines in the figure. It is recommended to show the relevant data (optimal accuracy and number of features) in the figure. For the three subplots included in the figure, please label them separately, e.g., A, B, and C to indicate them separately.)

Our reply:
We followed the suggestion and added letter labels to the subplots. In Figure 4, the parameters of the optimal small models (accuracy and number of features) are introduced in subplots B and E.

Comment 3. Remark concerns model performance evaluation: author should provide standard deviations of the obtained values.

Our reply:
In this paper, we consider a common case (dictated by the practice), when the model is trained on certain data sets and tested on completely different data sets, which are new and unfamiliar to it. In this setup cross-validation is not applicable, and therefore it is not possible to calculate the standard deviation on different folds. It is worth mentioning that for the situation we are considering, a common problem is the absence of some features in the test data and the need to impute them. The built model should always accept certain attributes, on which the training has been performed, even if they are missing in the test data for some reason (this problem is covered in the subsections "Imputation of missing values" of Results and Methods sections). In particular, the best-known epigenetic clock models of Horvath or Hannum are built on a strictly defined number of CpG sites (353 and 71 CpG sites, respectively). In independent test datasets, some of these CpG sites may be missing due to, for example, failed quality control checks. The epigenetic clock of Horvath and Hannum imputes missing values either by simple methods (mean) or KNN with 10 neighbors. The simplest methods within our

work do not show better results, nor does increasing the number of neighbors in KNN lead to better results for all the test datasets we consider.
It could be possible to combine all datasets together and apply cross-validation, but in this case one has to ensure that the most important features in the resulting model are not caused by the batch effects of certain datasets (which can be most evident in the case of non-harmonized data). Such features can be dataset-specific and not biologically relevant. They can have a strong impact on the quality of the model when validating it on completely new data. This task may be the subject of further research beyond the context of this study.

Comment 4. In this manuscript, the author used graphs to present the results and suggested that a table summarizing the performance results of the model would be intuitive.

Our reply:
Figures with the results of classification and imputation have been replaced by tables.

Comment 5. I didn't find how the authors optimize the hyper-parameters, usually using grid search.

Our reply:
To find the optimal combination of model parameters that provides the best accuracy, we made use of hyperparametric grid search (the corresponding values are presented in Supplementary Table 1). Previously, this was only mentioned in the Classification Models subsection of the Methods section. We also added information about hyperparametric search in the Classification Models subsection of Results. Supplementary tables have been renumbered according to this change.

Comment 6. The authors do not adequately address how their method outperforms existing methods in the discussion section.

Our reply:
The task of classifying cases and controls for Parkinson's disease and schizophrenia based on whole blood DNA methylation data is underrepresented in the literature. For Parkinson's disease, the task of classifying different neurodegenerative diseases using DNA methylation-derived profile scores was solved (Nabais et.al., 2021). For Parkinson's disease in this study, the best result was an AUROC value of 0.7 for binary classification of two different datasets with this disease as well as for binary classification of Parkinson's disease and rheumatoid arthritis. In the same study for schizophrenia, the best result was an AUROC value of 0.78 for the binary classification of schizophrenia and rheumatoid arthritis. For schizophrenia, the task of classifying cases and controls was considered, but only for CoRSIV probes with polygenic risk score (Gunasekara et. al., 2021). The paper does not provide accuracy values, but only positive predictive values for cases (not controls) with the best result of 86%. It is worth noting that this work uses the dataset GSE84727 for model training and GSE80417 for the testing. These 2 datasets use the same data preprocessing (as we mentioned in Table 1), which can affect the result. Both works did not use data harmonization, did not consider the problem of missing value imputation, and did not explain the model outputs.
Thus, in this formulation, the task of classifying cases and controls from whole blood DNA methylation data with harmonization, imputation of missing values, and application of XAI approaches is solved for the first time. We added references to the works mentioned above in Discussion.

Comment 7. The "Dimensionality reduction" section: I think this section is more appropriately called "feature selection", a sequence forward search method. First sort the features according to their importance values, then add or remove features from a candidate subset while evaluating the criterion.

Our reply:
We appreciate the suggestion, but would refrain from renaming "Dimensionality reduction" to "Feature selection" due to possible confusion. The initial feature selection is done in the "Meta-analysis and harmonization" subsection (workflow step). For each disease and for all CpG sites in the training datasets, we perform the Mann-Whitney U-test for the control group only. CpG sites with an adjusted p-value >0.05 have similar distributions of methylation levels among controls in different datasets. These CpG sites are used later for machine learning models. We do not use cases because their methylation levels may have high variability due to disease heterogeneity.
In the "Dimensionality reduction" subsection, we build multiple models for the best method with a varying number of features (from 10 to 1000 in increments of 10) based on the importance rating. The resulting best model has a much smaller number of features as compared to the original one, without much loss in accuracy. This can indeed be called a second feature selection, but we believe that it does

not need to be renamed so as not to be confused with the feature selection based on the equality of methylation level distributions in the control group. We first perform feature selection to build the model and then reduce the dimensionality of the best model already built.

Reviewer 2
In this study, a workflow is presented to generate classification models from DNA methylation data. Methods to deal with harmonization and missing data imputation are presented and the benefit of adopting them for classification tasks is tested on case-control datasets of schizophrenia and Parkinson disease. The authors support this workflow with source code.
Although mostly based on already known methodologies, the present study may help orient studies aimed at building and applying DNA methylation based models. However, some major concerns can be raised:

Majors:
Comment 1. In different points of the manuscript, the authors refer to their approach as a pipeline. Indeed, this approach should be composed of sequential modules, in which the output of a module becomes the input of the next one. Although the modules are clearly distinguishable, their organization in the pipeline is less straightforward (also considering that modules can be adopted both to build a model and to use it on new data). The authors could think to draw a scheme of the pipeline, or to adopt a different term to refer to the presented approach.

Our reply:
We renamed "pipeline" to "workflow" because, in our opinion, this term better reflects the essence of the proposed approach - a sequence of modules executed in a certain order, where the output of one module is the input of the next one. We have also added Figure 1 with an illustration of the proposed workflow.

Comment 2. From the model performance perspective, the ML models poorly perform for schizophrenia. The authors point to inner characteristics of the disease as a possible reason for this. However, this point should be better commented in the Discussion section. Besides this, the impact of the smaller number of samples included in the training set and the higher proportion of imputed features compared to Parkinson disease on the classification accuracy should be discussed. In addition, since the authors provided the code, is there a way to select samples to include in training/test sets based on random choice (classical 70-30% splitting) instead of source dataset?

Our reply:
The requested comments supported by the related literature have been added to Discussion. In particular, it is pointed out that schizophrenia is a complex disease characterized by many different symptoms, with a variety of causes and molecular patterns. The individual molecular landscape can vary considerably among patients with schizophrenia. Fewer samples in the training sample for schizophrenia may also affect the final result. Insufficient data may not fully describe the disease characteristics for the model.
The experiment with imputation of missing values in our case was synthetic. For both Parkinson's disease and schizophrenia, the test data sets did not contain missing values in those CpG sites that participated in the construction of the best small models. For each disease, we removed 100 CpG sites, which were top-ranked by importance value. These 100 CpG sites were then imputed using various methods, and their results were compared with the original model. Despite the fact that in our case there were no missing data, in real life such a factor as the number of missing values (and, accordingly, the quality of their filling) can have a significant impact on the final result.
Classic cross-validation can be applied in this case. This may have an impact on the result, since the training set will include samples from different datasets. Here, it is critical to make sure that the most important features in the resulting model are not due to batch effects or different preprocessing of specific datasets (which is most obvious in the case of non-harmonized data). Such attributes can affect the quality of the model when tested on entirely new data. However, the focus of our work is not the case where we have all the data on hand at once (and no new data are expected), but a closer to reality case in which the model is trained on some data and tested later on others (which may have a different preprocessing, bias, or batch effect). It is important that the model is not re-trained. In this formulation of the problem, we do not use cross-validation, so as not to mix training and test sets. Therefore, the harmonization step is particularly important.

Comment 3. "For machine learning models, we used only those CpG sites that have the same

distribution of methylation levels in different datasets in the control group (methylation levels in the case group typically have greater variability because of disease heterogeneity).": is this filtering performed only on the datasets included in the training set, or also on the test set? It seems the former, but the authors should clearly state this point.

Our reply:
We selected those CpG sites that have the same methylation level distributions in different train datasets in the control group only. An appropriate correction has been added to the "Meta-analysis and harmonization" subsection of the Results section and to the "Data harmonization" subsection of the Methods section.

Comment 4. Accuracy with weighted averaging should be defined with a formula in the methods section

Our reply:
We added a formula for accuracy with weighted averaging to the "Classification models" subsection of the Methods section.

Comment 5. Regarding the ML models, the authors chose different types of decision-trees ensemble, along with a deep learning one. They should contextualize this choice (why different models from the same family?). In addition, ML models built on DNA methylation are often based on elastic net or Support-Vector Machines, which are not accounted for in this work. The authors should comment on this aspect in limitations, and state whether the code they provided for their approach could be customized to adopt different models from the ones they presented.

Our reply:
Gradient-boosting decision trees (GBDT) have recently become state-of-the-art models for solving classification and regression problems on tabular data. GBDTs train multiple decision trees to predict the outcome. Although the three considered models, XGBoost, CatBoost, and LightGBM, belong to the same group of methods, they all have structural differences. XGBoost iteratively trains an ensemble of shallow decision trees, with each iteration using error residuals from the previous model to fit the next model. The final prediction is a weighted sum of all tree predictions. CatBoost builds symmetric trees. At each step, the leaves of the previous tree are separated by the same condition. A feature-split pair is selected and used for all nodes, which provides the least losses. LightGBM splits the tree by leaf with the simplest fit, whereas other boosting algorithms split the tree by depth or by level rather than by leaf. The Neural Oblivious Decision Ensembles (NODE) architecture generalizes ensembles of oblivious decision trees, but benefits from both end-to-end gradient-based optimization and multilevel hierarchical learning capabilities. Information about the differences between the considered models is presented in the "Classification models" subsection of the Methods section. In our experiments, these models demonstrate different performance. For example, for non-harmonized Parkinson's disease data, the LightGBM model shows the best performance, exceeding the accuracy of the CatBoost model by 5%. At the same time, for harmonized schizophrenia data, the CatBoost model's result exceeds the NODE model's result by 6%.
The Elastic Net model is indeed often applied to DNA methylation data, especially for the epigenetic clock. However, this approach in the classical sense is used to solve the regression problem, while logistic regression with the elastic net penalty is used for classification.
We added the Logistic Regression and Support-Vector Machine (SVM) models to consideration. We applied both methods to both diseases, Parkinson's disease and schizophrenia, to non-harmonized and harmonized data. We found that these classical approaches do not outperform the GBDT models. These methods have also been added to the code repository.

Comment 6. Regarding the Imputation Method column in Table 2, the meaning is not clear. Are the different imputation methods described in the Imputation of missing values section paired with the ML models presented in Table 2? If yes, some of the methods (like KNN) are missing.

Our reply:
Table 2 provides information about the missing value imputation methods integrated into the considered models and provided by their API. Since not all models perform imputation themselves, we consider it as a separate necessary step in the described workflow. To avoid misunderstanding, we removed the Imputation method column from Table 2.

Comment 7. In the harmonization section, Models for case-control classification are trained on different numbers and sets of CpGs. To assess the effect of harmonization alone, the number of CpGs should be

instead fixed. This is especially critical for schizophrenia, when the number of features for the non-harmonized data is 35145 whereas the one for harmonized data is 110,137.

Our reply:
The first step in the described workflow is data harmonization. The regRCPqn approach is used (regional regression on correlated probes with quantile normalization). One of the goals is to compare classification results using data without and with harmonization. However, not all CpG sites are used to build classification models.
Let us first consider Parkinson's disease. The two training datasets are GSE145361 and GSE111629. Only the control group is considered. For all CpG sites in these datasets, the Mann-Whitney U-test is performed for the control group to compare the distributions of methylation levels. We select only those CpG sites for which the adjusted p-value is >0.05. If the data are not harmonized, there will be 43019 such CpG sites. In the case of harmonized data, the condition is satisfied for 50911 CpG sites. Classification models are constructed for these selected CpG sites. Similarly for schizophrenia. The two training datasets are GSE84727 and GSE80417. In the control group, the Mann-Whitney U-test is performed for these datasets for all CpG sites. For non-harmonized data, the condition for the adjusted p-value is satisfied for 35145 CpG sites, and for harmonized data, the condition for 110137 CpG sites is satisfied. Thus, we consider all CpG sites that passed the test for p-value for each case.
The intersection of the resulting CpG sites for non-harmonized and harmonized data contains 13370 CpG sites for Parkinson's disease and 4539 CpG sites for schizophrenia. The overlap is not too great. This may be due to the fact that for non-harmonized data, CpG sites may have a similar distribution of methylation levels for a variety of reasons related to batch-related variability, chip position in batches, different preprocessing, and other non-biological reasons. Whereas, the same methylation level distributions for the harmonized data are devoid of dependence on the specific dataset and largely reflect biologically relevant causes.
We performed classification experiments for the best models: LightGBM for Parkinson's disease and CatBoost for schizophrenia. CpG sites from the intersection of lists for non-harmonized and harmonized data were used as features. Thus, the model for Parkinson's disease was trained on 13370 features, and the model for schizophrenia was trained on 4539 features. For GSE72774 (Parkinson’s disease) LightGBM has reached accuracy of 0.75 for the non-harmonized data and 0.94 for harmonized data. For GSE152027 (Schizophrenia) CatBoost has reached accuracy of 0.67 for non-harmonized data and 0.71 for harmonized data. For GSE116379 (Schizophrenia) CatBoost has reached accuracy of 0.58 for non-harmonized data and 0.67 for harmonized data. The results retain the trend found for the complete data. Harmonization works well for Parkinson's disease; for schizophrenia, improved accuracy is observed for the test dataset with different preprocessing. However, the overall accuracy values were lower than those described in the paper.
Our results for non-harmonized and harmonized data show that harmonization either leaves the result the same or improves it. Therefore, in practice, when working with multiple test datasets within our workflow, it is preferable to perform harmonization. In this case, adding information related to non-harmonized data does not bring substantial benefit.

Comment 8. Dimensionality reduction section: are the models from imputed and not-imputed data trained only on harmonized data? And how the set of 50911CpG sites for Parkinson and 110137 CpG sites for schizophrenia is selected?

Our reply:
For the best models in terms of accuracy, all the following steps (dimensionality reduction, imputation of missing values, XAI) are performed using only harmonized data. Models for Parkinson's are trained on 50911 harmonized CpGs; models for schizophrenia are trained on 110137 harmonized CpGs. They are chosen based on distributions of methylation levels in controls. For the harmonized data, the Mann-Whitney U-test is performed, and CpG sites are selected for which the p-value >0.05. Such CpG sites have the same distribution of methylation levels in the training data sets from controls.
This approach avoids the situation in which the machine learning model selects CpG sites specific to a particular dataset as the most important features. These CpG sites will be useless for other datasets, where the distribution of methylation levels for them may be different.
This approach minimizes the risk of a situation where the model implicitly tries to solve separate binary classification problems in each individual dataset and finally solves a new multiclass classification problem whose classes correlate with specific datasets (instead of the "case vs control" problem, the "case_dataset_1 vs control_dataset_1 vs case_dataset_2 vs control_dataset_2 vs ..." problem would be solved implicitly).

Comment 9. Imputation of missing values section: it is not clear on which CpGs and on which samples

imputation is performed. Also, it is not clear whether the imputation has been tested on the best-performing model.

Our reply:
The small optimal models built in the "Dimensionality reduction" subsection (for harmonized data) are used for the imputation stage. The LightGBM model with 890 CpG sites is used for Parkinson's disease, and the CatBoost model with 670 CpG sites is used for schizophrenia.
For each model, 100 CpG sites with the highest values are excluded. The number of CpG sites was chosen to cause a significant drop in accuracy and to emphasize the differences in efficiency between the imputation methods. There are no missing features in the resulting small optimal models, so real CpG sites are excluded from consideration.
The different imputation methods are tested on the same datasets as before: GSE72774 for Parkinson's disease, GSE152027, and GSE116379 for schizophrenia. We examine how the accuracy of the "cases vs controls" classification changes when different imputation methods are used relative to the baseline model on data without missing features.
The text of the subsection "Imputation of missing values" is rephrased accordingly.

Minors:
Comment 1. Page 1, line 2: "DNA methylation is associated with epigenetic modification". DNA methylation is an epigenetic mark itself. Do the authors mean histone marks?

Our reply:
Indeed, DNA methylation is an epigenetic modification. This wording is a typo, and it has been corrected.

Comment 2. Page 1, from line 7: "DNA methylation consists of binding a methyl group to cytosine in the cytosine-guanine dinucleotides (CpG sites). Hypermethylation of CpG sites near the gene promoter is known to repress transcription, while hypermethylation in the gene body appears to have an opposite, also less pronounced effect.": references should be added

Our reply:
We followed the suggestion and added references that mention this statement: (Jones, 2012) and (Jjino et. al., 2012).

Comment 3. Page 2, from line 2 : "Current epigenome-wide association studies (EWAS) test DNAm associations with human phenotypes, health conditions and diseases.": references should be added

Our reply:
We followed the suggestion and added references supporting this statement: (Rakyan et. al., 2011), (Liu et. al., 2019) and (Birney et. al., 2016).

Comment 4. Page 3: "In most cases, an increase in dimensionality does not provide significant benefits, since lower dimensionality data may contain more relevant information".
This point could be presented in a reverse way (higher dimensionality data may contain redundant information), introducing the collinearity issue.
In addition, this issue could be introduced before the missing values and imputation section.

Our reply:
We have rephrased the statement about the relationship between the dimensionality of the data and the information it contains.

Comment 5. Page 3: references for "Modern machine-learning-based artificial intelligence systems are powerful and promising tools" could be more specific for the field of epigenetics and DNA methylation.

Our reply:
We have rephrased the statement and added references to works related to the analysis of biomedical data and, in particular, DNA methylation. In this paragraph, we wanted to point out the problem of explainability from a global point of view, as this is a separate problem for any models, not only for DNA methylation analysis.

Close
