# Supplementary material for: Disease classification for whole-blood DNA methylation: Meta-analysis, missing values imputation, and XAI
Source: Gigascience. 2022 Oct 19;11:giac097. doi: 10.1093/gigascience/giac097 (PMC9718659; doi:10.1093/gigascience/giac097)
Supplement: giac097_Response_to_Reviewer_Comments_Revision_1 [file giac097_response_to_reviewer_comments_revision_1.pdf]

Author's Response To Reviewer Comments

Close

Newly developed software DNAmClassMeta is registered on the portals bio.tools and SciCrunch.org, registration numbers are added to the Code availability statement.
RRIDs of the used software libraries have been added to the Methods section.
ORCID IDs have been added for all authors.

Close
