# Supplementary material for: Disease classification for whole-blood DNA methylation: Meta-analysis, missing values imputation, and XAI
Source: Gigascience. 2022 Oct 19;11:giac097. doi: 10.1093/gigascience/giac097 (PMC9718659; doi:10.1093/gigascience/giac097)
Supplement: giac097_Reviewer_1_Report_Original_Submission — Liang Yu -- 6/25/2022 Reviewed [file giac097_reviewer_1_report_original_submission.pdf]

Reviewer Report

Title: Disease classification for whole blood DNA methylation: meta-analysis, missing values imputation, and XAI

Version: Original Submission **Date:** 6/25/2022

Reviewer name: Liang Yu

Reviewer Comments to Author:

The paper by Kalyakulina et al. described the disease classification for whole blood DNA methylation. The author proposed a comprehensive approach of combined DNA methylation datasets to classify controls and patients. The solution includes data harmonization, construction of machine learning classification models, dimensionality reduction of models, imputation of missing values, and explanation of model predictions by explainable artificial intelligence algorithms. For Parkinson's disease and schizophrenia, the author also demonstrates that a method for classifying healthy individuals and patients with various disorders based on whole blood DNA methylation data is an efficient and comprehensive approach. Overall, the manuscript is well organized. I have some suggestions for the authors to improve their work:

1. The manuscript has constructed different models for the prediction study of CpG sites for different types of data. It is suggested to add a flowchart of the whole model construction process to the manuscript so that readers can understand the study more clearly.
2. In Figure 4, the author only shows the top 10 important features and marks the highest accuracy and number of features with black lines in the figure. It is recommended to show the relevant data (optimal accuracy and number of features) in the figure. For the three subplots included in the figure, please label them separately, e.g., A, B, and C to indicate them separately.)
3. Remark concerns model performance evaluation: author should provide standard deviations of the obtained values.
4. In this manuscript, the author used graphs to present the results and suggested that a table summarizing the performance results of the model would be intuitive.
5. I didn't find how the authors optimize the hyper-parameters, usually using grid search.
6. The authors do not adequately address how their method outperforms existing methods in the discussion section.
7. The "Dimensionality reduction" section: I think this section is more appropriately called "feature selection", a sequence forward search method. First sort the features according to their importance values, then add or remove features from a candidate subset while evaluating the criterion.

Methods

Are the methods appropriate to the aims of the study, are they well described, and are necessary controls included? Choose an item.

Conclusions

Are the conclusions adequately supported by the data shown? Choose an item.

Reporting Standards

Does the manuscript adhere to the journal's guidelines on minimum standards of reporting? Choose an item.

Choose an item.

Statistics

Are you able to assess all statistics in the manuscript, including the appropriateness of statistical tests used? Choose an item.

Quality of Written English

Please indicate the quality of language in the manuscript: Choose an item.

Declaration of Competing Interests

Please complete a declaration of competing interests, considering the following questions:

- Have you in the past five years received reimbursements, fees, funding, or salary from an organisation that may in any way gain or lose financially from the publication of this manuscript, either now or in the future?
- Do you hold any stocks or shares in an organisation that may in any way gain or lose financially from the publication of this manuscript, either now or in the future?
- Do you hold or are you currently applying for any patents relating to the content of the manuscript?
- Have you received reimbursements, fees, funding, or salary from an organization that holds or has applied for patents relating to the content of the manuscript?
- Do you have any other financial competing interests?
- Do you have any non-financial competing interests in relation to this paper?

If you can answer no to all of the above, write 'I declare that I have no competing interests' below. If your reply is yes to any, please give details below.

I declare that I have no competing interests

I agree to the open peer review policy of the journal. I understand that my name will be included on my report to the authors and, if the manuscript is accepted for publication, my named report including any attachments I upload will be posted on the website along with the authors' responses. I agree for my

report to be made available under an Open Access Creative Commons CC-BY license (http://creativecommons.org/licenses/by/4.0/). I understand that any comments which I do not wish to be included in my named report can be included as confidential comments to the editors, which will not be published.

Choose an item.

To further support our reviewers, we have joined with Publons, where you can gain additional credit to further highlight your hard work (see: https://publons.com/journal/530/gigascience). On publication of this paper, your review will be automatically added to Publons, you can then choose whether or not to claim your Publons credit. I understand this statement.

Yes Choose an item.
