# Supplementary material for: Disease classification for whole-blood DNA methylation: Meta-analysis, missing values imputation, and XAI
Source: Gigascience. 2022 Oct 19;11:giac097. doi: 10.1093/gigascience/giac097 (PMC9718659; doi:10.1093/gigascience/giac097)
Supplement: giac097_Reviewer_2_Report_Original_Submission — Giulia De Riso -- 6/29/2022 Reviewed [file giac097_reviewer_2_report_original_submission.pdf]

Reviewer Report

Title: Disease classification for whole blood DNA methylation: meta-analysis, missing values imputation, and XAI

Version: Original Submission **Date:** 6/29/2022

Reviewer name: Giulia De Riso

Reviewer Comments to Author:

In this study, a workflow is presented to generate classification models from DNA methylation data. Methods to deal with harmonization and missing data imputation are presented and the benefit of adopting them for classification tasks is tested on case-control datasets of schizophrenia and Parkinson disease. The authors support this workflow with source code.

Although mostly based on already known methodologies, the present study may help orient studies aimed at building and applying DNA methylation based models. However, some major concerns can be raised:

Majors:

In different points of the manuscript, the authors refer to their approach as a pipeline. Indeed, this approach should be composed of sequential modules, in which the output of a module becomes the input of the next one. Although the modules are clearly distinguishable, their organization in the pipeline is less straightforward (also considering that modules can be adopted both to build a model and to use it on new data). The authors could think to draw a scheme of the pipeline, or to adopt a different term to refer to the presented approach.

From the model performance perspective, the ML models poorly perform for schizophrenia. The authors point to inner characteristics of the disease as a possible reason for this. However, this point should be better commented in the Discussion section. Besides this, the impact of the smaller number of samples included in the training set and the higher proportion of imputed features compared to Parkinson disease on the classification accuracy should be discussed. In addition, since the authors provided the code, is there a way to select samples to include in training/test sets based on random choice (classical 70-30% splitting) instead of source dataset?

"For machine learning models, we used only those CpG sites that have the same distribution of methylation levels in different datasets in the control group (methylation levels in the case group typically have greater variability because of disease heterogeneity).": is this filtering performed only on the datasets included in the training set, or also on the test set? It seems the former, but the authors should clearly state this point.

Accuracy with weighted averaging should be defined with a formula in the methods section

Regarding the ML models, the authors chose different types of decision-trees ensemble, along with a deep learning one. They should contextualize this choice (why different models from the same family?). In addition, ML models built on DNA methylation are often based on elastic net or Support-Vector Machines, which are not accounted for in this work. The authors should comment on this aspect in

limitations, and state whether the code they provided for their approach could be customized to adopt different models from the ones they presented.
Regarding the Imputation Method column in Table 2, the meaning is not clear. Are the different imputation methods described in the Imputation of missing values section paired with the ML models presented in Table 2? If yes, some of the methods (like KNN) are missing.
In the harmonization section, Models for case-control classification are trained on different numbers and sets of CpGs. To assess the effect of harmonization alone, the number of CpGs should be instead fixed. This is especially critical for schizophrenia, when the number of features for the non-harmonized data is 35145 whereas the one for harmonized data is 110,137.
Dimensionality reduction section: are the models from imputed and not-imputed data trained only on harmonized data? And how the set of 50911CpG sites for Parkinson and 110137 CpG sites for schizophrenia is selected?
Imputation of missing values section: it is not clear on which CpGs and on which samples imputation is performed. Also, it is not clear whether the imputation has been tested on the best-performing model.
Minors:
Page 1, line 2: "DNA methylation is associated with epigenetic modification". DNA methylation is an epigenetic mark itself. Do the authors mean histone marks?
Page 1, from line 7: "DNA methylation consists of binding a methyl group to cytosine in the cytosine-guanine dinucleotides (CpG sites). Hypermethylation of CpG sites near the gene promoter is known to repress transcription, while hypermethylation in the gene body appears to have an opposite, also less pronounced effect.": references should be added
Page 2, from line 2 : "Current epigenome-wide association studies (EWAS) test DNAm associations with human phenotypes, health conditions and diseases.": references should be added
Page 3: "In most cases, an increase in dimensionality does not provide significant benefits, since lower dimensionality data may contain more relevant information".
This point could be presented in a reverse way (higher dimensionality data may contain redundant information), introducing the collinearity issue.
In addition, this issue could be introduced before the missing values and imputation section.
Page 3: references for "Modern machine-learning-based artificial intelligence systems are powerful and promising tools" could be more specific for the field of epigenetics and DNA methylation.

Methods

Are the methods appropriate to the aims of the study, are they well described, and are necessary controls included? Choose an item.

Conclusions

Are the conclusions adequately supported by the data shown? Choose an item.

Reporting Standards

Does the manuscript adhere to the journal's guidelines on [minimum standards of reporting?]() Choose an item.

Choose an item.

Statistics

Are you able to assess all statistics in the manuscript, including the appropriateness of statistical tests used? Choose an item.

Quality of Written English

Please indicate the quality of language in the manuscript: Choose an item.

Declaration of Competing Interests

Please complete a declaration of competing interests, considering the following questions:

- Have you in the past five years received reimbursements, fees, funding, or salary from an organisation that may in any way gain or lose financially from the publication of this manuscript, either now or in the future?
- Do you hold any stocks or shares in an organisation that may in any way gain or lose financially from the publication of this manuscript, either now or in the future?
- Do you hold or are you currently applying for any patents relating to the content of the manuscript?
- Have you received reimbursements, fees, funding, or salary from an organization that holds or has applied for patents relating to the content of the manuscript?
- Do you have any other financial competing interests?
- Do you have any non-financial competing interests in relation to this paper?

If you can answer no to all of the above, write 'I declare that I have no competing interests' below. If your reply is yes to any, please give details below.

I declare that I have no competing interests

I agree to the open peer review policy of the journal. I understand that my name will be included on my report to the authors and, if the manuscript is accepted for publication, my named report including any attachments I upload will be posted on the website along with the authors' responses. I agree for my report to be made available under an Open Access Creative Commons CC-BY license (http://creativecommons.org/licenses/by/4.0/). I understand that any comments which I do not wish to be included in my named report can be included as confidential comments to the editors, which will not be published.

Choose an item.

To further support our reviewers, we have joined with Publons, where you can gain additional credit to further highlight your hard work (see: https://publons.com/journal/530/gigascience). On publication of this paper, your review will be automatically added to Publons, you can then choose whether or not to claim your Publons credit. I understand this statement.

Yes Choose an item.
